# Sotigalimab and/or nivolumab with chemotherapy in first-line metastatic pancreatic cancer: clinical and immunologic analyses from the randomized phase 2 PRINCE trial

**DOI:** 10.1038/s41591-022-01829-9

**Published:** 2022-06-03

**Authors:** Lacey J. Padrón, Deena M. Maurer, Mark H. O’Hara, Eileen M. O’Reilly, Robert A. Wolff, Zev A. Wainberg, Andrew H. Ko, George Fisher, Osama Rahma, Jaclyn P. Lyman, Christopher R. Cabanski, Jia Xin Yu, Shannon M. Pfeiffer, Marko Spasic, Jingying Xu, Pier Federico Gherardini, Joyson Karakunnel, Rosemarie Mick, Cécile Alanio, Katelyn T. Byrne, Travis J. Hollmann, Jonni S. Moore, Derek D. Jones, Marco Tognetti, Richard O. Chen, Xiaodong Yang, Lisa Salvador, E. John Wherry, Ute Dugan, Jill O’Donnell-Tormey, Lisa H. Butterfield, Vanessa M. Hubbard-Lucey, Ramy Ibrahim, Justin Fairchild, Samantha Bucktrout, Theresa M. LaVallee, Robert H. Vonderheide

**Affiliations:** 1 Parker Institute for Cancer Immunotherapy, San Francisco, CA USA; 2 Abramson Cancer Center of the University of Pennsylvania, Philadelphia, PA USA; 3 Memorial Sloan Kettering Cancer Center, New York, NY USA; 4 The University of Texas MD Anderson Cancer Center, Houston, TX USA; 5 University of California, Los Angeles, Los Angeles, CA USA; 6 University of California, San Francisco, San Francisco, CA USA; 7 Stanford University, Stanford, CA USA; 8 Dana-Farber Cancer Institute, Boston, MA USA; 9 Parker Institute of Cancer Immunotherapy at the University of Pennsylvania, Philadelphia, PA USA; 10 Department of Systems Pharmacology and Translational Therapeutics, University of Pennsylvania, Perelman School of Medicine, Philadelphia, PA USA; 11 Institute for Immunology, University of Pennsylvania, Perelman School of Medicine, Philadelphia, PA USA; 12 Biognosys AG, Schlieren, Switzerland; 13 Personalis, Inc., Menlo Park, CA USA; 14 Apexigen, Inc., San Carlos, CA USA; 15 Bristol Myers Squibb, New York, NY USA; 16 Cancer Research Institute, New York, NY USA

**Keywords:** Pancreatic cancer, Cancer immunotherapy, Tumour biomarkers, Pancreatic cancer

## Abstract

Chemotherapy combined with immunotherapy has improved the treatment of certain solid tumors, but effective regimens remain elusive for pancreatic ductal adenocarcinoma (PDAC). We conducted a randomized phase 2 trial evaluating the efficacy of nivolumab (nivo; anti-PD-1) and/or sotigalimab (sotiga; CD40 agonistic antibody) with gemcitabine/nab-paclitaxel (chemotherapy) in patients with first-line metastatic PDAC (NCT03214250). In 105 patients analyzed for efficacy, the primary endpoint of 1-year overall survival (OS) was met for nivo/chemo (57.7%, *P* = 0.006 compared to historical 1-year OS of 35%, *n* = 34) but was not met for sotiga/chemo (48.1%, *P* = 0.062, *n* = 36) or sotiga/nivo/chemo (41.3%, *P* = 0.223, *n* = 35). Secondary endpoints were progression-free survival, objective response rate, disease control rate, duration of response and safety. Treatment-related adverse event rates were similar across arms. Multi-omic circulating and tumor biomarker analyses identified distinct immune signatures associated with survival for nivo/chemo and sotiga/chemo. Survival after nivo/chemo correlated with a less suppressive tumor microenvironment and higher numbers of activated, antigen-experienced circulating T cells at baseline. Survival after sotiga/chemo correlated with greater intratumoral CD4 T cell infiltration and circulating differentiated CD4 T cells and antigen-presenting cells. A patient subset benefitting from sotiga/nivo/chemo was not identified. Collectively, these analyses suggest potential treatment-specific correlates of efficacy and may enable biomarker-selected patient populations in subsequent PDAC chemoimmunotherapy trials.

## Abstract (web summary)

In a randomized phase 2 trial, sotigalimab, a CD40 agonist, did not significantly improve overall survival in patients with previously untreated metastatic pancreatic cancer when combined with chemotherapy or with nivolumab and chemotherapy. Multi-omic exploratory analyses provide insights into immunologic features associated with clinical benefit.

## Main

PDAC remains one of the most intractable challenges in oncology. Pancreatic cancer is predicted to become the second-leading cause of cancer death in the United States by 2030 (ref. ^1^). Although combination chemotherapy reliably offers tumor control and clinical stabilization, both standard regimens of gemcitabine plus nab-paclitaxel and FOLFIRINOX (oxaliplatin, irinotecan, fluorouracil and leucovorin) are limited in response durability and incur toxicity. Thus, there is urgent necessity for new treatment strategies in this disease.

Immune checkpoint inhibition has revolutionized cancer care in the past decade—with now nearly 70 distinct US Food and Drug Administration label indications across more than 18 histologies^2^—but these therapies have yet to show meaningful clinical benefit in PDAC beyond rare (<1%) patients exhibiting microsatellite instability (MSI) in the tumor^3^. Single-agent and combinations of PD-1, PD-L1 or CTLA-4 inhibitors in patients with advanced PDAC are ineffective (objective response rates (ORRs) <5%)^4–6^, including in patients with positive PD-L1 expression, a biomarker that enriches for response in other cancers. Postulated mechanisms of resistance to immunotherapy in PDAC include poor T cell infiltration, low tumor mutational burden and a highly immunosuppressive tumor microenvironment (TME). However, recent in-depth profiling of PDAC tumors indicates that as many as 20–30% of patients exhibit moderate T cell content and that, in some settings, tumor immunogenic neo-epitopes and T cell immunity can correlate with OS^7–9^.

Combinations of gemcitabine and nab-paclitaxel (chemo) with CD40 agonist antibody with or without immune checkpoint inhibition overcome immune suppression in genetically engineered mouse models of PDAC^10,11^. In these experiments, chemotherapy drives the release of cancer cell antigens and induces tumor regression, and survival is dependent on T cells, dendritic cells (DCs) and immunologic memory—justifying testing of these strategies in clinical trials. In our recent phase 1b study of the CD40 agonist antibody sotigalimab (sotiga) with chemo, with or without nivolumab (nivo), we reported acceptable toxicity and promising rates of tumor regressions in patients with newly diagnosed metastatic PDAC (mPDAC)^12^.

Here we report clinical and translational results of a randomized, multi-center, open-label phase 2 trial for first-line treatment of patients with mPDAC randomized to receive nivo/chemo, sotiga/chemo or sotiga/nivo/chemo. The clinical study was accompanied by comprehensive and serial biospecimen acquisition and multi-omic profiling, allowing for hypothesis-generating exploratory analyses that identified multiple distinct and treatment-specific biomarkers.

## Results

### Trial design and patient characteristics

**Fig. 1:**
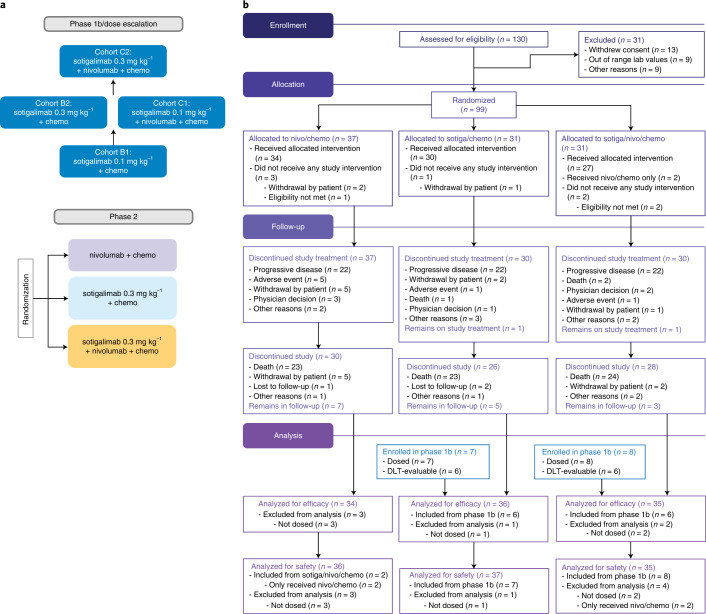
PRINCE study design and CONSORT diagram. **a**, PRINCE was a seamless phase 1b/2 study, with the phase 2 portion randomizing patients to treatment with nivo/chemo, sotiga/chemo or sotiga/nivo/chemo. **b**, CONSORT diagram of the phase 2 portion of the study. Patients enrolled in cohorts B2 and C2 during phase 1b were included in safety and/or efficacy analyses of the phase 2 portion.

**Extended Data Fig. 1:**
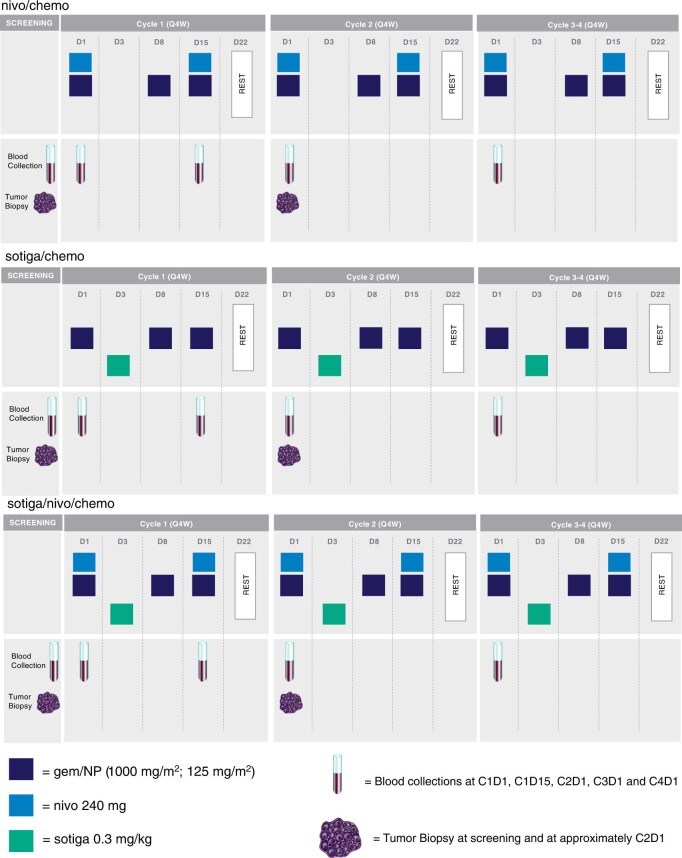
PRINCE schema for dosing and sample collection schedule. Schema shows standard dosing schedule and relevant sample collection timepoints for each treatment arm. All drugs were given intravenously. The study protocol provides additional details on allowable modifications to the dosing schedule.

From 30 August 2018 through 10 June 2019, 99 patients were randomly allocated into one of three treatment arms (*n* = 37, 31 and 31 to nivo/chemo, sotiga/chemo and sotiga/nivo/chemo, respectively; Fig. 1 and Extended Data Fig. 1). Six patients (*n* = 3, 1 and 2, respectively) were randomized but not dosed and were excluded from analysis (Fig. 1). Efficacy was assessed for 105 patients (*n* = 34, 36 and 35), which included 93 patients randomized and dosed in phase 2 and 12 dose-limiting toxicity (DLT)-evaluable patients from the non-randomized phase 1b study^12^ (six each on sotiga/chemo and sotiga/nivo/chemo). Safety was assessed for 108 patients (*n* = 36, 37 and 35, respectively), which included the 105 patients assessed for efficacy plus three non-DLT-evaluable patients from phase 1b. The cutoff date for clinical data analysis was 24 March 2021.

Baseline characteristics for the efficacy population were generally balanced across arms (Table 1 and Supplementary Table 1). However, a higher proportion of patients on sotiga/chemo had an Eastern Cooperative Oncology Group (ECOG) performance status score of 0 at screening (56% versus 43–44%). Across arms, 74–79% of patients had de novo stage IV disease.

**Table 1:** Demographic and baseline disease characteristics for patients in the efficacy population

	nivo/chemo (*n* = 34)	sotiga/chemo (*n* = 36)	sotiga/nivo/chemo (*n* = 35)
Characteristic			
Age, years			
Median (range)	62.5 (47–75)	60.5 (35-78)	62.0 (41–78)
≥65 years, *n* (%)	14 (41)	14 (39)	13 (37)
Sex, *n* (%)			
Female	14 (41)	13 (36)	16 (46)
Male	20 (59)	23 (64)	19 (54)
Race and ethnic group, *n* (%)			
Asian	3 (9)	4 (11)	0
Black	0	3 (8)	2 (6)
White	29 (85)	28 (78)	31 (89)
Other	2 (6)	1 (3)	2 (6)
Hispanic	1 (3)	1 (3)	1 (3)
ECOG performance status score, *n* (%)			
0	15 (44)	20 (56)	15 (43)
1	19 (56)	16 (44)	20 (57)
Pancreatic tumor location, *n* (%)			
Head	14 (41)	17 (47)	19 (54)
Body	12 (35)	9 (25)	10 (29)
Tail	8 (24)	10 (28)	6 (17)
Select sites of metastatic disease, *n* (%)			
Liver	28 (82)	29 (81)	27 (77)
Lung	10 (29)	10 (28)	11 (31)
Peritoneum	8 (24)	9 (25)	11 (31)
Stage at initial PDAC diagnosis, *n* (%)			
Stages I−III	7 (21)	9 (25)	9 (26)
Stage IV	27 (79)	27 (75)	26 (74)
Time from diagnosis to first dose—months, median (range)^a^	1.1 (0.4–69.8)	1.0 (0.4–29.1)	1.1 (0.4–45.3)
Prior cancer treatment, *n* (%)			
Chemotherapy	9 (27)	7 (19)	6 (17)
Radiation therapy	7 (21)	1 (3)	4 (11)
Surgery	11 (32)	11 (31)	8 (23)
Tumor burden (RECIST), mm^b^			
Median	78.5	68.5	79.0
Range	13–160	19–214	10–194

The efficacy population includes all randomized and dosed patients in phase 2 and DLT-evaluable patients from phase 1b enrolled at the recommended phase 2 dose of sotiga.

^a^Calculations exclude one participant from nivo/chemo who did not report a date of diagnosis.

^b^Tumor burden is the sum of the largest diameters of all target lesions (shortest diameter for lymph nodes).

Pre-treatment PD-L1^+^ tumor percentages were similar between the nivo/chemo and sotiga/nivo/chemo arms but less in the sotiga/chemo arm (Supplementary Table 2). Sixty-three (60%) patients had pre-treatment tumor tissue of high enough quality for whole-exome sequencing (WES). By WES, treatment arms were balanced for somatic mutation frequencies in *KRAS*, *SMAD4* and *TP53* in mPDAC (Supplementary Table 2). The tumor tissue for one patient (in nivo/chemo) was MSI-high. Only one patient (in sotiga/nivo/chemo) had a pathogenic tumor variant of BRCA2. The BRCA variant detected in the tumor tissue was the indel tumor variant BRCA2c.5946delT; this patient experienced a partial response but withdrew consent after 2.8 months, and their 1-year OS status is unknown. Additionally, the arms were relatively balanced for gene expression signatures in pre-treatment tumor tissues and had similar baseline frequencies of immune cell populations within circulation.

At the time of analysis, median duration of follow-up for patients in the efficacy population was 24.2 months (interquartile range (IQR), 20.5–26.3) with 15 months of minimum follow-up. Two patients remained on treatment, one each on sotiga/chemo and sotiga/nivo/chemo. Median time on treatment was similar between the three arms (median (IQR), months: 5.2 (1.9–8.1), 5.1 (3.4–8.9) and 4.7 (2.4–7.9) for nivo/chemo, sotiga/chemo and sotiga/nivo/chemo, respectively). Exposure to each drug in the combination was also similar between arms (Supplementary Table 3).

### Clinical activity

The primary endpoint was 1-year OS rate for each arm versus a historical control rate of 35%^13^. Secondary endpoints included progression-free survival (PFS), duration of response (DOR), investigator-assessed ORR and disease control rate (DCR). This study was not powered for comparison between arms. The survival analysis was based on 78 (74%) deaths.

**Fig. 2:**
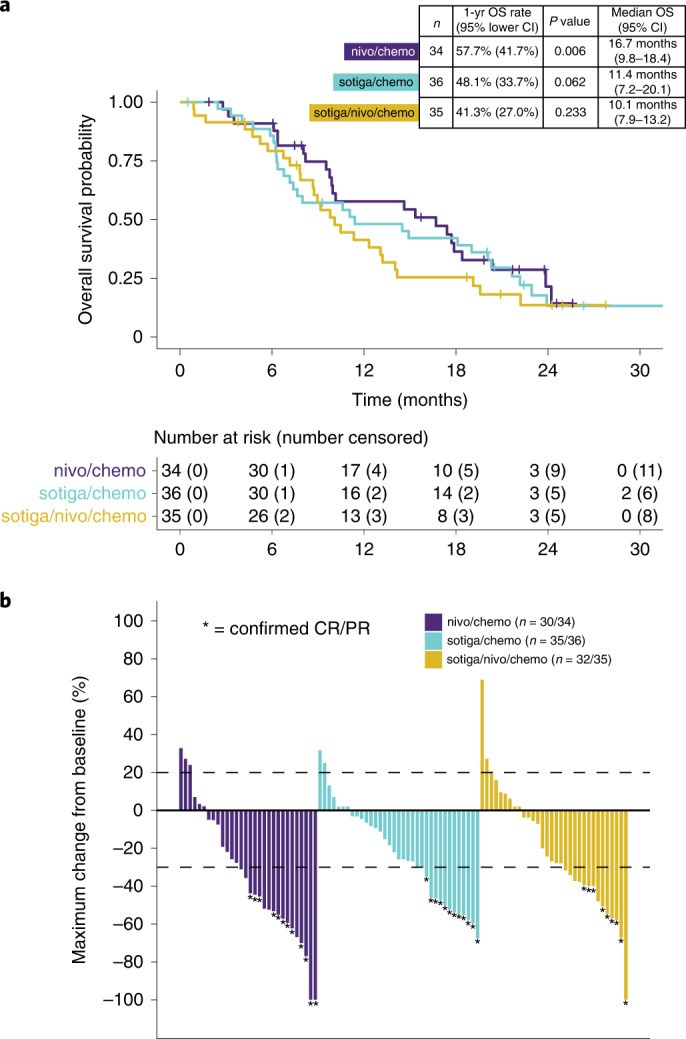
OS and tumor response. **a**, Kaplan–Meier curves of OS of patients in the efficacy population. The 1-year OS rate and corresponding one-sided, 95% lower confidence bound were estimated by the Kaplan–Meier method. *P* values were calculated using a one-sided, one-sample *z-*test of the Kaplan–Meier estimate of the 1-year OS rate (and its standard error) against the historical rate of 35%. *P* values were not adjusted for multiple comparisons. Median OS and corresponding two-sided, 95% CI were estimated by the Kaplan–Meier method. **b**, Maximum percentage change from baseline in the sum of the diameters of the target lesions for each patient with at least one post-baseline tumor assessment. Four patients in the nivo/chemo arm, one in the sotiga/chemo arm and three in the sotiga/nivo/chemo arm did not have any post-baseline tumor assessments. Confirmed CR or PR is defined as two consecutive tumor assessments at least 4 weeks apart with an overall response of CR/PR.

**Extended Data Fig. 2:**
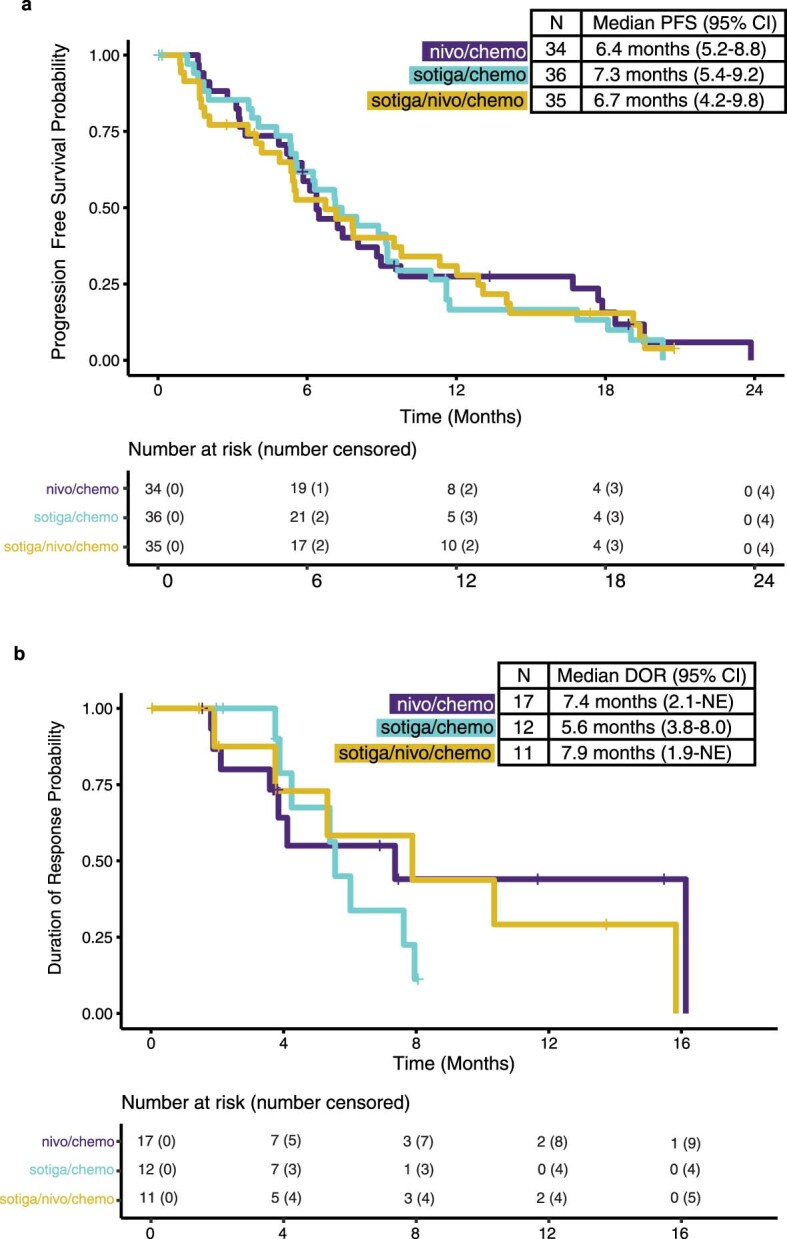
Kaplan-Meier curves of progression-free survival and duration of response. **a**, Progression-free survival (PFS) of patients in the efficacy population. **b**, Duration of response (DOR) of patients in the efficacy population who had a partial or complete response. PFS, DOR and the corresponding 2-sided 95% confidence interval (CI) were estimated by the Kaplan-Meier method. CI = confidence interval; NE = not estimable.

For nivo/chemo, the 1-year OS rate was 57.7% (one-sided *P* = 0.006, one-sided 95% lower confidence bound = 41.7%), and median OS was 16.7 months (95% confidence interval (CI): 9.8–18.4) (Fig. 2a). Median PFS was 6.4 months (95% CI: 5.2–8.8); ORR was 50% (95% CI: 32–68); DCR was 74% (95% CI: 56–87); and median DOR was 7.4 months (95% CI: 2.1–not estimable) (Fig. 2b, Extended Data Fig. 2 and Extended Data Table 1).

For sotiga/chemo, the 1-year OS rate was 48.1% (one-sided *P* = 0.062, one-sided 95% lower confidence bound = 33.7%), and median OS was 11.4 months (95% CI: 7.2–20.1). The median PFS was 7.3 months (95% CI: 5.4–9.2); investigator-assessed ORR was 33% (95% CI: 19–51); DCR was 78% (95% CI: 61–90); and median DOR was 5.6 months (95% CI: 3.8–8.0).

For sotiga/nivo/chemo, the 1-year OS rate was 41.3% (one-sided *P* = 0.233, lower confidence bound=27.0%), and median OS was 10.1 months (95% CI: 7.9–13.2). The median PFS was 6.7 months (95% CI: 4.2–9.8); investigator-assessed ORR was 31% (95% CI: 17–49); DCR was 69% (95% CI: 51–83); and median DOR was 7.9 months (95% CI: 1.9–not estimable).

The use of subsequent systemic therapy was balanced between arms (63–67%), with chemotherapy being the most reported subsequent therapy. Post hoc subgroup analyses of baseline clinical characteristics revealed numerically improved OS in several patient subgroups, including patients initially diagnosed with stage I–III disease (Supplementary Table 4). However, the data suggest that the survival benefit observed in patients receiving nivo/chemo was not driven solely by patient imbalances in these subgroups nor MSI-high (OS = 8.1 months in a single MSI-high patient), KRAS wild-type (balanced across arms) or BRCA.

### Safety

The spectrum, frequency and severity of treatment-related adverse events (TRAEs), a secondary endpoint, were similar across the arms and consistent with the safety profile observed in phase 1b^12^. Overall, 106 (98%) patients reported at least one TRAE. The most common non-hematologic TRAEs of any grade were nausea, fatigue, pyrexia and chills (Extended Data Table 2). The most common grade 3–4 TRAEs were hematologic and generally transient in nature.

Adverse events of special interest (AESIs), including cytokine release syndrome (CRS), infusion reactions, thrombocytopenia and elevated liver function tests (LFTs), were observed in 92 (87%) patients (Supplementary Table 5). CRS was observed in 0, 9 (24%) and 12 (34%) patients in nivo/chemo, sotiga/chemo and sotiga/nivo/chemo, respectively, with five events assessed as grade 3 (three in sotiga/chemo and two in sotiga/nivo/chemo). Grade 4 or 5 CRS was not observed. Elevated LFTs were observed in 24 (67%), 30 (81%) and 26 (74%) patients, respectively. Infusion-related reactions were observed in 2 (6%), 5 (14%) and 5 (14%) patients, respectively. Thrombocytopenia occurred in 18 (50%), 21 (57%) and 22 (63%) patients, respectively, with 18 patients experiencing a grade 3 or 4 event (five (14%), six (16%) and seven (20%), respectively).

Six (17%) patients on nivo/chemo, one (3%) on sotiga/chemo and one (3%) on sotiga/nivo/chemo discontinued all study drugs due to an adverse event; most were grade 2 or grade 3, with one grade 4 (thrombic microangiopathy on nivo/chemo), and half were assessed by the investigator as related to chemotherapy only (Supplementary Table 6). Two patients died due to an adverse event: acute hepatic failure on sotiga/chemo (causality could not be determined so considered possibly related to all study drugs) and intracranial hemorrhage on sotiga/nivo/chemo (possibly related to all study drugs).

### Pharmacodynamic effects

As an exploratory trial endpoint, pharmacodynamic effects and potential underlying immune mechanisms were studied via multi-omic profiling of serial patient blood samples and tumor biopsies obtained pre-treatment and on-treatment (Extended Data Fig. 1). For all evaluable patients, tumor samples were profiled with RNA sequencing and multiplex immunofluorescence (mIF), whereas blood samples were profiled with high-dimensional flow cytometry (X50), mass cytometry time of flight (CyTOF) and serum protein profiling via Olink panels (see respective Methods sections for total sample numbers).

**Extended Data Fig. 3:**
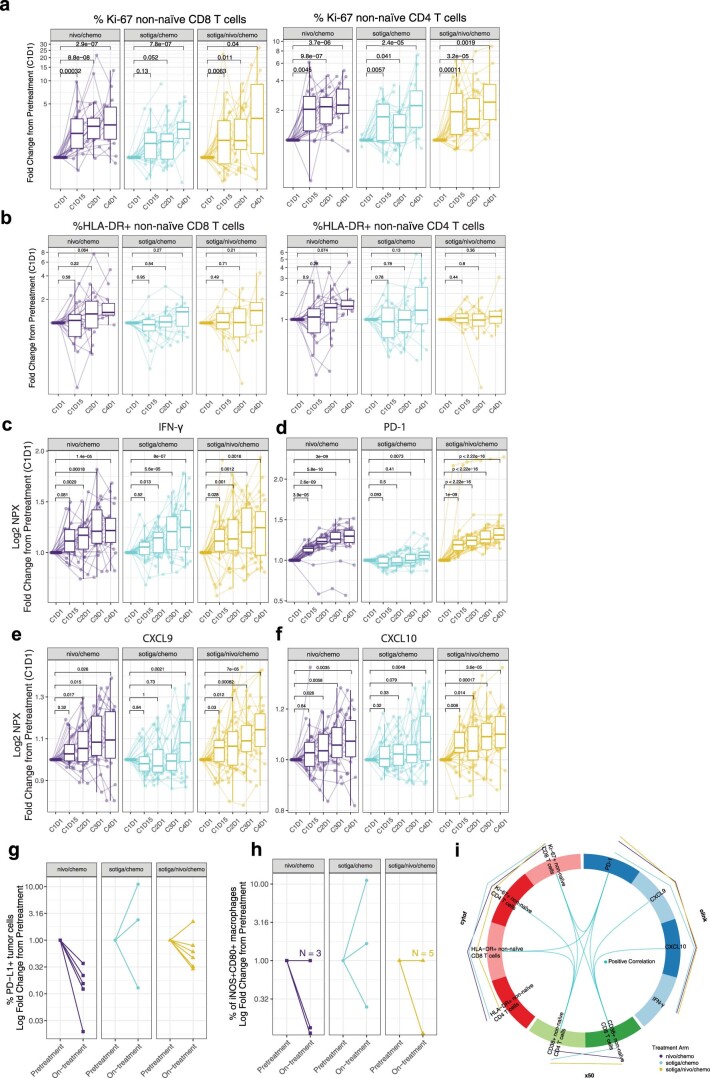
Biomarker signatures in blood and tumor reveal specific immune mechanisms of activation in response to nivo/chemo and sotiga/chemo treatment in patients with mPDAC. **a**, Change in frequencies of circulating Ki-67+ non-naïve CD8 (left panel) and CD4 (right panel) T cells, as a fraction of total non-naïve CD8 or CD4 T cells respectively, in patients from each arm over the course of treatment by flow cytometry. **b**, Change in frequencies of circulating HLA-DR+ non-naïve CD8 (b, left panel) and CD4 (b, right panel) T cells, as a fraction of total non-naïve CD8 or CD4 T cells respectively, in patients from each arm over the course of treatment by Cytof. **c,d,e,f**, Change in Log2 expression of circulating IFN-γ (c), PD-1 (d), CXCL9 (e) and CXCL10 (f) from pretreatment values from each arm by Olink analysis. Timeseries box plots in a-f are shown as fold change relative to C1D1 and plotted on a pseudo-log scale. Median values and quartiles are shown. The whiskers depict 95% CI. Individual patient values are shown in thin lines and colored by survival status at 1 year. P-values represent two-sided Wilcoxon signed-rank tests between timepoints, illustrating increases on-treatment. **g, h**, Frequencies of PD-L1+ tumor cells (g) and intratumoral iNOS+CD80+ macrophages (h) from mIF of on-treatment biopsies (C2D1 when feasible, see methods for details), shown as a fold change relative to pretreatment biopsy for each arm. **i**, DIABLO Circos plot showing results of integrative analysis where select factors from CyTOF, X50 flow cytometry and Olink, significantly associated with on-treatment (C2D1) effects and correlations among these factors and treatment arms. In the Circos plot, lines outside the circle indicate magnitude and direction of treatment association (the further distance from the circle, the greater the association). Lines inside the plot indicate positive (blue) correlations between biomarker factors. For all cell populations shown, frequencies are out of parent population. Sample sizes for all cell populations identified through CyTOF analysis (**b**): n = 25, 20, 23, 13; n = 29, 23, 24, 22; n = 26, 20, 26, 13 biologically independent samples at C1D1, C1D15, C2D1 and C4D1 in nivo/chemo, sotiga/chemo, sotiga/nivo/chemo treatment arms, respectively. Sample sizes for all cell populations identified through flow cytometry analysis (**a**): n = 26, 21, 25, 19; n = 28, 23, 27, 18; n = 32, 27, 29, 14 biologically independent samples at C1D1, C1D15, C2D1 and C4D1 in nivo/chemo, sotiga/chemo, sotiga/nivo/chemo treatment arms, respectively. Sample sizes for all soluble proteins identified through proteomic analysis (**c,d,e,f**): n = 32, 25, 27, 25, 23; n = 36, 29, 31, 25, 27; n = 35, 27, 32, 26, 25 biologically independent samples at C1D1, C1D15, C2D1, C3D1, and C4D1 in nivo/chemo, sotiga/chemo, sotiga/nivo/chemo treatment arms, respectively. Sample sizes for all cell populations identified through mIF (**g,h**): n = 5, 3, 6, biologically independent matched paired samples at C1D1 and approximately C2D1 in nivo/chemo, sotiga/chemo, sotiga/nivo/chemo treatment arms, respectively. (**i**): n = 22, 23, 21 biologically independent matched samples at C2D1 in nivo/chemo, sotiga/chemo, sotiga/nivo/chemo treatment arms respectively for CyTOF, X50 flow cytometry and Olink integrative analysis.

In all three arms, longitudinal profiling of peripheral blood mononuclear cells (PBMCs) revealed increases in proliferating (Ki-67^+^) non-naive CD8 and CD4 T cells on-treatment (Extended Data Fig. 3a, Supplementary Fig. 1a and Supplementary Table 7). This increase was strongest and observed earlier in the nivo-containing arms and, to a lesser extent, in sotiga/chemo. Patients treated with nivo/chemo also had increases in circulating activated (HLA-DR^+^) non-naive CD4 and CD8 T cells (Extended Data Fig. 3b and Supplementary Fig. 1b). Additionally, increases in circulating T cells expressing other activation markers, such as CD38, were observed in all treatment arms (Supplementary Fig. 1c).

To evaluate circulating proteins known to associate with immune and inflammatory activities, an array of 172 serum proteins was analyzed. Treatment of patients in all three arms resulted in significant increases in IFN-γ (Extended Data Fig. 3c). Additionally, soluble PD-1 (sPD-1) levels increased on-treatment in sera from patients in nivo-containing arms. In contrast, sPD-1 levels remained relatively consistent in sera from patients treated with sotiga/chemo (Extended Data Fig. 3d). Consistent with known pharmacodynamic effects of immunotherapy treatment^14^, several chemokines increased in the sera in response to all treatments, including CXCL9 and CXCL10 (Extended Data Fig. 3e,f). However, earlier increases (C1D15 and C2D1) were observed in the nivo-containing arms (Extended Data Fig. 3e,f).

To evaluate whether any biologic associations emerged from the circulating orthogonal biomarker assays, integrated analysis of all features measured in response to chemoimmunotherapy treatment (C2D1) was performed. This analysis revealed correlations between proteins and cell populations across different platforms (Extended Data Fig. 3i). Of note, CXCL10, CXCL9 and sPD-1 correlated with activated, proliferating T cells in all treatment arms. Changes in CD38^+^ non-naive CD8 T cells, sPD-1 and CXCL9 were associated with the nivo-containing arms (Extended Data Fig. 3i).

Analysis of paired pre-treatment and on-treatment (~C2D1) biopsies from individual patients revealed that nivo/chemo treatment led to a numerically decreased percentage of tumor cells expressing PD-L1 in all samples measured (*n* = 5). In contrast, changes in the percentage of PD-L1^+^ tumor cells were heterogeneous for sotiga/chemo (*n* = 3). The combination of sotiga/nivo/chemo decreased PD-L1^+^ tumor cells in five of six patients analyzed (Extended Data Fig. 3g). For sotiga/chemo, two of three patients with paired biopsies exhibited increases in tumor-infiltrating iNOS^+^CD80^+^CD68^+^ cells (macrophages), an effect that was not observed for paired biopsies from patients treated in nivo-containing arms (Extended Data Fig. 3h). The observed pharmacodynamic effects in both circulation and the TME highlight immune modulation with immunotherapy/chemotherapy combinations in patients with mPDAC.

### Assessment of correlates of clinical benefit

To identify subsets of patients who are more likely to benefit from a specific treatment, we performed exploratory, hypothesis-generating analyses using comprehensive multi-omic, multi-parameter immune and tumor biomarker data for associations with survival. An approach of focusing on biological networks indicated across multiple assays helped to identify signals of underlying systems that are more likely to have robustness in the context of a small phase 2 study. This deep, integrated analysis approach provided a comprehensive view of tumor and immune contexture and identified distinct biomarkers that associated with survival benefit in each arm (Supplementary Table 8). These associations frequently remained when accounting for the clinical covariates of initial stage at diagnosis and prior chemotherapy treatment (Supplementary Table 8). Although statistical tests were used to evaluate biomarkers associated with survival, the associated *P* values were not adjusted for multiplicity as this is a post hoc, exploratory analysis. The aim of these statistical tests was to assist in ranking and identifying potential biomarker candidates that could be targets in a prospective study; the magnitude of the *P* values should not be interpreted.

It is important to note that, due to the effect on tissue of origin on bulk RNA sequencing, we chose to limit all tumor gene expression analyses to the most common biopsy site—liver metastases—which constituted 64% of biopsies. Tissue origin did not affect major immune population frequencies observed by mIF, and, because of this and the smaller numbers of biopsies profiled via mIF, we did not limit analyses by biopsy site for immunophenotyping.

### Correlates of survival benefit after nivo/chemo

**Fig. 3:**
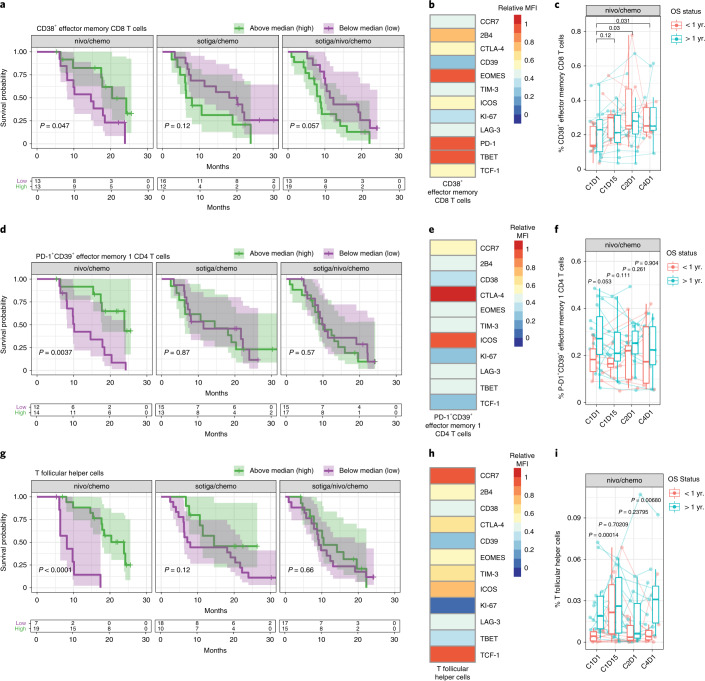
Activated, antigen-experienced non-naive T cells and T_fh_ cells in the periphery are associated with survival in patients with mPDAC treated with nivo/chemo. **a**, Kaplan–Meier curves for OS stratified by frequencies of circulating CD38^+^ EM CD8 T cells by flow cytometry, pre-treatment (C1D1) above and below the median frequency. **b**, Heat map of relative median fluorescence intensity of markers on CD38^+^ EM CD8 T cells from pre-treatment PBMC samples across patients in the nivo/chemo arm. **c**, Frequencies of CD38^+^ EM CD8 T cells pre-treatment (C1D1) and on-treatment PBMC samples (C1D15, C2D1 and C4D1), grouped by patient survival status at 1 year. **d**, Kaplan–Meier curves for OS stratified by frequencies of circulating PD-1^+^CD39^+^ EM1 CD4 T cells. **e**, Heat map of relative median fluorescence intensity of markers present on PD-1^+^CD39^+^ EM1 CD4 T cell population from pre-treatment PBMC samples across patients in the nivo/chemo arm. **f**, Frequencies of PD-1^+^CD39^+^ EM1 CD4 T cells in pre-treatment (C1D1) and on-treatment PBMC samples (C1D15, C2D1 and C4D1), grouped by patient survival status at 1 year. **g**, Kaplan–Meier curves for OS stratified by frequencies of circulating T_fh_ (CXCR5^+^PD-1^+^CD4^+^) cells. **h**, Heat map of relative median fluorescence intensity of markers present on pre-treatment T_fh_ cells across all patients from pre-treatment PBMC samples in the nivo/chemo arm. **i**, Frequencies of T_fh_ cells pre-treatment and on-treatment (C1D15, C2D1 and C4D1). For all cell populations shown, frequencies are out of parent population. Box plots show median and quartiles, and whiskers depict 95% CI. Individual patient values are shown in thin lines. Color depicts survival status at 1 year. *P* values for time series represent two-sided Wilcoxon signed-rank tests between time points, illustrating changes on-treatment (**c**) or survival groups at each time point (**f** and **i**). On Kaplan–Meier curves, median values were determined using all data across the three arms; *P* values are from a log-rank test between groups; and shaded regions illustrate 95% CI. Sample sizes for cell populations are shown (**c**, **f** and **i**): *n* = 26, 21, 25 and 19 biologically independent samples at C1D1, C1D15, C2D1 and C4D1, respectively.

Survival benefit after nivo/chemo was associated with a diverse, immunocompetent circulating T cell response pre-treatment. CD4 and CD8 T cells were classified as non-naive, central memory (CM) or effector memory (EM). EM T cells were further subdivided based on CCR7 expression: EM1, EM2 and EM3 (refs. ^15,16^) (Supplementary Fig. 2 and Supplementary Table 7). Higher frequencies of activated (CD38^+^) EM CD8 T cells were associated with longer survival (Fig. 3a). These cells co-expressed PD-1, 2B4, Eomes and Tbet (Fig. 3b). Although this cell population became more abundant with treatment, only pre-treatment levels were associated with 1-year survival status (Fig. 3c). Similarly, antigen-experienced (PD-1^+^CD39^+^) EM1 (Fig. 3d) and CM CD4 T cells (Supplementary Fig. 3a) were associated with longer survival. These cells co-expressed CTLA-4 and ICOS (Fig. 3e and Supplementary Fig. 3b). Co-expression of CCR7 and TCF-1 was unique to antigen-experienced (PD-1^+^CD39^+^) CM CD4 T cells (Supplementary Fig. 3b). Patients with >1 year survival expressed numerically higher frequencies of this cellular phenotype on-treatment (Fig. 3f and Supplementary Fig. 3c). In addition, T follicular helper (T_fh_) cells (CD4^+^PD-1^+^CXCR5^+^) were associated with longer survival (Fig. 3g) and had the highest predictive value of the strongest circulating biomarkers in the nivo/chemo arm in a combined multivariable model (Supplementary Fig. 4a). These cells had high expression of TCF-1, CCR7 and ICOS (Fig. 3h). High frequencies of these cells late on-treatment (C4D1) were most differentiating between patients with >1 year and <1 year OS (Fig. 3i).

**Extended Data Fig. 4:**
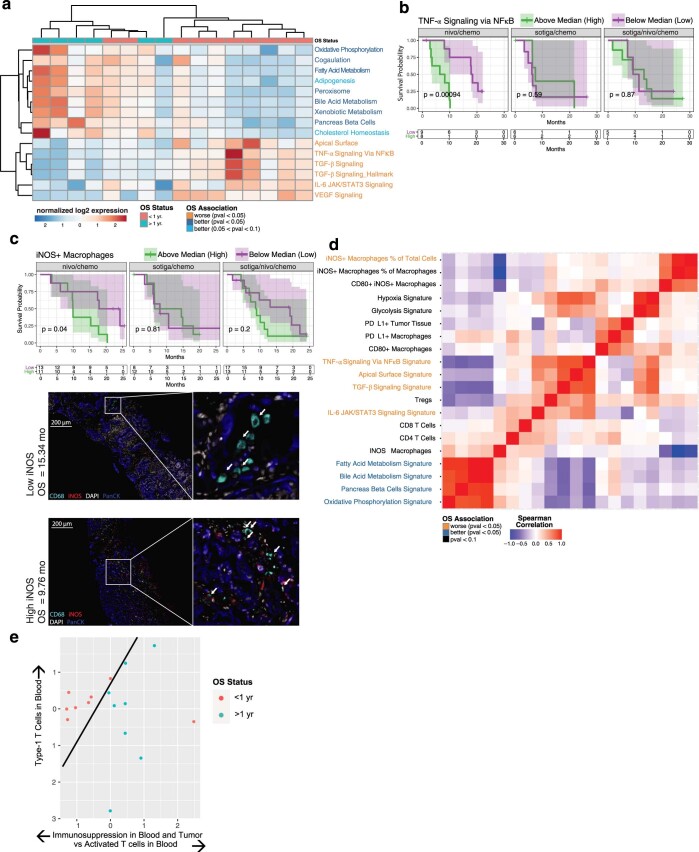
A non-immunosuppressive tumor microenvironment and activated circulating CD8 T cells before treatment are associated with survival in mPDAC patients treated with nivo/chemo. **a**, Heatmap showing results of unsupervised clustering of gene expression signatures and survival status in the nivo/chemo arm. Individual patients are shown in columns and annotated by survival status at 1 year to illustrate association. Gene expression signature labels are color coded based on survival association by log-rank test. KM curves for overall survival stratified by median values at baseline of **b**, TNF-α via NFκB hallmark pathway signature score and **c**, Percentage of iNOS+ intratumoral macrophages out of total cells from mIF (c, top panel). Representative pretreatment tumor mIF images showing iNOS+ cells from two patients with labels showing marker grouping (low = below median, high= above median) and individual patient survival (c, bottom panel). **d**, Spearman correlation matrix of tumor immune populations and gene expression signatures in pretreatment tumor biopsies, with labels color coded by association with survival association by log-rank test. Note the Y-axis labels are to be repeated along the X-axis (bottom to top on Y-axis corresponding to left to right on X-axis). **e**, Multi-omic dimensionality reduction of circulating factors and tumor data using Independent Component Analysis, with each dot representing a single patient colored by survival status at one year and with position determined by reduced dimensionality across all tumor and circulating biomarkers. Black separating line serves to illustrate a separation and is not computationally derived. On all KM curves, median values were determined using all data across the 3 arms, P-values are from a log-rank test between groups, and shaded regions illustrate 95% CI.

We identified 15 gene expression signatures that associated with survival (*P* < 0.1) in the nivo/chemo arm and used unsupervised clustering to group patients by expression of these signatures to study associations with 1-year OS (Extended Data Fig. 4a and Supplementary Table 8). Among these signatures, higher expression of genes associated with oxidative phosphorylation, fatty acid metabolism, xenobiotic metabolism and bile acid metabolism were associated with longer survival, whereas higher expression of TGF-β, TNF-α signaling via NFκB and IL6/JAK STAT3 gene signatures were associated with shorter survival (Extended Data Fig. 4a). The association with TNF-α signaling via NFκB and survival was unique to the nivo/chemo arm (Extended Data Fig. 4b). Patients with lower frequencies of tumor-infiltrating iNOS^+^ macrophages also had longer survival after nivo/chemo (Extended Data Fig. 4c). Higher frequencies of PD-L1^+^ tumor cells had a weak association with more than 1-year survival (Supplementary Fig. 5). Intratumoral metabolic gene expression signatures displayed a negative correlation with TGF- β and TNF-α signatures (Extended Data Fig. 4d).

Multi-omic dimensionality reduction analysis of both circulating and tumor factors recapitulated these findings and revealed the primary axes of independent variance in the data, showing a separation between patients with survival >1 year and <1 year (Extended Data Fig. 4e). Overall, patients with longer survival after nivo/chemo had lower pre-treatment immunosuppressive molecules and higher pre-treatment frequencies of activated, type-1 (Tbet^+^) T cells (Extended Data Fig. 4e and Supplementary Table 8).

### Correlates of survival benefit after sotiga/chemo

**Fig. 4:**
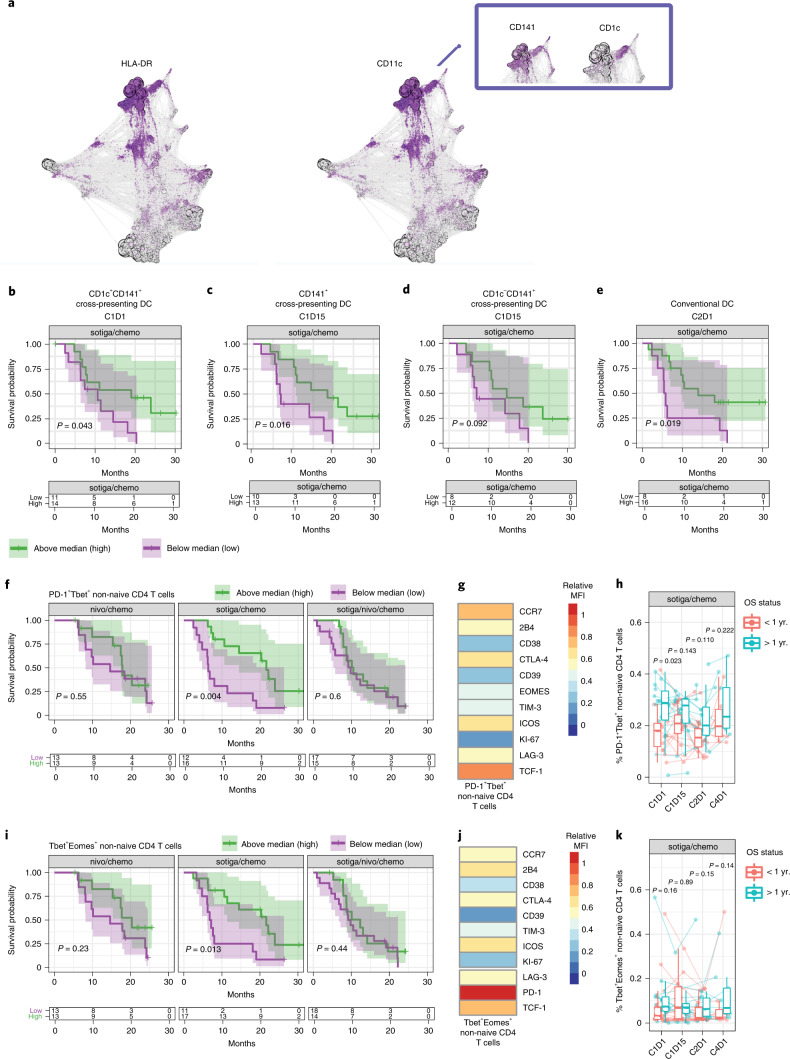
Cross-presenting, activated APCs and type-1 helper T cells in circulation associate with survival in patients receiving sotiga/chemo treatment. **a**, Force-directed graph visualization of unsupervised clustering of cells from CyTOF across all patients and time points, with callout box of DC phenotypes associating with survival and followed up on with gating analysis in further panels. **b**–**f**, Kaplan–Meier curve for OS stratified by median values. **b**, Circulating CD1c^+^ cross-presenting DCs (CD141^+^) at C1D1 **c**, Cross-presenting DCs (CD141^+^) at C1D15. **d**, CD1c^−^ cross-presenting DCs (CD141^+^) at C1D15. cDCs at C2D1 (**e**) and pre-treatment PD-1^+^Tbet^+^ non-naive CD4 T cells (**f**). **g**, Heat map of pre-treatment median fluorescence intensity of markers present on PD-1^+^Tbet^+^ non-naive CD4 T cells. **h**, Frequencies of PD-1^+^Tbet^+^ non-naive CD4 T cells pre-treatment (C1D1) and on-treatment (C1D15, C2D1 and C4D1), grouped by survival status at 1 year. **i**, Kaplan–Meier curves for OS stratified by frequency of pre-treatment Tbet^+^Eomes^+^ non-naive CD4 T cells. **j**, Heat map of pre-treatment median fluorescence intensity of markers present on Tbet^+^Eomes^+^ non-naive CD4 T cells. **k**, Frequencies of Tbet^+^Eomes^+^ non-naive CD4 T cells pre-treatment (C1D1) and on-treatment (C1D15, C2D1 and C4D1), grouped by survival status at 1 year. For DC populations, frequencies are out of total leukocytes. For T cell populations, frequencies are out of parent. Box plots show median and quartiles, and whiskers depict 95% CI. Individual patient values are shown in thin lines and colored by survival status at 1 year. *P* values for time series represent two-sided Wilcoxon signed-rank tests between survival groups at each time point. On Kaplan–Meier curves, median values were determined using all data across the three arms; *P* values are from a log-rank test between groups; and shaded regions illustrate 95% CI. Sample sizes for cell populations (**a**−**e**): *n* = 29, 23, 24 and 22 biologically independent samples at C1D1, C1D15, C2D1 and C4D1, respectively. Sample sizes for cell populations are shown (**f**–**k**): *n* = 28, 23, 27 and 18 biologically independent samples at C1D1, C1D15, C2D1 and C4D1, respectively.

**Extended Data Fig. 5:**
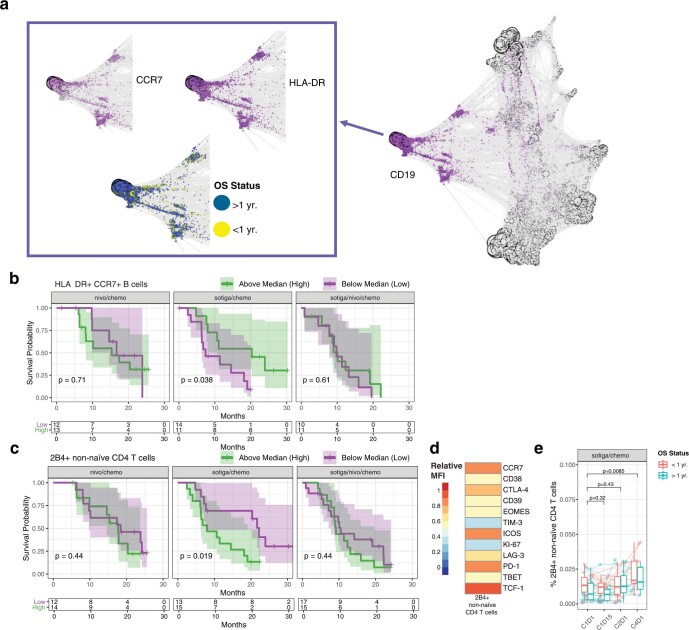
Higher frequencies of specific B cell populations and lower concentrations of 2B4+ T cells are associated with survival in patients treated with sotiga/chemo. **a**, Force-directed graph visualization of unsupervised clustering of cells from CyTOF across all patients and timepoints, illustrating a specific population of B cells (CD19+, CCR7+, HLA-DR+) associating with survival and followed up on with gating analysis in further panels. **b**, KM curves for overall survival stratified by frequencies of pretreatment circulating HLA-DR+ CCR7+ B cells out of total leukocytes, above and below the median frequency. **c**, KM curves for overall survival stratified by frequency of pretreatment circulating 2B4+ non-naïve CD4 T cells out of total non-naïve CD4 T cells. **d**, Heatmap of pretreatment relative median fluorescence intensity of markers present on 2B4+ non-naïve CD4 T cells across all patients. **e**, Frequencies of 2B4+ non-naïve CD4 T cells pretreatment (C1D1) and on-treatment (C1D15, C2D1, and C4D1), grouped by survival status at 1 year. Box plots show median and quartiles and whiskers depict 95% CI. Individual patient values are shown in thin lines and colored by survival status at 1 year. P-values represent two-sided Wilcoxon signed-rank tests between timepoints, illustrating changes on-treatment. On all KM curves, median values were determined using all data across the 3 arms, P-values are from a log-rank test between groups, and shaded regions illustrate 95% CI. Sample sizes for cell populations shown in e: n = 28, 23, 27 and 18 biologically independent samples at C1D1, C1D15, C2D1 and C4D1.

We hypothesized that patients who experienced survival benefit after sotiga/chemo would have differentiating attributes of the APC compartment in circulation compared to patients who did not experience survival benefit based on earlier pre-clinical and clinical data^10,17^. We used unsupervised clustering to identify multiple circulating DC subsets (Fig. 4a and Supplementary Table 8) associated with survival as measured before and after treatment with sotiga/chemo. After using manual gating to delineate these DC subsets, we found that patients with longer OS had higher pre-treatment frequencies of cross-presenting DCs (CD1c^+^CD141^+^ DCs; Fig. 4b) and higher on-treatment frequencies of CD141^+^ DCs, with reduced CD1c co-expression associated with longer survival (C1D15; Fig. 4c,d). Pre-treatment cross-presenting DCs had the greatest predictive value of the strongest circulating biomarkers in the sotiga/chemo arm in a combined multivariable survival model (Supplementary Fig. 4b). Higher on-treatment (C2D1) frequencies of conventional DCs (cDCs; Supplementary Fig. 6 and Supplementary Table 7) were also associated with longer survival (Fig. 4e). Furthermore, when circulating proteins associated with DC maturation were examined, higher on-treatment (C1D15) concentrations of soluble CD83 and soluble ICOSL associated with longer survival (Supplementary Fig. 7). In addition, higher pre-treatment frequencies of circulating HLA-DR^+^CCR7^+^ B cells associated with longer survival (Extended Data Fig. 5a,b). Overall, patients with longer survival after sotiga/chemo treatment, in contrast to patients who survived longer following nivo/chemo, had higher pre-treatment frequencies of circulating DCs and B cells and DC phenotypic changes on-treatment.

Pre-treatment frequencies of some CD4 T cell populations also associated with survival benefit after sotiga/chemo treatment. Higher pre-treatment frequencies of circulating antigen-experienced (PD-1^+^Tbet^+^) non-naive CD4 T cells associated with longer survival (Fig. 4f). These cells co-expressed high levels of TCF-1 (Fig. 4g). Patients with >1 year survival expressed numerically higher frequencies of this cellular phenotype on-treatment (C1D15 and C2D1) (Fig. 4h). Additionally, type-1 helper (Tbet^+^Eomes^+^) non-naive CD4 T cells associated with longer survival (Fig. 4i). These cells co-expressed high levels of PD-1 (Fig. 4j). Patients with >1 year survival expressed numerically higher frequencies of this cellular phenotype on-treatment (C2D1 and C4D1) (Fig. 4k). Lower pre-treatment frequencies of circulating non-naive CD4 T cells expressing 2B4 also associated with longer survival (Extended Data Fig. 5c). These cells co-expressed other molecules associated with exhausted or anti-inflammatory phenotypes (PD-1, CTLA-4 and LAG-3) and did not express Ki-67 (Extended Data Fig. 5d). Additionally, the frequency of this phenotype increased on-treatment (C4D1) in circulation but did not remain associated with survival status (Extended Data Fig. 5e). Overall, pre-treatment type-1 (Tbet^+^) CD4 T cells in circulation associated with survival benefit after sotiga/chemo, whereas higher levels of potentially dysfunctional 2B4^+^ CD4 T cells were associated with shorter survival.

**Extended Data Fig. 6:**
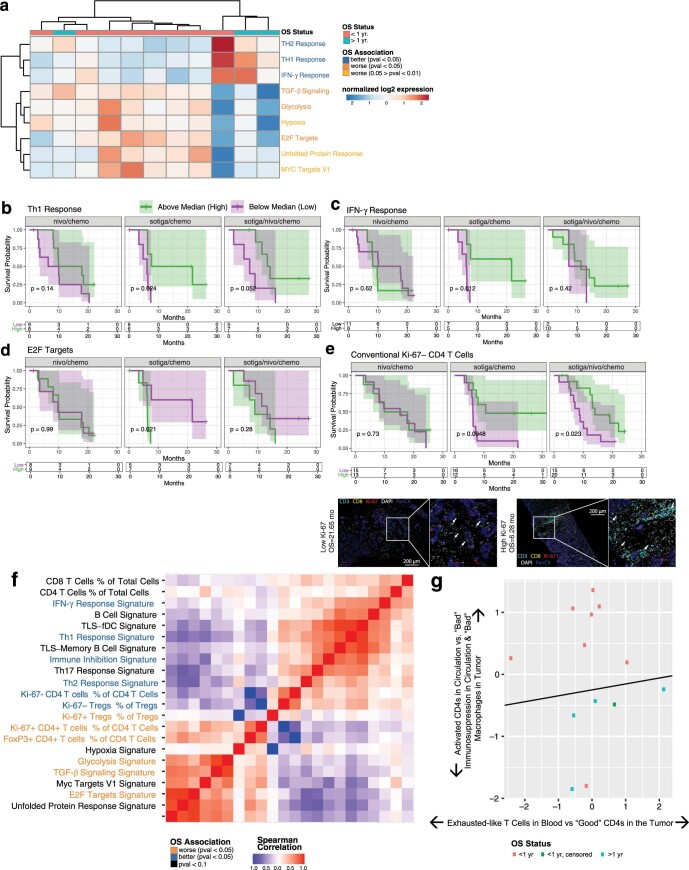
T helper signatures and proliferating CD4 T cells in the tumor associate with survival in patients receiving sotiga/chemo treatment. **a**, Heatmap showing results of unsupervised clustering of gene expression signatures and survival status in the sotiga/chemo arm. Individual patients are shown in columns and annotated by survival status at 1 year to illustrate association. Gene expression signature labels are color coded based on survival association by log-rank test, calculated independently from unsupervised clustering. **b, c, d**, KM curves for overall survival stratified by median values of Th1 (b), IFNγ (c), and E2F (d) gene expression signatures. **e**, KM curve for overall survival stratified by median values of Ki-67^−^ Foxp3- CD4 T cells from mIF on pretreatment tumor samples (e, top panel) and representative images from tumor samples high and low in Ki-67- Foxp3- CD4 T cells (e, bottom panel). Labels indicate marker grouping and patient survival values. **f**, Spearman correlation matrix of tumor immune infiltrate and gene expression signatures in pretreatment tumor biopsies, with labels colored by association with overall survival by log-rank test. **g**, Multi-omic dimensionality reduction of circulating factors and tumor data using Independent Component Analysis, with each dot representing a single patient colored by survival status at one year and with position determined by reduced dimensionality across all tumor and circulating biomarkers. Black separating line serves to illustrate a separation and is not computationally derived. On all KM curves, median values were determined using all data across the 3 arms, P-values are from a log-rank test between groups, and shaded regions illustrate 95% CI.

Patients with longer survival had a distinct CD4 helper T cell infiltrate in the tumor tissue lower immunosuppressive tumor gene expression signatures and frequencies of immune cell types associated with immune suppression. We identified nine gene expression signatures associated with survival (*P* < 0.1) and used unsupervised clustering to group patients by expression of these signatures to study associations with 1-year OS (Extended Data Fig. 6a and Supplementary Tables 8 and 9). CD4 T cell gene expression signatures associated with longer survival included Th2, Th1 and IFN-γ response signatures (Extended Data Fig. 6a). Higher expression of TGF-β, E2F signaling and glycolysis gene signatures was associated with shorter survival (Extended Data Fig. 6a). The association between survival and higher expression of Th1 and IFN-γ response signaling signatures was specific to the sotiga/chemo arm (Extended Data Fig. 6b,c). Similarly, the association observed between survival and lower expression of E2F signaling was unique to sotiga/chemo (Extended Data Fig. 6d). In addition, patients with longer survival had higher frequencies of tumor-infiltrating non-proliferating (Ki-67^−^) conventional and regulatory (Foxp3^+^) CD4 T cells (Extended Data Fig. 6e and Supplementary Table 8) and lower frequencies of infiltrating proliferating (Ki-67^+^) CD4 T cells (Supplementary Table 8). Tumor-infiltrating proliferating conventional and regulatory CD4 T cells were positively correlated with increased E2F signaling as well as hypoxic and glycolytic gene signatures. In contrast, non-proliferative conventional and regulatory CD4 T cells positively correlated with CD4 helper immune gene signatures (Extended Data Fig. 6f). Multi-omic dimensionality reduction analysis of both circulating and tumor factors at baseline showed that patients were separated by OS status in the reduced dimensional space, and this separation was driven by circulating CD4^+^ T cells and immunosuppressive markers in the circulation and tumor-infiltrating macrophages (Extended Data Fig. 6g and Supplementary Table 8).

**Fig. 5:**
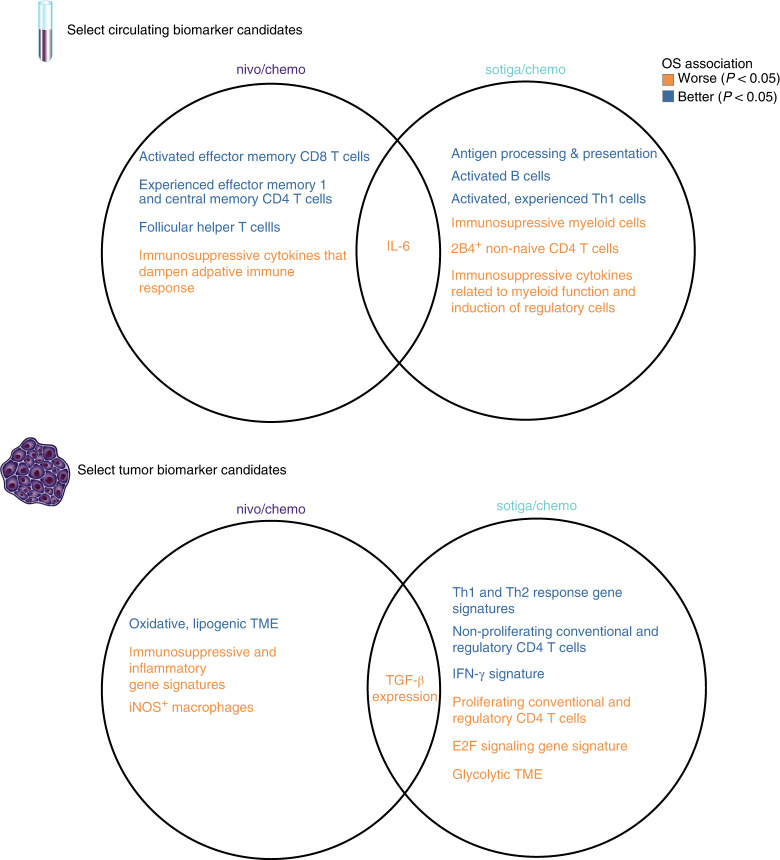
Biomarkers of survival after nivo/chemo and sotiga/chemo and their overlap. Venn diagrams of broad categories of circulating biomarkers (top). Left circle shows biomarkers of survival after nivo/chemo; right circle shows biomarkers of survival after sotiga/chemo; and center shows overlapping biomarkers that are associated with survival in both treatment groups. Color indicates direction of association, with blue for higher values associating with longer survival and red for higher values associating with shorter survival by log-rank test. The same structure is shown for tumor biomarkers (bottom).

Thus, pre-treatment biomarker profiles in both blood and tumor tissue that associated with survival benefit after sotiga/chemo and nivo/chemo treatment were distinct (Fig. 5 and Supplementary Table 8). As all patients received chemotherapy, these potential predictive markers may not merely relate with prognosis or chemotherapy treatment. This conclusion is strengthened by the strong mechanistic relationship of each set of biomarkers to the PD-1 and CD40 axis.

### Correlates of survival benefit after sotiga/nivo/chemo

**Extended Data Fig. 7:**
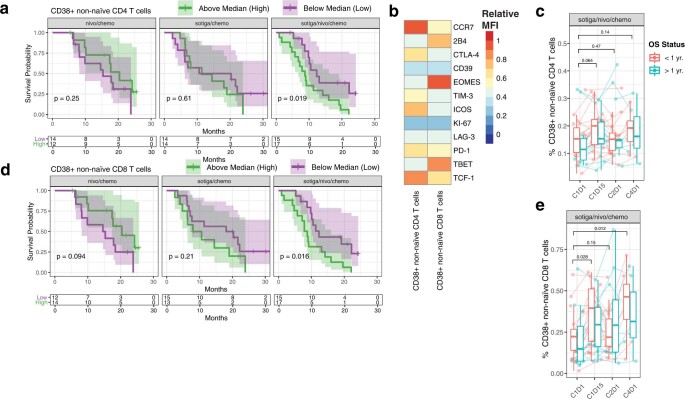
Lower frequencies of circulating CD38+ non-naïve T cells are associated with longer survival in patients treated with sotiga/nivo/chemo. **a,d**, KM curves for overall survival stratified by frequencies of circulating CD38+ non-naïve CD4 (a) and CD8 (d) T cells at baseline, above and below the median frequency value. **b**, Heatmaps of relative median fluorescent intensity of pretreatment markers present on CD38+ non-naïve CD4 and CD8 T cells across all patients. **c, e**, Frequencies of CD38+ non-naïve CD4 (c) and CD8 (e) T cells pretreatment (C1D1) and on-treatment (C1D15, C2D1, and C4D1), grouped by survival status at 1 year. For all cell populations shown, frequencies are out of parent. Box plots show median and quartiles and whiskers depict 95% CI. Individual patient values are shown in thin lines and colored by survival status at 1 year. P-values for timeseries represent two-sided Wilcoxon signed-rank tests between timepoints, illustrating changes on-treatment. On KM curves, median values were determined using all data across the 3 arms, P-values are from a log-rank test between groups, and shaded regions illustrate 95% CI. Sample sizes for cell populations shown (c, e): n = 32, 27, 29 and 14 biologically independent samples at C1D1, C1D15, C2D1 and C4D1.

**Extended Data Fig. 8:**
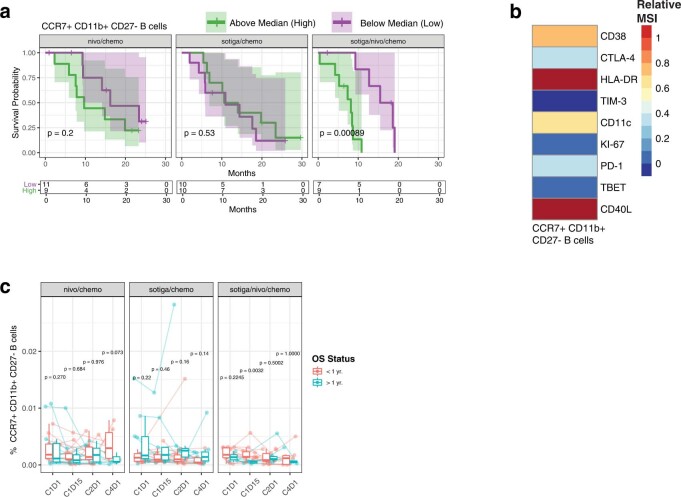
Survival in response to combinational therapy of sotiga/nivo/chemo may be affected by regulatory B cells in circulation. **a**, KM curves for overall survival stratified by CCR7+ CD11b+ CD27- B cells above and below the median frequency value. **b**, Heatmap of relative median signal intensity of different proteins present on CCR7+ CD11b+ CD27- B cells on-treatment (C1D15) across all patients. **c**, Frequencies of CCR7+ CD11b+ CD27- B cells pretreatment (C1D1) and on-treatment (C1D15, C2D1, C4D1), grouped by survival status at 1 year, for each treatment arm. For all cell populations shown, frequencies are out of total leukocytes. On KM curves, median values were determined using all data across the 3 arms, P-values are from a log-rank test between groups, and shaded regions illustrate 95% CI. Box plots show median and quartiles and whiskers depict 95% CI. Individual patient values are shown in thin lines and colored by survival status at 1 year. P-values for timeseries represent two-sided Wilcoxon signed-rank tests between survival groups at each timepoint. Sample sizes for cell populations shown (c): n = 25, 20, 23, 13; n = 29, 23, 24, 22; n = 26, 20, 26, 13 biologically independent samples at C1D1, C1D15, C2D1 and C4D1 in nivo/chemo, sotiga/chemo, sotiga/nivo/chemo treatment arms, respectively.

We found that, for sotiga/nivo/chemo, many biomarkers that associated with longer survival after sotiga/chemo and nivo/chemo individually were not predictive (Supplementary Table 8). However, we identified several unique cell populations that were associated with longer survival after sotiga/nivo/chemo treatment, including lower frequencies of activated (CD38^+^) non-naive CD4 T cells (Extended Data Fig. 7a). The CD38^+^ non-naive CD4 T cell population co-expressed CCR7, TCF-1, CTLA-4, PD-1 and ICOS (Extended Data Fig. 7b, left column). The frequency of this cellular phenotype numerically increased on-treatment but was not related to survival status (Extended Data Fig. 7c). Similarly, lower frequencies of activated (CD38^+^) non-naive CD8 T cells were associated with longer survival (Extended Data Fig. 7d). These cells co-expressed CCR7, PD-1, Tbet, Eomes, TCF-1 and 2B4 (Extended Data Fig. 7b, right column). The frequency of these cells numerically increased on-treatment but was not related to survival status (Extended Data Fig. 7e). Using unsupervised clustering analysis for discovery, followed by manual gating, we identified lower frequencies of CCR7^+^CD11b^+^CD27^−^ B cells in circulation on-treatment (C1D15) associated with longer survival (Extended Data Fig. 8a and Supplementary Table 8). No association between survival and CCR7^+^CD11b^+^CD27^−^ B cells was observed in the nivo/chemo or sotiga/chemo arms. In the nivo/sotiga/chemo arm, these cells co-expressed CD40L, HLA-DR, CD11c and CD38 (Extended Data Fig. 8b). On-treatment (C2D1 and C4D1), these cells did not associate with survival status (Extended Data Fig. 8c). Collectively, these data suggest that higher frequencies of chronically activated T cells before treatment and on-treatment and the presence of CCR7^+^CD11b^+^CD27^−^ B cells on-treatment relate to shorter survival after sotiga/nivo/chemo.

## Discussion

The non-randomized phase 1b portion of the PRINCE trial demonstrated that sotiga/chemo +/- nivo is tolerable, clinically active and a potential chemoimmunotherapy combination for this disease^12^. In the randomized phase 2 portion of this study, modest OS increases were observed for the nivo/chemo and sotiga/chemo arms versus historical control, and only the nivo/chemo arm met the primary endpoint. Although the ORR of nivo/chemo was 50%, many of the responses had short duration and were not confirmed by a subsequent scan. A previous study of nivo/chemo failed to demonstrate clinical benefit in first-line therapy for patients with mPDAC^18^. Acknowledging the limitations of cross-study comparisons, possible explanations for the contradictory results include our study having a larger proportion of patients with baseline PD-L1 >1% (56% versus 30%), our prohibition of steroids as premedication with chemotherapy and ~1.5× higher chemotherapy exposure in our study. Post hoc subgroup analyses of OS did not reveal any imbalances in clinical characteristics to which the survival increases could be solely attributed. No clear benefit was observed for ORR or PFS in any arm. This study was not powered to compare between arms; therefore, we cannot conclude that sotiga/chemo is inferior to nivo/chemo. The data suggest that these treatment regimens are not appropriate for an all-comers mPDAC population but that a biomarker selection strategy may be warranted for future studies of both nivo/chemo and sotiga/chemo.

Our exploratory data on pharmacodynamic effects aligned with the expected mechanism of action of either PD-1 blockade or CD40 activation^19,20^. Additionally, unique immune pharmacodynamic effects for nivo/chemo and sotiga/chemo were individually identified. These data indicate that the immune therapies evaluated here have distinct activity over and above the chemotherapeutic effect.

In addition to pharmacodynamic effects, we examined biomarkers associated with survival. This exploratory analysis demonstrated that patients with longer survival after nivo/chemo and sotiga/chemo can be identified by various predictive biomarkers from the circulation and tumor and that these predictive biomarkers are distinct for the two treatment arms. In the nivo/chemo arm, most circulating predictive biomarkers were T cell subsets. In particular, many subsets of antigen-experienced, type-1 (Tbet^+^) CD4 T cells before treatment were strongly predictive. In the tumor, gene expression signatures of immune suppression and metabolic state were predictive of shorter survival. In the sotiga/chemo arm, CD4 T cell, B cell and DC subsets were strongly associated with longer survival. The particular B cell subsets associated with survival align with the expected mechanism of the CD40 agonist and may relate to the presence of germinal centers^21^. Furthermore, the pre-treatment and on-treatment DC subsets observed to associate with survival suggest the benefit of stronger cross-presentation on-treatment, aligning with previous studies that have suggested that agonistic CD40 treatment induces cross-presenting DCs and may promote epitope spreading^22–24^. In the tumor, several pre-treatment gene expression signatures and immune cell population abundances associated with shorter survival, including many observed in preclinical KPC mouse models^25^.

A prospective study is needed to demonstrate that these biomarkers are truly predictive of survival with these regimens. Based on our data, T_fh_ cells could make a good target for patient selection for nivo/chemo, as these cells are found in relatively high abundance and have the highest predictive value against all circulating biomarkers in the nivo/chemo arm. Baseline assessment of circulating CD4 T cells may provide the most tractable biomarker for patient selection for sotiga/chemo in subsequent studies. Several predictive biomarkers found in our study could inform mechanisms and future therapies for patients with mPDAC. Notably, unlike data reported from other solid cancers^26–29^, circulating antigen-experienced CD8 T cells or infiltrating CD8 T cells were not associated with OS after either immunotherapy regimen. In contrast, the associations with survival were mainly observed with higher frequencies of circulating CD4 T cells before treatment. Furthermore, infiltrating T cells in all tumor samples were largely CD4 T cells, and, surprisingly, very few patients’ tumor samples had CD8 T cell infiltration. We hypothesize that the CD4 T cell compartment may have a critical role in response to chemoimmunotherapy treatment in mPDAC.

The sotiga/nivo/chemo arm did not demonstrate a meaningful improvement in the 1-year OS rate, and relatively few tumor and circulating immune biomarkers were associated with survival. In particular, biomarkers associated with longer survival in the sotiga/chemo and nivo/chemo monotherapy immunotherapy treatment arms were not relevant. In addition, many of the pharmacodynamic effects observed in the other two arms were somewhat attenuated in the sotiga/nivo/chemo arm, potentially indicative of a decreased or antagonistic effect when the dual immunotherapies and chemotherapy are used in combination. We hypothesize that this treatment resulted in systemic hyperactivation of the immune system, leading to a less functional immune state and, thus, decreased anti-tumor immunity. Indeed, a specific population of CD38^+^ CD4 and CD8 T cells associated with shorter survival in response to sotiga/nivo/chemo treatment. The immunologic phenotype of these cells suggests that excessive T cell activation on-treatment could be leading to a terminally exhausted state^30^. Additionally, sotiga/nivo/chemo treatment led to increases in circulating CCR7^+^CD11b^+^CD27^−^ B cells that tracked with shorter survival at two time points after treatment. The expression of CD11b on B cells has been associated with a tolerogenic or regulatory response in the lupus setting^31^ and is postulated to have a dampening effect on anti-tumor immunity. Preclinical work in glioma has suggested that agonistic CD40 impairs response to PD-1 blockade in part through the induction of regulatory B cells^32^. Thus, we hypothesize that regulatory B cells could contribute to suppressed immunity after the dual immunotherapy combination in mPDAC. However, mechanistic studies need to be conducted to further understand these findings and how B cells potentially affect anti-tumor immune responses and durable clinical benefit in the mPDAC setting.

One inherent limitation of this study design is the intentional omission of a chemotherapy control arm, which accelerated enrollment of patients unwilling to be randomized to control chemotherapy arms but, analytically, hampers our ability to assess the survival benefit against contemporaneous control patients. Although we benchmarked OS against the initial, definitive study of gemcitabine/nab-paclitaxel, a subsequent phase 3 study reported a higher 1-year OS rate of approximately 40–45%^33^. Second, this study enrolled patients across a small number of tertiary care cancer centers. To assess whether this introduced bias, we generated a synthetic control arm of patients receiving chemotherapy from PRINCE’s top enrolling sites who met this study’s inclusion and exclusion criteria and observed a 45% 1-year OS rate^34^. These updated rates are still numerically smaller than the 1-year OS observed for nivo/chemo and sotiga/chemo, suggesting that the addition of nivo or sotiga may provide additional clinical benefit. Regarding the translational analyses, a chemotherapy control arm will eventually be needed to ensure that the identified biomarkers are truly predictive of an immunotherapy response. However, very few biomarkers are overlapping between the arms, suggesting that the identified biomarkers are related to a specific immunotherapy response rather than a chemotherapy response.

The phase 1b/2 PRINCE trial leveraged a unique study design, relatively rapid enrollment^35,36^, centralized sample processing and multi-omic profiling to generate a sizable clinical–translational dataset to identify potential mechanisms of response and resistance to chemoimmunotherapy regimens in mPDAC. Our findings do not support additional trials testing these chemoimmunotherapy combinations in an all-comer mPDAC population because only a subset of patients is likely to realize the full benefits of these regimens. Rather, as a first step toward characterizing which patients derive clinical benefit, we have identified here potential biomarkers that can now be tested prospectively to determine if this allows for minimally invasive biomarker-enrichment designs for chemoimmunotherapy treatment in mPDAC.

## Methods

### Study design and safety monitoring

In this phase 1b/2 study, patients 18 years of age or older with mPDAC were enrolled from seven academic hospitals in the United States that are part of the Parker Institute for Cancer Immunotherapy pancreas cancer consortium. Inclusion and exclusion criteria were identical for the phase 1b and phase 2 portions of the study. Prior treatment for metastatic disease was not allowed, although prior adjuvant and neoadjuvant chemo/radiotherapy was allowed if completed >4 months before enrollment. Patients were required to have archival or fresh tumor specimens available before treatment or be able to undergo a biopsy to acquire tissue. Additional key eligibility criteria included ECOG performance status score of 0–1, adequate organ function and the presence of at least one measurable lesion per Response Evaluation Criteria in Solid Tumors (RECIST) version 1.1. Patients were excluded if they had previous exposure to agonistic CD40, anti-PD-1, anti-PD-L1 monoclonal antibodies or any other immunomodulatory anti-cancer agent. Patients were also excluded if they had ongoing or recent autoimmune disease requiring systemic immunosuppressive therapy, had undergone solid organ transplantation or had a concurrent cancer, unless indolent or not considered to be life-threatening (for example, basal cell carcinoma).

The phase 1b trial was a non-randomized, open-label, multi-center, four-cohort, dose-ranging study that aimed to identify the recommended phase 2 dose (RP2D) of anti-CD40 sotigalimab (sotiga) in combination with chemotherapy (gemcitabine (gem) and nab-paclitaxel (NP)), with or without anti-PD1 nivolumab (nivo)^12^. The phase 2 trial was a randomized, open-label, multi-center, three-arm study of chemotherapy combined with nivo, sotiga or both immune modulating agents.

An RP2D of 0.3 mg kg^−1^ of sotiga was defined during the phase 1b portion of the study by a data review team (DRT) comprised of investigators and sponsor clinical staff. During phase 2, the DRT met to review all safety data for each study arm on a quarterly basis. A Bayesian termination rule was employed to monitor toxicity and determine whether enrollment or dosing in a study arm(s) needed to be halted. A minimally informative beta (0.5, 2.5) prior was assumed. For each treatment arm, if the number of patients with an unacceptable toxicity (as defined in Section 6.1 of the Study Protocol) was greater than or equal to the number provided in Table 19 of the study protocol, then termination of that particular treatment arm would be considered, as it is likely that the true toxicity rate is over 30%, as noted by Bayesian posterior probabilities. This rule was intentionally conservative early in the enrollment phase.

The protocol and all amendments were approved by the lead institutional review board at the University of Pennsylvania and were accepted at all participating sites. The study was conducted in accordance with the principles of the Declaration of Helsinki and International Conference on Harmonisation Good Clinical Practice guidelines. All patients provided written informed consent before enrollment. The Study Protocol and Statistical Analysis Plan are available as part of the Supplementary Information.

### Randomization and blinding

The phase 2 trial was open label with no blinding. Patients were randomly assigned to one of three arms: nivo/chemo, sotiga/chemo or sotiga/nivo/chemo. Twelve DLT-evaluable patients (six each on sotiga/chemo and sotiga/nivo/chemo) from the non-randomized phase 1b study were included in analyses of phase 2 efficacy (see the ‘Statistical analysis’ section for details on analysis population definitions). To achieve balance in the total number of patients enrolled in each arm, the first 12 patients enrolled in phase 2 were randomly allocated in a 4:1:1 ratio to nivo/chemo, sotiga/chemo or sotiga/nivo/chemo, respectively (because nivo/chemo did not accrue patients in phase 1b, more patients needed to be enrolled in that arm). The remaining patients were randomly allocated in a 1:1:1 ratio. Randomization was managed by the Parker Institute for Cancer Immunotherapy using an interactive voice/web response system (IxRS). A permuted block design, without stratification by baseline patient or tumor characteristics, was used for randomization. Patients who were randomized but did not receive any study drug were replaced via randomization of additional patients.

### Procedures

For each 28-day cycle, gem/NP at 1,000 mg m^−^^2^ and 125 mg m^−^^2^, respectively, were administered intravenously on days 1, 8 and 15 for each arm. Nivo was administered at 240 mg intravenously on days 1 and 15. Sotiga was administered at 0.3 mg kg^−1^ intravenously on day 3, 2 days after chemotherapy. Alternatively, sotiga could be administered on day 10 if not administered on day 3, provided patients received chemotherapy on day 8. Investigators were also given the option to use 21-day chemotherapy cycles, in which case the day 15 dose was not administered. Up to two dose reductions were permitted for sotiga and gem, and up to three dose reductions were permitted for NP for management of toxicity. Nivo was allowed to be withheld, but dose reductions were not permitted. A maximum interruption of 4 weeks was permitted per protocol before study discontinuation was required.

Patients were assessed radiographically every 8 weeks for the first year and every 3 months thereafter, regardless of dose delays. Disease assessments were collected until radiographic progression or initiation of subsequent therapy, whichever occurred first. Patients were subsequently followed for survival. Safety assessments included vital signs, physical examinations, electrocardiograms and laboratory tests. Adverse events were graded according to the National Cancer Institute Common Terminology Criteria for Adverse Events, version 4.03. Adverse event terms were coded using the Medical Dictionary for Regulatory Activities version 23.0.

Blood samples for isolation of PBMCs were collected longitudinally at participating clinical sites, shipped overnight and processed at a central location (Infinity Biologix) over a Ficoll gradient and cryopreserved. Serum was processed within 2 hours of collection at each site and frozen immediately at −80 °C and then batch shipped to a central biorepository. Blood sampling for immune biomarkers occurred during screening, at cycle 1 days 1 and 15, at cycles 2–4 day 1 and at treatment discontinuation. If a patient began any new anti-cancer therapy before their end-of-treatment visit, samples were not collected. For patients who remained on treatment for at least 1 year, blood was collected at 1 year and every 6 months thereafter.

Baseline or archival as well as post-treatment tumor specimens were collected for biomarker analyses. Fresh tumor biopsies were immediately snap-frozen or formalin-fixed and paraffin-embedded (FFPE). Any medically feasible post-treatment tumor samples were accepted; however, the preference was for a sample during cycle 2, after the second dose of sotiga or third dose of nivo depending on the assigned treatment arm. Additional biopsies were allowed for patients who had prolonged stable disease, defined as more than two consecutive disease assessments demonstrating response via RECIST version 1.1 as well as at the time of disease progression. Ad hoc biopsy collection was permitted with the approval of the medical monitor.

### Outcomes

The primary endpoint was the 1-year OS rate of each treatment arm, compared to the historical rate of 35% for gem/NP^13^. Secondary endpoints were PFS, DOR, ORR, DCR and the incidence of adverse events. Key exploratory endpoints included the evaluation of immune pharmacodynamic effects and tumor and immune biomarker analyses.

### Statistical analysis of clinical data

Efficacy analyses were conducted on the efficacy population, defined as (1) all patients who were randomized in phase 2 and received at least one dose of any study drug and (2) the 12 DLT-evaluable patients (six on sotiga/chemo and six on sotiga/nivo/chemo; defined as experiencing a DLT or receiving at least two doses of chemotherapy and one dose of sotiga during cycle 1) who were enrolled at the RP2D in phase 1b^12^. For efficacy analyses, patients were grouped according to the treatment arm assigned at randomization. Safety analysis was conducted on all phase 1b (DLT-evaluable and non-DLT-evaluable) and phase 2 patients who received at least one dose of any study drug at the RP2D (defined as the safety population). For safety analyses, patients were grouped according to the study treatment actually received (that is, ‘as treated’). Specifically, two phase 2 patients were randomly allocated to sotiga/nivo/chemo but only received doses of chemotherapy and nivo (that is, sotiga was not received); these patients were grouped as sotiga/nivo/chemo for efficacy analyses (that is, the arm assigned at randomization) and as nivo/chemo for safety and biomarker analyses (that is, using an ‘as treated’ approach).

This study did not include a control arm of gem/NP (chemotherapy). Therefore, the 1-year OS rate for each arm was estimated and compared to a historical value of 35%^13^. This study was not powered for statistical comparison between arms, and no adjustment for multiple comparisons was performed for the clinical endpoints.

OS was defined as the time from treatment initiation until death from any cause. Patients who were not reported as having died at the time of analysis were censored at the most recent contact date. OS and the 1-year OS rate were estimated by the Kaplan–Meier method for each treatment arm. The 1-year OS rate and corresponding one-sided, 95% CI were calculated to determine whether the lower bound of the CI excluded the assumed historical value of 35%. *P* values were calculated using a one-sided, one-sample *z-*test of the Kaplan–Meier estimate of the 1-year OS rate (and its standard error) against the historical rate of 35%. The null hypothesis was a 1-year OS rate of 35%, and the alternative hypothesis was a 1-year OS rate of 55%. Planned enrollment was 105 patients (35 per arm), which included 12 DLT-evaluable patients from the non-randomized phase 1b. A sample size of 35 patients per arm provided 81% power to test this hypothesis, using a one-sample *z-*test with a one-sided 5% type I error rate.

ORR was defined as the proportion of patients with an investigator-assessed partial response (PR) or complete response (CR) per RECIST version 1.1—confirmation of response was not required. DCR was the proportion of patients with a PR, CR or stable disease lasting at least 7 weeks as best response; DOR was the time from the first tumor assessment demonstrating response until the date of radiographic disease progression; and PFS was the time from treatment initiation until radiographic disease progression or death (whichever occurred first). CIs for ORRs were calculated using the Clopper–Pearson method. The Kaplan–Meier method was used to estimate DOR and PFS and the corresponding CIs. Safety and tolerability were summarized descriptively in terms of adverse events. Statistical analyses were performed using R version 4.1.0 or higher.

### Interim analysis

Two pre-specified interim analyses (IAs) of phase 2 clinical data were performed. These IAs were strictly meant to support decision marking for future studies. No adaptations to the study design or conduct, including termination due to lack of efficacy, were planned based on the interim results, and no control of type I error was applied for any of the endpoints at the interim or final analysis. The IAs were performed by the Parker Institute for Cancer Immunotherapy, and results were shared with the study investigators and pharmaceutical partners (Apexigen and Bristol Myers Squibb).

The first IA occurred approximately 4 months after the last patient was randomized in phase 2, and the second IA occurred approximately 9 months after the last patient was randomized. Both IAs assessed safety and all efficacy endpoints (ORR, DCR, DOR, OS and PFS) for patients enrolled in phase 1b. In addition, the first IA included phase 2 analysis of ORR and DCR, and the second IA included phase 2 analysis of all efficacy endpoints excluding OS (that is, ORR, DCR, DOR and PFS). Phase 2 OS data were not analyzed during any IA.

### Immunophenotyping by CyTOF

A broad immunophenotyping panel was used on cryopreserved PBMCs by CyTOF analysis run under uniform protocols (PMID: 31315057) at Primity Bio in a blinded fashion. Cryopreserved PBMCs were thawed in 37 °C pre-warmed RPMI-1640 containing 10% FBS and 25 U ml^−1^ of benzonase. Samples were washed once more in RPMI-1640 containing 10% FBS and 25 U ml^−1^ of benzonase and a third time in 37 °C pre-warmed RPMI-1640 containing 10% FBS. Samples were resuspended in 1,000 nM of cisplatin for viability discrimination, prepared in PBS containing 0.1% BSA, for 5 minutes at room temperature, and then washed with staining buffer. Human BD Fc block was added to the cells for 10 minutes at 4 °C, followed by the surface antibody cocktail. The surface staining cocktail was incubated for 30 minutes at 4 °C. Samples were washed out of the stain twice with staining buffer. The cells were then resuspended in FoxP3 Transcription Factor 1× Fix/Perm buffer (eBioscience) for 1 hour at room temperature to prepare the cells for intracellular staining. The fixation was then followed by a wash in 1× permeabilization buffer. The intracellular staining cocktail was prepared in the permeabilization buffer and added to the samples and incubated at room temperature for 1 hour. After the intracellular stain, the samples were washed twice with the permeabilization buffer and once with staining buffer. Before acquisition on the CyTOF, samples were resuspended in an iridium-intercalating solution for at least 24 hours and stored at 4 °C. On the day of acquisition, the samples were washed five times in cell culture grade water (HyClone) and run on the CyTOF Helios instrument (Fluidigm). Details on the CyTOF panel are displayed in Supplementary Table 10. Data were analyzed using CellEngine version 1 cloud-based flow cytometry analysis software (CellCarta).

Supervised gating was performed manually by a scientist without reference to clinical outcome. High-level gates were tailored per sample. Single marker gates were drawn uniformly for analysis across patients and time points, with example gating strategy provided in Supplementary Fig. 6.

After gating for live singlets, immune populations were defined as follows, as shown in Supplementary Fig. 6. CD4 and CD8 T naive, effector and memory populations were identified based on CD45RA, CD27 and CCR7 expression. Tregs were identified based on Foxp3, CD25 and CD127 expression. B cells were identified based on CD19 expression and further distinguished into memory versus naive versus plasmablast based on expression of CD38 versus CD27. NK cells were identified based on CD56 expression and further subdivided based on CD56 versus CD16 expression. Monocytes were identified based on expression of CD14 and HLA-DR and further subdivided in classical, non-classical and intermediate based on the expression of CD14 versus CD16. DCs were defined as HLA-DR^+^CD14^−^CD16^−^ non-lymphocytes and further distinguished between myeloid and plasmacytoid based on expression of CD11c versus CD123, respectively. Myeloid DCs were further subdivided on the basis of CD141 expression into cDCs type 1 (cDC1; CD141^+^) and conventional DCs type 2 (cDC2; CD141^−^).

In addition to manual gating of defined populations, data were analyzed in an unsupervised fashion. To do this, all samples for all patients and all time points were combined together and run through a clustering algorithm^37,38^. After clustering, clusters were visualized using a force-directed graph layout^37,38^ and colored by association with OS. Using this visualization, clusters of interest were identified, and then the relevant populations were added to the manual gating hierarchy. All time series and survival analyses shown in the results are derived from gated populations, whether discovered by manual gating or unsupervised analysis.

Optimized concentrations/dilutions for antibodies used in CyTOF experiments were: CD45, CD3, CD19, CD117, CD11b, CD4, CD8a, CD11c, CD14, FcER1, CD123, gdTCR, CD45RA, CD366, CD274, CD27, Tbet, CD152, FoxP3, CD33, CD45RO, CD127, CD197, Ki-67, CD25, TCRVa24-Ja18, CD38, HLA-DR, CD56 and CD16 (all used at 1:100 per the manufacturer’s recommendation); CD66d, 3 µg ml^−1^; CD7, 3 µg ml^−1^; CD86, 6 µg ml^−1^; CD1c, 3 µg ml^−1^; CD64, 6 µg ml^−1^; CD206, 3 µg ml^−1^; CD141, 3 µg ml^−1^; CD154, 3 µg ml^−1^; CD40, 1.5 µg ml^−1^; CD192, 6 µg ml^−1^; nivolumab, 1 µg ml^−1^; and anti-human IgG4, 1 µg ml^−1^.

Sample sizes for all cell populations identified through CyTOF analysis (Fig. 4a-e and Extended Data Figs. 3b, 5a,b and 8a–c) are listed as follows: nivo/chemo: C1D1 (*n* = 25), C1D15 (*n* = 20), C2D1 (*n* = 23) and C4D1 (*n* = 13); sotiga/chemo: C1D1 (*n* = 29), C1D15 (*n* = 23), C2D1 (*n* = 24) and C4D1 (*n* = 22); and sotiga/nivo/chemo: C1D1 (*n* = 26), C1D15 (*n* = 20), C2D1 (*n* = 26) and C4D1 (*n* = 13).

### High-parameter flow cytometry of T lymphocytes

Cryopreserved PBMC samples for fluorescent flow cytometry were analyzed in the Translational Cytometry Laboratory of the Penn Cytomics and Cell Sorting Shared Resource (University of Pennsylvania) on an extensively pre-qualified 28-color BD Symphony A5 cytometer (BD Biosciences). Staff were blinded to treatment arm and clinical outcome. At the time of analysis, cryopreserved PBMC samples were thawed in 37 °C pre-warmed RPMI-1640 medium (Gibco) containing 10% FBS and 100 U ml^−1^ of penicillin–streptomycin (Gibco). Samples were washed, counted and resuspended in medium containing 1 mg ml^−1^ of DNase I (Roche) and 5 mM magnesium chloride and incubated at 37 °C for 1 hour. After resting, cells were washed with PBS without additives (Corning) and transferred to staining tubes. PBMCs were incubated with 1 ul (0.2 µg) of 0.2 mg ml^−1^ of nivolumab antibody (Selleck Chemicals) for 5 minutes at room temperature, followed by the addition of a Fixable Viability Stain 510 for 10 minutes at room temperature in the dark. Cells were then washed twice with FACS wash buffer (PBS, 1% BSA, 2 mM EDTA). A surface antibody cocktail (T cell phenotyping antibody panel; Supplementary Table 11) was prepared daily and used to stain up to 1 × 10^7^ cells per tube. Cells were incubated for 20 minutes at room temperature, followed by washing twice with FACS staining buffer. The cells were resuspended in FoxP3 Transcription Factor Staining Buffer Fix/Perm solution (eBiosciences) and incubated for 1 hour at room temperature to prepare the cells for intracellular staining. After fixation, the samples were washed with Foxp3 permeabilization buffer. A freshly prepared cytoplasmic/intracellular staining cocktail master mix was added to the samples and incubated overnight at 4 °C. The next day, the samples were washed with permeabilization buffer and resuspended in FACS wash buffer. Cells were stored at 4 °C in the dark and acquired within 2 hours. After daily quality control, the instrument was standardized by setting hard dyed beads (BD Biosciences, Cytometer Setup and Tracking Beads (CS&T)) to predetermined target channels. Compensation controls (Invitrogen UltraComp eBeads or cells for Live/Dead stain) were prepared daily along with a frozen PBMC process control. The compensation matrix was calculated in Diva software (BD Biosciences) and used only for that day’s run. Data were analyzed using CellEngine cloud-based flow cytometry analysis software. High-level gates were tailored per patient across all time points by at least two investigators blinded to patient outcome. Single marker gates were drawn uniformly for analysis across patients and time points, with representative gating strategy provided in Supplementary Fig. 2.

After gating for live cells and the CD3^+^ population, T cell populations were defined as following, as shown in Supplementary Fig. 2. A combination of CD45RA, CD27 and CCR7 expression on CD4^+^ and CD8^+^ T cells was used to define naive (CD45RA^+^CD27^+^CCR7^+^), T central memory (CM; CD45RA^−^CD27^+^CCR7^+^), T effector memory 1 (EM1; CD45RA^−^CD27^+^CCR7^−^), T effector memory 2 (EM2; CD45RA^−^CD27^−^CCR7^+^), T effector memory 3 (EM3; CD45RA^−^CD27^−^CCR7^−^) and terminally differentiated effector memory (TEMRA) (CD45RA^+^CD27^−^CCR7^–^) subpopulations. CD4^+^ regulatory T cells were defined as Foxp3^+^CD25^hi^CD127^−/low^. The non-naive CD4^+^ and CD8^+^ T cell populations used in time series and survival analyses included the defined EM, CM and TEMRA populations defined above. Expression of additional differentiation, activation and inhibitory markers were evaluated within each of these compartments.

In addition to manual gating of defined populations, data were analyzed in an unsupervised fashion. To do this, all samples for all patients and all time points were combined together and run through a clustering algorithm^37,38^. After clustering, clusters were visualized using a force-directed graph layout^37,38^ and colored by association with OS. Using this visualization, clusters of interest were identified, and then the relevant populations were added to the manual gating hierarchy. All time series and survival analyses shown in the results are derived from gated populations, whether discovered by manual gating or unsupervised analysis.

Optimized concentrations/dilutions for antibodies used in the high-parameter flow cytometry experiments were: CD45RA, 1:200; CD8a, 1:160; CD185, 1:400; CD25, 1:200; CD226, 1:65; CD27, 1:500; CD4, 1:800; CD197, 1:40; CD223, 1:100; CD14, 1:40; CD19, 1:160; CD41a, 1:260; CD3, 1:65; CD137, 1:100; CD244, 1:20; CD366, 1:200; CD39, 1:100; CD28, 1:100; CD278, 1:100; CD127, 1:160; CD38, 1:160; TIGIT, 1:40; Eomes, 1:100; CD152, 1:400; FoxP3, 1:400; T-bet, 1:600; TCF1, 1:125; Ki-67, 1:600; KLRG1, 1:100; nivolumab, 1 mg ml^−1^; and anti-human IgG4, 1:200.

Sample sizes for all cell populations identified through X50 analysis (Figs. 3a-i and 4f–k, Supplementary Figs. 1c and 3a-c and Extended Data Figs. 3a, 5c–e and 7a–e) are listed as follows: nivo/chemo: C1D1 (*n* = 26), C1D15 (*n* = 21), C2D1 (*n* = 25) and C4D1 (*n* = 19); sotiga/chemo: C1D1 (*n* = 28), C1D15 (*n* = 23), C2D1 (*n* = 27) and C4D1 (*n* = 18); sotiga/nivo/chemo: C1D1 (*n* = 32), C1D15 (*n* = 27), C2D1 (*n* = 29) and C4D1 (*n* = 14).

### Serum proteomics profiling

Serum proteins were quantified using Olink multiplex proximity extension assay (PEA) panels (Olink Proteomics, www.olink.com) according to the manufacturer’s instructions^39^. The assay was performed at the Olink Analysis Service Center. The basis of PEA is a dual-recognition immunoassay, where two matched antibodies labelled with unique DNA oligonucleotides simultaneously bind to a target protein in solution. This brings the two antibodies into proximity, allowing their DNA oligonucleotides to hybridize, serving as template for a DNA polymerase-dependent extension step. This creates a double-stranded DNA ‘barcode’ that is unique for the specific antigen and quantitatively proportional to the initial concentration of target protein. The hybridization and extension are immediately followed by PCR amplification, and the amplicon is then finally quantified by microfluidic qPCR using the Fluidigm BioMark HD system (Fluidigm). Data were normalized using internal controls in every single sample, inter-plate control and negative controls and correction factor and expressed as log_2_ scale, which is proportional to the protein concentration. The final assay readout is reported as normalized protein expression (NPX) values, which is an arbitrary unit on a log_2_ scale where a higher value corresponds to a higher protein expression. One NPX difference equals to the doubling of the protein concentration. In this study, two Olink panels (Target96 Immuno-Oncology and Target96 Immune Response) were used, which consist of 172 unique analytes. Additional details about the analytes, detection range, data normalization and standardization are available at https://www.olink.com/resources-support/document-download-center/.

Sample sizes for all soluble proteins identified through targeted Olink platforms (Extended Data Fig. 3e,h and Supplementary Fig. 7a,b) are listed as follows: nivo/chemo: C1D1 (*n* = 32), C1D15 (*n* = 25), C2D1 (*n* = 27), C3D1 (*n* = 25) and C4D1 (*n* = 23); sotiga/chemo: C1D1 (*n* = 36), C1D15 (*n* = 29), C2D1 (*n* = 31), C3D1 (*n* = 25) and C4D1 (*n* = 27); sotiga/nivo/chemo: C1D1 (*n* = 35), C1D15 (*n* = 27), C2D1 (*n* = 32), C3D1 (*n* = 26) and C4D1 (*n* = 25).

### Whole-exome and transcriptome sequencing

FFPE tumor and normal PBMC samples were profiled using ImmunoID NeXT (Personalis)—an augmented exome/transcriptome platform and analysis pipeline that produces comprehensive tumor mutation information, gene expression quantification, neoantigen characterization, HLA typing and allele-specific HLA loss-of-heterozygosity data (HLA LOH), TCR repertoire profiling and TME profiling. Whole-exome library preparation and sequencing were performed by Personalis as a service using augmented exome sequencing^40^. DNA extracted from tumor and PBMCs was used to generate whole-exome capture libraries using the KAPA HyperPrep Kit and Agilent’s SureSelect Target Enrichment Kit, according to manufacturer recommendations, with the following amendments. (1) Target probes were used to enhance coverage of biomedically and clinically relevant genes. (2) Protocols were modified to yield an average library insert length of approximately 250 base pairs. And (3) KAPA HiFi DNA Polymerase (Kapa Biosystems) was used in place of Herculase II DNA polymerase (Agilent). Paired-end sequencing was performed on NovaSeq instrumentation (Illumina).

Whole-transcriptome sequencing results were aligned using STAR^41^, and normalized expression values in transcripts per million (TPM) were calculated using the ImmunoID NeXT tool, Expressionist (Personalis). For RNA sequencing and alignment quality control, the following metrics were evaluated: average read length, average mapped read pair length, percentage of uniquely mapped reads, number of splice sites, mismatch rate per base, deletion/insertion rate per base, mean deletion/insertion length and anomalous read pair alignments, including inter-chromosomal and orphaned reads. The ImmunoID NeXT DNA and RNA Analysis Pipeline aligns reads to the hs37d5 reference genome build. The pipeline performs alignment, duplicate removal and base quality score recalibration using best practices outlined by the Broad Institute^42,43^. The pipeline uses Picard to remove duplicates and the Genome Analysis Toolkit to improve sequence alignment and correct base quality scores. Aligned sequence data are returned in BAM format according to SAM specification. Raw read counts from were also normalized using R to get weighted trimmed mean of the log expression ratios (trimmed mean of M values (TMM)).

To calculate gene expression signatures on a given gene set, scores were determined via geometric mean of the normalized count values of respective gene signatures. Patient tumor samples were collected from a range of primary tumors and metastatic sites. Sample sizes from pre-treatment liver biopsies for all gene signatures identified are as follows: nivo/chemo (*n* = 17); sotiga/chemo (*n* = 12); and sotiga/nivo/chemo (*n* = 12).

### Multiplex tissue staining and imaging

Tumor tissue was collected before treatment (fresh baseline biopsy or archival tissue), on-treatment (during cycle 2) and optionally at the end of treatment. Tissues were fixed in formalin followed by paraffin embedding. All tissue imaging was performed under the guidance of an expert pathologist (T.J.H.) in the Advanced Immunomorphology Platform Laboratory at Memorial Sloan Kettering Cancer Center. Primary antibody staining conditions were optimized using standard immunohistochemical staining on the Leica Bond RX automated research stainer with DAB detection (Leica Bond Polymer Refine Detection DS9800). Using 4-µm tissue sections and serial antibody titrations on control tonsil tissue, the optimal antibody concentration was determined, followed by transition to a seven-color multiplex assay with equivalency (see Supplementary Fig. 8 for control staining). Four antibody panels were used for staining. Panels A1 and B1 were used for tissues collected in phase 1b. Panels A2 and B2 were further optimized for distribution of cellular markers and were used for tissues collected in phase 2. Multiplex assay antibodies and conditions are described in Supplementary Table 12.

#### Seven-color multiplex imaging assay

FFPE tissue sections were baked for 3 hours at 62 °C in a vertical slide orientation with subsequent deparaffinization performed on the Leica Bond RX, followed by 30 minutes of antigen retrieval with Leica Bond ER2, followed by six sequential cycles of staining with each round including a 30-minute combined block and primary antibody incubation (Akoya antibody diluent/block). For Ki-67 and panCK, detection was performed using a secondary horseradish peroxidase (HRP)-conjugated polymer (Akoya Opal polymer HRP Ms+Rb; 10-minute incubation). Detection of all other primary antibodies was performed using a goat anti-mouse Poly HRP secondary antibody or goat anti-rabbit Poly HRP secondary antibody (Invitrogen, 10-minute incubation). The HRP-conjugated secondary antibody polymer was detected using fluorescent tyramide signal amplification using Opal dyes 520, 540, 570, 620, 650 and 690 (Akoya Biosciences). The covalent tyramide reaction was followed by heat-induced stripping of the primary/secondary antibody complex using Akoya AR9 buffer and Leica Bond ER2 (90% AR9 and 10% ER2) at 100 °C for 20 minutes preceding the next cycle. After six sequential rounds of staining, sections were stained with Hoechst 33342 (Invitrogen) to visualize nuclei and mounted with ProLong Gold antifade reagent mounting medium (Invitrogen).

#### Multispectral imaging and spectral unmixing

Seven-color multiplex stained slides were imaged using the Vectra Multispectral Imaging System version 3 (Akoya Biosciences). Scanning was performed at ×20 (×200 final magnification). Filter cubes used for multispectral imaging were DAPI, FITC, Cy3, Texas Red and Cy5. A spectral library containing the emitted spectral peaks of the fluorophores in this study was created using the Vectra image analysis software (Akoya Biosciences). Using multispectral images from single-stained slides for each marker, the spectral library was used to separate each multispectral cube into individual components (spectral unmixing), allowing for identification of the seven marker channels of interest using Inform 2.4 image analysis software.

#### mIF image analysis

Individual region of interest (ROI) images were exported to TIFF files and run through a pipeline for multiplexed imaging quality control and processing under the supervision of an expert pathologist. A machine learning cell segmentation algorithm was used to segment individual whole cells along the membrane border using nuclear as well as multiple membrane markers to enable drawing borders for all cell types. For each cell segment, pixel values within each region were averaged to give a single intensity value per cell and per marker. Using these single-cell intensity values, cell type assignments were made manually by a scientist determining cutoff points for positive marker expression for each sample. To do this manual thresholding, the distribution of single-cell marker values and the appearance of fluorescence on the images themselves were simultaneously inspected using CellEngine software along with Mantis Viewer, a custom in-house open-source software used for fluorescent image visualization (10.5281/zenodo.4009579), and thresholds for each marker were drawn per sample. Using these individual marker thresholds, cell types were defined by positivity of combined associated markers in the panel as described in Supplementary Table 13. Once cell types were defined, the percentage out of total cells and out of the parent population was calculated for each ROI. Then, for each sample, the median across ROIs was taken for percent of total cells, percent of parent population and occasionally percent of other relevant populations.

Sample sizes for cell populations identified using mIF (Extended Data Figs. 3g,h, 4c,d and 6d,e and Supplementary Fig. 5) from pre-treatment biopsies are as follows: nivo/chemo (*n* = 25); sotiga/chemo (*n* = 25); and sotiga/nivo/chemo (*n* = 29). Sample sizes for cell populations identified using mIF (Extended Data Fig. 3g,h) from on-treatment biopsies are as follows: nivo/chemo (*n* = 5); sotiga/chemo (*n* = 3); and sotiga/nivo/chemo (*n* = 6).

### Analysis of all data for association with survival and pharmacodynamic changes

#### Data storage and structure

All processed biomarker data were combined with cleaned clinical data and loaded into a proprietary in-house database called the Cancer Data & Evidence Library (CANDEL). CANDEL uses the database technology Datomic (www.datomic.com) and a suite of tools built to enable storage of molecular and clinical data and fast query and visualization from the R programming language.

#### Data analysis in R

All molecular data were analyzed for association with outcomes and treatment using the R programming language with the packages and versions listed in Supplementary Table 14. Association with survival was analyzed for cell population percentages, protein values and gene expression signatures by calculating the median value across all patients in all arms and then separating patients into two groups below the median and above or equal to the median. Between these two groups, for each arm, Kaplan–Meier plots were created, and log-rank *P* value significance was determined using the survminer and survival packages. To visualize differences between any defined groups or to visualize changes on treatment, ggplot2 and base R plotting were used. To determine differences between pre-treatment and on-treatment values, as well as differences between survival groups (>1 year and <1 year) at any given time point, a two-sided Wilcoxon sign-rank test with a significance cutoff of *P* = 0.05 was used. Median log fold change was calculated to determine additional pharmacodynamic differences seen from pre-treatment to on-treatment. Multivariable Cox proportional hazard models were also generated in relation to survival in each arm, with individual biomarkers in Supplementary Table 8 controlling for an additional clinical variable, de novo/recurrent staging at initial diagnosis or prior chemotherapy usage, using the survival and survminer packages. Forest plots were generated for most significant circulating biomarkers in each arm to determine hazard ratio and CI of each biomarker in relation to each other. Circus plots for multi-omic analysis were generated using the DIABLO method in the mixOmics R package. Heat maps were generated using pheatmap, and correlations were calculated using the Spearman method.

### Reporting Summary

Further information on research design is available in the Nature Research Reporting Summary linked to this article.

## Online content

Any methods, additional references, Nature Research reporting summaries, source data, extended data, supplementary information, acknowledgements, peer review information; details of author contributions and competing interests; and statements of data and code availability are available at 10.1038/s41591-022-01829-9.

### Supplementary information

Supplementary Information Supplementary Figs. 1–8, Supplementary Tables 1–14, Clinical Study Protocol and Statistical Analysis Plan

Reporting Summary

## Figures and Tables

**Extended Data Table 1:**
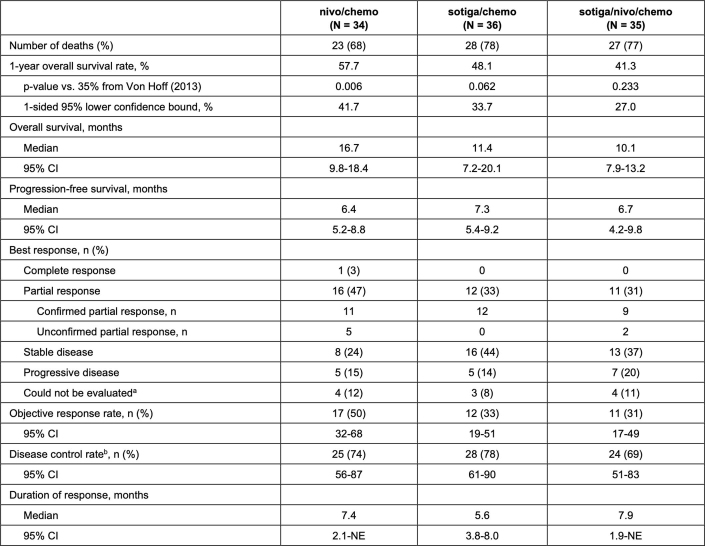
Clinical activity in the efficacy population

The efficacy population includes all randomized and dosed patients in Phase 2 and DLT-evaluable patients from Phase 1b enrolled at the recommended Phase 2 dose of sotiga. The 1-year overall survival (OS) rate and corresponding 1-sided, 95% lower confidence bound were estimated by the Kaplan-Meier method. P-values were calculated using a 1-sided, one-sample Z test of the Kaplan-Meier estimate of the 1-year OS rate (and its standard error) against the historical rate of 35%. P-values were not adjusted for multiple comparisons. OS, progression-free survival, and duration of response and the corresponding 2-sided, 95% CIs were estimated by the Kaplan-Meier method. Abbreviations: CI = confidence interval; N or n = number; NE = not estimable.

^a^Not evaluable includes patients who only had one tumor assessment with overall response of Not Evaluable (1 in nivo /chemo) or who did not have any post-baseline tumor assessments due to: initiation of another systemic cancer therapy after treatment discontinuation (1 in nivo /chemo, 2 in sotiga /chemo, 1 in sotiga/nivo/chemo), death (2 in sotiga/nivo/chemo), withdrawal of consent / lost to follow-up (1 each in nivo/chemo and sotiga/chemo), or inability due to clinical deterioration (1 each in nivo/chemo and sotiga/nivo/chemo).

^b^Disease control rate is defined as the proportion of patients with a best overall response of complete or partial response or stable disease at least 7 weeks after study drug initiation.

**Extended Data Table 2:**
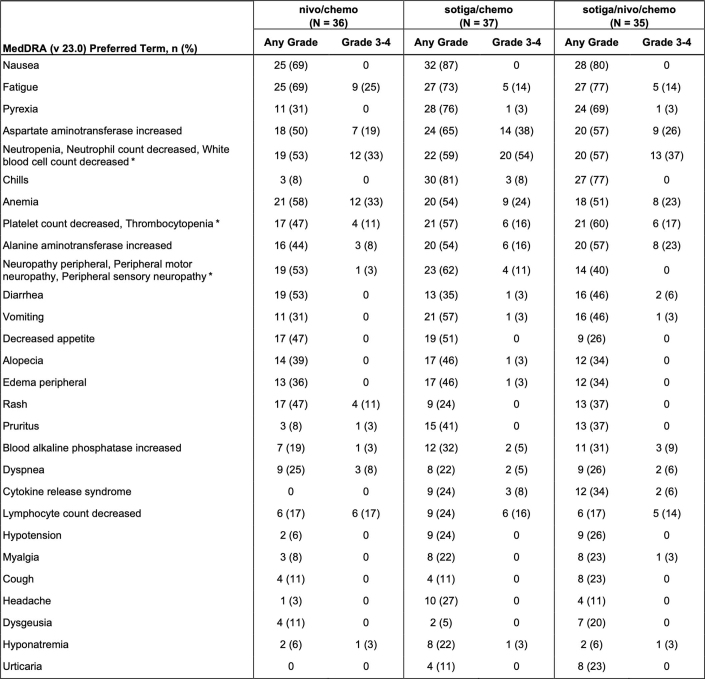
Treatment-related adverse events with incidence ≥ 20% in any arm

The safety population includes all Phase 1b and Phase 2 patients who received at least 1 dose of any study drug. For safety analyses, patients were grouped according to the study treatment actually received. Similar MedDRA preferred terms were combined into a single category to aid in interpretability for rows designated with an asterisk (*). Abbreviations: MedDRA = Medical Dictionary for Regulatory Activities; n or N = number.

## Data Availability

Summary datasets generated during and/or analyzed during the current study are available in the GitHub repository ParkerICI/prince-trial-data. These datasets include a de-identified limited clinical dataset with demographic and response information for each patient, processed RNA sequencing files and summary tables of cell proportions found via mIF, CyTOF and flow cytometry. The full clinical dataset generated in this study is considered commercially sensitive and, therefore, is not publicly available. Requests for additional clinical data should be emailed to the corresponding author and should include a brief description of the proposed analysis. Requests for data access will be reviewed individually, and a decision will be communicated within 4 weeks of receipt. Data might be shared in the form of aggregate data summaries and via a data transfer agreement, which will outline any potential restrictions on data use. Individual patient-level raw data containing confidential or identifiable patient information are subject to patient privacy and cannot be shared.

## Code availability

Mantis Viewer, a custom in-house, open-source software used for fluorescent image visualization, is available at 10.5281/zenodo.4009579.
