# Supplementary material for: Sotigalimab and/or nivolumab with chemotherapy in first-line metastatic pancreatic cancer: clinical and immunologic analyses from the randomized phase 2 PRINCE trial
Source: Nat Med. 2022 Jun 3;28(6):1167–77. doi: 10.1038/s41591-022-01829-9 (PMC9205784; doi:10.1038/s41591-022-01829-9)
Supplement: Supplementary file 1 — Supplementary Figs. 1–8, Supplementary Tables 1–14, Clinical Study Protocol and Statistical Analysis Plan [file 41591_2022_1829_MOESM1_ESM.pdf]

nature portfolio

https://doi.org/10.1038/s41591-022-01829-9

Supplementary information

Sotigalimab and/or nivolumab with chemotherapy in first-line metastatic pancreatic cancer: clinical and immunologic analyses from the randomized phase 2 PRINCE trial

In the format provided by the authors and unedited

Sotigalimab and/or nivolumab with chemotherapy in first-line metastatic pancreatic cancer: Clinical and immunologic analyses from the randomized Phase 2 PRINCE trial: Supplementary Information

Table of Contents

Supplementary Figure 1	Activated T cell frequencies increase with nivo/chemo treatment	3
Supplementary Figure 2	T cell phenotyping gating strategy	4
Supplementary Figure 3	Antigen-experienced non-naïve central memory T cells in the periphery are associated with survival in mPDAC patients treated with nivo/chemo	5
Supplementary Figure 4	Multivariable Cox Proportional Hazard Models reveal Tfh Cells and Cross Presenting DCs have the highest predictive value for nivo/chemo and sotiga/chemo, respectively.	6
Supplementary Figure 5	PD-L1 expression on tumor cells prior to treatment tends with longer survival in mPDAC patients treated with nivo/chemo	7
Supplementary Figure 6	CyTOF gating strategy	8
Supplementary Figure 7	Soluble molecules associated with dendritic cell maturation are associated with survival on-treatment (C1D15) in mPDAC patients treated with sotiga/chemo	9
Supplementary Figure 8	Single marker control images for mIF experiments using tonsil tissue from one donor	10
Supplementary Table 1	Demographic and Baseline Disease Characteristics for Patients in the Safety Population	11
Supplementary Table 2	Baseline Tumor PD-L1 and Mutational Status for Patients in the Efficacy Population	13
Supplementary Table 3	Treatment Exposure and Dose Modifications for Patients in the Efficacy Population	14
Supplementary Table 4	Post hoc Subgroup Analyses of Overall Survival	16
Supplementary Table 5	Summary of Adverse Events of Special Interest (AESI) by Highest Grade	18
Supplementary Table 6	Summary of Treatment Discontinuations Due to an Adverse Event	19
Supplementary Table 7	Select Immune Cell Population Definitions	20
Supplementary Table 8	Pretreatment Biomarker Factors Associated with Survival with Tumor and/or Immune Function Relevance	21
Supplementary Table 9	Gene Signatures	25
Supplementary Table 10	CyTOF Antibody Panel	26

Supplementary Table 11	T cell Phenotyping Antibody Panel (X50)	28
Supplementary Table 12	Multiplex Imaging Staining Panels	29
Supplementary Table 13	Cell Population Definitions Used in Multiplex Imaging Analysis	30
Supplementary Table 14	Packages Used in R Analysis	31
Clinical Study Protocol		32
Statistical Analysis Plan		168

Supplementary Figure 1

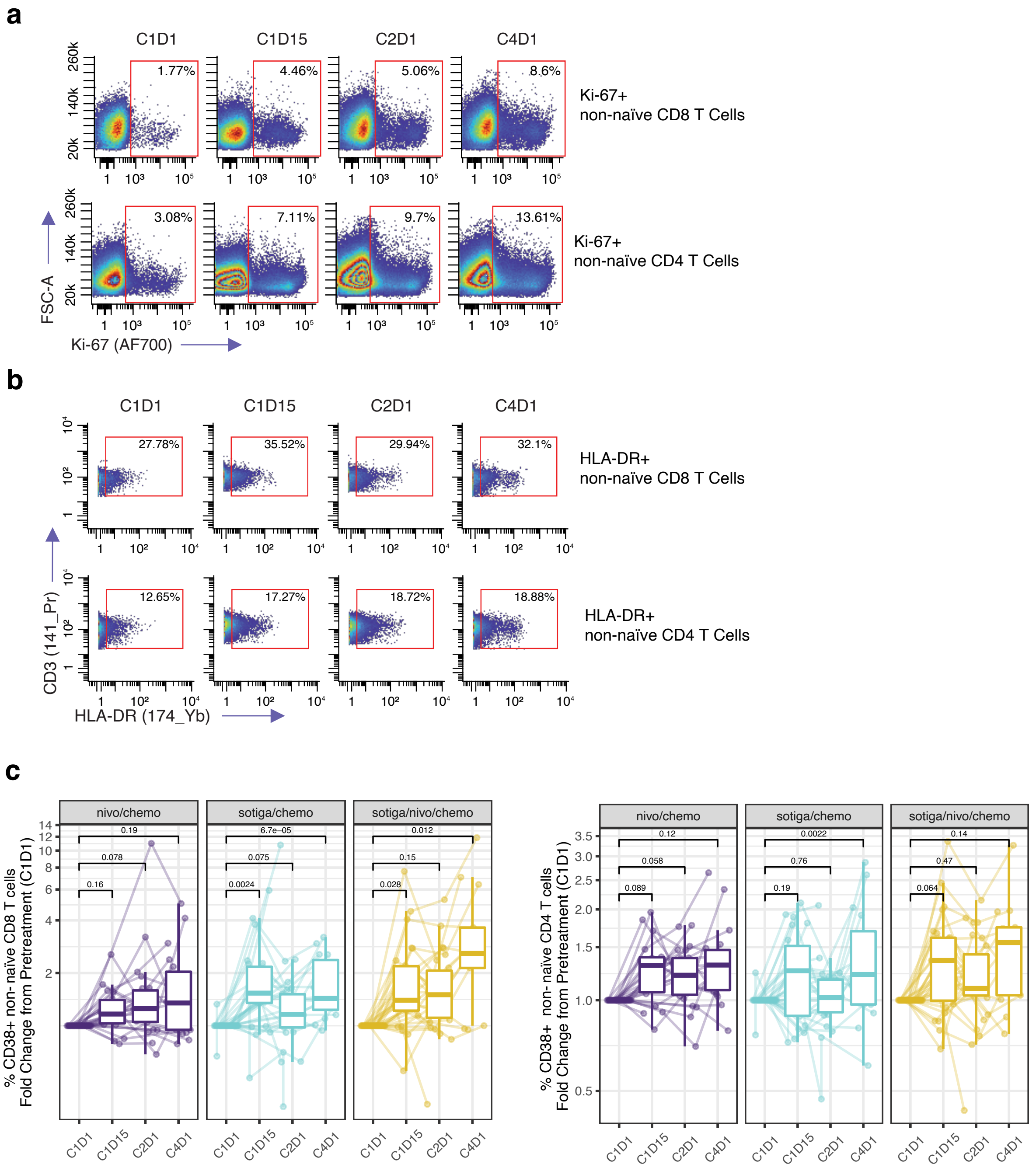

Supplementary Figure 1: Activated T cells frequencies increase with nivo/chemo treatment.

a, Representative flow plots using PBMC samples over time from a patient in the nivo/chemo treatment arm showing an increase in Ki-67+ non-naïve CD8 (top panel) and CD4 (bottom panel) T cells. **b,** Representative flow plots using PBMC samples over time from a patient in the nivo/chemo treatment arm showing an increase in HLA-DR+ non-naïve CD8 (top panel) and CD4 (bottom panel) T cells. **c,** Change in frequencies of circulating CD38+ non-naïve CD8 (left panel) and CD4 (right panel) T cells, as a fraction of total non-naïve CD8 or CD4 T cells respectively, in patients from each arm over the course of treatment. Box plots are shown as fold change relative to C1D1 (pretreatment) and plotted on a pseudo-log scale. Median values and quartiles are shown. The whiskers depict 95% CI. Individual patient values are shown and colored by survival status at 1 year. P-values for timeseries represent two-sided Wilcoxon signed-rank tests between timepoints, illustrating increases on-treatment. Sample sizes for cell populations shown (c): n = 26, 21, 25, 19; n = 28, 23, 27, 18; n = 32, 27, 29, 14 biologically independent samples at C1D1, C1D15, C2D1 and C4D1 in nivo/chemo, sotiga/chemo, sotiga/nivo/chemo treatment arms, respectively.

Supplementary Figure 2

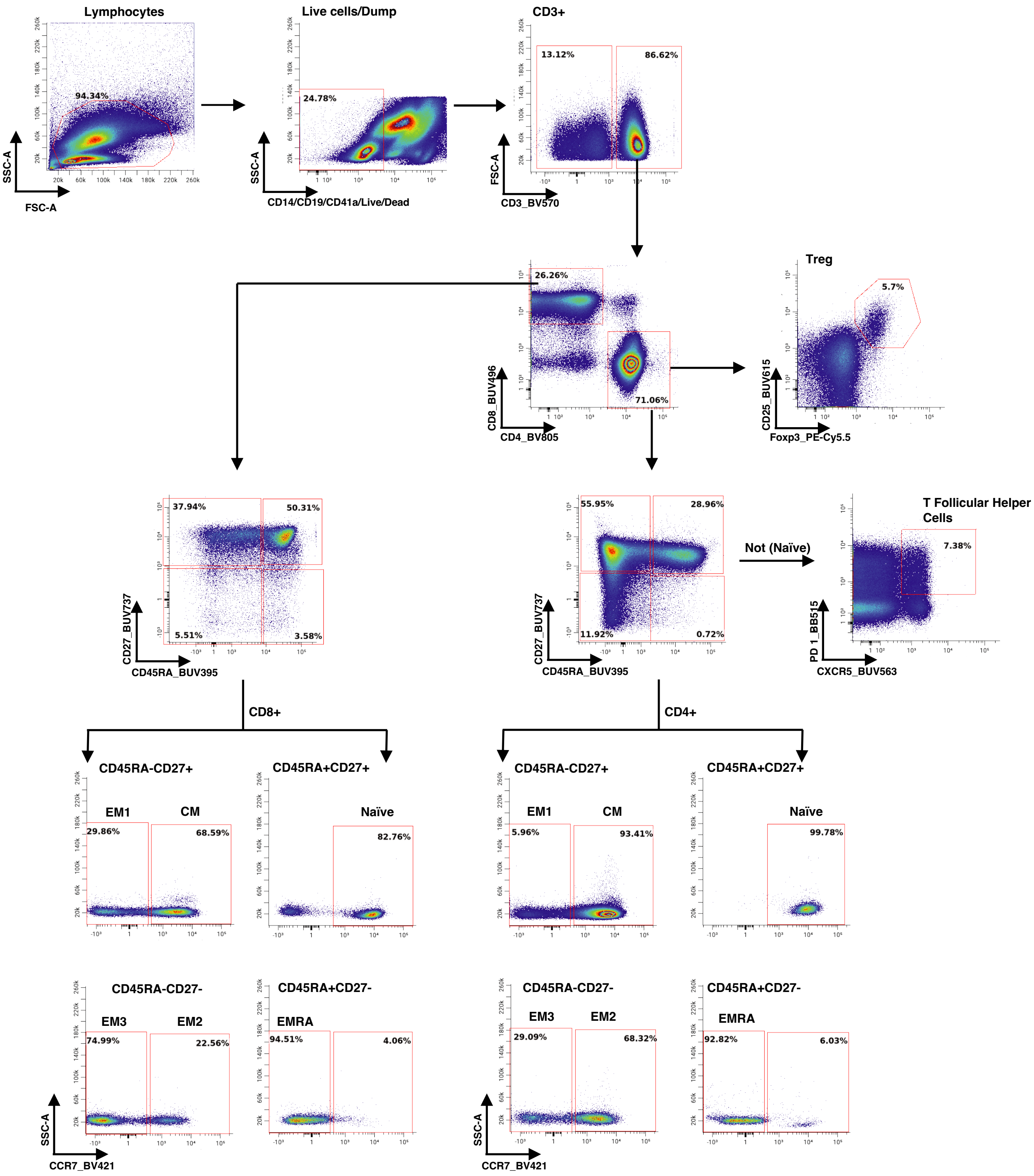

Supplementary Figure 2: T cell phenotyping gating strategy. Gating strategy used to define T cell populations by flow cytometry analysis. Representative flow plots are shown.

Supplementary Figure 3

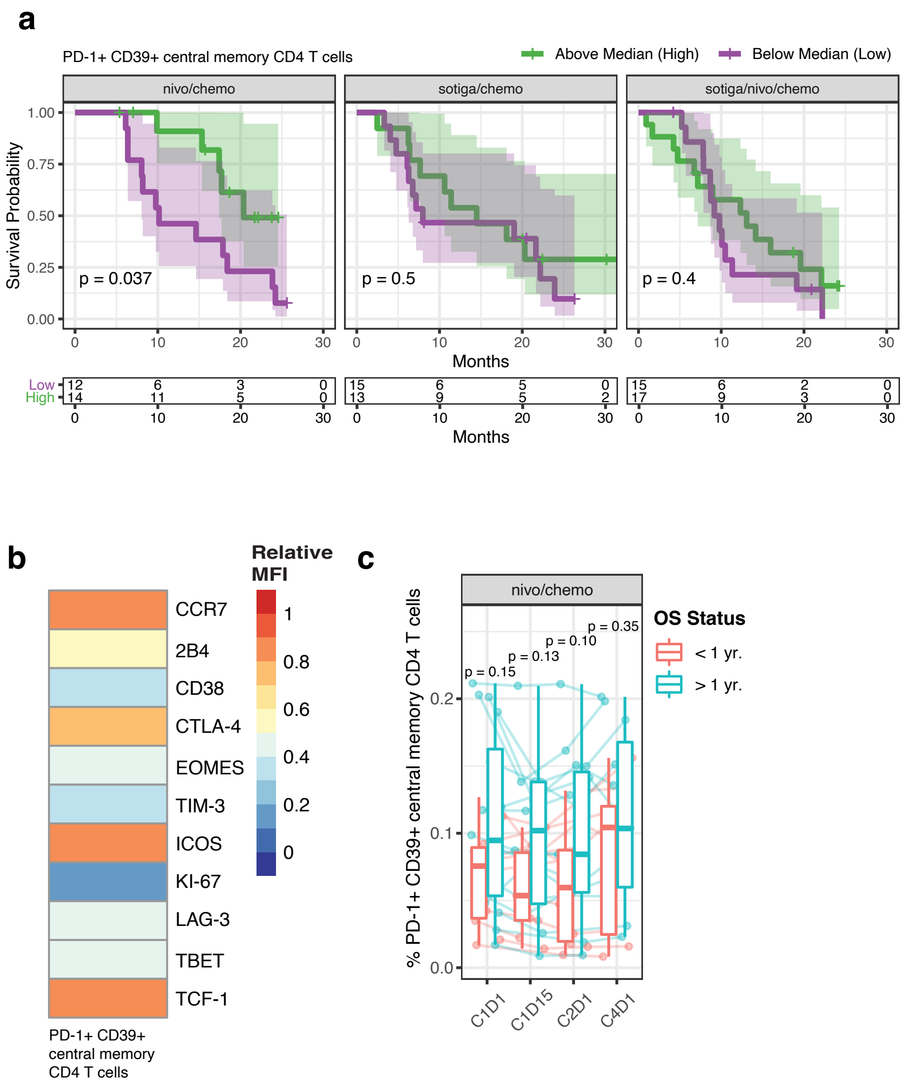

Supplementary Figure 3: Antigen-experienced non-naïve central memory T cells in the periphery are associated with survival in mPDAC patients treated with nivo/chemo.

a, KM curves for overall survival stratified by frequencies of circulating PD-1+ CD39+ central memory CD4 T cells above and below the median across all patients in all arms. **b,** Heatmap of relative median fluorescence intensity of proteins present on pretreatment PD-1+ CD39+ central memory CD4 T cells across all patients in the nivo/chemo arm. **c,** Frequencies of PD-1+ CD39+ central memory CD4 T cells pretreatment (C1D1) and on-treatment (C1D15, C2D1, C4D1). Box plots show median and quartiles and whiskers depict 95% CI. Individual patient values are shown and colored by survival status at 1 year. P-values for timeseries represent two-sided Wilcoxon signed-rank tests between survival groups at each timepoint. On KM curves, median values were determined using all data across the 3 arms, P-values are from a log-rank test between groups, and shaded regions illustrate 95% CI. Sample sizes for cell populations shown (c,): n = 26, 21, 25, 19 biologically independent samples at C1D1, C1D15, C2D1 and C4D1.

Supplementary Figure 4

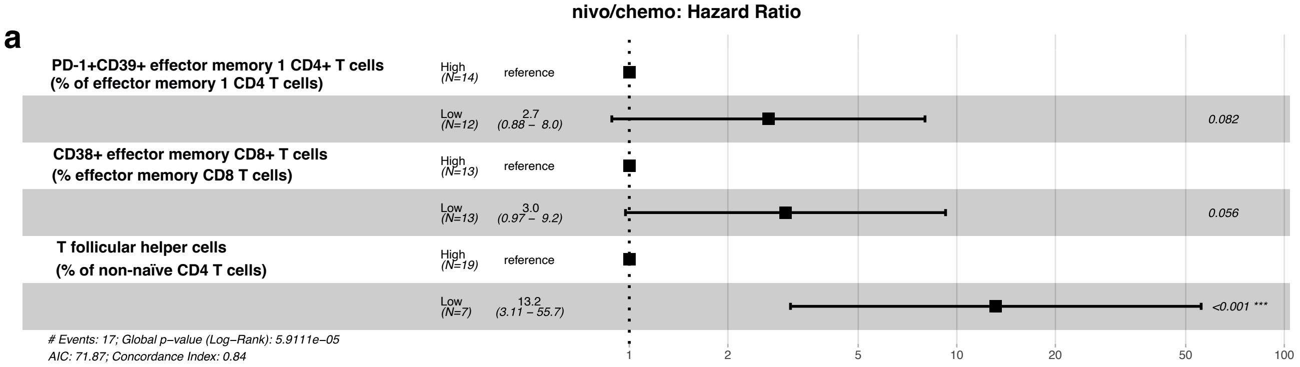

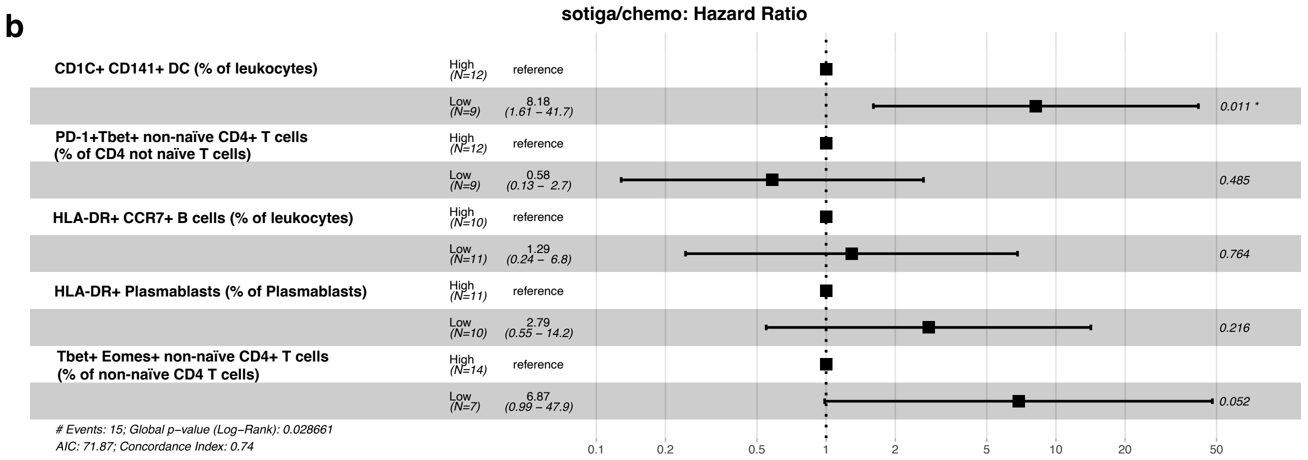

Supplementary Figure 4: Multivariable Cox Proportional Hazard Models reveal Tfh Cells and Cross Presenting DCs have the highest predictive value for nivo/chemo and sotiga/chemo, respectively.

Hazard Ratios from the multivariable Cox proportional models from the primary circulating biomarker features in the **a,** nivo/chemo treatment arm or in the **b,** sotiga/chemo treatment arm. Error bars represent +/- 1.96 standard error (SE).

Supplementary Figure 5

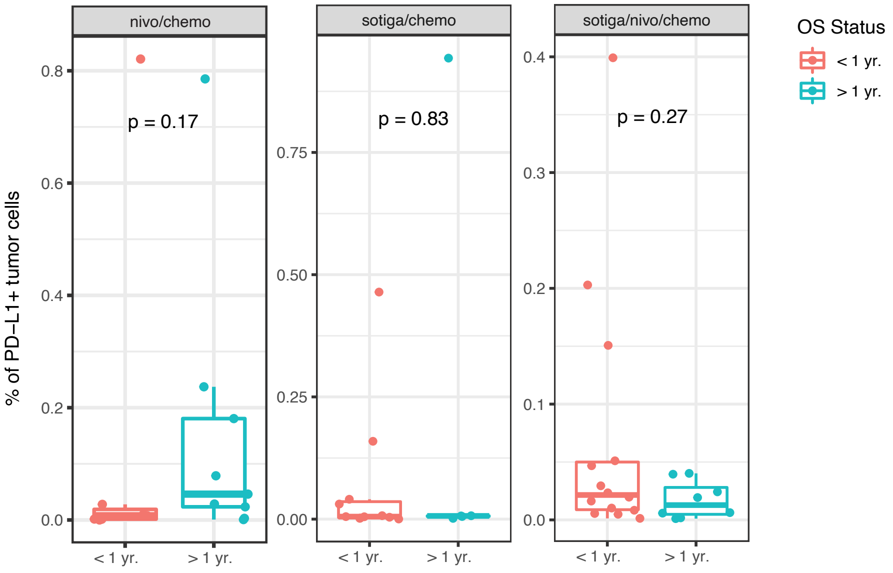

Supplementary Figure 5: PD-L1 expression on tumor cells prior to treatment trends with longer survival in mPDAC patients treated with nivo/chemo.
Percentage of tumor cells in pretreatment biopsies expressing PD-L1 by mIF, stratified by overall survival status at 1 year. P-value is a two-sided Wilcoxon signed-rank test between survival groups. Median values and quartiles are shown. The whiskers depict 95% CI. Individual patient values are shown and colored by survival status at 1 year.

Supplementary Figure 6

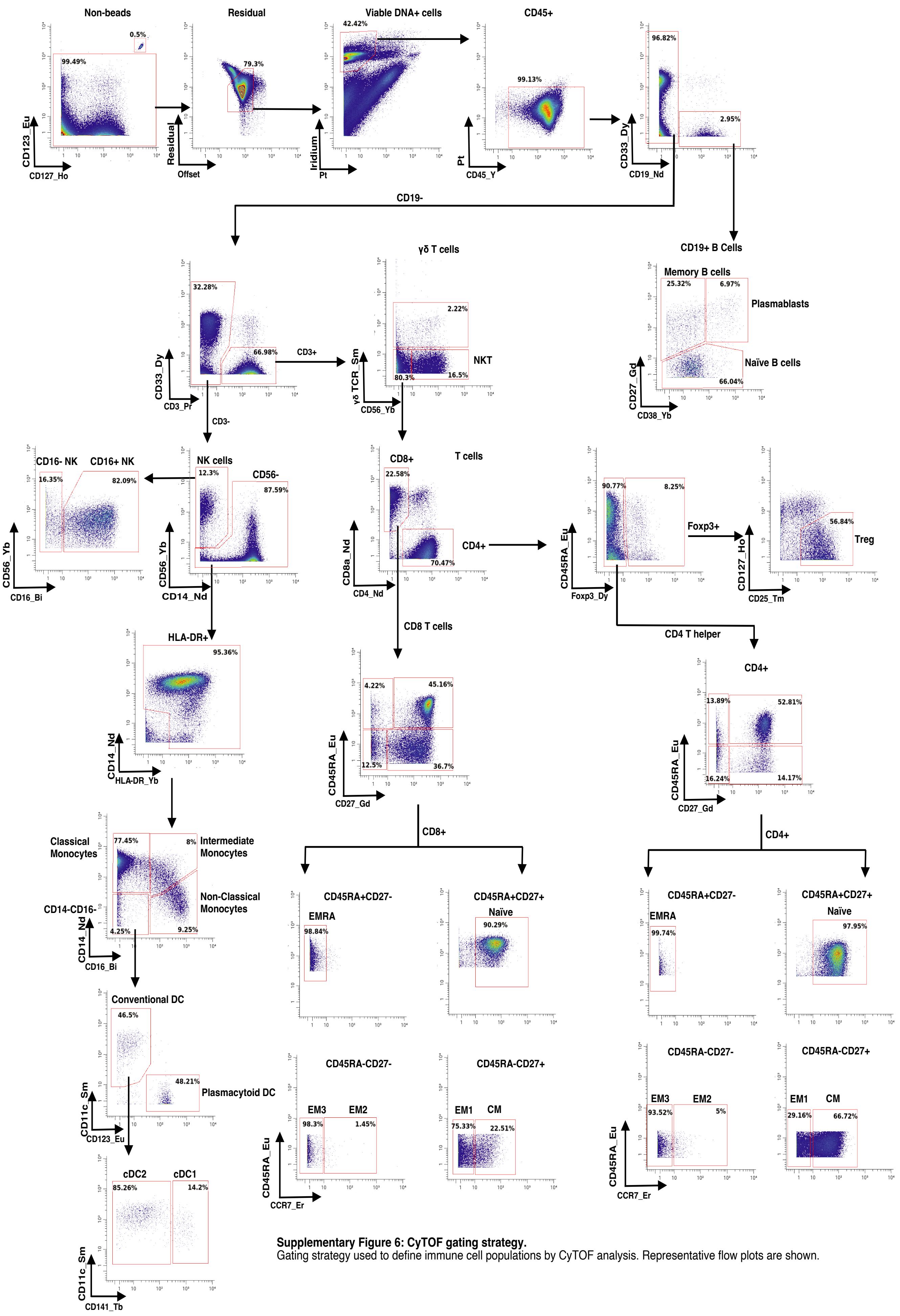

Supplementary Figure 6: CyTOF gating strategy.
Gating strategy used to define immune cell populations by CyTOF analysis. Representative flow plots are shown.

Supplementary Figure 7

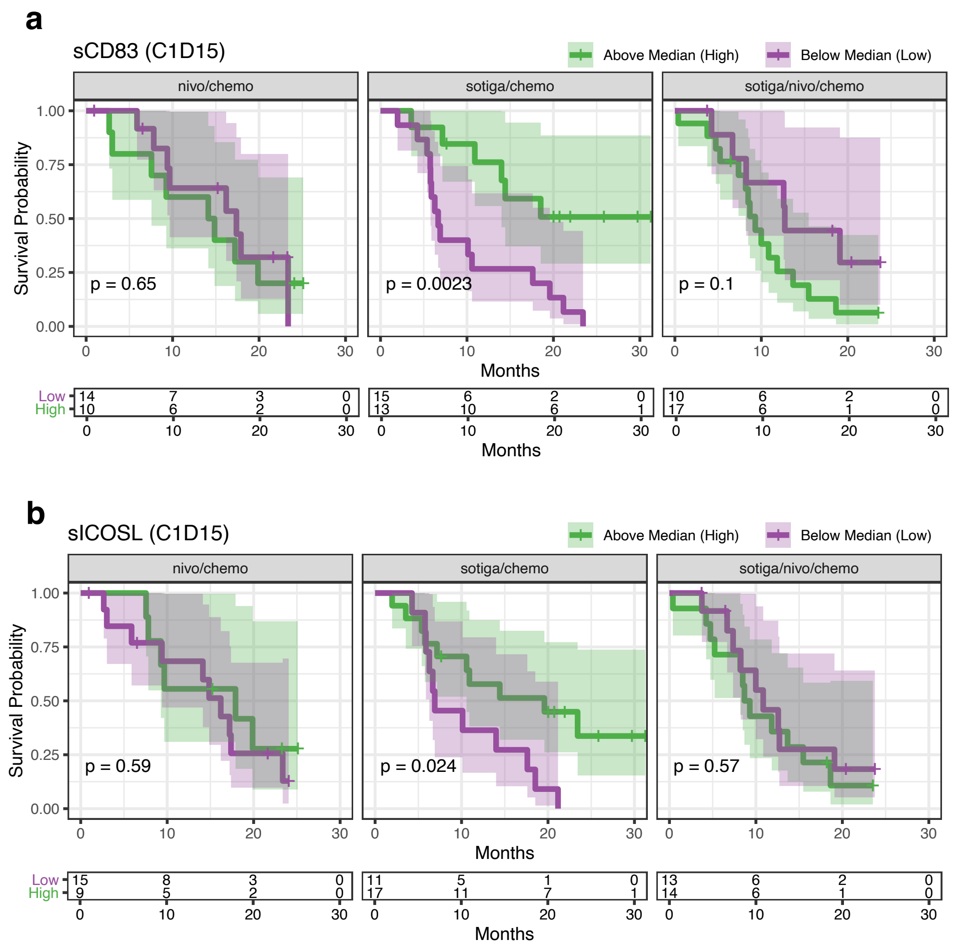

Supplementary Figure 7: Soluble molecules associated with dendritic cell maturation are associated with survival on-treatment (C1D15) in mPDAC patients treated with sotiga/chemo.

a, Kaplan-Meier (KM) curves for overall survival stratified by soluble CD83 (sCD83) protein expression above and below the median signature value across all patients in all arms at C1D15. **b,** KM curves for overall survival stratified by soluble ICOSL (sICOSL) protein expression above and below the median value across all patients in all arms at C1D15. On KM curves, median values were determined using all data across the 3 arms, P-values are from a log-rank test between groups, and shaded regions illustrate 95% CI.

Supplementary Figure 8

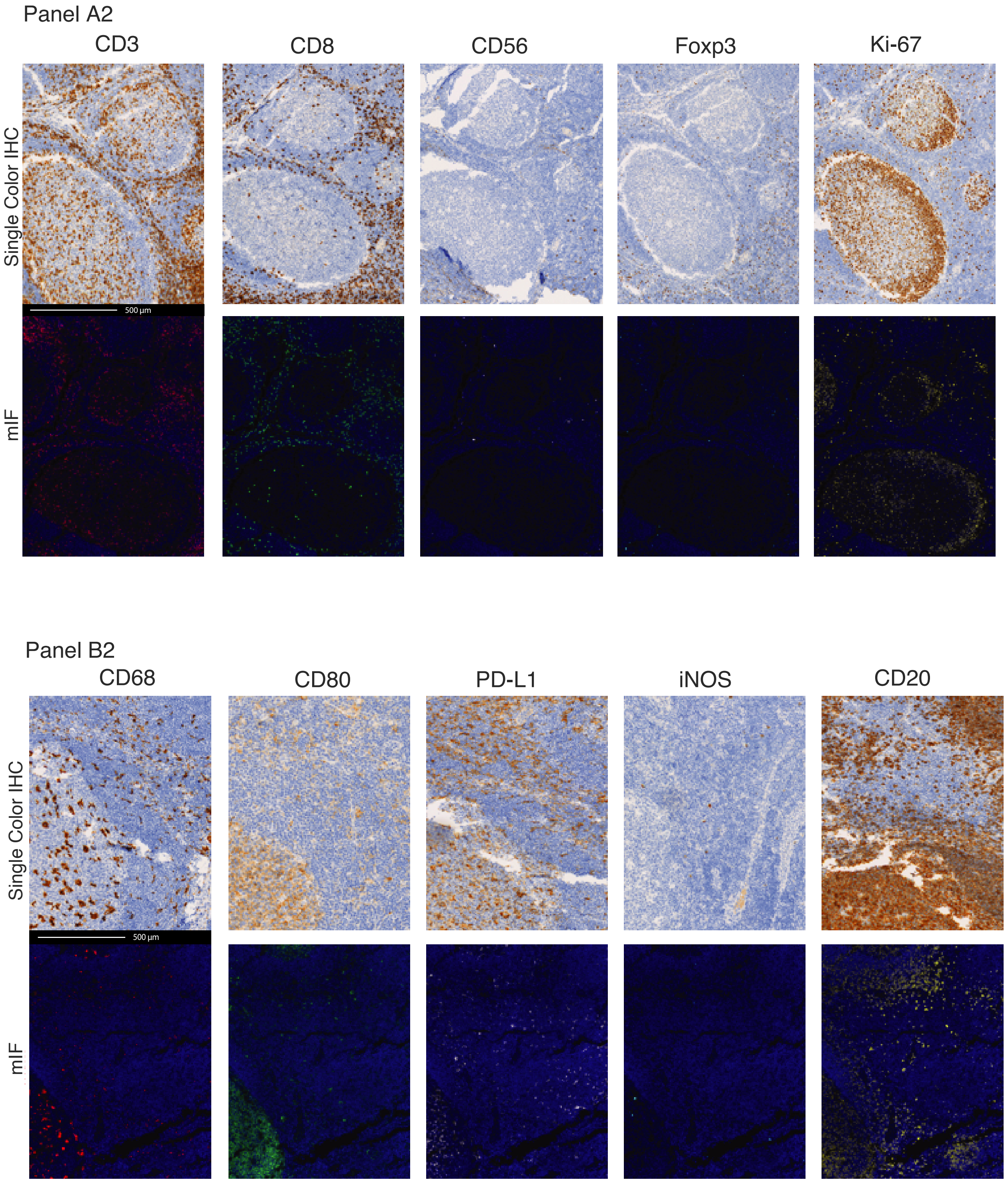

Supplementary Figure 8: Single marker control images for mIF experiments using tonsil tissue from one donor.
Equivalency of single-marker optimized antibody immunohistochemistry (IHC) developed with 3, 3'-diaminobendidine (DAB) on human tonsil tissue (top rows) with corresponding multiplexed immunofluorescence (mIF) on tonsil tissue from an individual donor (bottom rows). The immunofluorescent images represent individual marker position within the 7-color assay performed. The magnification used was 20x (0.499 μm/pixel).

**Supplementary Table 1. Demographic and Baseline Disease Characteristics for Patients in the Safety Population.**			
	**nivo/chemo (N = 36)**	**sotiga/chemo (N = 37)**	**sotiga/nivo/chemo (N = 35)**
**Characteristic**			
Age — years			
Median (range)	61.5 (41 – 75)	61.0 (35 – 78)	62.0 (39 – 78)
≥65 years, n (%)	14 (39)	15 (41)	14 (40)
Sex, n (%)			
Female	14 (39)	13 (35)	17 (49)
Male	22 (61)	24 (65)	18 (51)
Race and ethnic group, n (%)			
Asian	3 (8)	4 (11)	1 (3)
Black	0	3 (8)	2 (6)
White	31 (86)	29 (78)	30 (86)
Other	2 (6)	1 (3)	2 (6)
Hispanic	1 (3)	1 (3)	1 (3)
ECOG performance status, n (%)			
0	16 (44)	20 (54)	16 (46)
1	20 (56)	17 (46)	19 (54)
Pancreatic tumor location, n (%)			
Head	15 (42)	17 (46)	19 (54)
Body	13 (36)	10 (27)	9 (26)
Tail	8 (22)	10 (27)	7 (20)
Select sites of metastatic disease, n (%)			
Liver	29 (81)	30 (81)	27 (77)
Lung	11 (31)	11 (30)	11 (31)
Peritoneum	9 (25)	10 (27)	11 (31)
Stage at initial PDAC diagnosis, n (%)			
Stages I–III	8 (22)	9 (24)	9 (26)
Stage IV	28 (78)	28 (76)	26 (74)
Time from diagnosis to first dose – months, median (range) [a]	1.3 (0.4 – 69.8)	1.0 (0.2 – 29.1)	1.1 (0.4 – 29.6)
Prior cancer treatment, n (%)			
Chemotherapy	10 (28)	7 (19)	6 (17)
Radiation therapy	7 (19)	1 (3)	5 (14)
Surgery	12 (33)	11 (30)	8 (23)

Abbreviations: ECOG = Eastern Cooperative Oncology Group; N or n = number; PDAC = pancreatic ductal adenocarcinoma.

Includes all Phase 1b and Phase 2 patients who received at least 1 dose of any study drug. For safety analyses, patients were grouped according to the study treatment actually received.

[a] Calculations exclude one participant from nivo/chemo who did not report a date of diagnosis.

**Supplementary Table 2.** **Baseline Tumor PD-L1 and Mutational Status for Patients in the Efficacy Population.**			
**Characteristic**	**nivo/chemo (N = 34)**	**sotiga/chemo (N = 36)**	**sotiga/nivo/chemo (N = 35)**
PD-L1+ tumor percentage[a]			
> 1%[b], n/number evaluable (%)	10/18 (56)	7/19 (37)	14/24 (58)
Somatic tumor genomic variant mutation data available[b], n (%)	23 (79)	19 (69)	21 (63)
KRAS, n/number evaluable[c] (%)	19/23 (83)	16/19[d](89)	15/21[e] (76)
G12D mutation	9/23 (33)	7/19 (24)	7/21 (14)
G12R mutation	2/23 (11)	1/19 (4)	3/21 (9)
G12V mutation	7/23(22)	5/19 (24)	5/21 (27)
G12S mutation	0/23 (0)	1/19 (4)	0/21 (0)
Q61H mutation	1/23 (4)	2/19 (8)	1/21 (5)
Q61R mutation	0/23 (0)	1/19 (4)	0/21 (5)
MSI-high, n/number evaluable (%)	1/23 (1)	0/19 (0)	1/21 (5)
SMAD, n/number evaluable (%)	4/23 (15)	3/19 (12)	1/21(9)
TP53, n/number evaluable (%)	16/23 (70)	14/19 (74)	11/21 (64)
BRCA1, n/number evaluable[f] (%)	0/23 (0)	0/19 (0)	0/21 (0)
BRCA2, n/number evaluable[f] (%)	0/23 (0)	0/19 (0)	1/21 (5)

Abbreviations: MSI = microsatellite instability; N or n = number.

Includes all randomized and dosed patients in Phase 2 and DLT-evaluable patients from Phase 1b enrolled at the recommended Phase 2 dose of sotiga.

[a] PD-L1+ was assayed with a multiplex research assay and tumor percentage was calculated in a method most similar to the Combined Positive Score (CPS). However, PD-L1+ tumor percentages were assessed by multiplex IHC on multiple regions of interest on a single FFPE tumor sample slide analyzed by computational methods and, thus, are not directly comparable to single-marker IHC assays assessed by a trained pathologist.

[b] Data are unavailable for tumor mutation analysis due to not having a pre-treatment tumor sample of sufficient quality for DNA sequencing for 11, 17, and 14 patients in the nivo/chemo, sotiga/chemo, and sotiga/nivo/chemo arms, respectively.

[c] No other KRAS mutations detected.

[d] One patient in the sotiga/chemo arm had two *KRAS* variants detected. This patient's tumor tissue had detectable *G12S* and *Q16H* mutations.

[e] One patient in the sotiga/nivo/chemo arm had two *KRAS* variants detected. This patient's tumor tissue had detectable *G12D* and *G12R* mutations.

[f] For *BRCA1* and *BRCA2*, mutations tabulated include all mutations found in the tumor, including those also present in sequenced normal (germline) samples. All other genes include only somatic tumor mutations, ie those not found in the normal tissue.

Supplementary Table 3. Treatment Exposure and Dose Modifications for Patients in the Efficacy Population.

	nivo/chemo (N = 34)	sotiga/chemo (N = 36)	sotiga/nivo/chemo (N = 35)
**Treatment exposure**			
Treatment duration (months), median (range)	5.2 (0-19)	5.1 (0-20)	4.7 (0-24)
Chemotherapy treatment cycles, median (range)	6.0 (1-23)	6.0 (1-22)	6.0 (1-25)
Patients who received ≥1 dose, n (%)			
Sotigalimab	0	34 (94)	33 (94)
Nivolumab	34 (100)	0	35 (100)
Gemcitabine	34 (100)	36 (100)	35 (100)
nab-Paclitaxel	34 (100)	36 (100)	35 (100)
Relative dose intensity, median (IQR), %			
Sotigalimab	--	100 (81-100)	100 (100-100)
Nivolumab	89 (74-100)	--	100 (83-100)
Gemcitabine	76 (58-95)	80 (64-89)	68 (52-88)
nab-Paclitaxel	69 (52-89)	71 (60-84)	68 (51-88)
Cumulative dose, median (IQR)			
Sotigalimab, mg/kg	--	1.7 (1.2-2.6)	1.5 (1.2-2.4)
Nivolumab, mg	2,280 (960-3,300)	--	2,400 (960-3,120)
Gemcitabine, mg/m^2	14,200 (6,200-18,000)	14,400 (9,480-21,320)	11,080 (5,800-16,600)
nab-Paclitaxel, mg/m^2	1,388 (768-2,108)	1,788 (1,020-2,444)	1,385 (728-2,063)
**Dose modifications**			
Patients with ≥1 dose reduction, n (%)	21 (62)	25 (69)	22 (63)
Sotigalimab, mg/kg	--	7 (19)	1 (3)
Nivolumab, mg	0	--	0
Gemcitabine, mg/m^2	21 (62)	25 (69)	22 (63)
nab-Paclitaxel, mg/m^2	25 (74)	26 (72)	22 (63)
Patients with ≥1 dose not administered, n (%)			
Sotigalimab, mg/kg	--	17 (47)	13 (37)
Nivolumab, mg	22 (65)	--	17 (49)
Gemcitabine, mg/m^2	22 (65)	27 (75)	25 (71)
nab-Paclitaxel, mg/m^2	23 (68)	28 (78)	24 (69)
Patients with ≥1 dose interrupted, n (%)			
Sotigalimab, mg/kg	--	21 (58)	13 (37)
Nivolumab, mg	2 (6)	--	2 (6)
Gemcitabine, mg/m^2	1 (3)	0	2 (6)

nab-Paclitaxel, mg/m^2	1 (3)	2 (6)	0

Abbreviations: IQR = interquartile range; kg = kilogram; m^2 = meters squared; mg = milligram; N or n = number.

Includes all randomized and dosed patients in Phase 2 and DLT-evaluable patients from Phase 1b enrolled at the recommended Phase 2 dose of sotiga.

**Supplementary Table 4. Post hoc Subgroup Analyses of Overall Survival.**				
**Treatment Arm**	**Subgroup**	**N**	**1-year OS Rate [95% CI]**	**Median OS (months) [95% CI]**
All Patients				
nivo/chemo		34	57.7% [38-73]	16.7 [9.8-18.4]
sotiga/chemo		36	48.1% [31-63]	11.4 [7.2-20.1]
sotiga/nivo/chemo		35	41.3% [24-58]	10.1 [7.9-13.2]
All Patients, grouped by treatment received[a]				
nivo/chemo		36	57.3% [39-72]	15.3 [9.8-18.4]
sotiga/chemo		36	48.1% [31-63]	11.4 [7.2-20.1]
sotiga/nivo/chemo		33	40.8% [23-58]	10.1 [7.9-14.0]
Stage at Initial Diagnosis				
nivo/chemo	Stages I-III	7	85.7% [33-98]	16.7 [6.4-NE]
	Stage IV	27	50.8% [30-68]	14.6 [8.2-23.9]
sotiga/chemo	Stages I-III	9	77.8% [37-94]	22.2 [5.9-23.9]
	Stage IV	27	38.5% [20-56]	9.3 [6.3-18.1]
sotiga/nivo/chemo	Stages I-III	9	59.3% [19-85]	12.3 [0.9-NE]
	Stage IV	26	36.7% [19-55]	9.0 [7.2-13.2]
Received Prior Radiation Therapy				
nivo/chemo	Prior Radiation	7	85.7% [33-98]	17.4 [6.4-NE]
	No Prior Radiation	27	50.8% [30-68]	14.6 [8.2-18.4]
sotiga/chemo	Prior Radiation	1	100% [NE-NE]	14.9 [NE-NE]
	No Prior Radiation	35	46.6% [29-62]	11.1 [6.8-20.1]
sotiga/nivo/chemo	Prior Radiation	4	33.3% [1-77]	10.1 [9.2-NE]
	No Prior Radiation	31	42.2% [24-59]	9.8 [7.8-13.2]
Primary Tumor Location in Pancreas				
nivo/chemo	Head	14	76.0% [42-92]	17.4 [9.8-NE]
	Body or Tail	20	44.9% [22-66]	10.2 [6.4-17.9]
sotiga/chemo	Head	17	68.2% [40-85]	20.3 [7.7-23.9]
	Body or Tail	19	31.6% [13-52]	7.4 [6.1-18.1]
sotiga/nivo/chemo	Head	19	50.0% [26-70]	11.8 [8.7-19.6]
	Body or Tail	16	30.1% [10-54]	8.7 [4.2-13.1]
Presence of Liver Lesions at Baseline				
nivo/chemo	Liver lesion(s)	28	56.7% [36-73]	15.3 [9.8-17.9]
	No liver lesions	6	66.7% [20-90]	NR [6.1-NE]
sotiga/chemo	Liver lesion(s)	29	34.8% [18-52]	7.8 [6.3-18.1]
	No liver lesions	7	100% [NE-NE]	23.9 [14.5-NE]
sotiga/nivo/chemo	Liver lesion(s)	27	32.9% [16-51]	9.0 [6.7-13.1]
	No liver lesions	8	71.4% [26-92]	14.0 [7.9-22.2]
Baseline CA19-9 Level[b]				

nivo/chemo	< 59x ULN	9	76.2% [33-94]	NR [2.8-NE]
	≥ 59x ULN	21	45.4% [22-66]	10.2 [8.0-17.7]
sotiga/chemo	< 59x ULN	15	49.0% [22-72]	11.4 [6.3-22.9]
	≥ 59x ULN	13	15.4% [3-39]	6.8 [4.8-10.6]
sotiga/nivo/chemo	< 59x ULN	18	41.5% [19-63]	10.5 [4.7-14.2]
	≥ 59x ULN	4	34.8% [11-60]	8.7 [5.7-13.2]
Baseline Albumin Level				
nivo/chemo	< 4 g/dL	15	51.9% [25-74]	14.6 [3.5-17.4]
	≥ 4 g/dL	19	62.7% [35-81]	17.9 [9.9-24.2]
sotiga/chemo	< 4 g/dL	20	36.8% [17-58]	7.4 [6.3-19.0]
	≥ 4 g/dL	16	61.4% [33-81]	18.1 [7.7-22.2]
sotiga/nivo/chemo	< 4 g/dL	16	37.5% [15-60]	9.5 [4.7-13.1]
	≥ 4 g/dL	19	44.9% [21-67]	11.3 [7.2-19.1]
Baseline Neutrophil-Lymphocyte Ratio				
nivo/chemo	< 5	18	80.0% [50-93]	18.4 [10.2-NE]
	≥ 5	16	33.3% [12-56]	8.2 [3.5-16.7]
sotiga/chemo	< 5	25	59.3% [38-76]	19.0 [10.6-22.2]
	≥ 5	11	20.0% [3-48]	6.3 [2.5-8.0]
sotiga/nivo/chemo	< 5	19	59.0% [33-78]	14.2 [9.0-22.2]
	≥ 5	16	20.3% [5-43]	7.2 [4.7-11.3]

Abbreviations: CI = confidence interval; N = sample size; NE = not estimable; NR = not reached; OS = overall survival; ULN = upper limit of normal.

Includes all randomized and dosed patients in Phase 2 and DLT-evaluable patients from Phase 1b enrolled at the recommended Phase 2 dose of sotiga.

The subgroup analyses displayed in this table were not pre-specified in the protocol or statistical analysis plan. The 1-year overall survival (OS) rate, median OS, and corresponding 2-sided, 95% CIs were estimated by the Kaplan-Meier method. Results were not adjusted for multiple comparisons.

[a] Two Phase 2 patients were randomly allocated to sotiga/nivo/chemo but only received chemo and nivo (i.e., sotiga was not received); these patients are grouped as nivo/chemo for the as-treated analysis.

[b] Fifteen patients (4 in nivo/chemo, 8 in sotiga/chemo, and 3 in sotiga/nivo/chemo) were excluded from the CA19-9 subgroup analysis because CA19-9 was not measured for these patients at baseline.

**Supplementary Table 5. Summary of Adverse Events of Special Interest (AESI) by Highest Grade.**			
	**nivo/chemo (N = 36)**	**sotiga/chemo (N = 37)**	**sotiga/nivo/chemo (N = 35)**
Patients with at least one AESI, n (%)	28 (78)	33 (89)	31 (89)
Cytokine release syndrome	0	9 (24)	12 (34)
Grade 1	0	0	1 (3)
Grade 2	0	6 (16)	9 (26)
Grade 3	0	3 (8)	2 (6)
Increased liver function test results	24 (67)	30 (81)	26 (74)
Grade 1	5 (14)	1 (3)	5 (14)
Grade 2	7 (19)	11 (30)	6 (17)
Grade 3	12 (33)	18 (49)	14 (40)
Grade 4	0	0	1 (3)
Infusion related reaction	2 (6)	5 (14)	5 (14)
Grade 1	0	2 (5)	0
Grade 2	1 (3)	2 (5)	5 (14)
Grade 3	1 (3)	1 (3)	0
Thrombocytopenia	18 (50)	21 (57)	22 (63)
Grade 1	5 (14)	7 (19)	5 (14)
Grade 2	8 (22)	8 (22)	10 (29)
Grade 3	3 (8)	4 (11)	6 (17)
Grade 4	2 (6)	2 (5)	1 (3)

Abbreviations: AESI = adverse event of special interest; N or n = number

Includes all Phase 1b and Phase 2 patients who received at least 1 dose of any study drug. For safety analyses, patients were grouped according to the study treatment actually received.

Adverse events were graded according to the National Cancer Institute Common Terminology Criteria for Adverse Events (NCI CTCAE), version 4.03. As a limitation, due to overlapping characteristics, there is potential for variability in assessment between terms (e.g., infusion related reaction and cytokines release syndrome), which may lead to under or over-representation of incidence of specific terms.

Cytokine release syndrome is defined as an adverse event with a MedDRA Preferred Term matching 'Cytokine release syndrome', regardless of seriousness, severity or relationship to study drugs.

Increased liver function test results is defined as an adverse event with a MedDRA Preferred Term matching 'Alanine aminotransferase increased', 'Aspartate aminotransferase increased', 'Blood alkaline phosphatase increased', 'Blood bilirubin increased', 'Hepatic enzyme increased' or 'Hyperbilirubinaemia', regardless of seriousness, severity or relationship to study drugs.

Infusion related reaction is defined as an adverse event with a MedDRA Preferred Term matching 'Infusion related reaction', regardless of seriousness, severity or relationship to study drugs.

Thrombocytopenia is defined as an adverse event with a MedDRA Preferred Term matching 'Platelet count decreased' or 'Thrombocytopenia', regardless of seriousness, severity or relationship to study drugs.

**Supplementary Table 6. Summary of Treatment Discontinuations Due to an Adverse Event.**			
**Treatment Arm**	**Adverse Event Term (MedDRA Preferred Term)**	**Toxicity Grade**	**Study Drug(s) Assessed as Related per Investigator**
nivo/chemo	Hyperbilirubinemia	Grade 2	gemcitabine, nab-paclitaxel
	Myocarditis	Grade 3	nivolumab
	Neuropathy peripheral	Grade 2	nivolumab, nab-paclitaxel
	Pneumonitis	Grade 3	nivolumab, nab-paclitaxel, gemcitabine
	Thrombotic microangiopathy	Grade 4	gemcitabine
	Thrombotic microangiopathy	Grade 3	gemcitabine
sotiga/chemo	Pneumonitis	Grade 3	gemcitabine, nab-paclitaxel
sotiga/nivo/chemo	Pyrexia	Grade 2	sotigalimab, gemcitabine

Abbreviations: MedDRA = Medical Dictionary for Regulatory Activities.

Includes all Phase 1b and Phase 2 patients who received at least 1 dose of any study drug. For safety analyses, patients were grouped according to the study treatment actually received.

Supplementary Table 7. **Select Immune Cell Population Definitions.**	
**Immune cell population**	**Markers used to define population**
Non-naïve T cells	(CD45RA-CD27+), (CD45RA-CD27-), (CD45RA+CD27-)
Effector memory T cells	CD45RA-CCR7-
Effector memory 1 (EM1) T cells	CD45RA-CD27+CCR7-
Effector memory 2 (EM2) T cells	CD45RA-CD27-CCR7+
Effector memory 3 (EM3) T cells	CD45RA-CD27-CCR7-
Central memory T cells	CD45RA-CD27+CCR7+
Terminally differentiated effector memory T cells (EMRA)	CD45RA+CD27-
Conventional dendritic cells	HLA-DR+CD14-CD16-CD11c+
Cross presenting dendritic cells	HLA-DR+CD14-CD16-CD11c+CD141+
B cells	CD19+
Monocytic myeloid derived suppressor cells	CD14+CD16-HLA-DRlo

Supplemental Table 8. Pretreatment Biomarker Factors Associated with Survival with Tumor and/or Immune Function Relevance.

			nivo/chemo	sotiga/chemo	sotiga/nivo/ chemo
**Classification**	**Biomarker feature**	**Sample type**	**Overall survival association of above median biomarker values (Log rank P-value)**	**Overall survival association of above median biomarker values (Log rank P-value)**	**Overall survival association of above median biomarker values (Log rank P-value)**
Proliferating and/or activated T cells	Soluble CD27	Serum	Shorter survival (0.018)[1,2]	Not significant (NS) (0.49)	NS (0.14)
	CD38+ non-naïve CD8+ T cells (% non-naïve CD8 T cells)	PBMC	NS (0.094)	NS (0.21)	Shorter survival (0.016)[1,2]
	CD38+ CD4+ non-naïve T cells (% of CD4 not naïve)	PBMC	NS (0.25)	NS (0.61)	Shorter survival (0.019)[1,2]
	CD38+ effector memory CD8+ T cells (% effector memory CD8 T cells)	PBMC	Longer survival (0.047)[1]	NS (0.12)	NS (0.057)
	HLA-DR+ T cells (% of leukocytes)	PBMC	NS (0.36)	NS (0.086)	Longer survival (0.014)[1,2]
	Ki-67+ T cells (% of CD3+ cells)	PBMC	Longer survival (0.022)[1,2]	NS (0.92)	Longer survival (0.021)
	Soluble PD-1	Serum	Longer survival (0.045)[1]	NS (0.066)	NS (0.23)
	Interleukin 2 (IL-2)	Serum	NS (0.62)	NS (0.8)	Shorter survival (0.0092)[1,2]
"Antigen-experienced" T cells	PD-1+CD39+ effector memory 1 CD4+ T cells (% of effector memory 1 CD4 T cells)	PBMC	Longer survival (0.0037)[1,2]	NS (0.87)	NS (0.57)
	PD-1+CD39+ central memory CD4+ T cells (% of central memory CD4 T cells)	PBMC	Longer survival (0.037)	NS (0.5)	NS (0.4)
	PD-1+Tbet+ non-Naïve CD4+ T cells (% of CD4 not naïve T cells)	PBMC	NS (0.55)	Longer survival (0.004)[1,2]	NS (0.6)
Cytotoxic T cells	NKT cells (% of leukocytes)	PBMC	Longer survival (0.0049)[1,2]	NS (0.78)	NS (0.5)
	CD8+ EMRA T cells (% of leukocytes)	PBMC	Longer survival (0.0054)[1,2]	NS (0.57)	NS (0.21)
CD4 T helper cell response	T follicular helper cells (% of non-naïve CD4 T cells)	PBMC	Longer survival (<0.0001)[1,2]	NS (0.12)	NS (0.66)
	Ki-67- Foxp3- CD4+ T cells (% of CD4 T cells)	Tumor tissue	NS (0.73)	Longer survival (0.0048)[1,2]	Longer survival (0.023)

Supplemental Table 8. Pretreatment Biomarker Factors Associated with Survival with Tumor and/or Immune Function Relevance.

			nivo/chemo	sotiga/chemo	sotiga/nivo/chemo
**Classification**	**Biomarker feature**	**Sample type**	**Overall survival association of above median biomarker values (Log rank P-value)**	**Overall survival association of above median biomarker values (Log rank P-value)**	**Overall survival association of above median biomarker values (Log rank P-value)**
Type 1 immune response	Tbet+ T cells (% of CD3+ cells)	PBMC	Longer survival (0.035)[1,2]	Longer survival (0.022)[1,2]	NS (0.25)
	Tbet+ Eomes+ non-naïve CD4+ T cells (% of non-naïve CD4 T cells)	PBMC	NS (0.23)	Longer survival (0.013)[1,2]	NS (0.44)
	$Tbet^{+}$ TCRγδ+ T cells (% of TCRγδ T cells)	PBMC	Longer survival (0.0083)[1,2]	Longer survival (0.0024)[1,2]	NS (0.89)
	Th1 response gene signature	Tumor tissue	NS (0.14)	Longer survival (0.024)	NS (0.052)
	IFN- γ response gene signature	Tumor tissue	NS (0.62)	Longer survival (0.012)	NS (0.42)
Type 2 immune response	Interleukin 13 (IL-13)	Serum	NS (0.16)	NS (0.066)	Shorter survival 0.0036[1,2]
	Interleukin 4 (IL-4)	Serum	NS (0.26)	Longer survival (0.046)[2]	Shorter Survival (0.011)[1,2]
	Th2 response gene signature	Tumor tissue	NS (0.92)	Longer survival (0.021)	NS (0.11)
Regulatory T cells	ICOS+ Tregs (% of Tregs)	PBMC	NS (0.99)	Longer survival (0.0013)[1,2]	NS (0.94)
	CTLA-4+ Tregs (% of Tregs)	PBMC	NS (0.28)	Longer survival (0.028)[1,2]	NS (0.3)
	TCF-7+ Tregs (% of Tregs)	PBMC	NS (0.4)	Longer survival (0.03)[1,2]	NS (0.67)
	Ki-67- Tregs (% of Tregs)	Tumor tissue	NS (0.34)	Longer survival (0.025)[1,2]	NS (0.67)
Immune checkpoint molecules	2B4+ non-naïve CD4+ T cells (% of non-naïve CD4 T cells)	PBMC	NS (0.44)	Shorter survival (0.019)[2]	NS (0.44)
Activated, possibly GC related B cells	HLA-DR+ CCR7+ B cells (% of leukocytes)	PBMC	NS (0.71)	Longer survival (0.038)	NS (0.68)
Dendritic cell biology	Soluble CD83	Serum	Longer survival (0.049)	NS (1)	NS (0.55)
	CD205	Serum	NS (0.72)	Longer survival (0.018)[1,2]	NS (0.75)
	LAMP3	Serum	NS (0.15)	Longer survival (0.05)	NS (0.31)
	CD1C+ CD141+ DC (% of leukocytes)	PBMC	NS (0.13)	Longer survival (0.043)[1]	NS (0.25)
	CD40+ pDC (% of pDC)	PBMC	NS (0.81)	Longer survival (0.041)[1,2]	NS (0.97)

Supplemental Table 8. Pretreatment Biomarker Factors Associated with Survival with Tumor and/or Immune Function Relevance.

			nivo/chemo	sotiga/chemo	sotiga/nivo/chemo
Classification	Biomarker feature	Sample type	Overall survival association of above median biomarker values (Log rank P-value)	Overall survival association of above median biomarker values (Log rank P-value)	Overall survival association of above median biomarker values (Log rank P-value)
B cell Immunity	HLA-DR+ Plasmablasts (% of Plasmablasts)	PBMC	NS (0.31)	Longer survival (0.0015)[1,2]	NS (0.13)
Macrophage (innate immunity)	PD-L1- macrophages (% of total cells)	Tumor tissue	NS (0.94)	Longer survival (0.0038)[1,2]	NS (0.39)
	iNOS+ macrophages (% of total cells)	Tumor tissue	Shorter Survival (0.040)	NS (0.81)	NS (0.2)
	CD80- macrophages (% of total cells)	Tumor tissue	NS (0.94)	Longer survival (0.034)	NS (0.37)
Immune cell co-stimulation	Soluble CD137	Serum	NS (0.98)	NS (0.30)	Shorter survival (0.00083)[1,2]
Chemokines	Chemokine (C-C motif) ligand 7 (CCL7)	Serum	NS (0.13)	NS (0.12)	Shorter survival (0.00024)[1,2]
	Chemokine (C-C motif) ligand 19 (CCL19)	Serum	NS (0.27)	Shorter survival (0.019)[1,2]	NS (0.4)
	Chemokine (C-C motif) ligand 23 (CCL23)	Serum	Shorter survival (0.040)[1]	Shorter survival (0.00072)[1,2]	NS (0.088)
Promotes leukocyte migration	Hematopoietic cell-specific Lyn Substrate 1 (HCLS1)	Serum	Shorter survival (0.0096)[1,2]	NS (0.19)	NS (0.13)
Inflammatory response	TNF-*α* gene signature	Tumor tissue	Shorter survival (0.00094)[1,2]	NS (0.59)	NS (0.87)
Immunosuppression	Nitric oxide synthase 3 (NOS3)	Serum	Shorter survival (0.011)[1,2]	NS (0.30)	NS (0.48)
	Carbonic anhydrase (CAH9)	Serum	Shorter survival (0.016)[1,2]	NS (0.91)	NS (0.93)
	Arginase 1 (ARG1)	Serum	Shorter survival (0.018)[1,2]	NS (0.31)	NS (0.41)
	Galectin 9 (Gal-9)	Serum	NS (0.19)	Shorter survival (0.011)[1,2]	Shorter survival (0.00046)[1,2]
	Interleukin 10 (IL-10)	Serum	NS (0.76)	Shorter Survival 0.021	NS (0.2)
	Interleukin 6 (IL-6)	Serum	Shorter survival (0.035)[1,2]	Shorter survival (0.0067)[1,2]	Shorter survival (< 0.0001)[1,2]
	Interleukin 8 (IL-8)	Serum	NS (0.17)	Shorter survival (0.0086)[1,2]	NS (0.12)
	Hepatocyte growth factor (HGF)	Serum	NS (0.074)	Shorter survival (0.0012)[1,2]	Shorter survival (0.0015)[1,2]
	Matrix metallopeptidase 7 (MMP7)	Serum	NS (0.19)	Shorter Survival (0.0033)[1,2]	NS (0.53)
	Matrix metallopeptidase 12 (MMP12)	Serum	NS (0.72)	Shorter survival (0.029)[1,2]	NS (0.17)
	IL-6 JAK/STAT3 gene signature	Tumor tissue	Shorter survival (0.0098)[1,2]	NS (0.84)	NS (0.62)

Supplemental Table 8. Pretreatment Biomarker Factors Associated with Survival with Tumor and/or Immune Function Relevance.

			nivo/chemo	sotiga/chemo	sotiga/nivo/ chemo
**Classification**	**Biomarker feature**	**Sample type**	**Overall survival association of above median biomarker values (Log rank P-value)**	**Overall survival association of above median biomarker values (Log rank P-value)**	**Overall survival association of above median biomarker values (Log rank P-value)**
	E2F targets gene signature	Tumor tissue	NS (0.99)	Shorter survival (0.021)	NS (0.28)
	TGF-β gene signature	Tumor tissue	Shorter survival (0.0011)[1,2]	Shorter survival (0.032)[1]	NS (0.18)
	CD14+ HLA-DRlo m-MDSC (% of leukocytes)	PBMC	NS (0.91)	Shorter survival (0.050)	NS (0.97)
Tumor metabolism	Fatty acid metabolism gene signature	Tumor tissue	Longer survival (0.0068)[1,2]	NS (0.46)	NS (0.27)
	Oxidative phosphorylation gene signature	Tumor tissue	Longer survival (0.013)[1]	NS (0.76)	NS (0.98)
	Xenobiotic metabolism gene signature	Tumor tissue	Longer survival (0.0068)[1,2]	NS (0.46)	NS (0.27)
	Peroxisome gene signature	Tumor tissue	Longer survival (0.024)[1]	NS (0.17)	NS (0.27)
	Bile acid metabolism gene signature	Tumor tissue	Longer survival (0.0068)[1,2]	NS (0.46)	NS (0.27)
	Glycolysis gene signature	Tumor tissue	NS (0.95)	Shorter survival (0.036)[1]	NS (0.52)
Angiogenesis	Angiopoietin-1 Receptor (TIE2)	Serum	NS (0.72)	Shorter survival (0.00032)[1,2]	NS (0.92)
	Vascular endothelial growth factor A (VEGF A)	Serum	Shorter survival (0.030)[1,2]	NS (0.64)	Shorter survival (0.00036)[1,2]
	VEGF gene signature	Tumor tissue	Shorter survival (0.0082)[1,2]	NS (0.95)	NS (0.39)
Apoptosis	Caspase 8	Serum	Shorter survival (0.044)[2]	NS (0.12)	NS (0.2)

Abbreviations: NS = not significant.

Orange font indicates biomarker associated (log rank p-value < 0.05) or trending (0.05 < p-value < 0.1) with shorter survival; blue font indicates biomarker associated (log rank p- value < 0.05) or trending (0.05 < p-value < 0.1) with longer survival; PBMC evaluated with multiplex immune panels using X50 and/or Cytof analysis; tumor tissue cell populations in the TME were identified using multiplex immunofluorescent imagining or gene expression signatures were identified using RNA-sequencing; serum was analyzed for proteomics using Olink.

1. This biomarker was found to be significant in a separate multivariate Cox proportional hazard model of survival when accounting for initial stage of diagnosis within the patients of the arm using median as a cutoff.
2. This biomarker was found to be significant in a separate multivariate Cox proportional hazard model of survival when accounting for prior chemotherapy treatment within the patients of the arm using median as a cutoff.

**Supplemental Table 9. Gene Signatures.**	
**Signature**	**Gene list**
Th1	*CXCR3, IFNG, IFNG-AS1, IL12RB1, IL18R1, IL18RAP, STAT1, STAT4, TBX21*
Th2	*GATA3, IL4, IRF4, MAF, STAT5A, STAT6*
Th17	*AHR, BATF, CCR6, IL17A, IL17F, IL21, IL6R, RORC, RUNX1, STAT3*
TLS-fDC	*CD2, CR1, FCER2, HLA-DRA*
TLS-memory B cell	*CD27, CD69, CD86, CR2, CXCR3, IGHD, MS4A1*
B cell	*BTLA, FCRL5, IDO1, IFNG, IGLL5, JCHAIN, MZB1*
IFN-γ response	*CD8A, CD274, LAG3, STAT1*

Note: This table includes gene signatures other than HALLMARK gene signatures, which can be found in the Molecular Signatures Database (MSigDB).

**Supplementary Table 10.** CyTOF Antibody Panel.							
**Mass**	**Element**	**Target**	**Clone**	**Source**	**Cat #**	**Biology**	**Staining**
89	Y	CD45	H130	Fluidigm	3089003B	Pan	Surface
113	In	CD66cd	YTH71.3	Invitrogen	Custom	Granulocytes	Surface
115	In	CD7	M-T701	BD Biosciences	Custom	T cell subset/NK/monocyte	Surface
140	Ce	CD86	IT2.2	BioLegend	Custom	T cell costim/inhibitory	Surface
141	Pr	CD3	UCHT1	Fluidigm	3141019B	Pan T cells	Surface
142	Nd	CD19	HIB19	Fluidigm	3142001B	Pan B cells	Surface
143	Nd	CD117 (c-kit)	104D2	Fluidigm	3143001B	Mast cells/ primitive immune cells	Surface
144	Nd	CD11b	IRCF44	Fluidigm	3144001B	Macrophage/monocyte	Surface
145	Nd	CD4	RPA-T4	Fluidigm	3145001B	T cell subset/monocyte	Surface
146	Nd	CD8a	RPA-T8	Fluidigm	3146001B	T cell subset/NK	Surface
147	Sm	CD11c	BU15	Fluidigm	3147008B	DC/macrophage/monocy te	Surface
148	Nd	CD14	RMO52	Fluidigm	3148010B	Macrophage/monocyte	Surface
149	Sm	CD1c (BDCA1)	L161	BioLegend	Custom	DC	Surface
150	Nd	FcER1	AER-37	Fluidigm	3150027B	DC/pDC/basophil	Surface
151	Eu	CD123 (IL-3Ra)	6H6	Fluidigm	3151001B	pDC/basophil	Surface
152	Sm	gdTCR	11F2	Fluidigm	3152008B	gd T cell	Surface
153	Eu	CD45RA	HI100	Fluidigm	3153001B	T cell naïve/memory	Surface
154	Sm	CD366 (TIM3)	F38-2E2	Fluidigm	3154010B	Checkpoint	Surface
155	Gd	CD64	10.1	BioLegend	Custom	FcgammaRI	Surface
156	Gd	CD274 (PD-L1)	29E.2A3	Fluidigm	3156026B	Checkpoint	Surface
157	Gd	CD206	15-2	BioLegend	Custom	Macrophage	Surface
158	Gd	CD27	L128	Fluidigm	3158010B	B/T cell memory	Surface
159	Tb	CD141	1A4	BioLegend	Custom	mDC	Surface
160	Gd	Tbet	4B10	Fluidigm	3160010B	Th1 polarization/NK	Intra-cellular
161	Dy	CD152 (CTLA-4)	14D3	Fluidigm	3161004B	Checkpoint	Intra-cellular
162	Dy	Foxp3	PCH101	Fluidigm	3162011A	Treg	Intra-cellular
163	Dy	CD33	WM53	Fluidigm	3163023B	Pan myeloid	Surface
164	Dy	CD45RO	UCHL1	Fluidigm	3164007B	T cell naïve/memory	Surface
165	Ho	CD127 (IL-7Ra)	A019D5	Fluidigm	3165008B	T cell subset/Treg	Surface
166	Er	CD154 (CD40L)	24-31	BioLegend	Custom	T cell activation	Surface
167	Er	CD197 (CCR7)	G043H7	Fluidigm	3167009A	T cell subset (eff/memory)	Surface

Supplementary Table 10. **CyTOF Antibody Panel.**

Mass	Element	Target	Clone	Source	Cat #	Biology	Staining
168	Er	Ki67	B56	Fluidigm	3168007B	Proliferation	Intra-cellular
169	Tm	CD25	2A3	Fluidigm	3169003B	Treg	Surface
170	Er	TCR Va24-Ja18	6B11	Fluidigm	3170015B	iNKT	Surface
171	Yb	CD40	5C3	BioLegend	Custom	APC	Surface
172	Yb	CD38	H1T2	Fluidigm	3172007B	B cell/NK/plasma cell	Surface
173	Yb	CD192 (CCR2)	K036C2	BioLegend	Custom	Chemokine receptor	Surface
174	Yb	HLA-DR	L243	Fluidigm	3174001B	APC	Surface
175	Lu	PD-1 (Nivo)/anti-IgG4	HP6025	Selleck Chem/ Southern Bio	Custom	Checkpoint	Surface
176	Yb	CD56	NCAM16.2	Fluidigm	3176008B	NK	Surface
209	Bi	CD16	3G8	Fluidigm	3209002B	Fc receptor/NK/ neutrophil/monocyte	Surface

**Supplementary Table 11.** T cell Phenotyping Antibody Panel (X50).						
**Fluorophore**	**Target**	**Clone**	**Source**	**Cat #**	**Category**	**Staining**
BUV395	CD45RA	HL100	BD	740298	Differentiation	Surface
BUV496	CD8a	RPA-T8	BD	612942	Lineage	Surface
BUV563	CD185 (CXCR5)	RF8B2	BD	741316	Lineage	Surface
BUV615	CD25	2A3	BD	612996	Lineage/ activation	Surface
BUV661	CD226 (DNAM-1)	DX11	BD	749934	Activation	Surface
BUV737	CD27	L128	BD	612829	Differentiation	Surface
BUV805	CD4	SK3	BD	612887	Lineage	Surface
BV421	CD197 (CCR7)	G043H7	Biolegend	353208	Differentiation	Surface
BV480	CD223 (LAG3)	T47-530	BD	746609	Exhaustion	Surface
BV510	Fixable Viability Stain (FVS)	n/a	BD	564406	Dump	Surface
BV510	CD14	M5E2	BD	740163	Dump	Surface
BV510	CD19	SJ25C1	BD	562947	Dump	Surface
BV510	CD41a	HIP8	BD	563250	Dump	Surface
BV570	CD3	UCHT1	Biolegend	300436	Lineage	Surface
BV605	CD137 (4-1BB)	4B4-1	BD	745256	Activation	Surface
BV650	CD244 (2B4)	2-69	BD	custom	Activation/ exhaustion	Surface
BV711	CD366 (Tim3)	7D3	BD	565566	Exhaustion	Surface
BV750	CD39	TU66	BD	747079	Exhaustion	Surface
BV786	CD28	CD28.2	BD	740996	Differentiation	Surface
BB515	CD279 (PD-1) + anti-Human IgG4	nivolumab, G17-4	Selleck Chem; BD	A2002; custom	Activation/ exhaustion	Surface
BB660	CD278 (ICOS)	DX29	BD	custom	Activation	Surface
BB700	CD127 (IL-7Ra)	HIL-7R-M21	BD	566398	Differentiation	Surface
BB790	CD38	HIT2	BD	custom	Differentiation/ activation	Surface
PE	TIGIT	MBSA43	eBioscience	12-9500-42	Exhaustion	Surface
PE-eFluor610	Eomes	WD1928	eBioscience	61-4877-42	Differentiation/ exhaustion	Intra-cellular
PE-Cy5	CD152 (CTLA-4)	BNI3	BD	555854	Exhaustion	Intra-cellular
PE-Cy5.5	FoxP3	PCH101	eBioscience	35-4776-42	Lineage	Intra-cellular
PE-Cy7	T-bet	O4-46	BD	custom	Lineage/activation	Intra-cellular
AF647	TCF-1 (TCF7)	S33-966	BD	566693	Differentiation	Intra-cellular
AF700	Ki-67	B56	BD	561277	Proliferation	Intra-cellular
APC-Fire750	KLRG1	SA231A2	Biolegend	367718	Exhaustion	Surface

Supplementary Table 12. Multiplex Imaging Staining Panels.

Marker	Antibody clone	Source	Dilution	Detection dye (cycle)
**Panel A1**				
**Marker**	**Antibody clone**	**Source**	**Dilution**	**Detection dye (cycle)**
CD8	C8/114B	CST	0.042 ug/mL	Opal 520 (1)
Ki67	SP6	Biocare	1:100	Opal 540 (2)
CD68	PG-M1	Dako	0.15 ug/mL	Opal 570 (3)
FOXP3	236A/E7	Biocare	1:2	Opal 620 (4)
PDL1	73-10	Abcam	0.18 ug/mL	Opal 650 (5)
panCK	AE1/AE3	DAKO	0.665 ug/mL	Opal 690 (6)
**Panel B1**				
**Marker**	**Antibody clone**	**Source**	**Dilution**	**Detection Dye (cycle)**
CD3	BC33	Biocare	1:200	Opal 520 (1)
INOS	13F5.1	Millipore	1:2500	Opal 540 (2)
Granzyme B	EPR8260	Abcam	1:100	Opal 570 (3)
CD20	E7B7T	CST	0.055 ug/mL	Opal 620 (4)
CD56	MRQ-42	CellMarque	1:2	Opal 650 (5)
panCK	AE1/AE3	DAKO	0.665 ug/mL	Opal 690 (6)
**Panel A2**				
**Marker**	**Antibody clone**	**Source**	**Dilution**	**Detection Dye (cycle)**
CD3	BC33	Biocare	1:200	Opal 520 (1)
Ki67	SP6	Biocare	1:100	Opal 540 (2)
CD56	MRQ-42	CellMarque	1:2	Opal 570 (3)
FOXP3	236A/E7	Biocare	1:2	Opal 620 (4)
CD8	C8/114B	CST	0.042 ug/mL	Opal 650 (5)
panCK	AE1/AE3	DAKO	0.665 ug/mL	Opal 690 (6)
**Panel B2**				
**Marker**	**Antibody clone**	**Source**	**Dilution**	**Detection Dye (cycle)**
CD80	EPR1157(2)	Abcam	1:500	Opal 520 (1)
INOS	13F5.1	Millipore	1:2500	Opal 540 (2)
CD68	PG-M1	Dako	0.15ug/mL	Opal 570 (3)
CD20	E7B7T	CST	0.055ug/mL	Opal 620 (4)
PDL1	73-10	Abcam	0.18ug/mL	Opal 650 (5)
panCK	AE1/AE3	DAKO	0.665 ug/mL	Opal 690 (6)

Supplementary Table 13. Cell Population Definitions Used in Multiplex Imaging Analysis.

Cell population	Marker expression
B cells	CD68-, CD20+
Macrophages	CD68+
iNOS+ macrophages	CD68+, iNOS+
CD80+ macrophages	CD68+, CD80+
iNOS+CD80+ macrophages	CD68+, iNOS+, CD80+
PD-L1+ macrophages	CD68+, PD-L1+
NK cells	CD3-, CD56+
T cells	CD3+
CD8 T cells	CD3+, CD8+
CD4 T cells	CD3+, CD8-
Ki-67- CD4 T cells	CD3+, CD8-, FoxP3-, Ki-67-
T regulatory (Treg) cells	CD3+, CD8-, FoxP3+
Ki-67+ Treg cells	CD3+, CD8-, FoxP3+, Ki-67+
Tumor cells	PanCK+
PD-L1+ tumor cells	PanCK+, PD-L1+

**Supplementary Table 14. Packages Used in R Analysis.**	
**Package Name**	**Version**
ggplot2	3.3.5
wick	1.1
survminer	0.4.9
dplyr	1.0.7
plyr	1.8.6
Reshape2	1.4.4
Data.table	1.14.0
survival	3.2-13
tidyr	1.1.4
tidyverse	1.3.1
ggpubr	0.4.0
limma	3.48.3
readxl	1.3.1
msigdbr	7.4.1
stringr	1.4.0
venn	1.10
mixOmics	6.16.3
pheatmap	1.0.12
readr	2.0.1

FINAL CLINICAL STUDY PROTOCOL

Protocol Number: PICI0002

Protocol Title: Open-label, Multicenter, Phase 1b/2 Clinical Study to Evaluate the Safety and Efficacy of CD40 Agonistic Monoclonal Antibody (APX005M) Administered Together with Gemcitabine and nab-Paclitaxel with or without PD-1 Blocking Antibody (Nivolumab) in Patients with Previously Untreated Metastatic Pancreatic Adenocarcinoma

**IND Number:**	132683
**Name of Products:**	APX005M (experimental)<br>Nivolumab (experimental)<br>Gemcitabine (standard of care)<br>nab-Paclitaxel (standard of care)
**Phase of Development:**	1b/2
**Indication:**	Previously untreated metastatic pancreatic cancer
**Sponsor:**	Parker Institute for Cancer Immunotherapy<br>1 Letterman Drive<br>Suite D3500<br>San Francisco, CA 94129<br>Tel: 415-610-5466<br>Fax: 415-872-5305
**Collaborator**	Cancer Research Institute<br>One Exchange Plaza<br>55 Broadway, Suite1802<br>New York, NY 10006
**Protocol Date:**	Final Protocol: March 13, 2017<br>Amendment 1: April 14, 2017<br>Amendment 2: May 15, 2017<br>Amendment 3: September 29, 2017<br>Amendment 4: November 30, 2017<br>Amendment 5: March 22, 2018<br>Amendment 6: September 26, 2018<br>Amendment 7: October 11, 2019

-CONFIDENTIAL-

This document and its contents are the property of and confidential to Parker Institute for Cancer Immunotherapy. Any unauthorized copying or use of this document is prohibited.

SPONSOR APPROVAL PAGE

_______________________________ Date: 11 OCT 2019

Ramy Ibrahim, MD
Chief Medical Officer

INVESTIGATOR PROTOCOL AGREEMENT PAGE

I agree:

- To assume responsibility for the proper conduct of the study at this site.
- To conduct the study in compliance with this protocol, any future amendments, and with any other study conduct procedures provided by Parker Institute for Cancer Immunotherapy.
- Not to implement any changes to the protocol without written agreement from Parker Institute for Cancer Immunotherapy and prior review and written approval from the Institutional Review Board (IRB) or Independent Ethics Committee (IEC) except where necessary to eliminate an immediate hazard to patients.
- That I am thoroughly familiar with the appropriate use of the study drug(s), as described in this protocol and any other information provided by Parker Institute for Cancer Immunotherapy including, but not limited to, the current Investigator's Brochure (IB).
- That I am aware of, and will comply with, Good Clinical Practices (GCP) and all applicable regulatory requirements.
- To ensure that all persons assisting me with the study are adequately informed about the study drugs, the Parker Institute for Cancer Immunotherapy study protocol, and of their study-related duties and functions as described in the protocol.

Signature: ____ Date: ___________________

Name (print): ____
Principal Investigator

Site Number: ___________

1 SYNOPSIS

**Title of Study:**	Open-label, Multicenter, Phase 1b/2 Clinical Study to Evaluate the Safety and Efficacy of CD40 Agonistic Monoclonal Antibody (APX005M) Administered Together with Gemcitabine and nab-Paclitaxel with or without PD-1 Blocking Antibody (Nivolumab) in Patients with Previously Untreated Metastatic Pancreatic Adenocarcinoma
**Protocol Number:**	PICI0002
**Phase of Development:**	1b/2
**Objectives:**	**Phase 1b:**<br>Primary:<br>1. To determine the feasibility, safety and dose-limiting toxicities (DLT) of each treatment cohort.<br>2. To determine the recommended Phase 2 dose (RP2D) of APX005M when combined with nab-paclitaxel (NP)/gemcitabine (Gem).<br>3. To determine the RP2D of APX005M when combined with nivolumab/NP/Gem.<br>Secondary:<br>1. To determine objective response (OR) and duration of response (DOR) of each treatment cohort.<br>Exploratory:<br>1. To assess the pharmacokinetics (PK) of APX005M in Cycles 1 to 4.<br>2. To assess immune pharmacodynamic effects of each treatment cohort, in both blood and tumor tissue.<br>**Phase 2:**<br>Primary:<br>1. To estimate overall survival (OS) of each treatment arm.<br>2. To compare 1-year OS rate of each treatment arm to the historical rate for NP/Gem.<br>Secondary:<br>1. To determine the objective response rate (ORR), disease control rate (DCR), DOR, and progression-free survival (PFS) of each treatment arm.<br>2. To further characterize the feasibility and safety of each treatment arm.<br>Exploratory:<br>1. To assess the PK of APX005M in Cycles 1 to 4 (Arms B and C).<br>2. To assess immune pharmacodynamic effects of each treatment arm, in both blood and tumor tissue.<br>3. To assess associations between immune biomarkers and clinical outcomes.<br>4. To evaluate baseline and on-treatment microbiome profiles.<br>5. To construct multivariable linear models to dissect the pharmacodynamic effects of APX005M and nivolumab on immune biomarkers.

**Study Design:**	This is a multicenter, open-label, Phase 1b/2 study to evaluate the immunotherapy agents APX005M and nivolumab, in combination with Gem and NP in patients with previously untreated metastatic pancreatic adenocarcinoma.<br><br>**Phase 1b**<br>In the Phase 1b portion of the study, the following 4 treatment cohorts will be evaluated for feasibility and safety:<br>B1: NP/Gem/APX005M 0.1 mg/kg<br>B2: NP/Gem/APX005M 0.3 mg/kg<br>C1: Nivolumab/NP/Gem/APX005M 0.1 mg/kg<br>C2: Nivolumab/NP/Gem/APX005M 0.3 mg/kg<br>Enrollment in Cohorts B2 and C1 may occur concurrently. Enrollment in Cohort C2 may begin once enrollment in Cohort C1 has been completed.<br>Each cohort in the Phase 1b portion of the study will include approximately 6 DLT-evaluable patients. A cohort corresponding to Arm A of Phase 2 (nivolumab/NP/Gem) will not be tested, since an external study is being conducted to confirm the safety of nivolumab in combination with NP/Gem.<br>DLT is defined as any Grade 3 or higher toxicity that is treatment-related but not related to the natural progression of the tumor and occurs during the DLT observation period.<br><br>**Phase 2 (Randomized)**<br>Patients will be randomized to one of three arms: Arm A1, Arm B2, or Arm C2 (shown below).<br><u>Treatment arms:</u><br>A1: Nivolumab/NP/Gem<br>B2: NP/Gem/APX005M 0.3 mg/kg<br>C2: Nivolumab/NP/Gem/APX005M 0.3 mg/kg<br><br>A total of approximately 93 patients will be randomized/enrolled in Phase 2 (35 Arm A1, 29 Arm B2, 29 Arm C2). Twelve DLT-evaluable patients from the Phase 1b study, enrolled at the RP2D of APX005M in Arm C (i.e., 6 patients on B2 and 6 patients on C2) will be included in the Phase 2 analysis. Thus, each arm will enroll 35 patients, for a total of approximately105 patients. In the first step of randomization, 12 patients will be randomized in a 4:1:1 allocation to achieve balance in the total number of patients in each arm (since Arm A1 did not enroll patients in Phase 1b, more patients have to be allocated to Arm A1). Once the 12 patients are randomized, step 2 will randomize the remaining 81 patients in a 1:1:1 allocation.

**Selection of Patients:**	Main Inclusion Criteria:<br>1. Patient has histologically or cytologically documented diagnosis of pancreatic adenocarcinoma with metastatic disease. Locally advanced patients are not eligible.<br>2. Patient must have measurable disease by Response Evaluation Criteria in Solid Tumors (RECIST) v1.1.<br>3. Patients must be age 18 years or older.<br>4. Patients must have an Eastern Cooperative Oncology Group (ECOG) performance status of 0 or 1.<br>5. A baseline tumor tissue sample is mandatory for enrollment. If archival tumor tissue is not available, then a fresh tumor biopsy must be provided.<br>6. Patients must have the following laboratory values at Screening, without transfusions or growth factors, within 2 weeks of the first dose of investigational agents:<br>♦ Absolute neutrophil count (ANC) ≥ 1.5 x 10^9/L (in absence of growth factor support)<br>♦ Platelet count ≥ 150 x 10^9/L<br>♦ Hemoglobin ≥ 9 g/dL(without transfusion support)<br>♦ Serum creatinine ≤ 1.5 mg/dL, and creatinine clearance ≥ 50 ml/min as measured by Cockcroft and Gault formula<br>♦ Aspartate aminotransferase (AST) and alanine aminotransferase (ALT) ≤ 2.5 x institution's ULN for patients with no concurrent liver metastases, OR ≤ 5.0 x institution's ULN for patients with concurrent liver metastases<br>♦ Total bilirubin ≤ 1.5 x ULN, except in patients with documented Gilbert's Syndrome who must have a total bilirubin ≤ 3 x ULN<br>7. Women of childbearing potential (WOCBP) must have a negative pregnancy test (serum or urine) within the 7 days prior to study drug administration, and a negative urine pregnancy test within the 3 days before the first study drug administration, or a negative serum pregnancy test within 24 hours before the first study drug administration.<br>8. WOCBP and male patients who are sexually active with WOCBP must agree before receiving the first dose of study drugs to use 2 highly effective methods of contraception (including a physical barrier) during the study and for 5 months for women and 7 months for men following the last dose of study drug, as described in the body of the protocol.<br>9. Patients must have the ability to understand and willingness to sign a written informed consent document.<br>Main Exclusion Criteria:<br>1. Patient must not have received any prior treatment, including chemotherapy, for metastatic pancreatic adenocarcinoma, with the following exceptions and notes:<br>a. Patients who have received prior adjuvant or neoadjuvant therapy for pancreatic adenocarcinoma are eligible if the last dose of adjuvant therapy was more than 4 months before the date of study entry. In this case, prior Gem and/or NP are allowable.<br>b. Prior resection surgery is allowable.<br>c. Patients initially diagnosed with locally advanced pancreatic cancer who have undergone chemotherapy then resection and were with no evidence of disease are eligible if metastatic relapse of disease has occurred and if the last dose of chemotherapy was more than 4 months before the date of study entry.

	2. Patients must not have another active invasive malignancy, with the following exceptions and notes:<br>a. History of a non-invasive malignancy, such as cervical cancer in situ, non-melanomatous carcinoma of the skin, in situ melanoma, or ductal carcinoma in situ of the breast, is allowed.<br>b. History of malignancy that is in complete remission after treatment with curative intent is allowed.<br>c. No current or history of a hematologic malignancy is allowed, including patients who have undergone a bone marrow transplant.<br>3. History of clinically significant sensitivity or allergy to monoclonal antibodies, their excipients or intravenous gamma globulin<br>4. Previous exposure to CD40, PD-1, PD-L1, CTLA-4 antibodies or any other immunomodulatory agent<br>5. History of (non-infectious) pneumonitis that required corticosteroids or current pneumonitis, or history of interstitial lung disease<br>6. Patients must not have a known or suspected history of an autoimmune disorder, including but not limited to inflammatory bowel disease, celiac disease, Wegner syndrome, Hashimoto syndrome, systemic lupus erythematosus, scleroderma, sarcoidosis, or autoimmune hepatitis, within 3 years of the first dose of investigational agent, except for the following:<br>a. Patients with Type 1 diabetes mellitus, hypothyroidism only requiring hormone replacement, skin disorders such as vitiligo, or alopecia not requiring systemic therapy, or conditions not expected to recur in the absence of an external trigger are eligible.<br>b. Patients with a history of Hashimoto syndrome within 3 years of the first dose of investigational agent, which resolved to hypothyroidism alone.<br>7. Patients must not have an uncontrolled intercurrent illness, including an ongoing or active infection, current pneumonitis, symptomatic congestive heart failure (New York Heart Association class III or IV), unstable angina, uncontrolled hypertension, cardiac arrhythmia, interstitial lung disease, active coagulopathy, or uncontrolled diabetes.<br>8. Patients must not have a history of myocardial infarction within 6 months or a history of arterial thromboembolic event within 3 months of the first dose of investigational agent.<br>9. Patients must not have a history of human immunodeficiency virus, hepatitis B (HB), or hepatitis C, except for the following:<br>a. Patients with anti-HB core antibody but with undetectable HB virus deoxyribonucleic acid (DNA) and negative for HB surface antigen<br>b. Patients with resolved or treated hepatitis C virus (HCV) (i.e. HCV antibody positive but undetectable HCV RNA)<br>10. Patients must not have a history of primary immunodeficiency.<br>11. Patients must not receive concurrent or prior use of an immunosuppressive agent within 14 days of the first dose of investigational agent, with the following exceptions and notes:<br>a. Systemic steroids at physiologic doses (equivalent to dose of 10 mg oral prednisone) are permitted. Steroids as anti-emetics for chemotherapy are not allowed.<br>b. Intranasal, inhaled, topical, intra-articular, and ocular corticosteroids with minimal systemic absorption are permitted.

	c. Patients with a condition with anticipated use of systemic steroids above the equivalent of 10 mg prednisone are excluded.<br>d. Transient courses of steroids may be approved by the Medical Monitor on a case by case basis, dependent on dose and reason.<br>12. Patients must not have a history of clinically manifested central nervous system (CNS) metastases.<br>a. Patients with known or suspected leptomeningeal disease or cord compression are not eligible.<br>13. Patients must not have had major surgery as determined by the PI within 4 weeks before the first dose of study drug.<br>14. Patients must not have received another investigational agent within the shorter of 4 weeks or 5 half-lives before the first dose of investigational agent.<br>15. Patients must not have received a live attenuated vaccine within 28 days before the first dose of investigational agent, and patients, if enrolled, should not receive live vaccines during the study or for 180 days after the last dose of investigational agent.<br>16. Females who are pregnant or lactating or who intend to become pregnant during participation in the study are not eligible to participate.<br>17. Patients who have any clinically significant psychiatric, social, or medical condition that, in the opinion of the investigator, could increase the patient's risk, interfere with protocol adherence, or affect the patient's ability to give informed consent are ineligible to participate in the study.
**Planned Sample Size:**	Up to 24 DLT-evaluable patients will be enrolled in the Phase 1b portion of the study. A total of approximately 93 patients will be randomized/enrolled in Phase 2. Thus, the total sample size is expected to be approximately 117 patients.
**Investigational Therapy:**	**Phase 1b:**<br>APX005M (0.1 or 0.3 mg/kg) in combination with NP (125 mg/m^2) and Gem (1000 mg/m^2), all administrated intravenously (IV)<br>**OR**<br>APX005M (0.1 or 0.3 mg/kg) in combination with nivolumab (240 mg), NP (125 mg/m^2) and Gem (1000 mg/m^2), all administered IV<br><br>**Phase 2:**<br>Nivolumab (240 mg) in combination with NP (125 mg/m^2) and Gem (1000 mg/m^2), all administered IV<br>**OR**<br>APX005M (0.3 mg/kg) in combination with NP (125 mg/m^2) and Gem (1000 mg/m^2), all administrated IV<br>**OR**<br>APX005M (0.3 mg/kg) in combination with nivolumab (240 mg), NP (125 mg/m^2) and Gem (1000 mg/m^2), all administered IV

**Treatment Duration:**	Assuming all 4 cohorts are tested, upon completion of enrollment to Phase 1b, 1 additional month of follow-up will occur before declaring the RP2D of APX005M. Then the Phase 2 portion of the study will have 12 additional months of follow-up. Target enrollment completion is within 24 months; however, enrollment will proceed until met or as determined by the study sponsor (Parker Institute for Cancer Immunotherapy [PICI]). Considering several months for data management and statistical analysis, the total duration of the study is likely to be 5 years.<br>Patients will undergo screening and, if eligible, will undergo treatment in the assigned arm of the study until unacceptable toxicity, progression of disease, or withdrawal of consent. All patients will be followed for survival status until death or a maximum of 5 years.
**Study Endpoints:**	**Phase 1b:**<br>Primary:<br>• The frequency of DLT<br>• The RP2D of APX005M when combined with NP/Gem or nivolumab/NP/Gem<br>• The incidence of treatment-emergent AEs (TEAEs), serious adverse events (SAEs), and adverse events (AEs) causing discontinuation<br>Secondary:<br>• OR is determined by RECIST v1.1<br>• DOR is defined as the time from first documentation of response (complete response [CR] or partial response [PR]) to first documentation of progressive disease (PD)<br>Exploratory:<br>• PK of APX005M will be determined in Cycles 1 to 4.<br>• Immune pharmacodynamic endpoints may include, but are not limited to, the following:<br>o Changes in the tumor microenvironment (including cellularity, stromal content, cellular infiltration, and tumor apoptosis) may be assessed by tumor multiplex immunohistochemistry or other appropriate technology. Pharmacodynamic and PK parameters, if available, may be used to influence the RP2D.<br>o Gene expression may be determined by tumor RNA sequencing, peripheral blood RNA sequencing, or other appropriate technology. Other sequencing technologies, such as ATAC sequencing, may be performed.<br>o Tumor genomics may be determined when possible by Clinical Laboratory Improvement Amendment-certified mutational panel assessments and/or by whole exome sequencing.<br>o For variant calling and human leukocyte antigen (HLA) determination, normal tissue whole exome sequencing may be performed. In some cases, data regarding germ-line BRCA1/2 mutations or microsatellite instability may be incorporated into analyses.<br>o Cytokine and/or circulating factor analysis may be determined by a multiplex assay or other appropriate technology.<br>o Flow cytometry or other related technologies, such as CyTof analysis of peripheral blood, may be used to assess phenotype, function, and other changes in immune cellular subsets.<br>o Other markers to measure tumor burden, including circulating tumor DNA, tumor cells, and protein markers, may be measured in an exploratory fashion if material is available

	**Phase 2:**<br>Primary:<br>• OS is defined as the time from initiation of study therapy to date of death due to any cause or date of most recent patient contact. Patients who have not died are censored on their most recent contact date.<br>• 1-year OS rate in each treatment arm.<br>Secondary:<br>• Investigators' assessment of OR is determined by RECIST v1.1 and the ORR is defined as the proportion of patients who achieve a CR or PR.<br>• DCR is defined as the proportion of patients who achieve a CR or PR or SD.<br>• DOR is defined as the time from first documentation of response (CR or PR) to first documentation of PD.<br>• PFS is defined as the time from initiation of study therapy to date of first documented progression of disease, date of death due to any cause or date of most recent patient contact which documented progression-free status (i.e., clinic visit date or scan date). Patients who have not progressed or died are censored on their most recent progression-free date.<br>• The incidence of AEs defined as unacceptable toxicities in Phase 2, TEAEs, SAEs, and AEs causing treatment discontinuation<br>• Clinical laboratory data and vital signs (descriptive statistics) and numbers of patients with values outside limits of the normal range at each time point.<br>Exploratory:<br>• The exploratory endpoints for Phase 2 are the same as those described above for Phase 1b with the addition of evaluation of baseline and on-treatment microbiome profiles with treatment outcomes.
**Statistical Methods and Planned Analyses:**	This is a multi-center, open-label Phase 1b/2 study of CD40 agonistic monoclonal antibody, APX005M, and/or PD-1 blocking antibody, nivolumab, in combination with NP and Gem and for patients with newly diagnosed metastatic pancreatic cancer. The primary objectives of the Phase 1b study are to determine the feasibility, safety and DLT of each treatment cohort and to determine the RP2D of APX005M in combination with NP/Gem and with nivolumab/NP/Gem. The primary objective of the randomized Phase 2 study is to evaluate OS in three treatment arms: nivolumab/NP/Gem, NP/Gem/APX005M and nivolumab/NP/Gem/APX005M by comparing the 1-year OS rate with the historical value for NP/Gem.<br>**The safety population** consists of all patients who received at least 1 dose of any study drug. This is the population for the primary analyses of safety.<br>**The DLT-evaluable population** consists of patients who received 2 or 3 doses of NP/Gem and 1 dose of APX005M during Cycle 1, thus have completed the DLT observation period (ie, from the time of first administration of study drugs until prior to Cycle 2 Day 1) Patients who do not meet these criteria will be replaced in Phase 1b only, to assist with DLT and RP2D decision-making.<br>**The efficacy population** consists of (1) all patients who were randomized/enrolled in Phase 2 and received at least 1 dose of any study drug and (2) the 12 DLT-evaluable patients (6 on Arm B and 6 on Arm C) who were enrolled in Phase 1b at the RP2D. The efficacy population is the population for the primary analyses of efficacy.<br>**Phase 1b Design:** Four treatment cohorts will be evaluated for feasibility and safety. Cohorts B1 and B2 will escalate the dose of APX005M when combined with NP/Gem, and then Cohorts C1 and C2 will escalate the dose of APX005M

	when combined with nivolumab/NP/Gem. Enrollment in Cohorts B2 and C1 may occur concurrently. Enrollment in Cohort C2 may begin once enrollment in Cohort C1 has been completed. Approximately 6 DLT-evaluable patients will be enrolled in each cohort. A1 (nivolumab/NP/Gem) will not be tested in Phase 1b, since an external study is being conducted to confirm the safety of nivolumab in combination with NP/Gem.<br>Statistical analyses will include the following:<br>• The number of patients treated in each cohort will be reported, and reasons why any patient is not DLT evaluable will be summarized.<br>• Approximately 6 DLT-evaluable patients will be fully analyzed in each treatment cohort.<br>• Feasibility issues will be described for each treatment cohort.<br>• Toxicities will be graded by NCI-CTCAE, causality attributed, and tabulated by treatment cohort.<br>• RP2D of APX005M when combined with NP/Gem and with nivolumab/NP/Gem will be determined.<br>• RECIST OR will be scored and tabulated along with DOR, by treatment cohort.<br>• PK of APX005M.<br>• Immune pharmacodynamic effects will be measured, including change from baseline, and reported by treatment cohort.<br><br>**Phase 2 Design:** Once the RP2D of APX005M has been defined, the randomized Phase 2 portion of the study will commence. Patients will be randomized to one of 3 arms, defined by the addition of one or more immunotherapy agents to standard of care NP/Gem. The arms will be either A1 vs B2 vs C2 or A1 vs B1 vs C1. Note that the APX005M dose must be the same in Arms B and C, regardless of whether a higher APX005M dose was determined to be safe in Arm B. For each regimen, efficacy will be evaluated by comparing the 1-year overall survival (OS) rate to the historical value for NP/Gem.<br>Statistical analyses will include the following:<br>• Thirty-five patients will be analyzed on each treatment arm. Twelve DLT-evaluable patients from Phase 1b (Arms B and C) and 93 patients from Phase 2 will comprise the population for the final analysis of efficacy.<br>• OS will be estimated by the Kaplan-Meier method for each treatment arm.<br>• The 1-year OS rate and 1-sided 95% confidence interval (CI) will be calculated for each treatment arm, to determine whether the lower bound of the CI excludes the historical value for NP/Gem. A 1-sided one-sample Z test will also be conducted. The goal is to compare the survival probability at 1-year to the historical value of 0.35.<br>• ORR and DCR and their 95% CIs will be calculated for each treatment arm.<br>• PFS will be estimated by the Kaplan-Meier method for each treatment arm.<br>• DOR will be calculated from dates of first documented response and progression of disease.<br>• Toxicities will be graded by CTCAE v4.03 and tabulated by treatment arm.<br>• PK of APX005M in Cycles 1 to 4 (Arms B and C).<br>• Immune pharmacodynamic effects may be measured, including but not limited to change from baseline, and reported by treatment arm.<br>• Test of associations between immune biomarkers and clinical outcomes

	• Construct multivariable linear models to dissect the pharmacodynamic effects of APX005M and nivolumab on immune biomarkers.

2 TABLE OF CONTENTS

1 SYNOPSIS ... 4
2 TABLE OF CONTENTS ... 13
IN-TEXT TABLES ... 19
IN-TEXT FIGURES ... 20
3 LIST OF ABBREVIATIONS ... 21
4 INTRODUCTION ... 25
4.1 Background on Metastatic Pancreatic Cancer ... 25
4.2 Background on APX005M ... 27
4.2.1 Pharmacology ... 27
4.2.2 Pharmacokinetics ... 28
4.2.3 Clinical Experience ... 28
4.2.4 Summary of the Known and Potential Risks and Benefits ... 29
4.2.5 Dose Rationale ... 30
4.3 Background on Nivolumab ... 30
4.3.1 Clinical Studies of Nivolumab ... 30
4.4 Background on Gemcitabine and nab-Paclitaxel ... 32
4.5 Rationale ... 33
5 STUDY OBJECTIVES AND ENDPOINTS ... 35
5.1 Study Objectives ... 35
5.1.1 Phase 1b ... 35
5.1.1.1 Primary Objectives: ... 35
5.1.1.2 Secondary Objectives: ... 35
5.1.1.3 Exploratory Objectives: ... 35
5.1.2 Phase 2 ... 35
5.1.2.1 Primary Objectives: ... 35
5.1.2.2 Secondary Objectives: ... 35
5.1.2.3 Exploratory Objectives: ... 35
5.2 Study Endpoints ... 36
5.2.1 Phase 1b ... 36
5.2.1.1 Primary Endpoints ... 36
5.2.1.2 Secondary Endpoints ... 36
5.2.1.3 Exploratory Endpoints ... 36
5.2.2 Phase 2 ... 37

5.2.2.1 Primary Endpoints 37
5.2.2.2 Secondary Endpoints 37
5.2.2.3 Exploratory Endpoints 37

6 INVESTIGATIONAL PLAN 38

6.1 Description of Overall Study Design and Plan 38

6.1.1 Duration of Study Participation 40

6.1.2 Total Number of Patients 41

6.1.3 Early Termination Rules for Unacceptable Toxicity in Phase 2....................41

6.1.4 Treatment Beyond Unequivocal Disease Progression 41

7 SELECTION AND WITHDRAWAL OF PATIENTS 42

7.1 Inclusion Criteria 42

7.1.1 Childbearing Potential and Highly Effective Methods of Contraception 43

7.2 Exclusion Criteria 44

7.3 Discontinuation, Withdrawal, and Replacement of Patients 46

7.3.1 Discontinuation of Study Drug 46

7.3.2 Withdrawal/Discontinuation of Patients 47

7.3.3 Replacement of Patients 47

7.4 Follow-Up for Drug Discontinuation/Patient Withdrawal from Study 47

7.5 Lost to Follow-Up 48

7.6 Study Termination 48

8 TREATMENTS 49

8.1 Details of Study Treatments 50

8.1.1 APX005M 50

8.1.2 Nivolumab 51

8.1.3 Nab-Paclitaxel 52

8.1.4 Gemcitabine 54

8.2 Preparation and Administration of Study Treatment 57

8.2.1 APX005M 57

8.2.2 Nivolumab 57

8.2.3 Nab-paclitaxel 57

8.2.4 Gemcitabine 58

8.3 Monitoring Following APX005M Administration 58

8.4 Dosage Schedule 58

8.5 Management of Study Drug-Related Toxicities 59
8.5.1 Day 1 60
8.5.2 Day 3 62
8.5.3 Day 8 64
8.5.4 Day 15 66
8.5.5 Criteria for Treatment Continuation within a Cycle 69
8.5.6 APX005M, NP, and Gem Dose Modifications 70
8.6 Study Treatment Assignment 70
8.6.1 Phase 1b 70
8.6.2 Phase 2 70
8.7 Blinding 71
8.8 Treatment Accountability and Compliance 71
8.9 Prior and Concomitant Illnesses and Medications 71
8.9.1 Prior and Concomitant Illnesses 71
8.9.2 Prior and Concomitant Medications 71
8.9.3 Prohibited Medications 72
9 STUDY PROCEDURES 73
10 EFFICACY ASSESSMENTS 83
10.1 Response Criteria 84
10.1.1 Definitions of Measurable and Non-Measurable Disease 84
10.1.2 Guidelines for Evaluation of Measurable Disease 84
10.1.3 Measurement of Effect 85
10.1.3.1 Target Lesions 85
10.1.3.2 Non-Target Lesions 85
10.1.3.3 Response Criteria 85
10.1.3.4 Evaluation of target lesions 85
10.1.3.5 Evaluation of non-target lesions 86
10.1.3.6 Overall Objective Status 86
11 PHARMACOKINETICS AND PHARMACODYNAMICS 87
11.1 Pharmacokinetic Sampling 87
11.1.1 Blood Samples 87
11.1.2 Analytical Methodology 87
11.2 Immune Endpoints 87
11.2.1 Immune Biomarkers 88

11.2.3 Anti-drug antibodies 89
12 SAFETY ASSESSMENTS 90
12.1 Vital Signs 90
12.2 Physical Examination 90
12.3 Electrocardiogram 90
12.4 Laboratory Assessments 90
12.5 Adverse Events, Adverse Events of Special Interest, and Serious Adverse Events 91
12.5.1 Time Period and Frequency for Collecting Adverse Events, Serious Adverse Events, and Other Reportable Safety Event Information 91
12.5.2 Definition of Adverse Events 92
12.5.2.1 Definition of Serious Adverse Events 92
12.5.2.2 Definition of Adverse Events of Special Interest 93
12.5.3 Recording Adverse Events 93
12.5.4 Disease-related Events and/or Disease-related Outcomes Not Qualifying as Adverse Events or Serious Adverse Events 95
12.5.5 Serious Adverse Event Reporting 95
12.5.6 Regulatory Reporting Requirements for Serious Adverse Events 96
12.5.6.1 Sponsor Reporting of Serious Adverse Events 96
12.5.7 Pregnancy 96
12.5.8 Overdose 97
13 STATISTICAL ANALYSIS 98
13.1 Determination of Sample size 102
13.1.1 Phase 1b 102
13.1.2 Phase 2 102
13.2 Analysis Populations 103
13.3 Demographic and Baseline Characteristics 103
13.4 Efficacy Analysis 103
13.4.1 Primary Efficacy Endpoint 103
13.4.2 Secondary Efficacy Endpoints 104
13.4.3 Exploratory Endpoints 104
13.5 Pharmacokinetic Analysis 105
13.6 Safety Endpoints 105

13.6.1 Phase 1b: Assessing Feasibility, Dose-Limiting Toxicity, and Recommended Dose for Phase 2 105
13.6.2 Analysis of Adverse Events 105
13.7 Interim Analysis 106
13.8 Data Monitoring 106
13.8.1 Data Review Team (DRT) 106
14 STUDY MANAGEMENT 107
14.1 Approval and Consent 107
14.1.1 Regulatory Guidelines 107
14.1.2 Institutional Review Board/Independent Ethics Committee 107
14.1.3 Informed Consent 107
14.2 Data Handling 107
14.3 Source Documents 108
14.4 Record Retention 108
14.5 Monitoring 108
14.6 Quality Control and Quality Assurance 108
14.7 Protocol Amendments and Protocol Deviations 109
14.7.1 Protocol Amendments 109
14.7.2 Protocol Deviations 109
14.8 Ethical Considerations 109
14.9 Financing and Insurance 109
14.10 Publication Policy / Disclosure of Data 109
15 REFERENCES 110
16 APPENDICES 114
16.1 ECOG Performance Scale 114
16.2 Nivolumab Toxicity Management Guide 115
16.3 Guidance for the Management of Infusion Reaction/Cytokine Release Syndrome 123
16.4 Document Revision History 124
16.4.1 Key Revisions in Amendment 1 124
16.4.2 Key Revisions in Amendment 2 124
16.4.3 Key Revisions in Amendment 3 125
16.4.4 Key Revisions in Amendment 4 127

16.4.5 Key Revisions in Amendment 5 130
16.4.6 Key Revisions in Amendment 6 132
16.4.7 Key Revisions in Amendment 7 135

IN-TEXT TABLES

Table 1 Highly Effective Methods of Contraception 44
Table 2 Study Cohorts/Arms 49
Table 3 Treatment Regimens and Schedules 58
Table 4 Management of Study Treatment-Related Toxicities on Day 1 60
Table 5 Management of Study Treatment-Related Toxicities on Day 3 63
Table 6 Management of Study Treatment-Related Toxicities on Day 8 64
Table 7 Management of Study Treatment-Related Toxicities on Day 15 66
Table 8 APX005M, NP, and Gem Dose Modifications 70
Table 9 Phase 1b Treatment Assignment 70
Table 10 Phase 2 Design 71
Table 11 Schedule of Assessments (For Arms Including APX005M, Phase Ib) 74
Table 12 Schedule of Assessments (For Arms Including APX005M, Phase 2) 77
Table 13 Schedule of Assessments (For Arms Not Including APX005M, Phase 2) 80
Table 14 Laboratory Assessments 91
Table 15 Classification of Adverse Events by Intensity 94
Table 16 Classification of Adverse Events by Relationship to Study Drug 94
Table 17 Phase 1b Statistical Design 99
Table 18 Phase 2 Statistical Design 100
Table 19 Bayesian Termination Rules 101
Table 20 ECOG Performance Scale 114
Table 21 Infusion-related Reaction/Cytokine Release Syndrome Management Recommendations 123

IN-TEXT FIGURES

Figure 1 Study Flow Chart 38

3 LIST OF ABBREVIATIONS

Abbreviation	Definition
ADA	Anti-drug antibody(ies)
ADCC	Antibody-dependent cell-mediated cytotoxicity
AE	Adverse event
AESI	Adverse event(s) of special interest
ALT (SGPT)	Alanine aminotransferase (serum glutamic pyruvic transaminase)
ANC	Absolute neutrophil count
APC	Antigen-presenting cell
AST (SGOT)	Aspartate aminotransferase (serum glutamic oxaloacetic transaminase)
AUC	Area under the plasma concentration-time curve
BLOQ	Below the limit of quantitation
BUN	Blood urea nitrogen
CD	Cluster of differentiation
C_{avgss}	Average concentration at steady state
C_{min1}	Minimum concentration at 1 hour
C_{minss}	Minimum concentration at steady state
C_{max}	Maximum serum concentration
C_{maxss}	Maximum concentration at steady state
CNS	Central nervous system
CO_2	Carbon dioxide
CR	Complete response
CRF	Case Report Form
CT	Computed tomography
CTCAE	Common Terminology for Cancer Adverse Events
DC	Dendritic cell
dCTP	Deoxycytidine triphosphate
DILI	Drug-induced liver injury
DLT	Dose-limiting toxicity
DNA	Deoxyribonucleic acid
DOR	Duration of response

DRT	Data Review Team
eCRF	Electronic Case Report Form
ECG	Electrocardiogram
ECOG	Eastern Cooperative Oncology Group
EDC	Electronic data capture
EOI	End of infusion
EOT	End of treatment
Fc	Fragment crystallizable region
FDA	Food and Drug Administration
FDG-PET/CT	Positive emission tomography/computed tomography using fluorodeoxyglucose
FT3	Free Triiodothyronine
FT4	Free thyroxine
GCP	Good Clinical Practice
Gem	Gemcitabine
GGT	Gamma-glutamyl transpeptidase
GLP	Good Laboratory Practices
HCG	Human chorionic gonadotropin
HB	Hepatitis B
HCV	Hepatitis C virus
HEENT	Head, eye, ear, nose and throat examination
HIPPA	Health Insurance Portability Accountability Act
HLA	Human leukocyte antigen
HSR	Hypersensitivity reaction
HUS	Hemolytic uremic syndrome
ICH	International Council for Harmonisation
IEC	Independent Ethics Committee
IgG	Immunoglobulin G
i.p.	Intraperitoneally
IRB	Institutional Review Board
IV	Intravenous
IWRS	Interactive Web Response System
LDH	Lactate dehydrogenase
LFT	Liver function test
MCH	Mean corpuscular hemoglobin

MCHC	Mean corpuscular hemoglobin concentration
MCV	Mean corpuscular volume
MRI	Magnetic resonance imaging
MTD	Maximum tolerated dose
NCI	National Cancer Institute
NK	Natural killer
NOAEL	No observed adverse effect
NP	Nab-paclitaxel/Abraxane
NSCLC	Non-small-cell lung cancer
OR	Objective response
ORR	Objective response rate
OS	Overall survival
PBMC	Peripheral blood mononuclear cells
PC	Pancreatic cancer
PD	Progressive disease or pharmacodynamic
PDA	Pancreatic ductal adenocarcinoma
PET	Positron emission tomography
PFS	Progression-free survival
PI	Principal Investigator
PK	Pharmacokinetic
PR	Partial response
RBC	Red blood cell
RCC	Renal cell carcinoma
RECIST	Response Evaluation Criteria in Solid Tumors
RNA	Ribonucleic acid
RP2D	Recommended Phase 2 Dose
SAE	Serious adverse event
SAERF	Serious Adverse Event Report Form
SD	Stable disease
T3	Triiodothyronine
TEAE	Treatment-emergent adverse event
TME	Tumor immune microenvironment
TNFR	Tumor necrosis factor receptor
TSH	Thyroid-Stimulating Hormone
ULN	Upper limit of normal

WBC	White blood cell
WES	Whole exome sequencing
WOCB	Women of childbearing potential
US	United States
USP	United States Pharmacopeia

4 INTRODUCTION

4.1 Background on Metastatic Pancreatic Cancer

Pancreatic cancer (PC) is one of the most lethal malignancies of the gastrointestinal tract, with a 5-year survival of about 8%. In the United States (US) an estimated total of 53,070 PCs will occur in 2016, with 41,780 estimated deaths among these patients. In 2016, PC surpassed breast cancer to become the third leading cause of cancer death in the US and is projected to become the second leading cause of cancer death by 2030.[1] There are several potential reasons for the increased mortality associated with pancreatic adenocarcinoma. First, the majority of patients are diagnosed with pancreatic adenocarcinoma at an advanced stage, when the patient has unresectable disease and is therefore incurable. Even in the 15% to 20% of patients with resectable, and therefore potentially curable, disease at diagnosis, only about 10% to 15% of those patients are alive at 5 years with surgery alone. This only improves to 20% to 25% with gemcitabine (Gem) and/or concurrent chemoradiation adjuvant therapy.[2-4] The low survival associated with resectable disease is thought to be due to microscopic metastases emerging early in the course of PC development. Furthermore, PC tends to be relatively resistant to chemotherapy, which accounts for the modest benefit of adjuvant therapy as noted above. Even with the development of more aggressive and effective regimens – namely FOLFIRINOX and Gem and nab-paclitaxel (NP) – the median overall survival (OS) is still less than a year for patients with metastatic pancreatic adenocarcinoma.[5,6] Continued advancements in treatment of pancreatic adenocarcinoma are imperative.

Immunotherapy

Among the promising approaches to activating therapeutic antitumor immunity is the modulation of host immune system. Immune modulation includes inhibitory or stimulatory pathways in the immune system that are crucial for activating the immune response, maintaining self-tolerance, and modulating the duration and amplitude of physiological immune responses. Modulation of immune checkpoints by antibodies against immune inhibitory molecules has shown clinical benefits for patients with malignancies such as melanoma.[7,8] Currently, antagonistic antibodies against immune inhibitory molecules such as cytotoxic T-lymphocyte-associated protein 4 (CTLA-4) and programmed cell death-1 (PD-1)/ programmed cell death ligand-1 (PD-L1) and agonistic antibodies against immune costimulatory molecules such as cluster of differentiation (CD)40 are under active development for different cancer indications. To date, the anti-CTLA4 antibody ipilimumab is US Food and Drug Administration (FDA) approved for use in metastatic melanoma both as a single agent or in combination with PD-1 inhibitors (nivolumab). The PD-1 inhibitors nivolumab and pembrolizumab are now approved in various diseases.

Immunotherapy in Pancreatic Cancer

While checkpoint inhibitors have been effective in melanoma and lung cancer, clinical benefit of CTLA-4 and PD-1 inhibitors as single agents in the management in PC patients has not been noted.[9,10] Current immunotherapy studies utilize a combination of traditional anti-cancer therapy with checkpoint inhibitors, vaccines, or other immunotherapy agents as single or combination therapies, such as CD40 monoclonal antibodies (mAbs).[11]

In this study, we are evaluating the combination of a CD40 agonist, APX005M, with a PD-1 inhibitor, nivolumab, and standard chemotherapy for pancreatic adenocarcinoma, Gem and NP.

CD40 Monoclonal Antibodies

The cell surface molecule CD40, a member of the tumor necrosis factor receptor (TNFR) superfamily, plays an important role in induction of tumor apoptosis and regulation of immune activation, especially in crosstalk between T cells and antigen presenting cells (APCs).[12] CD40 is expressed by dendritic cells (DCs), B cells, monocytes, and some non-lymphoid cells.[13] The natural ligand (CD40L) for CD40 is CD154, which is expressed on activated T cells and provides a major component of T cell "help" for immune response. Agonistic CD40 antibodies can substitute for the function of CD154 on T cells to boost immunity.

Signaling through CD40 on APCs, including DCs, monocytes, and B cells, can, in turn, enhance the T cell response via improvement in antigen processing and presentation, and through the release of cytokines from activated APCs.[14,15] Therefore, an agonistic CD40 antibody can activate and stimulate both innate and adaptive immunity.

CD40 is also expressed on many tumor cells and can mediate a direct cytotoxic effect. In addition to B cell lymphoma, CD40 expression has been reported in 30% to 70% of primary human solid tumor samples, including melanoma and carcinomas[16] and 25% of PCs.[17] Activation of CD40 on tumor cells results in tumor cell apoptosis and inhibition of tumor growth.[18] Due to its action on both immune and tumor cells, CD40 has been studied as a target for novel cancer immunotherapy; agonistic anti-CD40 antibodies have been demonstrated to be potent stimulators of tumor immune responses in both animal models and cancer patients.[19-22]

The potential mechanisms of action for an agonistic anti-CD40 antibody, depending on its isotype, include stimulation of immune response by activating antigen processing and presentation, recruitment of immune effectors such as natural killer (NK) cells and macrophages, and direct cytotoxic effects on tumor cells. Thus, the desired therapeutic CD40 agonist antibody should have these functionalities.

A few CD40 agonistic antibodies have been evaluated in human clinical studies. Objective responses (ORs) have been observed with nearly every CD40 antibody formulation used as single agents across a variety of diseases, including PC. Toxicities have been transient and manageable. The majority of the clinical studies in cancer patients with solid tumors have been conducted with the fully human immunoglobulin G (IgG)2 CD40 antibody CP-870,893. In a Phase 1 clinical study, CP-870,893 was well tolerated; the maximum tolerated dose (MTD) was found to be 0.2 mg/kg. The main toxicity of CP-870,893 was cytokine release syndrome of mild to moderate severity. Antitumor activity was observed in several melanoma patients treated with CP-870,893.[23,24]

One study indicated that CP-870,893 can also mediate an antitumor effect when combined with chemotherapy in patients with metastatic PC.[25] Other CD40 agonistic antibodies that have been studied in human clinical studies are SGN-40 and ChiLob 7/4. SGN-40 is an IgG1 humanized anti-CD40 antibody that is a weak CD40 agonist, which has been tested predominantly in hematological malignancies as monotherapy or in combination with rituximab and chemotherapy.[26,27] The major adverse effects of SGN-40 were anemia, pleural effusion, and thrombocytopenia.[26]

SEA-CD40 is an engineered version of SGN-40. SEA-CD40 and SGN-40 have the same amino acid sequences, but the fragment crystallizable (Fc) region of SEA-CD40 is defucosylated, leading to increased binding to and cross-linking by FcγRIIIa.

ChiLob 7/4 is an IgG1 chimeric CD40 agonistic antibody that has been tested in a Phase 1 clinical study in patients with solid tumors. The MTD of ChiLob 7/4 is 200mg/weekly × 4, and the major dose-limiting toxicity (DLT) was reversible liver enzyme elevation.[28] Although CP-870,893 has potent CD40 agonistic activities, it is an IgG2 antibody, and thus lacks antibody effector functions that constitute an important mechanism of action for CD40 antibody-mediated antitumor activities.[24] Due to its chimeric structure, ChiLob 7/4 might be immunogenic, especially considering that the immune response is boosted by its CD40 agonistic effects.

ADC-1013 is a fully human IgG1 CD40-agonistic antibody in Phase 1 clinical trial for treatment of solid tumors.[29] ADC-1013 is intended for intra-tumoral delivery.

SGN-40 is an IgG1 antibody, but a weak CD40 agonist. Due to its chimeric structure, ChiLob 7/4 may potentially be more immunogenic considering that the immune response may be boosted by its CD40-agonistic effects. ADC-1013 has similar binding affinity as APX005M but is a weaker CD40-agonistic antibody likely due to its lack of binding to FcγRIIb. Although SEA-CD40 has increased potency compared with SGN-40, its high-affinity binding to FcγRIIIa may lead to enhanced antibody effector functions such as antibody-dependent cell-mediated cytotoxicity (ADCC) on CD40-expressing cells such as DCs and B cells.

An IgG1 humanized antibody that can utilize Fc receptors to cluster CD40 and enhance CD40-agonistic effects, like APX005M, might be preferable both in terms of potential decreased immunogenicity and increased anti-tumor activity for use in cancer immunotherapy.

4.2 Background on APX005M

4.2.1 Pharmacology

APX005M is an IgG1 humanized mAb with the S267E mutation at the Fc region. APX005M binds with high affinity to human CD40 (Kd = 1.2 × 10-10 M) and monkey CD40 (Kd = 3.5 × 10−10 M), but does not cross-react with mouse or rat CD40. APX005M blocks the binding of CD40 to CD40L. The APX005M binding epitope has been mapped to 2 specific regions on CD40. These are 92TSEACESCVLHRSCSP107 and 125PCPVGFFSNVSSAFEKCHPW144. The region 92TSEACESCVLHRSCSP107 is known as a CD40L-binding domain. It has been shown that CD40L-blocking antibodies tend to have more potent CD40 agonistic activities than CD40L-non-blocking antibodies.[31]

Preclinical experiments with APX005M showed that it activates the CD40 signaling pathway, leading to APC activation, as demonstrated by an increased expression of CD80, CD83, and CD86 and by expression and release of cytokines from human DCs and lymphocytes. As a result of APC activation, APX005M enhances T-cell proliferation to alloantigen, triggers production of interferon-gamma (IFN-γ) in response to viral antigens, and enhances T-cell response to tumor antigens. APX005M combined with a toll-like receptor 4 (TLR4) agonist or an antibody against PD-L1 synergistically enhances T-cell responses. In comparison with other CD40-agonistic antibodies, such as CP-870,893, SGN-40, and ADC-1013 analogs, APX005M is the most potent CD40 agonist. APX005M did not appear to have a substantive effect on

normal human DC and T-cell counts, but could partially reduce B-cell counts in vitro. The potential for APX005M to induce expression of cytokines was evaluated with peripheral blood mononuclear cells (PBMC) obtained from normal humans and treatment naïve cynomolgus monkeys, including anti-CD3 antibody as a positive control. Cytokine secretion differed significantly between species with much less secretion from monkey PBMCs compared with human PBMCs. These data suggest that APX005M is a strong CD40-agonistic antibody that can activate APCs (DCs, B cells, and monocytes) and in turn stimulate T-cell response.

The direct cytotoxicity effect and the antibody effector functions such as antibody-dependent cellular phagocytosis (ADCP) of APX005M were determined in CD40 positive human lymphoma xenograft models in mice. In human lymphoma Ramos models, APX005M was capable of inhibiting tumor growth in a dose-dependent manner, and eradicated established tumors at 3 mg/kg and 10 mg/kg. A significant anti-tumor effect was also observed in the rituximab-resistant Namalwa model [APX005M IB]. These data suggest that APX005M, as a single agent, can induce potent growth inhibition of CD40-expressing human tumors.

Preliminary human data show that APX005M induces a dose-dependent activation of APCs (as demonstrated by increases in expression of activation markers such as CD54, CD70, CD80, CD86, human leukocyte antigen [HLA]-DR), T cell activation and increases in circulating levels of interleukin (IL)12, IFN-γ, TNF-α and IL6.

4.2.2 Pharmacokinetics

Nonclinical pharmacokinetics (PK) of APX005M were determined in a Good Laboratory Practice (GLP) repeat-dose toxicology study using cynomolgus monkeys. Weekly intravenous (IV) administration of 5 doses of APX005M was well tolerated at 0.3, 3, and 30 mg/kg. The PK properties of APX005M are typical of other mAbs and comprise low clearance (average range of 0.401–7.27 mL/h/kg), small volume of distribution (average range of 57–80.1 mL/kg), and long terminal half-life (average > 66 hours at 3 mg/kg and 30 mg/kg). Positive anti-drug antibodies (ADA) titers were observed in all animals in the low-dose group (0.3 mg/kg) but not in the high-dose group (30 mg/kg) [IB]. Based on these results, the no observed adverse effect level (NOAEL) was considered 30 mg/kg.

There are limited human PK data with APX005M at this time. In the first in human study of APX005M, exposures to APX005M at dose levels of 0.03 mg/kg or less were for the most part below the limit of quantitation (BLOQ). IV administration of APX005M at doses between 0.1 and 1 mg/kg led to rapid increase in serum concentrations, reaching a maximum just after the end of the infusion. Levels declined rapidly thereafter and were for the most part BLOQ between 24 and 168 hours after the start of dosing. Increases in the dose of APX005M (0.1 mg/kg to 1 mg/kg) led to approximately dose-proportional increases in maximum serum concentration (C_{max}) and area under the curve at the last measurable time point ($AUC_{0–t}$). No accumulation of APX005M was observed with every 21 days dosing.

4.2.3 Clinical Experience

Study APX005M-001 is a first-in-human Phase 1 dose-escalation study of APX005M with 8 pre-planned dose levels. APX005M was administered to study subjects at doses up to 1 mg/kg. At the 1 mg/kg dose level, 1 out of 6 DLT-evaluable subjects experienced a DLT (Grade 4 cytokine release syndrome). Two additional subjects at the 1 mg/kg dose level experienced serious adverse events (SAE) in later cycles (Grade 3 cytokine release syndrome

and Grade 4 thrombocytopenia). On May 2, 2016 Apexigen decided to discontinue dose escalation and enroll up to 6 subjects in dose level 0.6 mg/kg (originally designed as an intermediate de-escalation dose level) and an additional 3 subjects at the previously completed dose level 0.3 mg/kg to better characterize the safety and pharmacodynamics of APX005M and to help establish the single agent recommended Phase 2 dose (RP2D).

As of 08 November 2016, 30 subjects have received APX005M with a median exposure of 3 cycles and had 321 adverse events (AE):

- 89% of AEs were ≤ Grade 2.
- 9% AEs were Grade 3.
- 1.5% of AEs were Grade 4.
- The majority of AEs were considered unrelated to APX005M by the Investigator.

APX005M demonstrated a dose-dependent activation of APCs (as demonstrated by increases in expression of activation markers such as CD54, CD70, CD80, CD86, HLA-DR), T-cell activation and increases in circulating levels of IL12, IFN-γ, TNF-α and IL6.

For further details on the APX005M-001 study, please refer to latest version of the APX005M Investigator's Brochure (IB).

4.2.4 Summary of the Known and Potential Risks and Benefits

Symptoms associated with cytokine release syndrome (including but not limited to flushing, itchiness, chills, fever, rash, tachycardia, hypotension, hypertension, rigor, and myalgia) after administration of APX005M are possible and have been observed in some of the patients receiving APX005M. Guidance for monitoring and management of cytokine release syndrome are included in Section 16.3 of this protocol and in the APX005M IB.

Transient transaminase elevations (≤ Grade 2) have been observed in several patients with liver metastases, which were not associated with a particular dose of APX005M. Six patients with liver metastases enrolled in the study experienced a transient increase in total bilirubin. Liver function test abnormalities tend to resolve to baseline within 7 days from APX005M administration.

Transient decreases in peripheral blood lymphocyte count in general and B-cell count in particular have been observed for APX005M as well as for other CD40-agonistic mAbs, and are believed to be a pharmacodynamic effect. Transient decreases in platelet counts were observed for some of the patients receiving higher doses of APX005M but were not associated with bleeding or other clinical manifestations.

Other symptoms might also occur, including allergic reactions, which could be severe, pulmonary edema, and rarely, thromboembolic events, myocardial infarction and/or death.

In the ongoing Phase 1 study APX005M-001, APX005M demonstrated a dose-dependent activation of APCs, T cell activation and increases in circulating levels of cytokines.

The biological effects and the overall tolerability of APX005M up to 1 mg/kg suggest a best in class profile for APX005M and the possibility of a safe and tolerable combination with other immunomodulatory antibodies such as nivolumab.

4.2.5 Dose Rationale

APX005M has been administered in the APX005M-001 study to patients with solid tumors as a single agent at 7 dose levels starting at 0.1 mg/kg up to 1.0 mg/kg every 21 days. At 0.6 and 1 mg/kg dose levels 1 out of 6 patients experienced a DLT. The RP2D for APX005M as a single agent every 21 days is 0.3 mg/kg.

The proposed doses of APX005M are 0.1 mg/kg (10 times lower than the highest dose of APX005M administered to human patients) and 0.3 mg/kg (approximately 3.3 times lower than the highest dose of APX005M administered to human patients).

At all dose levels proposed, APX005M has been well tolerated; all adverse reactions have been moderate (≤ Grade 2), transient and easily managed in outpatient setting. All available data suggests that 0.1 and 0.3 mg/kg every 21 days is a safe and pharmacodynamically active dose of APX005M.

4.3 Background on Nivolumab

Nivolumab is a fully human monoclonal immunoglobulin G4 that targets the PD-1 blocking antibody. PD-1, also known as CD279, is a cell surface membrane receptor predominantly expressed on activated T and B lymphocytes and memory T lymphocytes and, when bound to its ligands PD-L1 or programmed cell death ligand-2 (PD-L2), negatively regulates the immune system. While PDL-1 and PDL-2 are commonly expressed on DCs and other APCs, a variety of different tumor cells can also express these ligands.32

4.3.1 Clinical Studies of Nivolumab

Nivolumab is a human IgG4 mAb has specific FDA-approved indications in multiple diseases: metastatic melanoma, non-small-cell lung cancer (NSCLC), renal cell carcinoma (RCC), squamous cell carcinoma of the head and neck, and Hodgkin’s disease. Single-agent nivolumab has been studied33 at doses of 0.1 to 10 mg/kg every 2 weeks (Q2W) in 296 subjects with melanoma (n = 104), NSCLC (n = 122), castration-resistant prostate cancer (n = 17), RCC (n = 34), or colorectal carcinoma (n = 19). Drug-related Grade 3 or 4 AEs occurred in 14% of subjects, and there were 3 deaths from pulmonary toxicity. No MTD was defined at the doses tested. A relative dose intensity of ≥ 90% was achieved in 86% of subjects. Fifteen of 296 subjects (5%) discontinued due to treatment-related AEs. The most common AEs, irrespective of causality, were fatigue, anorexia, diarrhea, nausea, cough, dyspnea, constipation, vomiting, rash, pyrexia, and headache. Common treatment-related AEs included fatigue, rash, diarrhea, pruritus, anorexia and nausea. The most common (≥ 5%) Grade ≥ 3 AEs included fatigue (5%). Treatment-related SAEs occurred in 32 of 296 subjects (11%) and > 1% included pneumonitis (2%). Drug-related events of special interest included pneumonitis, vitiligo, colitis, hepatitis, hypophysitis, uveitis, and thyroiditis. The spectrum, frequency and severity of treatment-related AEs were similar across dose levels tested.

Antitumor activity was observed at all doses tested. ORs were observed NSCLC, melanoma, and RCC and in sites of metastasis including liver, lung, lymph nodes and bone. In subjects with NSCLC, ORs were observed at doses of 1.0, 3.0, or 10.0 mg/kg with rates of 6%, 32%, and 18% respectively. Responses were observed in 6/18 subjects with squamous tumors (33%), in 7/56 (12%) of those with non-squamous histology, and in 1 of 2 (50%) of those with unknown histology. Stable disease (SD) lasting ≥ 24 weeks was observed in 7 subjects (7%)

with lung cancer. In melanoma, 26 ORs were observed at doses ranging from 0.1 to 10.0 mg/kg. At a dose of 3.0 mg/kg ORs were observed in 7 of 17 subjects (41%). SD lasting ≥ 24 weeks was observed in 6 subjects (6%). In RCC, ORs were seen in 4 of 17 subjects (24%) treated with 1.0 mg/kg and in 5 of 16 subjects (31%) treated with 10.0 mg/kg. SD lasting ≥ 24 weeks was observed in 9 subjects (27%). No responses were observed in subjects with colorectal or prostate cancer.

A Phase 1 study has investigated nivolumab in combination with platinum-based doublet chemotherapy in chemotherapy-naïve NSCLC.34 Subjects with stage IIIB/IV NSCLC were randomized by histology to nivolumab (10 mg/kg Day 1/21)/Gem (1250 mg/m^2 Days 1, 8/21)/cisplatin (75 mg/m^2 Day 1/21), nivolumab (10 mg/kg Day 1/21)/pemetrexed (500 mg/m^2 Day 1/21)/cisplatin (75 mg/m^2 Day 1/21) or nivolumab (10 mg/kg Day 1/21)/carboplatin (AUC 6 Day 1/21)/paclitaxel (200 mg/m^2 Day 1/21). Nivolumab doses were started at 10 mg/kg every 3 weeks until progressive disease (PD); platinum doublet chemotherapy was given for a maximum of 4 cycles. Across the treatment arms of the study, no DLTs were seen at the 10 mg/kg nivolumab dose in combination with the platinum doublets. Tumor responses ranged from 33% to 47%, with carboplatin/paclitaxel in combination with nivolumab 10 mg/kg, demonstrating a 47% objective response rate (ORR). An additional arm assessed carboplatin/paclitaxel with 5 mg/kg nivolumab, which showed similar ORR of 43%, suggesting a lack of a correlation of dose with response at these doses tested. Across the arms, responses were durable, with an estimated median duration of 24 to 85 weeks. Median progression-free survival (PFS) in the carboplatin/paclitaxel arm in combination with 5 and 10 mg/kg nivolumab was 31 and 21 weeks, respectively; and 1-year overall survival (OS) rate was 86% and 60%. The most common treatment-related Grade 3 or 4 AEs were pneumonitis (4 subjects, 7%), fatigue (3 subjects, 5%) and acute renal failure (3 subjects, 5%).34

A Phase 1 study is exploring nivolumab in combination with taxanes chemotherapy in PC, NSCLC, and metastatic breast cancer. The interim results from the PC and NSCLC cohorts were published recently.35-36

The primary objective of Part 1 is to evaluate DLTs. Subjects treated with ≥ 2 cycles of nivolumab with chemotherapy and remained on study for 14 calendar days or who discontinued due to DLT prior to completing 2 cycles of nivolumab were considered DLT evaluable. If deemed safe, treatment arms will be expanded in Part 2 to further assess safety, tolerability, and antitumor activity. In Arm A Part 1, subjects with advanced PC and 1 prior chemotherapy regimen received NP 125 mg/m^2 on Days 1, 8, and 15 and nivolumab 3 mg/kg on Days 1 and 15 of a 28-day cycle.35-36

If Arm A is safe, a cohort of CT-naive subjects will be enrolled in Arm B and treated with NP and Gem 1000 mg/m once weekly for 3 weeks of every 4 weeks + nivolumab. In Arm C, treatment-naive subjects with stage IIIB/IV NSCLC received 4 cycles of NP 100 mg/m^2 on Days 1, 8, and 15 and carboplatin AUC 6 on Day 1 and nivolumab 5 mg/kg on Day 15 of a 21-day cycle. If Arm C is safe, subjects in Arm D will receive the Arm C regimen, except nivolumab will start at Cycle 3. In both NSCLC arms, nivolumab monotherapy begins at Cycle 5.35-36

As of June 28, 2016, 11 and 6 subjects were treated in Arms A and B in Part 1, respectively. No DLTs were reported in Arm A, and 1 in Arm B (nonimmune hepatitis, suspected to be due to Gem; resolved and subject continued nivolumab + NP without Gem). The most common

Grade 3 or 4 treatment-emergent AEs were pulmonary embolism, neutropenia, and anemia in 2 of 11 subjects (18%) in Arm A and anemia in 2 of 6 subjects (33%) in Arm B. Nine subjects discontinued due to PD (8 in Arm A, 1 in Arm B). Of the 9 response-evaluable subjects in Arm A, 2 had a partial response (PR), 4 had a SD and 3 had PD. Of the 6 response-evaluable subjects in Arm B, 3 had a PR and 3 had a SD.[36]

It was concluded that adding nivolumab to NP ± Gem is feasible for subjects with advanced PC, and antitumor activity of this regimen appears to be encouraging.[36]

Dose Rationale for Nivolumab

The safety and efficacy of a 240 mg Q2W flat dose of nivolumab is expected to be similar to the 3 mg/kg Q2W dosing regimen. Per the nivolumab IB, a flat dose of nivolumab 240 mg Q2W was selected since it is identical to a dose of 3 mg/kg for subjects weighing 80 kg, the observed median body weight in nivolumab treated cancer subjects. Using a population PK model, the overall distributions of nivolumab exposures (average concentration at steady state [C_{avgss}], minimum concentration at steady state [C_{minss}], C_{maxss}, and minimum concentration at 1 hour [C_{min1}]) are comparable after treatment with either 3 mg/kg or 240 mg nivolumab. The predicted range of nivolumab exposures (median and 90% prediction intervals) resulting from a 240 mg flat dose across the 35 to 160 kg weight range is maintained well below the corresponding exposures observed with the well tolerated 10 mg/kg nivolumab Q2W dosage.

In a clinical study, a dose association was observed for infusion site reactions and hypersensitivity reactions (1.7% at 0.3 mg/kg, 3.7% at 2 mg/kg and 18.5% at 10 mg/kg). All the events were Grade 1 to 2 and were manageable. An infusion duration of 30 minutes for 3 mg/kg nivolumab (30% of the dose provided at 10 mg/kg) is not expected to present any safety concerns compared to the prior experience at 10 mg/kg nivolumab dose infused over a 60-minute duration. The safety of nivolumab 3 mg/kg administered as a 30-minute infusion was assessed in CA209153 in subjects (n = 322) with previously treated advanced NSCLC. Overall, there were no clinically meaningful differences in the frequency of hypersensitivity/infusion-related reactions (of any cause or treatment-related) in subjects administered nivolumab over a 30-minute infusion compared with that reported for subjects with the 60-minute infusion. Thus, it was shown that nivolumab can be safely infused over 30 minutes.

4.4 Background on Gemcitabine and nab-Paclitaxel

In 1996, Gem was approved for the management of metastatic pancreatic adenocarcinoma due to an improvement in median OS over 5-fluorouracil (5-FU; 5.65 versus 4.41 months).[37] Gem monotherapy was the mainstay of treatment for over 10 years, before FOLFIRINOX demonstrated an improved 11.1-month median OS compared with 6.8 months with Gem monotherapy.[5] Though there was a marked improvement in OS, FOLFIRINOX was also associated with significant AEs, including Grade 3 or 4 neutropenia (45.7%), neutropenic fever (5.4%), fatigue (23.6%), vomiting (14.5%), diarrhea (12.7%), and neuropathy (9%).

In 2013, Gem and NP was approved for use in subjects with metastatic pancreatic adenocarcinoma, with studies demonstrating an 8.5-month median OS compared with 5.7 months with Gem monotherapy. The most common Grade 3 or greater AEs associated with Gem and NP included neutropenia (38%), fatigue (17%), neutropenic fever (3%), and neuropathy (17%).[6] Though Gem plus NP has not been compared head-to-head with

FOLFIRINOX, the side effects from Gem and NP tend to be less frequent, and this result may better allow for testing additional chemotherapy combinations. Results from a randomized Phase 3 clinical study comparing NP plus Gem versus Gem alone in 861 first-line PC subjects showed subjects on NP plus Gem had a median survival of 8.5 months compared with 6.7 months for those who received Gem alone.[6] At the end of 1 year and 2 year, 35% and 9%, respectively, of those receiving the combination were alive, compared with 22% and 4% of those being treated with Gem only. Toxicities were modest and centered on reversible myelosuppression and peripheral neuropathy.

4.5 Rationale

Scientific Summary

Preclinical studies show that in mouse models of pancreatic carcinoma, the combination of Gem and NP with agonist CD40 mAb triggers an effective T cell response, for which clinical benefit can be safely extended with anti-PD-1. NP/Gem is poorly effective in this model as is either single agent CD40 or PD-1 mAb. Combination therapy, however, induces a robust T-cell response, marked infiltration of T cells into the tumor (not otherwise observed) and tumor response and increased survival.

Clinical Summary

Tumor regressions in patients with metastatic PC have been observed with NP/Gem, nivolumab/NP, nivolumab/NP/Gem, and Gem/CP870,893 (anti-CD40). The protocol now incorporates all four of these approaches by combining NP/Gem with nivolumab and APX005M (anti-CD40).

Currently, no clinical data are available for the combination of APX005M plus Gem and NP with or without nivolumab. This combination is going to be explored in this Phase 1b/2 study, which includes a Phase 1b dose-finding part and a Phase 2 efficacy evaluation part in patients with previously untreated metastatic pancreatic adenocarcinoma (see Section 6.1 for study design details). The Phase 1 data will define the RP2D of APX005M when combined with the standard dose of Gem and NP, with or without nivolumab.

Two doses for APX005M are being explored, based on data from its manufacturer, Apexigen, from findings of the first-in-human Phase 1 dose-escalation study. The doses of APX005M chosen for this study are 0.1 mg/kg (10 times lower than the highest dose of APX005M administered to human patients) and 0.3 mg/kg (the recommended single-agent dose; approximately 3.3 times lower than the highest dose of APX005M administered to human patients). Doses for nivolumab are based on prior experience of using this antibody in combination with Gem and NP. The doses of nivolumab, Gem, and NP selected for this study are per the FDA-approved US labeling.

The second part of the study is aimed to evaluate in an exploratory manner the activity of APX005M combined with Gem and NP, with or without nivolumab in metastatic pancreas cancer patients. A randomization process will be used to avoid the potential bias in patient selection and to balance risk factors between treatment arms.

Following FDA approval in 2013, the combination of Gem and NP has become a gold standard in the first-line setting for patient with metastatic PC; however, the prognosis remains poor.

This persistent unmet medical need in PC is directing researchers to explore new treatments, including immunological approaches.

Nivolumab, a PD-1-specific antibody, has been shown to produce long-term remissions with limited toxicity in patients with advanced melanoma, and also showed activity in NSCLC, RCC, and Hodgkin's lymphoma. So far, PC appeared refractory to single-agent checkpoint blockade alone.10

Preclinical data support the rationale of this clinical study. A recent study using a genetically engineered mouse model of pancreatic ductal adenocarcinoma (PDA), which like human PDA exhibits minimal spontaneous immunity, demonstrated that despite robust expression of PD-1 and PD-L1 in the tumor microenvironment, treatment with αPD-1 with or without αCTLA-4 failed to improve the survival of mice or slow the growth of PDA tumors. However, administration of αCD40, Gem, and NP, induces T-cell immunity in mice with PDA, controls tumor growth and significantly improves survival in a CD8+ T-cell-dependent manner. In particular, αCD40/NP/Gem plus αPD-1 nearly doubles the median OS in genetically engineered Pdx1-Cre (KPC) mice with pre-established spontaneous pancreatic tumors.38 Moreover, the capability of treated mice to reject second and third subcutaneous tumor challenges in a CD8+ T-cell-dependent fashion, thereby rendering long-term survival, suggests the establishment of antitumor immune memory with curative potential. These findings indicate that poorly immunogenic tumors, epitomized by the KPC pancreatic tumor model, can nevertheless be controlled by the adaptive immune system provided a dual approach of therapeutic T cell induction and checkpoint blockade is utilized.

Mechanistically, a preclinical study in the KPC mouse model in PDA showed the ability of a single dose of αCD40 to alter T cells in the tumor immune microenvironment (TME), expand clonal T-cell populations, and convert the TME in PC to a site replete with infiltrating T cells.[39] In combination with a novel chemotherapy doublet, αCD40 treatment bypasses innate immune sensors to generate functional APCs and T cells, culminating in durable responses with curative potential, even in a highly immunosuppressive TME.

In this study, we are evaluating the safety and efficacy of the combination of αCD40 agonist, APX005M, with a PD-1 inhibitor, nivolumab, and standard chemotherapy for pancreatic adenocarcinoma, Gem and NP.

5 STUDY OBJECTIVES AND ENDPOINTS

This study will be conducted in 2 phases, each with its own objectives and endpoints.

5.1 Study Objectives

5.1.1 Phase 1b

5.1.1.1 Primary Objectives:

1. To determine the feasibility, safety, and DLTs of each treatment cohort.
2. To determine the RP2D of APX005M when combined with NP/Gem.
3. To determine the RP2D of APX005M when combined with nivolumab/NP/Gem.

5.1.1.2 Secondary Objectives:

1. To determine OR and duration of response (DOR) of each treatment cohort.

5.1.1.3 Exploratory Objectives:

1. To assess the PK of APX005M in Cycles 1 to 4.
2. To assess immune pharmacodynamic effects of each treatment cohort, in both blood and tumor tissue.

5.1.2 Phase 2

5.1.2.1 Primary Objectives:

1. To estimate the OS of each treatment arm.
2. To compare 1-year OS rate of each treatment arm with the historical rate for NP/Gem.

5.1.2.2 Secondary Objectives:

1. To determine the ORR, disease control rate (DCR), DOR, and PFS of each treatment arm.
2. To further characterize the feasibility and safety of each treatment arm.

5.1.2.3 Exploratory Objectives:

1. To assess the PK of APX005M in Cycles 1 to 4 (Arms B and C).
2. To assess immune pharmacodynamic effects of each treatment arm, in both blood and tumor tissue.
3. To assess associations between immune biomarkers and clinical outcomes.
4. To evaluate baseline and on-treatment microbiome profiles.
5. To construct multivariable linear models to dissect the pharmacodynamic effects of APX005M and nivolumab on immune biomarkers.

5.2 Study Endpoints

5.2.1 Phase 1b

5.2.1.1 Primary Endpoints

- The frequency of DLT.
- The RP2D of APX005M when combined with NP/Gem or nivolumab/NP/Gem.
- The incidence of treatment-emergent AEs (TEAEs), SAEs, and AEs causing treatment discontinuation.

5.2.1.2 Secondary Endpoints

- OR is determined by Response Evaluation Criteria in Solid Tumors (RECIST) v1.1.
- DOR is defined as time from first documentation of response (complete response [CR] or PR) to first documentation of PD.

5.2.1.3 Exploratory Endpoints

- PK of APX005M will be determined in Cycles 1 to 4 (as described in Section 13.5).
- Immune pharmacodynamic endpoints may include, but are not limited to, the following:
    - Changes in the tumor microenvironment (including cellularity, stromal content, cellular infiltration, and tumor apoptosis) may be assessed by tumor multiplex immunohistochemistry or other appropriate technology. Pharmacodynamic and PK parameters, if available, may be used to influence the RP2D.
    - Gene expression may be determined by tumor ribonucleic acid (RNA) sequencing, peripheral blood RNA sequencing, or other appropriate technology. Other sequencing technologies, such as assay for transposase-accessible chromatin (ATAC) sequencing, may be performed.
    - Tumor genomics may be determined when possible by Clinical Laboratory Improvement Amendment-certified mutational panel assessments and/or by whole exome sequencing.
    - For variant calling and HLA determination, normal tissue whole exome sequencing may be performed. In some cases, data regarding germline BRCA1/2 mutations or microsatellite (MS) instability will be incorporated into analyses.
    - Cytokine and/or circulating factor analysis may be determined by a multiplex assay or other appropriate technology.
    - Flow cytometry or other related technologies, such as CyTof analysis of peripheral blood, may be used to assess phenotype, function, and other changes in immune cellular subsets.
    - Other markers to measure tumor burden, including circulating tumor deoxyribonucleic acid (DNA), tumor cells, and protein markers, may be measured in an exploratory fashion if material is available.

5.2.2 Phase 2

5.2.2.1 Primary Endpoints

- OS, defined as the time from initiation of study therapy to date of death due to any cause or date of most recent patient contact. Patients who have not died are censored on their most recent contact date.
- 1-year OS rate in each treatment arm.

5.2.2.2 Secondary Endpoints

- Investigators' assessment of OR is determined by RECIST v1.1 and ORR is defined as the proportion of patients who achieve a CR or PR.
- DCR is defined as the proportion of patients who achieve a CR or PR or SD.
- DOR is defined as the time from first documentation of response (CR or PR) to first documentation of PD.
- PFS is defined as the time from initiation of study therapy to date of first documented progression of disease, date of death due to any cause or date of most recent patient contact which documented progression-free status (i.e., clinic visit date or scan date). Patients who have not progressed or died are censored on their most recent progression-free date.
- The incidence of AEs defined as unacceptable toxicities (see Section 6.1), TEAEs, SAEs, and AEs causing treatment discontinuation.
- Clinical laboratory data and vital signs (descriptive statistics) and numbers of patients with values outside limits of the normal range at each time point.

5.2.2.3 Exploratory Endpoints

The exploratory endpoints for Phase 2 are the same as those described for Phase 1b (see Section 5.2.1.3) with the addition of evaluation of baseline and on-treatment microbiome profiles with treatment outcomes.

6 INVESTIGATIONAL PLAN

6.1 Description of Overall Study Design and Plan

This is a multi-center, open-label, Phase 1b/2 study to evaluate the immunotherapy agents APX005M and nivolumab in combination with Gem and NP in patients with previously untreated metastatic pancreatic adenocarcinoma.

Phase 1b will involve 4 treatment cohorts, and Phase 2 will involve randomization to 1 of 3 treatment arms, as shown in Figure 1.

Figure 1 Study Flow Chart

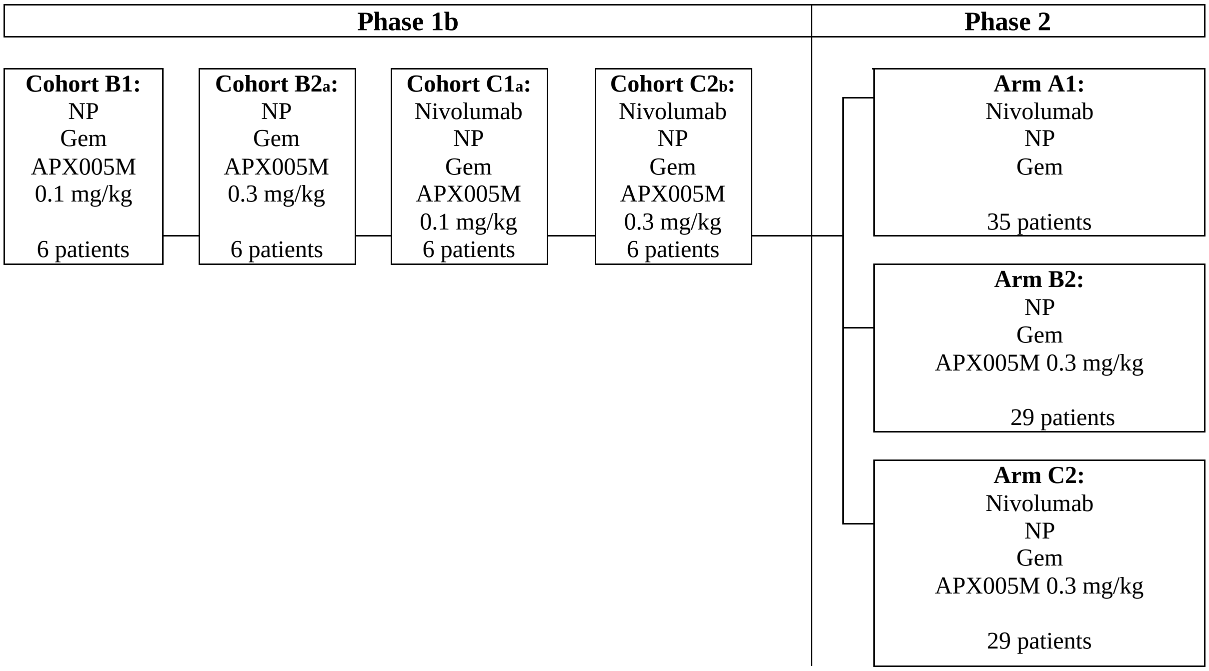

a Enrollment in Cohorts B2 and C1 may occur concurrently.

b Enrollment in Cohort C2 may begin once enrollment in Cohort C1 has been completed.

In both Phase 1b and Phase 2, participants will undergo the following tumor biopsy procedures to assess tumor and immune markers:

1. Prior to beginning study treatment (i.e., baseline biopsy, mandatory. Archival tissue is acceptable)
2. During treatment (i.e., on-treatment biopsy; mandatory, if medically feasible); Cycle 2 after second dose of APX005M, or after third dose Nivolumab for arms not including APX005M. Otherwise, any on-treatment biopsy will be accepted unless there is no lesion that can be safely biopsied
3. Additional biopsies may be performed for patients who have prolonged stable disease, defined as stable for more than two, consecutive disease assessments by RECIST v1.1, and/or if tumor shrinkage is demonstrated, followed by a new lesion and/or radiological disease progression
4. Ad hoc collections may be performed with Medical Monitor approval

In both Phase 1b and Phase 2, NP/Gem could be dosed according to one of the schedules below depending on whether or not the patient experiences toxicity:

1. 2 weeks on, 1 week off
2. 3 weeks on, 1 week off

Please note: Day 15 assessments are still required on the 2 weeks on, 1 week off schedule.

Phase 1b

In the Phase 1b portion of the study, 4 treatment cohorts will be evaluated for feasibility and safety (see Section 8.6.1). Enrollment in Cohorts B2 and C1 may occur concurrently. Enrollment in Cohort C2 may begin once enrollment in Cohort C1 has been completed. Each cohort of the study will include approximately 6 DLT-evaluable patients, defined as patients who received 2 or 3 doses of NP/Gem and 1 dose of APX005M during Cycle 1, thus have completed the DLT observation period (ie, from the time of first administration of investigational agents until prior to Cycle 2 Day 1). Patients who do not remain on study up to this time for reasons other than DLT will be replaced. DLTs will be graded according to the National Cancer Institute (NCI) Common Terminology Criteria for Adverse Events (CTCAE) version 4.03.

A DLT is defined as any Grade 3 or higher toxicity that is treatment-related but not related to the natural progression of the tumor and occurs during the DLT observation period. The following will be considered DLTs:

1. Grade 4 hematologic toxicity lasting ≥ 7 days
2. Grade 3 or 4 neutropenia with a single temperature of > 38.3ºC (101ºF) or a sustained temperature of ≥ 38ºC (100.4ºF) for more than one hour
3. Grade 4 thrombocytopenia (platelet count < 25,000 cells/mm^3) if associated with:
   a. A bleeding event which does not result in hemodynamic instability but requires an elective platelet transfusion, or
   b. A life-threatening bleeding event which results in urgent intervention and admission to an Intensive Care Unit
4. Grade 4 non-hematologic toxicity (not laboratory)
5. Grade 3 non-hematologic toxicity (not laboratory) lasting > 3 days despite optimal supportive care
6. Any Grade ≥ 3 non-hematologic laboratory value if:
   a. Medical intervention is required to treat the patient, or
   b. The abnormality leads to hospitalization, or
   c. The abnormality persists for > 1 week
7. Grade 3 AEs that compromise a major organ (e.g., congestive heart failure) regardless of duration
8. Grade 5 toxicity.

Randomized Phase 2

Once the RP2D of APX005M in combination with nivolumab in the Phase 1b portion of the study is determined, the randomized Phase 2 portion will commence. Patients will be randomized to Arm A1, Arm B2, or Arm C2, or to Arm A1, Arm B1, or Arm C1, if Cohort B2 and/or C2 are deemed unsafe in Phase 1b (see Section 8.6.2). Note that the APX005M dose must be the same in Arms B and C, regardless of whether a higher APX005M dose was determined to be safe in Arm B. For each regimen, efficacy will be evaluated by comparing the 1-year OS rate to the historical value for NP/Gem. A total of approximately 93 patients will be randomized/enrolled in Phase 2 (35 Arm A1, 29 Arm B2, 29 Arm C2).

Based on the safety and efficacy data from Phase 1b, Arm B2 and C2 were selected for Phase 2.

Randomization will be managed by the Parker Institute for Cancer Immunotherapy (PICI), using an interactive web response system (IWRS). Patients must receive the first dose of study drug (Cycle 1 Day 1) within 3 days of randomization.

An unacceptable toxicity is defined as any ≥ Grade 3 toxicity that is treatment-related but not related to the natural progression of the tumor and occurs during the Phase 2 period. The following will be considered unacceptable toxicities:

1. Grade 4 thrombocytopenia (platelet count < 25,000 cells/mm^3) if associated with:
    a. A life-threatening bleeding event which results in urgent intervention and admission to an Intensive Care Unit
2. Any ≥ Grade 3 non-hematologic laboratory value if:
    a. Medical intervention is required to treat the patient, and
    b. The abnormality leads to hospitalization, and
    c. The abnormality persists for > 1 week
3. Grade 3 or 4 AEs that compromise a major organ (e.g., congestive heart failure) regardless of duration
4. Grade 5 toxicity

6.1.1 Duration of Study Participation

The Phase 1b study will enroll for at least 9 months, assuming all 4 cohorts are tested, and have 1 additional month of follow-up before declaring the RP2D of APX005M. The Phase 2 portion of the study will enroll for approximately 18 months and have 12 additional months of follow-up. All patients will be followed for survival status until death or a maximum of 5 years. Considering several months for data management and statistical analysis, the total duration of the study is likely to be 5 years. In both Phase 1b and 2, enrollment will proceed until met or as determined by the study sponsor (Parker Institute for Cancer Immunotherapy).

Patients will undergo screening and, if eligible, will undergo treatment in the assigned arm of the study until unacceptable toxicity, progression of disease, or withdraw of consent as noted above. Treatment schedules per cohort/arm and phase of study are detailed in Section 8. All patients will be followed for survival status until death or a maximum of 5 years. Once a patient

is in follow-up, patients will be followed by a clinic visit or phone call approximately every 3 months as specified in Table 11, Table 12 and Table 13. In addition, ad hoc collection of survival and new anti-cancer therapy for active patients may be requested by the Sponsor.

6.1.2 Total Number of Patients

Up to 24 DLT-evaluable patients will be enrolled in Phase 1b. A total of approximately 93 patients will be randomized/enrolled in Phase 2. Thus, the total sample size is expected to be approximately 117 patients.

6.1.3 Early Termination Rules for Unacceptable Toxicity in Phase 2

A Bayesian rule will be employed to monitor toxicity during Phase 2 (as described in Section 13) to determine whether a study arm or the study needs to be terminated. A Medical and Statistical Supervision (MSS) process involving a Data Review Team (DRT) will be responsible for monitoring toxicity, as described in Section 13.8.1.

6.1.4 Treatment Beyond Unequivocal Disease Progression

Treatment decisions, particularly continuation versus discontinuation, will be made by the investigator on the basis of available clinical and imaging data at the site

For patients with radiological progression according to RECIST v1.1, in the absence of performance status decline and significant symptoms due to disease progression, the investigator may elect to keep the patient on treatment, and repeated assessment of disease status will be performed.

Patients must be informed of treatment beyond progression and this must be documented.

7 SELECTION AND WITHDRAWAL OF PATIENTS

Patients will be identified by referrals from physicians at participating cancer centers within the PICI, by outside physicians or health care providers, or by self-referral.

To enroll patients into Phase 1b cohorts, sites will have to contact PICI to request an assignment. During Phase 1b, dosing of the first 3 patients of each cohort will be staggered by at least one week. If at one week, and for each of the 3 patients, there are no ongoing symptoms of cytokine release syndrome related to the infusion, and if no DLT occurs, subsequent patients to the cohort may be dosed without restriction. A meeting of the DRT will be held prior to each dose escalation (i.e., between cohorts); ad hoc meetings may be held if a particular DLT requires immediate follow-up.

7.1 Inclusion Criteria

Patients must meet all of the following criteria at Screening and baseline to participate in the study:

1. Patient has histologically or cytologically documented diagnosis of pancreatic adenocarcinoma with metastatic disease. Locally advanced patients are not eligible.
2. Patient must have measurable disease by RECIST v1.1.
3. Patients must be age 18 years or older.
4. Patients must have an Eastern Cooperative Oncology Group (ECOG) performance status of 0 or 1 (see Appendix 16.1).
5. A baseline tumor tissue sample is mandatory for enrollment. If archival tumor tissue is not available, then a fresh tumor biopsy must be provided.
6. Patients must have the following laboratory values at Screening, without transfusions or growth factors, within 2 weeks of the first dose of investigational agents:
   - Absolute neutrophil count (ANC) ≥ 1.5 x 10^9/L (in absence of growth factor support)
   - Platelet count ≥ 150 x 10^9/L
   - Hemoglobin ≥ 9 g/dL(without transfusion support)
   - Serum creatinine ≤ 1.5 mg/dL, and creatinine clearance ≥ 50 mL/min as measured by Cockcroft and Gault formula
   - Aspartate aminotransferase (AST) and alanine aminotransferase (ALT) ≤ 2.5 x institution’s upper limit of normal (ULN) for patients with no concurrent liver metastases, OR ≤ 5.0 x institution’s ULN for patients with concurrent liver metastases
   - Total bilirubin ≤ 1.5 x ULN, except in patients with documented Gilbert’s syndrome, who must have a total bilirubin ≤ 3 x ULN
7. Women of childbearing potential (WOCBP; defined in Section 7.1.1) must have a negative pregnancy test (serum or urine) within the 7 days prior to study drug

administration, and within the 3 days before the first study drug administration, or a negative pregnancy test within the 24 hours before the first study drug administration.

8. WOCBP and male patients who are sexually active with WOCBP must agree to use 2 highly effective methods of contraception (including a physical barrier; see Section 7.1.1) before the first dose of study drugs, during the study, and for 5 months for women and 7 months for men following the last dose of study drug.
9. Patients must have the ability to understand and willingness to sign a written informed consent document.

7.1.1 Childbearing Potential and Highly Effective Methods of Contraception

A WOCBP is defined as any female who has experienced menarche and who has not undergone surgical sterilization (hysterectomy and/or bilateral oophorectomy) and is not postmenopausal. Menopause is defined as 12 months of amenorrhea in women over the age 45 years in the absence of other biological or physiological causes. In addition, females under the age of 55 years must have a serum follicle stimulating hormone level > 40 mIU/mL to confirm menopause.

WOCBP must have a negative pregnancy test before starting study treatment. Blood or urine pregnancy tests must have a minimum test sensitivity of at least 25 IU/L. Kits measuring either total human chorionic gonadotropin (hCG) or the beta (β) fraction are acceptable. Monthly pregnancy testing, either serum or urine (with a minimum sensitivity 5 IU/L or equivalent units of HCG), is required.

Females of childbearing potential who are sexually active with a nonsterilized male partner must use a highly effective method of contraception for 28 days prior to the first dose of investigational product, and must agree to continue using such precautions for 5 months after the final dose of investigational product; cessation of contraception after this point should be discussed with a responsible physician. Periodic abstinence, the rhythm method, and the withdrawal method are not acceptable methods of contraception. They must also refrain from egg cell donation for 6 months after the final dose of investigational product.

A highly effective method of contraception is defined as one that results in a low failure rate (i.e., less than 1% per year) when used consistently and correctly. The acceptable methods of contraception are described in Table 1.

Nonsterile males who are sexually active with a female partner of childbearing potential must use a highly effective method of contraception (see Table 1) from Day 1 through 7 months post last dose. For males adequate birth control methods is defined as double barrier contraception, i.e., condom + diaphragm, condom or diaphragm + spermicidal gel or foam. In addition, they must refrain from sperm donation for 7 months after the final dose of investigational product.

Table 1 Highly Effective Methods of Contraception

Barrier Methods	Hormonal Methods
Male condom with spermicide[a]	Implants
Male condom with diaphragm ± spermicide	Hormone shot or injection
Diaphragm with spermicide	Combined pill
Copper T intrauterine device	Minipill
Levonorgestrel-releasing intrauterine system (e.g., Mirena®)[b]	Patch

[a] If male condom without spermicide is used, another form of contraception is required to meet the definition of a highly effective method of contraception with a failure rate of less than 1%.

[b] This is also considered a hormonal method.

7.2 Exclusion Criteria

Patients meeting any of the following criteria are ineligible to participate in this study:

1. Patient must not have received any prior treatment, including chemotherapy, biological therapy, or targeted therapy for metastatic pancreatic adenocarcinoma, with the following exceptions and notes:
    a. Patients who have received prior adjuvant therapy for pancreatic adenocarcinoma are eligible if neoadjuvant and adjuvant therapy (including chemotherapy and/or radiotherapy) was fully completed more than 4 months before the start of study treatment. In this case, prior Gem and/or NP is allowable.
    b. Prior resection surgery is allowable.
    c. Patients initially diagnosed with locally advanced PC who have undergone chemotherapy then resection and were with no evidence of disease are eligible if metastatic relapse of disease has occurred and if the last dose of chemotherapy was more than 4 months before the date of study entry.
2. Patients must not have another active invasive malignancy, with the following exceptions and notes:
    a. History of a non-invasive malignancy, such as cervical cancer in situ, non-melanomatous carcinoma of the skin, in situ melanoma, or ductal carcinoma in situ of the breast, is allowed.
    b. History of malignancy that is in complete remission after treatment with curative intent is allowed.
    c. No current or history of a hematologic malignancy is allowed, including patients who have undergone a bone marrow transplant.
3. History of clinically significant sensitivity or allergy to mAbs, their excipients, or intravenous gamma globulin.

4. Previous exposure to CD40, PD-1, PD-L1, CTLA-4 antibodies or any other immunomodulatory agent.
5. History of (non-infectious) pneumonitis that required corticosteroids or current pneumonitis, or history of interstitial lung disease.
6. Patients must not have a known or suspected history of an autoimmune disorder, including but not limited to inflammatory bowel disease, celiac disease, Wegner syndrome, Hashimoto syndrome, systemic lupus erythematosus, scleroderma, sarcoidosis, or autoimmune hepatitis, within 3 years of the first dose of investigational agent, except for the following.
    a. Patients with Type 1 diabetes mellitus, hypothyroidism only requiring hormone replacement, skin disorders such as vitiligo, or alopecia not requiring systemic therapy, or conditions not expected to recur in the absence of an external trigger are eligible.
    b. Patients with a history of Hashimoto syndrome within 3 years of the first dose of investigational agent, which resolved to hypothyroidism alone.
7. Patients must not have an uncontrolled intercurrent illness, including an ongoing or active infection, current pneumonitis, symptomatic congestive heart failure (New York Heart Association class III or IV), unstable angina, uncontrolled hypertension, cardiac arrhythmia, interstitial lung disease, active coagulopathy, or uncontrolled diabetes.
8. Patients must not have a history of myocardial infarction within 6 months or a history of arterial thromboembolic event within 3 months of the first dose of investigational agent.
9. Patients must not have a history of human immunodeficiency virus, hepatitis B virus (HBV), or hepatitis C virus (HCV), except for the following:
    a. Patients with anti-hepatitis B core antibody but with undetectable HBV DNA and negative for hepatitis B surface antigen (HBsAg)
    b. Patients with resolved or treated HCV (i.e., HCV antibody positive but undetectable HCV RNA)
10. Patients must not have a history of primary immunodeficiency.
11. Patients must not receive concurrent or prior use of an immunosuppressive agent within 14 days of the first dose of investigational agent, with the following exceptions and notes:
    a. Systemic steroids at physiologic doses (equivalent to dose of oral prednisone 10 mg) are permitted. Steroids as anti-emetics for chemotherapy are not allowed.
    b. Intranasal, inhaled, topical, intra-articular, and ocular corticosteroids with minimal systemic absorption are permitted.
    c. Patients with a condition with anticipated use of systemic steroids above the equivalent of 10 mg prednisone are excluded.
    d. Transient courses of steroids may be approved by the Medical Monitor on a case by case basis, dependent on dose and reason.

12. Patients must not have a history of clinically manifested central nervous system (CNS) metastases.
    a. Patients with known or suspected leptomeningeal disease or cord compression are not eligible.
13. Patients must not have had major surgery as determined by the PI within 4 weeks before the first dose of investigational agent.
14. Patients must not have received another investigational agent within the shorter of 4 weeks or 5 half-lives before the first dose of investigational agent.
15. Patients must not have received a live attenuated vaccine within 28 days before the first dose of investigational agent, and patients, if enrolled, should not receive live vaccines during the study or for 180 days after the last dose of investigational agent.
16. Females who are pregnant or lactating or who intend to become pregnant during participation in the study are not eligible to participate.
17. Patients who have any clinically significant psychiatric, social, or medical condition that, in the opinion of the investigator, could increase the patient's risk, interfere with protocol adherence, or affect the patient's ability to give informed consent are ineligible to participate in the study.

7.3 Discontinuation, Withdrawal, and Replacement of Patients

7.3.1 Discontinuation of Study Drug

An individual patient will not receive any further study drug (discontinue study drug) if any of the following occur in the patient in question:

- Radiological progression, according to RECIST v1.1 (see Section 10.1), with a decline in performance status and significant symptoms due to PD. For criteria for treatment beyond disease progression please see Section 6.1.4.
- Complete withdrawal of consent from the study (no further data collection permitted)
- Withdrawal of consent from further treatment with study drug (data collection as per study schedule permitted)
- Lost to follow-up
- An AE that, in the opinion of the Investigator or the Sponsor, contraindicates further dosing
- DLT during Phase 1b
- Unacceptable Toxicity during Phase 2
- Pregnancy or intent to become pregnant
- Patient noncompliance that, in the opinion of the Investigator or Sponsor, warrants withdrawal (e.g., refusal to adhere to the scheduled visits)
- Initiation of alternative anticancer therapy (excluding surgery or palliative radiotherapy, as permitted per Section 8.9.3), including another investigational agent

- Intercurrent illness: a condition, injury, or disease unrelated to the primary diagnosis that became apparent during treatment and necessitated the patient's termination from the study
- General or specific changes in the patient’s condition that renders him/her ineligible for further treatment according to the inclusion/exclusion criteria

The primary reason for study drug discontinuation should be documented on the appropriate Electronic Case Report Form (eCRF) page.

7.3.2 Withdrawal/Discontinuation of Patients

When study drug is discontinued, patients should have an end of treatment (EOT)/discontinuation assessment and continue follow-up assessments as outlined in the SOAs. Information on survival follow-up and new anticancer therapy will be collected for all patients via telephone calls, patient medical records, and/or clinic visits, for a maximum of 5 years or until any of the following occurs:

- Death
- Lost to follow-up
- Study termination by the Sponsor
- Patient requests to be withdrawn from follow-up
- Investigator requests that the patient is withdrawn from follow-up

If a patient requests to be withdrawn from the study, the request must be documented in the source documents and signed by the Investigator. The primary reason for withdrawal from study should be documented on the appropriate eCRF page. If the patient withdraws from study, the Sponsor may retain and continue to use any data collected before such withdrawal of consent. In addition, the study staff may use a public information source (eg, county records) to obtain information about survival status only. However, patients who withdraw consent will not be followed for any reason after consent has been withdrawn. Patients who withdraw from the study will not be replaced.

7.3.3 Replacement of Patients

In Phase 1b only, patients who do not complete the DLT observation period (as defined in Section 6.1) will be replaced, but these patients will continue on study for safety evaluation until PD or administration of different anti-cancer therapy.

7.4 Follow-Up for Drug Discontinuation/Patient Withdrawal from Study

If a patient discontinues study treatment or is withdrawn from the study and/or treatment for any reason, the study site must immediately notify PICI. The date and the reason for study and/or treatment discontinuation must be recorded on the appropriate eCRF. Patients who discontinue study treatment are to attend the EOT, Day 30 and Day 100 Follow-up Visits to complete all assessments.

In the event that a patient discontinues treatment prematurely due to a TEAE or serious TEAE, the TEAE or serious TEAE will be followed until it resolves (returns to normal or baseline

values) or stabilizes, is judged by the Investigator to be no longer clinically significant or is deemed irreversible.

Once a patient is withdrawn from the study, the patient may not re-enter the study.

7.5 Lost to Follow-Up

A patient will be considered lost to follow-up if he or she repeatedly fails to return for scheduled visits and is unable to be contacted by the study site.

The following actions must be taken if a patient fails to return to the clinic for a required study visit:

- The site must attempt to contact the patient and reschedule the missed visit as soon as possible and counsel the patient on the importance of maintaining the assigned visit schedule and ascertain whether or not the patient wishes to and/or should continue in the study.
- Before a patient is deemed lost to follow-up, the Investigator or designee will make every effort to regain contact with the patient (where possible, 3 telephone calls and, if necessary, a certified letter to the participant's last known mailing address or local equivalent methods). These contact attempts should documented in the patient's medical record or study file.

Should the patient continue to be unreachable, he/she will be considered lost to follow-up and will have completed the study. This should be reflected in the End of Study eCRF.

7.6 Study Termination

This study may be terminated at the discretion of the Sponsor or any regulatory agency. An Investigator may elect to discontinue or stop the study at his or her site for any reason including safety or low enrollment.

8 TREATMENTS

In Phase 1b, patients will be enrolled into 1 of 4 cohorts (Cohort B1, Cohort B2, Cohort C1, and Cohort C2), as outlined in Table 2. A cohort corresponding to Arm A1 of Phase 2 (nivolumab/NP/Gem) will not be tested, since an external study is being conducted to confirm the safety of nivolumab in combination with NP/Gem.

Once the RP2D of APX005M in combination with nivolumab/NP/Gem is determined, patients in Phase 2 will be randomized between Arm A1, Arm B2, or Arm C2. If Arm B2 and/or C2 are determined to be toxic in the Phase 1b study, patients will be randomized between Arm A1, Arm B1, and Arm C1. Arms B1, B2, C1, and C2 will be the same as the corresponding cohorts in the Phase 1b study and Table 2. In Arm A1, patients will receive infusions of nivolumab 240 mg, NP 125 mg/m^2, and Gem 1000 mg/m^2. Twelve DLT-evaluable patients from the Phase 1b study, enrolled at the RP2D of APX005M (i.e., 6 patients on B2 and 6 patients on C2), will be included in the efficacy analysis in Phase 2. The remaining 93 patients will be randomized as per Section 8.6.

Table 2 Study Cohorts/Arms

Cohort/Arm	Dosing Days Per Cycle[a]			
	1	3	8	15
**A1**				
Nivolumab 240 mg IV	X			X
NP 125 mg/m^2 IV	X		X	X
Gem 1000 mg/m^2 IV	X		X	X
**B1**				
NP 125 mg/m^2 IV	X		X	X
Gem 1000 mg/m^2 IV	X		X	X
APX005M 0.1 mg/kg IV		X		
**B2**				
NP 125 mg/m^2 IV	X		X	X
Gem 1000 mg/m^2 IV	X		X	X
APX005M 0.3 mg IV		X		
**C1**				
Nivolumab 240 mg IV	X			X
NP 125 mg/m^2 IV	X		X	X
Gem 1000 mg/m^2 IV	X		X	X
APX005M 0.1 mg/kg IV		X		
**C2**				
Nivolumab 240 mg IV	X			X
NP 125 mg/m^2 IV	X		X	X
Gem 1000 mg/m^2 IV	X		X	X
APX005M 0.3 mg/kg IV		X		

Gem = gemcitabine; IV = intravenous; NP = nab-paclitaxel

a Depending on whether or not the patient experiences toxicity, NP/Gem could be administered according to one of the following dosing schedules: (1) 3 weeks on, 1 week off; and (2) 2 weeks on, 1 week off. Nivolumab should not be administered on Day 15 if NP/Gem is held on Day 15. Nivolumab doses should not be administered < 2 weeks apart.

8.1 Details of Study Treatments

Details of APX005M, nivolumab, NP, and Gem are provided below. Delay of treatment schedule, as allowed by the protocol, is permitted at the discretion of the treating Investigator (e.g. toxicity, weather).

8.1.1 APX005M

Classification: Humanized IgG1 CD40 agonistic antibody

Mechanism of Action: APX005M is a humanized IgG1 CD40 agonistic antibody that binds to CD40, activating the CD40 pathway, leading to activation of APCs, including B cells, monocytes, and DCs, and stimulates cytokine release.

Storage and Stability: APX005M must be stored in a secure location with limited access under controlled temperature conditions of 2°C to 8°C (36°F to 46°F) and in accordance with local regulations. Vials should be stored in their original folding carton to protect from light. During preparation and administration of diluted APX005M product, protection from light is not required.

Dose Specifics: 0.1 or 0.3 mg/kg IV on Day 3 of every Cycle (or Day 10 if delayed). Patients may only receive the delayed dose on Day 10 if they have received NP/Gem on Day 8. APX005M doses should not be administered < 2 weeks apart.

Preparation: APX005M is prepared in normal saline per the Pharmacy Manual.

Administration: Premedication is given as noted in Table 3. After dilution in normal saline, APX005M is administered by IV infusion over 60 minutes. It is recommended that the APX005M infusion does not exceed 120 minutes. APX005M must be administered to study patients by qualified personnel.

Safety Precautions: When handling APX005M, study personnel should wear laboratory coats and disposable protective gloves and avoid contact with eyes, skin, and clothing. APX005M should be protected from light and contamination.

Availability: APX005M will be provided by Apexigen. APX005M must be dispensed only from official study sites by authorized personnel according to local regulations. It is the responsibility of the Investigator to ensure that APX005M is only dispensed to study patients.

Packaging and Labeling: APX005M is supplied in 20 mL Type 1 clear glass vials for IV injection. Each depyrogenated vial contains 10 mg/mL APX005M in a sterile, clear to slight opalescent, colorless to slightly yellow, preservative-free solution (pH 5.5) containing 25 mM sodium acetate, 248 mM trehalose, and 0.02% polysorbate 20 in water for injection with a target fill volume of 16.9 mL per vial. Glass vials are plugged with Teflon-coated rubber stoppers and sealed with aluminum seals. The 20 mL vials (16.9 mL/vial) are intended for single use. Additional APX005M details are provided in the Pharmacy Manual.

Return and Destruction of APX005M: Upon completion or termination of the study, all unused and/or partially used APX005M must be returned to Apexigen or designee if not authorized by Apexigen or designee to be destroyed at the site. All returned APX005M must be accompanied by the appropriate documentation and clearly identified by protocol number and study site number on the outermost shipping container. Return supplies should be in the original containers (e.g., kits that have clinical labels attached). Empty containers should not

be returned. It is the Investigator's responsibility to arrange for disposal of all empty containers, provided that procedures for proper disposal have been established according to the applicable federal, state, local, and institutional guidelines and procedures and appropriate records of disposal are kept. The return of unused APX005M should be arranged by the responsible Study Monitor.

Side Effects: Symptoms associated with cytokine release syndrome (including but not limited to flushing, itchiness, chills, fever, rash, tachycardia, hypotension, hypertension, rigor, and myalgia) after administration of APX005M are possible and have been observed in some of the patients receiving APX005M. Guidance for monitoring and management of cytokine release syndrome is included (in Section 13.6 of this protocol and in the APX005M IB).

Transient transaminase elevations (≤ Grade 2) have been observed in several patients with liver metastases, which were not associated with a particular dose of APX005M. Six patients with liver metastases enrolled in the study experienced a transient increase in total bilirubin. Liver function test abnormalities tend to resolve to baseline within 7 days from APX005M administration.

Transient decreases in peripheral blood lymphocyte count in general and B-cell count in particular have been observed for APX005M as well as for other CD40-agonistic mAbs, and are believed to be a PD effect. Transient decreases in platelet counts were observed for some of the patients receiving higher doses of APX005M but were not associated with bleeding or other clinical manifestations.

Other symptoms might also occur, including allergic reactions, which could be severe, pulmonary edema, and rarely, thromboembolic events, myocardial infarction and/or death.

In the ongoing Phase 1 study APX005M-001, APX005M demonstrated a dose-dependent activation of APCs, T cell activation and increases in circulating levels of cytokines.

8.1.2 Nivolumab

Other Names: Opdivo, BMS-936558, MDX-1106

Classification: Human PD-1 antibody

Mechanism of Action: Binding of the PD-1 ligands, PD-L1 and PD-L2, to the PD-1 receptor found on T cells, inhibits T-cell proliferation and cytokine production. Upregulation of PD-1 ligands occurs in some tumors and signaling through this pathway can contribute to inhibition of active T-cell immune surveillance of tumors. Nivolumab is a human IgG4 mAb that binds to the PD-1 receptor and blocks its interaction with PD-L1 and PD-L2, releasing PD-1 pathway-mediated inhibition of the immune response, including the anti-tumor immune response. In syngeneic mouse tumor models, blocking PD-1 activity resulted in decreased tumor growth.

Storage and Stability: Vials of nivolumab injection must be stored at 2°C to 8°C (36°F to 46°F) and protected from light and freezing. The unopened vials can be stored at room temperature (up to 25°C, 77°F) and room light for up to 48 hours. The administration of infusion must be completed within 24 hours of preparation. If not used immediately, the infusion solution may be stored under refrigeration conditions (2°C to 8°C, 36°F to 46°F) for up to 24 hours, and a maximum of 8 hours of the total 24 hours can be at room temperature

(20°C to 25°C, 68°F to 77°F) and room light. The maximum 8-hour period under room temperature and room light conditions includes the product administration period.

Dose Specifics: 240 mg IV Day 1 and 15 of every 28-day cycle. Nivolumab doses should not be administered < 2 weeks apart. Nivolumab should not be administered on Day 15 if NP/Gem is held on Day 15.

Preparation: Withdraw the required volume of nivolumab and transfer to an IV container. Dilute nivolumab with 0.9% sodium chloride injection, United States Pharmacopeia (USP) or 5% dextrose injection, USP, to prepare an infusion with a final concentration ranging from 1 mg/mL to 10 mg/mL. Mixed diluted solution by gentle inversion; do not shake. Discard partially used vials of nivolumab.

Administration: Administer the infusion over 30 minutes through an IV line containing a sterile, nonpyrogenic, low protein binding in-line filter (pore size of 0.2 µm to 1.2 µm). Do not co-administer other drugs through the same IV line. Flush IV line at the end of the infusion.

Safety Precautions: When handling nivolumab, wear laboratory coats and disposable protective gloves. Avoid contact with eyes, skin, and clothing. Protect from light and contamination.

Availability: Nivolumab will be provided by Bristol-Myers Squibb in 100 mg vials (10 mg/mL) and labeled appropriately as investigational material for this study.

Side Effects:

- General disorders: Fatigue, anorexia, pyrexia, headache
- Respiratory, thoracic, and mediastinal disorders: Cough, upper respiratory tract infection, pneumonitis
- Hepatic: Increased AST, ALT, alkaline phosphatase, hepatitis
- Gastrointestinal: Diarrhea, constipation, nausea, vomiting, colitis
- Skin: Rash, pruritus, vitiligo
- Endocrine: Hypophysitis, thyroiditis
- Ophthalmologic: Uveitis

For management of toxicities, see Section 8.5.

8.1.3 Nab-Paclitaxel

Other Names: Abraxane

Classification: Mitotic inhibitor (cytoskeletal target)

Mechanism of Action: NP is a Cremophor EL-free, albumin-bound paclitaxel particle with a mean size of approximately 130 nm. NP is a unique protein formulation of a non-crystalline, amorphous form of paclitaxel in an insoluble particle state.

Paclitaxel is a cytoskeletal drug that targets tubulin. Unlike tubulin-targeting drugs (such as colchicine) that inhibit microtubule assembly, paclitaxel stabilizes the microtubule polymer and protects it from disassembly. Therefore, chromosomes are unable to form a metaphase

spindle formation. This blocks progression of mitosis. Prolonged activation of the mitotic checkpoint will then lead to apoptosis or lead the cell to return to G-phase without cell division.

Storage and Stability: NP should be stored as vials in original cartons at 20ºC to 25ºC (68ºF to 77ºF) and protected from bright light.

Dose Specifics: In the absence of toxicity, NP will be administered at 125 mg/m2 IV Day 1, 8 and 15 of every 28-day cycle (ie, 3 weeks on, 1 week off cycle). If toxicity occurs, NP could be administered on a 2 weeks on, 1 week off cycle (see Section 8.5 for dose modification guidelines). For example, patients whose Day 8 dose of NP is delayed due to toxicity may receive the dose on Day 22. However, to receive the Day 22 dose, they must have received APX005M on Day 3.

Preparation: Reconstitute each vial containing 100 mg of NP by injecting 20 mL of 0.9% Sodium Chloride Injection. Each mL of the reconstituted formulation will contain 5 mg/mL paclitaxel.

Administration: This drug may be administered IV as prepared above over 30 minutes. Given the possibility of extravasation, it is advisable to closely monitor the infusion site for possible infiltration during drug administration.

Availability: NP is commercially available as 100 mg of paclitaxel in a single-use vial.

Side Effects:

- Hematologic Disorders: Neutropenia was dose dependent and reversible. Pancytopenia has been observed in clinical studies.
- Infections: Infectious episodes were reported in 24% of the patients treated with paclitaxel. Oral candidiasis, respiratory tract infections and pneumonia were the most frequently reported infectious complications.
- Hypersensitivity Reactions (HSRs): Grade 1 or 2 HSRs occurred on the day of paclitaxel administration and consisted of dyspnea (1%) and flushing, hypotension, chest pain, and arrhythmia (all < 1%).
- Cardiovascular: Hypotension, during the 30-minute infusion, occurred in 5% of patients. Bradycardia, during the 30-minute infusion, occurred in < 1% of patients. These vital sign changes most often caused no symptoms and required neither specific therapy nor treatment discontinuation. Severe cardiovascular events possibly related to single-agent paclitaxel occurred in approximately 3% of patients. These events included cardiac ischemia/infarction, chest pain, cardiac arrest, supraventricular tachycardia, edema, thrombosis, pulmonary thromboembolism, pulmonary emboli, and hypertension. Cases of cerebrovascular attacks (strokes) and transient ischemic attacks have been reported. Electrocardiogram (ECG) abnormalities were common among patients at baseline. ECG abnormalities on study did not usually result in symptoms, were not dose-limiting, and required no intervention. ECG abnormalities were noted in 60% of patients. Among patients with a normal ECG prior to study entry, 35% of all patients developed an abnormal tracing while on study. The most frequently reported ECG modifications were non-specific repolarization abnormalities, sinus bradycardia, and sinus tachycardia.

- Respiratory: Dyspnea (12%), cough (7%), and pneumothorax (< 1%) were reported after treatment with paclitaxel.
- Neurologic: The frequency and severity of sensory neuropathy increased with cumulative dose. Sensory neuropathy was the cause of paclitaxel discontinuation in 7/229 (3%) patients. Twenty-four patients (10%) treated with paclitaxel developed Grade 3 peripheral neuropathy; of these patients, 14 had documented improvement after a median of 22 days; 10 patients resumed treatment at a reduced dose of paclitaxel and 2 discontinued due to peripheral neuropathy. Of the 10 patients without documented improvement, 4 discontinued the study due to peripheral neuropathy. No Grade 4 sensory neuropathies were reported. Only one incident of motor neuropathy (Grade 2) was observed in either arm of the controlled study.
- Vision Disorders: Ocular/visual disturbances occurred in 13% of all patients (n = 366) treated with paclitaxel and 1% were severe. The severe cases (keratitis and blurred vision) were reported in patients who received higher doses than those recommended (300 or 375 mg/m2). These effects generally have been reversible. Other possible side effects include conjunctivitis and increased lacrimation.
- Arthralgia/Myalgia: The symptoms were usually transient, occurred two or three days after paclitaxel administration, and resolved within a few days.
- Hepatic: Grade 3 or 4 elevations in gamma-glutamyl transpeptidase were reported for 14% of patients treated with paclitaxel.
- Renal: Overall 11% of patients experienced creatinine elevation, 1% severe. No discontinuations, dose reductions, or dose delays were caused by renal toxicities.

Other Clinical Events: Nail changes (changes in pigmentation or discoloration of nail bed) have been reported. Edema occurred in 10% of patients; no patients had severe edema. Dehydration and pyrexia were also reported. Skin reactions including generalized or maculopapular rash, erythema, and pruritus have been observed with paclitaxel. There have been case reports of photosensitivity reactions, radiation recall phenomenon, and in some patients previously exposed to capecitabine, reports of palmar-plantar erythrodysesthesia. Stevens-Johnson syndrome and toxic epidermal necrolysis have been reported. There have been reports of conjunctivitis, cellulitis, and increased lacrimation with paclitaxel injection.

8.1.4 Gemcitabine

Other Names: 2'-Deoxy-2', 2’-difluorocytidine monohydrochloride, Gemzar

Classification: Antimetabolite (nucleoside analog)

Mechanism of Action: Gem exhibits cell phase specificity, primarily killing cells undergoing DNA synthesis (S phase) and also blocking the progression of cells through the G1/S phase boundary. Gem is metabolized intracellularly by nucleoside kinases to the active diphosphate and triphosphate nucleosides. The cytotoxic effect of Gem is attributed to a combination of two actions of the diphosphate and the triphosphate nucleosides, which leads to inhibition of DNA synthesis. First, Gem diphosphate inhibits ribonucleotide reductase, which is responsible for catalyzing the reactions that generate the deoxynucleoside triphosphates for DNA synthesis. Inhibition of this enzyme by the diphosphate nucleoside causes a reduction in the concentrations of deoxynucleotides, including deoxycytidine triphosphate (dCTP). Second,

Gem triphosphate competes with dCTP for incorporation into DNA. The reduction in the intracellular concentration of dCTP (by the action of the diphosphate) enhances the incorporation of Gem triphosphate into DNA (self-potentiation). After the Gem nucleotide is incorporated into DNA, only one additional nucleotide is added to the growing DNA strands. After this addition, there is inhibition of further DNA synthesis. DNA polymerase epsilon is unable to remove the Gem nucleotide and repair the growing DNA strands (masked chain termination). In T lymphoblastoid cells of the CEM cell line, Gem induces internucleosomal DNA fragmentation, one of the characteristics of programmed cell death.

Storage and Stability: Un-reconstituted drug vials are stored at controlled room temperature. Reconstituted solution should be stored at controlled room temperature and used within 24 hours. Solutions of Gem should not be refrigerated; crystallization may occur. The unused portion should be discarded.

Dose Specifics: Gem is indicated as a single agent for the treatment of PC. In this indication, a dose of 1000 mg/m2 over 30 minutes once weekly for up to 7 weeks followed by a week of rest, then once weekly for 3 weeks of every 4 weeks is used. In this protocol, in the absence of toxicity, we use a common modification of Gem dosing of 3 weeks on, 1 week off. Therefore, 1000 mg/m2 IV is used on Days 1, 8 and 15 of every 28-day cycle. If toxicity occurs, Gem could be administered on a 2 weeks on, 1 week off cycle (see Section 8.5 for dose modification guidelines). For example, patients whose Day 8 dose of Gem is delayed due to toxicity may receive the dose on Day 22. However, to receive the Day 22 dose, they must have received APX005M on Day 3.

Preparation: Reconstitute the 200-mg vial with 5 mL and the 1-g vial with 25 mL preservative-free normal saline to make a solution containing 38 mg/mL. Shake to dissolve.

Administration: The drug may be administered IV as prepared above or further diluted with normal saline to a minimum concentration of 0.1 mg/mL. Gem is commonly diluted in 100 or 250 mL of saline. Gem administration will be over 30 minutes.

Availability: Gemcitabine is commercially available in 200-mg and 1-g vials.

Side Effects:

- Hematologic: Myelosuppression manifested by neutropenia, thrombocytopenia, and anemia occurs with Gem as a single agent and the risks are increased when Gemcitabine is combined with other cytotoxic drugs. In clinical studies, Grade 3-4 neutropenia, anemia, and thrombocytopenia occurred in 25%, 8%, and 5%, respectively of patients receiving single-agent. The frequencies of Grade 3-4 neutropenia, anemia, and thrombocytopenia varied from 48% to 71%, 8% to 28%, and 5% to 55%, respectively, in patients receiving Gem in combination with another drug.
- Dermatologic: A rash is seen in about 25% of patients and is associated with pruritus in about 10% of patients. The rash is usually mild, not dose-limiting, and responds to local therapy. Desquamation, vesiculation, and ulceration have been reported rarely. Alopecia is reported in < 1% of patients.
- Gastrointestinal: Nausea and vomiting are reported in about two-thirds of patients and requires therapy in about 20% of patients. It is rarely (< 1%) dose-limiting, and is easily

manageable with standard antiemetics. Diarrhea is reported in 8% of patients, constipation in 6%, and oral toxicity in 7%.

- Hepatic: Abnormalities of hepatic transaminase enzymes occur in two-thirds of patients, but they are usually mild, nonprogressive, and rarely necessitate stopping treatment. Drug-induced liver injury, including liver failure and death, has been reported in patients receiving Gem alone or in combination with other potentially hepatotoxic drugs. Administration of Gem in patients with concurrent liver metastases or a pre-existing medical history of hepatitis, alcoholism, or liver cirrhosis can lead to exacerbation of the underlying hepatic insufficiency.
- Pulmonary: Bronchospasm after injection has been reported in less than 1% of patients and is usually mild and transient, but parenteral therapy may be required. Dyspnea within a few hours of injection is reported in 10% of patients. It is usually mild, short-lived, rarely dose-limiting, and usually abates without any specific therapy. Cough and rhinitis are also commonly reported. Pulmonary toxicity, including interstitial pneumonitis, pulmonary fibrosis, pulmonary edema, and adult respiratory distress syndrome, has been reported. In some cases, these pulmonary events can lead to fatal respiratory failure despite discontinuation of therapy. The onset of pulmonary symptoms may occur up to 2 weeks after the last dose of Gem.
- Neurologic: Somnolence has been reported in 10% of patients, and insomnia is common.
- Cardiovascular: A few cases of hypotension were reported. Some cases of myocardial infarction, congestive heart failure, and arrhythmia have been reported, but there is no clear evidence that Gem causes cardiac toxicity. Peripheral edema is reported in about 30% of patients. Some cases of facial edema have also been reported. Edema is usually mild to moderate, rarely dose-limiting, sometimes painful, and reversible after stopping Gem treatment.
- Hemolytic Uremic Syndrome (HUS): HUS to include fatalities from renal failure or the requirement for dialysis can occur in patients treated with Gem. In clinical studies, HUS was reported in 6 of 2429 patients (0.25%). Most fatal cases of renal failure were due to HUS. Renal failure may not be reversible even with discontinuation of therapy.
- Embryofetal Toxicity: Gem can cause fetal harm when administered to a pregnant woman, based on its mechanism of action. Gem was teratogenic, embryotoxic, and fetotoxic in mice and rabbits. If this drug is used during pregnancy, or if a woman becomes pregnant while taking Gem, the patient should be apprised of the potential hazard to a fetus.
- Exacerbation of Radiation Therapy Toxicity: Gem is not indicated for use in combination with radiation therapy. Concurrent (given together or ≤ 7 days apart) — Life-threatening mucositis, especially esophagitis and pneumonitis occurred in a study in which Gem was administered at a dose of 1000 mg/m_2 to patients with non-small cell lung cancer for up to 6 consecutive weeks concurrently with thoracic radiation.

- Capillary Leak Syndrome: Capillary leak syndrome with severe consequences has been reported in patients receiving Gem as a single agent or in combination with other chemotherapeutic agents.
- Other: Flu-like symptoms are reported for about 20% of patients. This includes fever, headache, back pain, chills, myalgia, asthenia, and anorexia. Malaise and sweating are also commonly reported.

8.2 Preparation and Administration of Study Treatment

For all study drugs, either a peripheral IV or a central port or line is acceptable for infusion. Patient weight will be assessed at Screening and Day 1 of each cycle. Dose adjustments are not required unless the subject has a ≥ 5% change in comparison to their initial weight on Cycle 1, Day 1.

8.2.1 APX005M

APX005M will be prepared as per the Pharmacy Manual. Briefly, APX005M will be diluted in normal saline. After dilution in normal saline, APX005M will be administered by IV infusion over 60 minutes on Day 3 of each 28-day cycle. The APX005M infusion can be interrupted in the case of infusion reaction. Once symptoms resolve, infusion should be restarted at 50% of the initial infusion rate (e.g., from 50 mL/h to 25 mL/h).

For each treatment cycle, APX005M should only be administered if patients received NP/Gem on Day 1. For APX005M dose delays (from Day 3 to Day 10), patients may only receive the Day 10 dose if they have received NP/Gem on Day 8.

8.2.2 Nivolumab

Nivolumab will be prepared according to the package insert. Briefly, the required volume of nivolumab will be withdrawn and transferred to an IV container. Nivolumab will be diluted with 0.9% sodium chloride injection, USP or 5% dextrose injection, USP, to prepare an infusion with a final concentration ranging from 1 mg/mL to 10 mg/mL. This diluted solution will be mixed by gentle inversion.

The dose will be administered over 30 minutes through an IV line containing a sterile, nonpyrogenic, low protein binding in-line filter (pore size of 0.2 µm to 1.2 µm) on Days 1 and 15 of each 28-day cycle. Nivolumab doses should not be administered < 2 weeks apart. Nivolumab should not be administered on Day 15 if NP/Gem is held on Day 15. **On each day nivolumab is given, it should be administered before Gem and NP. Do not co-administer other drugs through the same IV line.** The IV line should be flushed at the end of the infusion.

8.2.3 Nab-paclitaxel

NP will be prepared according to the package insert. Briefly, each vial of NP containing 100 mg of drug will be reconstituted by injecting 20 mL of 0.9% sodium chloride injection. Each milliliter of the reconstituted formulation will contain 5 mg/mL paclitaxel. This dose will be administered IV as prepared above and over 30 minutes. In the absence of toxicity, NP could be administered on Days 1, 8, and 15 of each 28-day cycle (ie, 3 weeks on, 1 week off cycle). If toxicity occurs, NP may be administered on a 2 weeks on, 1 week off cycle. **NP should be given after nivolumab and before Gem when given on the same days as either**

of these drugs. Given the possibility of extravasation, it is advisable to closely monitor the infusion site for possible infiltration during drug administration.

For NP dose delays (from Day 8 to Day 22), patients may only receive the Day 22 dose if they have received APX005M on Day 3.

8.2.4 Gemcitabine

Gem will be prepared according to the package insert. Briefly, the 200-mg vial of Gem will be reconstituted with 5 mL and the 1-g vial with 25 mL preservative-free- normal saline to make a solution containing 38 mg/ml. This will be shaken and dissolved. The dose will be administered IV as prepared above or further diluted with normal saline to a minimum concentration of 0.1 mg/mL. Gem is commonly diluted in 100 mL or 250 mL of saline, and administered IV over 30 minutes. In the absence of toxicity, Gem could be administered on Days 1, 8, and 15 of each 28-day cycle (ie, every 3 weeks on, 1 week off cycle). If toxicity occurs, Gem may be administered on a 2 weeks on, 1 week off cycle. **Gem should be administered after nivolumab and NP when given on the same days**.

For Gem dose delays (from Day 8 to Day 22), patients may only receive the Day 22 dose if they have received APX005M on Day 3.

8.3 Monitoring Following APX005M Administration

Patients will be monitored during and after the infusion of APX005 for at least 5 hours after the first 2 infusions of APX005M and as clinically indicated thereafter. All patients will be discharged from the clinic after clinical evaluation. Patients should have stable vital signs, including lack of orthostatic hypotension (systolic blood pressure > 100 mmHg, or a reduction to no lower than 10 mmHg from baseline) without IV hydration (no hydration for at least 2 hours before discharge), lack of hypoxia (oxygen saturation > 90% without oxygen), temperature < 38ºC, and heart rate < 110 beats/min. After discharge, all patients should be monitored by a caregiver or by a healthcare professional for 24 hours after the first 2 infusions of APX005M and as clinically indicated thereafter.

8.4 Dosage Schedule

Study treatments will be administered as described in Table 3.

Table 3 Treatment Regimens and Schedule

**Arm A1**				
**Drug**	**Premedication**	**Dose**	**Route**	**Cycle**[a]
Nivolumab	None	240 mg	IV over 30 min	Days 1, 15
NP	Per institutional standards[b]	125 mg/m^2 [c]	IV over 30 min	Days 1, 8, 15
Gem		1000 mg/m^2 [c]	IV over 30 min	Days 1, 8, 15
**Arm B1**				
NP	Per institutional standards[b]	125 mg/m^2 [c]	IV over 30 min	Days 1, 8, 15
Gem		1000 mg/m^2 [c]	IV over 30 min	Days 1, 8, 15
APX005M	Yes[d]	0.1 mg/kg	IV over 60 min	Day 3
**Arm B2**				
NP		125 mg/m^2 [c]	IV over 30 min	Days 1, 8, 15

Gem	Per institutional standards[b]	1000 mg/m^2 [c]	IV over 30 min	Days 1, 8, 15
APX005M	Yes[d]	0.3 mg/kg	IV over 60 min	Day 3
**Arm C1**				
Nivolumab	None	240 mg	IV over 30 min	Days 1, 15
NP	Per institutional standards[b]	125 mg/m^2 [c]	IV over 30 min	Days 1, 8, 15
Gem		1000 mg/m^2 [c]	IV over 30 min	Days 1, 8, 15
APX005M	Yes[d]	0.1 mg/kg	IV over 60 min	Day 3
**Arm C2**				
Nivolumab	None	240 mg	IV over 30 min	Days 1, 15
NP	Per institutional standards[b]	125 mg/m^2 [c]	IV over 30 min	Days 1, 8, 15
Gem		1000 mg/m^2 [c]	IV over 30 min	Days 1, 8, 15
APX005M	Yes[d]	0.3 mg/kg	IV over 60 min	Day 3

Gem = gemcitabine; IV = intravenous(ly); NP = nab-paclitaxel

[a] Each cycle is 28 days. However, depending on whether or not the patient experiences toxicity, NP/Gem could be administered according to one of the following dosing schedules: (1) 3 weeks on, 1 week off; and (2) 2 weeks on, 1 week off. Nivolumab should not be administered on Day 15 if NP/Gem is held on Day 15. Nivolumab and APX005M doses should not be administered <2 weeks apart. Delay of treatment schedule, as allowed by the protocol, is permitted at the discretion of the treating Investigator (e.g. toxicity, weather).

[b] Institutional standards may include a 5-HT3 antagonist (ondansetron, granisetron), but steroids such as dexamethasone should not be given as an anti-emetic unless treatment with a 5-HT3 antagonist or other nausea medications is ineffective in treating nausea and the overall PI has been notified.

[c] Per commercial package insert.

[d] Premedication is given 30 minutes before each administration of APX005M and includes a regimen containing an oral H_1 antagonist (e.g., loratadine 10 mg), oral non-steroidal antiinflammatory- (e.g., ibuprofen 400 mg), acetaminophen 650 mg, and optionally an oral H_2 antagonist (e.g., ranitidine 150-300 mg, famotidine 10-40 mg), or also optionally, ondansetron doses (route of administration, and frequency per institutional standards). A window of -10 minutes is permitted (e.g. premedications may be administered up to 40 minutes, but no later than 30 minutes, prior to APX005M).

8.5 Management of Study Drug-Related Toxicities

Management of suspected adverse drug reactions may require temporary treatment hold, reducing the dose of APX005M, NP or Gem, or discontinuation of some or all investigational products as presented in the following sections. If a patient experiences several toxicities, the recommended dose modification should be based on the highest grade toxicity.

Up to 2 dose reductions are permitted for APX005M and Gem, and up to 3 dose reductions are permitted for NP. No dose reductions will be permitted for nivolumab per Table 4 and Table 7.

These dose adjustments are for AEs deemed related to the study medications. If, in the opinion of the treating Investigator, a toxicity is thought to be unrelated to study medications and resolves to a "Continue" or below lowest grade in Table 4, no dose adjustments for the study medications are necessary.

For NP and/or Gem-related toxicity leading to discontinuation of the chemotherapy, treatment with APX005M and nivolumab, or either agent alone, may be continued with approval of the Medical Monitor.

For any toxicity (regardless of grade) that, despite optimal supportive care, is felt by the treating Investigator to present a risk to the patient safety, additional dose reduction, treatment

delay, or treatment discontinuation is permitted at the discretion of the treating Investigator. Dose re-escalation will be permitted with approval of the Medical Monitor.

In the event APX005M is discontinued, due to drug-related toxicities, the participant may continue other study treatments with the approval of the Medical Monitor. In this case, Day 3 assessments listed in Section 9 will no longer be required.

8.5.1 Day 1

Guidelines for management of study treatment-related toxicity on Day 1 are summarized in Table 4.

Table 4 Management of Study Treatment-Related Toxicities on Day 1

Toxicity	Grade	Nivolumab	NP	Gem
Diarrhea/<br>colitis	2	1st occurrence: hold<br>2nd occurrence or if not improving in 1 week with steroids: discontinue	Continue	Continue
	3	Discontinue	Dose reduction	Dose reduction
	4	Discontinue	Hold. Dose reduction	Hold. Dose reduction
Mucositis	3-4	Hold	Hold. Dose reduction	Hold. Dose reduction
Vomiting	2-3	Hold	Continue. Add antiemetics	Continue. Add antiemetics
	4	Hold. Add antiemetics	Hold. Dose reduction. Add antiemetics	Hold. Dose reduction. Add antiemetics
Increased total bilirubin	2	If patient has a normal baseline AST, ALT, hold.<br><br>If patient's baseline AST, ALT is within Grade 1 toxicity range, continue	Hold.<br>Discontinue if not resolved in 4 weeks.	Hold.<br>Discontinue if not resolved in 4 weeks.
	3	Hold		
	4	Discontinue	Hold. Dose reduction. Discontinue if not resolved in 4 weeks.	Hold. Dose reduction. Discontinue if not resolved in 4 weeks.
Increased AST, ALT	2	If patient has a normal baseline AST, ALT, hold.<br><br>If patient's baseline AST, ALT is within Grade 1 toxicity range, continue	Continue	Continue
	3	Hold	Hold	Hold
	4	Discontinue	Discontinue	Discontinue
Hypophysitis/<br>Endocrino-<br>pathies	2	Hold	Continue	Continue
	3-4	1st occurrence: hold. Institute endocrine replacement therapy	Continue	Continue
Hyperthyroid<br>-ism	3	1st occurrence: hold<br>2nd occurrence: discontinue	Continue	Continue

Toxicity	Grade			
		Nivolumab	NP	Gem
	4	Discontinue	Hold	Hold
Rash or other skin toxicity	2	Continue	Dose reduction	Dose reduction
	3	Hold	Dose reduction	Dose reduction
	4	Discontinue	Discontinue	Discontinue
Neurological Toxicity	2	Hold	Dose reduction	Continue
	3-4	Discontinue	Hold. Dose reduction Discontinue if not resolved in 4 weeks	Continue
Infusion reaction	2	1st occurrence: continue 2nd occurrence: discontinue	Continue	Continue
	3	Discontinue	Hold	Hold
	4	Discontinue	Hold[a]	Hold
Pneumonitis	2	1st occurrence: hold 2nd occurrence or if not improving in 2 weeks: discontinue	Hold	Hold
	3-4	Discontinue	Discontinue	Discontinue
Creatinine Elevation	2	Hold If not improving in 1 week: discontinue	Continue	Continue
	3	Hold If not improving in 1 week: discontinue	Hold. Dose reduction. Discontinue if not resolved in 4 weeks.	Hold. Dose reduction. Discontinue if not resolved in 4 weeks
	4	Discontinue	Discontinue	Discontinue
Hypotension, dehydration or suspected adrenal crisis	3	Hold	Hold	Hold
	4	Discontinue	Discontinue	Discontinue
Fatigue	2 (for >6 weeks) 3	Grade 2: continue Grade 3: hold	Grade 2: continue Grade 3: hold	Grade 2: continue Grade 3: hold
Uveitis, eye pain, or blurred vision	2-4	Hold<br><br>Discontinue if not improved to grade 1 with topical treatment within 2 weeks or if requires systemic therapy	Hold	Hold

Toxicity	Grade	Nivolumab	NP	Gem
Neutropenia	3<br>4 ≤ 7 days	Hold	Dose reduction.[b]<br>Discontinue if not resolved in 4 weeks	Dose reduction.[b]<br>Discontinue if not resolved in 4 weeks
	4 > 7 days	Discontinue	Hold. Dose reduction. Discontinue if not resolved in 4 weeks.	Hold. Dose reduction. Discontinue if not resolved in 4 weeks.
Febrile Neutropenia	3-4	Hold	Hold. Dose reduction. Discontinue if not resolved in 4 weeks.	1st occurrence: hold. Dose reduction.<br>2nd occurrence: hold. 2 dose reductions.<br>Discontinue if not resolved in 4 weeks
Thrombocytopenia	2	Continue	Dose reduction.[b]<br>Discontinue if not resolved in 4 weeks	Dose reduction.[b]<br>Discontinue if not resolved in 4 weeks
	3	Hold	Hold. Dose reduction. Discontinue if not resolved in 4 weeks	Hold. Dose reduction. Discontinue if not resolved in 4 weeks
	3 with significant bleeding or transfusion<br>4	Discontinue	Hold. Dose reduction. Discontinue if not resolved in 4 weeks	Hold. Dose reduction. Discontinue if not resolved in 4 weeks
All Other Drug-Related Toxicity	3 or Severe	Hold	Hold[c] Dose reduction. Discontinue if not resolved in 4 weeks.	Hold. Dose reduction. Discontinue if not resolved in 4 weeks.
	4	Discontinue	Discontinue[d]	Discontinue[d]

Gem = gemcitabine; NP = nab-paclitaxel
Hold = Hold dosing of investigational product until toxicity resolves to “Continue” or below lowest Grade stated in this table.
Dose reduction = reduce dose by one dose level on following dose. The dose reduction applies to all subsequent doses.
Discontinue = permanently discontinue that investigational product
Note: The modifications in this table do not apply to Cycle 1.

a If grade 4 infusion reaction is thought to be related to NP hypersensitivity, nab-paclitaxel should be permanently discontinued.

b Reduce current dose. NP and Gem dose can be re-escalated to previous dose level for Grade 3 neutropenia and Grade 2 thrombocytopenia < 1 week

c Any other grade 3 nivolumab-related adverse event lasting > 7 days, except endocrinopathies controlled with physiologic hormone replacement or laboratory abnormalities, except as noted above, requires discontinuation of nivolumab.

d Patients may only continue treatment for transient Grade 4 toxicity with approval of the Medical Monitor.

8.5.2 Day 3

Guidelines for management of study treatment-related toxicities on Day 3 are summarized in Table 5.

Table 5 Management of Study Treatment-Related Toxicities on Day 3

Toxicity	Grade	APX005M
**Non-Hematologic Toxicities**		
Diarrhea/colitis	2	1st occurrence: hold<br>2nd occurrence: dose reduction
	3	1st occurrence: dose reduction<br>2nd occurrence: discontinue
	4	Discontinue
Mucositis	3-4	Continue
Vomiting	2-3	Hold. Add antiemetics
	4	Hold. Add antiemetics
Increased bilirubin	2	Hold
	3-4	Discontinue
Increased AST, ALT	2	Hold
	3	Hold. Dose reduction<br>For patients with liver metastasis reduce dose only if Grade 3 > 72 hours
	4	Discontinue
Hypophysitis/<br>Endocrinopathies	2	Continue
	3-4	1st occurrence: hold. Institute endocrine replacement therapy<br>2nd occurrence: discontinue
Hyperthyroidism	3	Hold. Dose reduction
	4	Discontinue
Rash or other skin toxicity	2-3	Hold
	4	Discontinue
Neurological Toxicity	2	Continue
	3-4	Hold
Infusion reaction	2	Continue
	3	Hold. Dose reduction.
	4	Discontinue
APX005M cytokine release syndrome	3	Dose reduction
	4	Discontinue
Pneumonitis	2	1st occurrence: hold<br>2nd occurrence: discontinue
	3-4	Discontinue
Creatinine Elevation	2	Hold and reassess on Day 10
	3	Hold
	4	Discontinue
Hypotension, dehydration or suspected adrenal crisis	3	Hold. Dose reduction
	4	Discontinue

Toxicity	Grade	APX005M
Fatigue	2 (for > 6 weeks) or 3	Dose reduction
All Other Drug-Related Toxicity	3 or Severe	1 occurrence: hold<br>2nd occurrence: dose reduction
	4	Discontinue
**Hematologic Toxicities**		
Neutropenia	3	Continue
	4	Hold
Febrile Neutropenia	3-4	Hold
Thrombocytopenia	2	Hold
	3	Hold
	3 with significant bleeding or transfusion<br>4	Hold. Dose reduction

Gem = gemcitabine; NP = nab-paclitaxel
Hold = Hold dosing of investigational product until toxicity resolves to "Continue" or below lowest Grade stated in this table. Dose of APX005M held on Day 3 can be administered on Day 10 if criteria for treatment continuation within a cycle are met and the subject received Day 8 NP/Gem. New cycles should be delayed until criteria for initiation of a new cycle are met (Section 8.5.5).
Dose reduction = reduce dose by one dose level on following dose
Discontinue = permanently discontinue that investigational product

8.5.3 Day 8

Guidelines for management of study treatment-related toxicities on Day 8 are summarized in Table 6.

Table 6 Management of Study Treatment-Related Toxicities on Day 8

**Toxicity**	**Grade**	**NP**	**Gem**
**Non-Hematologic Toxicities**			
Diarrhea/colitis	2	Continue	Continue
	3	Dose reduction	Dose reduction
	4	Hold. Dose reduction	Hold. Dose reduction
Mucositis	3-4	Hold. Dose reduction	Hold. Dose reduction
Vomiting	2-3	Continue. Add antiemetics	Continue. Add antiemetics
	4	Hold. Dose reduction. Add antiemetics	Hold. Dose reduction. Add antiemetics

**Toxicity**	**Grade**	**NP**	**Gem**
Increased bilirubin	2	Hold. Discontinue if not resolved in 4 weeks.	Hold. Discontinue if not resolved in 4 weeks.
	3-4	Hold. Dose reduction. Discontinue if not resolved in 4 weeks.	Hold. Dose reduction. Discontinue if not resolved in 4 weeks.
Increased AST, ALT	2	Continue	Continue
	3	Hold	Hold
	4	Discontinue	Discontinue
Hypophysitis/ Endocrinopathies	2	Continue	Continue
	3-4	Continue	Continue
Hyperthyroidism	3	Continue	Continue
	4	Hold	Hold
Rash or other skin toxicity	2-3	Dose reduction	Dose reduction
	4	Discontinue	Discontinue
Neurological Toxicity	2	Dose reduction	Continue
	3-4	Hold. Dose reduction Discontinue if not resolved in 4 weeks	Continue
Infusion reaction	2	Continue	Continue
	3	Hold	Hold
	4	Discontinue	Hold
Pneumonitis	2	Hold	Hold
	3-4	Discontinue	Discontinue
Creatinine Elevation	2	Continue	Continue
	3	Hold. Dose reduction. Discontinue if not resolved in 4 weeks.	Hold. Dose reduction. Discontinue if not resolved in 4 weeks
	4	Discontinue	Discontinue
Hypotension, dehydration or suspected adrenal crisis	3	Hold	Hold
	4	Discontinue	Discontinue
Fatigue	2 (for >6 weeks) or 3	Grade 2: continue Grade 3: hold	Grade 2: continue Grade 3: hold
All Other Drug-Related Toxicity	3 or Severe	Hold. Dose reduction. Discontinue if not resolved in 4 weeks	Hold. Dose reduction. Discontinue if not resolved in 4 weeks
	4	Discontinue[a]	Discontinue[a]

Toxicity	Grade	NP	Gem
**Hematologic Toxicity**			
Absolute neutrophil count 500 - 999 cells/mm^3		Dose reduction[b]	Dose reduction[b]
Absolute neutrophil count < 500 cells/mm^3		Hold	Hold
Platelets 50,000 – 74,999 cells/mm^3		Dose reduction[b]	Dose reduction[b]
Platelets < 50,000 cells/mm^3		Hold	Hold
Febrile Neutropenia	3-4	Hold. Dose reduction.[b] Discontinue if not resolved in 4 weeks.	1st occurrence: hold. Dose reduction.[b]<br>2nd occurrence: hold. 2 dose reductions.[b]<br>Discontinue if not resolved in 4 weeks

Gem = gemcitabine; NP = nab-paclitaxel
Hold = Hold dosing of investigational product until toxicity resolves to "Continue" or below lowest Grade stated in this table.
Dose reduction = reduce dose by one dose level on following dose
Discontinue = permanently discontinue that investigational product

a Patients may only continue treatment for transient Grade 4 toxicity with approval of the Medical Monitor.

b Reduce current dose. NP and Gem dose can be re-escalated to previous dose level for Grade 3 neutropenia and Grade 2 thrombocytopenia < 1 week

8.5.4 Day 15

Guidelines for management of study treatment-related toxicities on Day 15 are summarized in Table 7.

Table 7 Management of Study Treatment-Related Toxicities on Day 15

Toxicity	Grade	Nivolumab	NP	Gem
**Non-Hematologic Toxicities**				
Diarrhea/colitis	2	1st occurrence: hold<br>2nd occurrence or if not improving in 1 week with oral steroids: discontinue	Continue	Continue
	3	Discontinue	Dose reduction	Dose reduction
	4	Discontinue	Hold. Dose reduction	Hold. Dose reduction
Mucositis	3-4	Hold	Hold. Dose reduction	Hold. Dose reduction
Vomiting	2-3	Hold	Continue. Add antiemetics	Continue. Add antiemetics
	4	Hold. Add antiemetics	Hold. Dose reduction. Add antiemetics	Hold. Dose reduction. Add antiemetics

Toxicity	Grade	Nivolumab	NP	Gem
Increased bilirubin	2	If patient has a normal baseline AST, ALT, hold.<br>If patient's baseline AST, ALT is within Grade 1 toxicity range, continue.	Hold.<br>Discontinue if not resolved in 4 weeks.	Hold.<br>Discontinue if not resolved in 4 weeks.
	3	Hold		
	4	Discontinue	Hold. Dose reduction.<br>Discontinue if not resolved in 4 weeks.	Hold. Dose reduction.<br>Discontinue if not resolved in 4 weeks.
Increased AST, ALT	2	If patient has a normal baseline AST, ALT, hold.<br>If patient's baseline AST, ALT is within Grade 1 toxicity range, continue.	Continue	Continue
	3	Hold	Hold	Hold
	4	Discontinue	Discontinue	Discontinue
Hypophysitis/ Endocrinopathies	2	Hold	Continue	Continue
	3	1st occurrence: hold. Institute endocrine replacement therapy<br>2nd occurrence: discontinue	Continue	Continue
	4	Discontinue	Continue	Continue
Hyperthyroidism	3	1st occurrence: hold<br>2nd occurrence: discontinue	Continue	Continue
	4	Discontinue	Hold	Hold
Rash or other skin toxicity	2	Continue	Dose reduction	Dose reduction
	3	Hold	Dose reduction	Dose reduction
	4	Discontinue	Discontinue	Discontinue
Neurological Toxicity	2	Hold	Dose reduction	Continue
	3-4	Discontinue	Hold. Dose reduction<br>Discontinue if not resolved in 4 weeks	Continue
Nivolumab infusion reaction	2	1st occurrence: continue<br>2nd occurrence: discontinue	Continue	Continue
	3	Discontinue	Hold	Hold
	4	Discontinue	Hold	Hold
Pneumonitis	2	1st occurrence: hold<br>2nd occurrence or if not improving in 2 weeks: discontinue	Hold	Hold

Toxicity	Grade	Nivolumab	NP	Gem
	3-4	Discontinue	Discontinue	Discontinue
Creatinine Elevation	2	Hold<br>If not improving in 1 week: discontinue	Continue	Continue
	3	Hold<br>If not improving in 1 week: discontinue	Hold. Dose reduction. Discontinue if not resolved in 4 weeks.	Hold. Dose reduction. Discontinue if not resolved in 4 weeks
	4	Discontinue	Discontinue	Discontinue
Hypotension, dehydration or suspected adrenal crisis	3	Hold	Hold	Hold
	4	Discontinue	Discontinue	Discontinue
Fatigue	2 (for > 6 weeks) or 3	Grade 2: continue<br>Grade 3: hold	Grade 2: continue<br>Grade 3: hold	Grade 2: continue<br>Grade 3: hold
Uveitis, eye pain, or blurred vision	2-4	Hold<br><br>Discontinue if not improved to grade 1 with topical treatment within 2 weeks or if requires systemic therapy	Hold	Hold
All Other Drug-Related Toxicity[d]	3 or Severe	Hold	Hold. Dose reduction. Discontinue if not resolved in 4 weeks	Hold. Dose reduction. Discontinue if not resolved in 4 weeks
	4	Discontinue	Discontinue[c]	Discontinue[c]
**Hematologic Toxicities**				
If Day 8 doses were reduced or given without modification				
Absolute neutrophil count 500 – 999 cells/mm^3		Continue	Dose reduction	Dose reduction
Absolute neutrophil count < 500 cells/mm^3		Hold	Hold	Hold
Platelets 50,000 – 74,999 cells/mm^3		Continue	Dose reduction	Dose reduction
Platelets < 50,000 cells/mm^3		Hold	Hold	Hold
If Day 8 doses were held				
Absolute neutrophil count ≥ 1000 cells/mm^3		Continue	Dose reduction from Day 1 dose	Dose reduction from Day 1 dose
Absolute neutrophil count 500 - 999 cells/mm^3		Continue	Dose reduction 2 dose levels from Day 1 dose	Dose reduction 2 dose levels from Day 1 dose
Absolute neutrophil count < 500 cells/mm^3		Hold	Hold	Hold
Platelets ≥ 75,000 cells/mm^3		Continue	Dose reduction from Day 1 dose	Dose reduction from Day 1 dose

Toxicity	Grade	Nivolumab	NP	Gem
Platelets 50,000 – 74,999 cells/mm3		Continue	Dose reduction 2 dose levels from Day 1 dose	Dose reduction 2 dose levels from Day 1 dose
Platelets < 50,000 cells/mm3		Hold	Hold	Hold
Febrile Neutropenia	3-4	Hold	Hold. Dose reduction. Discontinue if not resolved in 4 weeks.	1st occurrence: hold. Dose reduction. 2nd occurrence: hold. 2 dose reductions. Discontinue if not resolved in 4 weeks

Gem = gemcitabine; NP = nab-paclitaxel
Hold = Hold dosing of investigational product until toxicity resolves to "Continue" or below lowest Grade stated in this table.
Discontinue = permanently discontinue that investigational product

a If Grade 4 infusion reaction is thought to be related to NP hypersensitivity, nab-paclitaxel- should be permanently discontinued.

b Reduce current dose. NP and gemcitabine dose can be re-escalated to previous dose level for Grade 3 neutropenia and Grade 2 thrombocytopenia < 1 week

c Patients may only continue treatment for transient Grade 4 toxicity with approval of the Medical Monitor.

d Any other Grade 3 nivolumab-related adverse event lasting > 7 days, except endocrinopathies controlled with physiologic hormone replacement or laboratory abnormalities, except as noted above, requires discontinuation of nivolumab.

8.5.5 Criteria for Treatment Continuation within a Cycle

During any cycle, administration of investigational products should be held for toxicities and grades specified in Section 8.5.1 through Section 8.5.4.

Dose of APX005M held on Day 3 can be administered on Day 10 if NP/Gem is given on Day 8 and all APX005M-related toxicities are ≤ Grade 1 excluding:

- Grade 2 neuropathy
- Grade 2 alopecia
- Grade 2 fatigue
- Grade 2 endocrinopathies
- Grade 2 or 3 neutropenia without fever

A dose of NP and/or gemcitabine held on Day 8 can be administered on Day 22 if all chemotherapy-related toxicities are ≤ Grade 1, except that the following are permitted:

- Grade 2 neuropathy
- Grade 2 alopecia
- Grade 2 fatigue
- Grade 2 AST or ALT increase
- Grade 2 endocrinopathies
- Grade 2 neutropenia

8.5.6 APX005M, NP, and Gem Dose Modifications

If, in the opinion of the Investigator, dose reductions are required, they should be instituted as shown in Table 8.

Table 8 APX005M, NP, and Gem Dose Modifications

**APX005M**	
**Current Dose**	**Modified Dose**
0.3 mg/kg	0.2 mg/kg
0.2 mg/kg	0.1 mg/kg
0.1 mg/kg	0.06 mg/kg
0.06 mg/kg	0.03 mg/kg
**NP**	
**Current Dose**	**Modified Dose**
125 mg/m2	100 mg/m2
100 mg/m2	80 mg/m2
80 mg/m2	65 mg/m2
**Gem**	
**Current Dose**	**Modified Dose**
1000 mg/m2	800 mg/m2
800 mg/m2	640 mg/m2

Gem = gemcitabine; NP = nab-paclitaxel

8.6 Study Treatment Assignment

Treatment assignments and randomization will be managed by PICI.

8.6.1 Phase 1b

In Phase 1b, patients will be enrolled into 1 of 4 cohorts (B1, B2, C1, and C2) as summarized in Table 9.

Table 9 Phase 1b Treatment Assignment

**Arm**	**Regimen**	**Number of DLT-Evaluable Patients**
B1	NP/Gem/APX005M 0.1 mg/kg	6
B2	NP/Gem/APX005M 0.3 mg/kg	6
C1	Nivolumab/NP/Gem/APX005M 0.1 mg/kg	6
C2	Nivolumab/NP/Gem/APX005M 0.3 mg/kg	6

DLT = dose-limiting toxicity; Gem = gemcitabine; NP = nab-paclitaxel

8.6.2 Phase 2

A total of approximately 105 patients will be randomized/enrolled in the Phase 2 portion of the study, including 12 patients from Phase 1b (i.e., 6 patients on B2 and 6 patients on C2). Approximately 93 patients will be randomized/enrolled and treated only in Phase 2.

Recruitment is competitive and regulated in the current version of the Cohort Management Plan (a separate document). In step 1 of randomization, 12 of the 93 new patients will be

randomized to the 3 arms in a 4:1:1 ratio in Arms A1, B2, and C2, to achieve balance in the total number of patients enrolled on the arms (since Arm A1 does not accrue in Phase 1b, more patients need to be enrolled in Arm A1). In step 2 of randomization, 81 patients will be randomized to Arms A1, B2, and C2 in a 1:1:1 allocation. The randomization cohorts are outlined in Table 10.

Table 10 Phase 2 Design

Arm	Regimen	Phase 1b	Phase 2		Total
			Step 1	Step 2	
		Number of Patients	Number of Patients	Number of Patients	Number of Patients
A1	Nivolumab/NP/Gem	0	8	27	35
B2	NP/Gem/APX005M 0.3 mg/kg	6	2	27	35
C2	Nivolumab/NP/Gem/APX005M 0.3 mg/kg	6	2	27	35

Gem = gemcitabine; NP = nab-paclitaxel

a If Arm B2 and/or C2 is too toxic in Phase 1b, Arms B1 and C1 (APX005M 0.1 mg/kg) will be used in the Phase 2 portion. The dose of APX005M will remain the same in each arm in Phase 2, so even if B2 is safe and C2 is toxic, B1 and C1 will still be used in the randomization

8.7 Blinding

This is an open-label study with no blinding.

8.8 Treatment Accountability and Compliance

Administration of study drugs will be supervised by study personnel or assigned infusion room nursing staff, who will monitor compliance.

8.9 Prior and Concomitant Illnesses and Medications

8.9.1 Prior and Concomitant Illnesses

Investigators should document all prior significant illnesses. Additional illnesses present at the time when informed consent is given and up to the time of first dosing (Cycle 1 Day 1) are to be regarded as concomitant illnesses. Illnesses first occurring or detected during the study and/or worsening of a concomitant illness during the study are to be documented as AEs on the appropriate eCRF.

8.9.2 Prior and Concomitant Medications

All medications and other treatments taken by the patient during the study, including those treatments initiated prior to the start of the study, must be recorded on the appropriate eCRF. The entry must include the dose, regimen, route, indication, and dates of use.

Medications taken by or administered to the patient for the time period before screening will be recorded on the eCRF.

Patients who are taking the following medications prior to screening must have the minimum washout periods specified below and must not take the medications for the duration of the study.

- Systemic corticosteroids at physiologic dose (equivalent to dose of 10 mg oral prednisone): 14 days before first dose
- Any investigational agent 28 days before first dose

8.9.3 Prohibited Medications

After the baseline visit, medication to treat minor treatment-emergent illness(es) is generally permitted; however, the following therapies are expressly prohibited throughout the study:

- Any other investigational drug, chemotherapy, extensive radiotherapy (involving ≥ 30% of bone marrow) or any other anti-cancer therapy (biologics or other targeted therapy) and anti-neoplastic steroid therapy.
- Immunosuppressive agents (except for patients treated for immune-mediated AE)
- Chronic systemic corticosteroids at physiologic dose (equivalent to dose of 10 mg oral prednisone) 14 days before first dose (except for patients who during the study developed endocrinopathies requiring stable doses of hormone replacement therapy such as hydrocortisone). A temporary course of steroids may be permitted, once discussed and approved by the medical monitor.
    - Transient use of steroids to control contrast agent allergies for radiographic studies are permitted.
- Any live attenuated vaccine therapies used for the prevention of infectious diseases (for up to 30 days prior or after to any dose of study drug).
- Antibodies or immunotherapy directed against interleukins or other cytokines or chemokines.

9 STUDY PROCEDURES

The timing of procedures and assessments to be performed throughout the study is presented in Table 11 and Table 12 (for treatment arms including APX005M) and Table 13 (for treatment arms not including APX005M).

Table 11 Schedule of Assessments (For Arms Including APX005M, Phase Ib)

		Cycle 1					Cycle 2					Cycle 3 and Subsequent Cycles				EOT	Follow-Up[h, s, t]		
**Study Day**	**Screening[a]**	**D1**	**D3[b]**	**D4[b]**	**D8**	**D15**	**D1**	**D3[b]**	**D4[b]**	**D8**	**D15**	**D1[u]**	**D3[b]**	**D8**	**D15**		**D30**	**D100**	**Survival**
Informed Consent	X																		
Inclusion/Exclusion Criteria	X																		
Medical/Disease History	X																		
Pregnancy Test[c]	X	X					X					X					X		
12-Lead Electrocardiogram[d]	X																		
Body Height	X																		
Physical Examination/Performance Status	X	X					X					X				X	X	X	
Serum Chemistry and Hematology[e]	X	X	X	X	X	X	X	X	X	X	X	X	X	X	X	X	X	X	
Concomitant Medications Review	X	X					X					X				X	X	X	
Vital Signs (temperature, blood pressure, respiratory rate, pulse)[f]	X	X	X	X	X	X	X	X	X	X	X	X	X	X	X	X	X	X	
Body Weight	X	X					X					X					X		
Administration of Study Drugs[g]		X	X		X	X	X	X		X	X	X	X	X	X				
Adverse Events Evaluation[h]	X	X	X	X	X	X	X	X	X	X	X	X	X	X	X	X	X	X	
Blood Sampling for PK[i] and ADA[j] Assessment			X	X	X	X		X					X			X			
Blood Sampling for Immune Biomarkers[k]	X[k]	X	X[l]	X[l]	X[l]	X	X					X				X[k]			
Tumor Marker[m]		X																	
Urinalysis		X																	
Disease Assessment[n, o]	X											X *Q8W*				X[p]			
Tumor Biopsy[q]	X								X										
Thyroid Function Testing[r]	X											X				X	X	X	
Follow-up for overall survival and new anti-cancer therapy[t]																X	X	X	X

ADA = anti-drug antibody; AE = adverse event; C = Cycle; CA19-9 = carbohydrate antigen 19-9; CEA = carcinoembryonic antigen; D = Day; EOI = end of infusion; EOT = end of treatment; PK = pharmacokinetics; Q8W = every 8 weeks; RECIST = Response Evaluation Criteria in Solid Tumors.

a Screening evaluations are to be conducted within 28 days before the start of protocol therapy. Some screening procedures may occur on Cycle 1 Day 1 as appropriate.

b Patients receiving APX005M on Day 3 must return to the study site within 24 hours after dose administration (ie, on Day 4) for assessment of AEs, vital signs, and collection of samples for clinical laboratory tests, PK/ADA and translational assessments. If APX005M dosing is delayed from Day 3 to Day 10 due to toxicity, as noted in Section 8.5.5, then the Day 4 assessments must be performed on Day 11.

c Pregnancy test (women of childbearing potential), at Screening and within 24 h before first dose protocol therapy unless screening happens with 24 h of first dose, in which cases testing is not to be done twice. Either serum or urine testing may be used. During the study, monthly pregnancy testing (on Day 1 of each cycle) is required.

d Performed in triplicate. May be repeated if necessary.

e See Table 14 for specific chemistry and hematology laboratory assessments. All laboratory assessments should be reviewed prior to Day 8 of each cycle.

f For Cycles 1-2, on days when APX005M is given, vital signs should be measured pre-infusion, at the end of infusion (EOI), as well as 2 and 5 hours post-EOI. During all other visits, vital signs should be measured pre-infusion. A window of ±10 minutes is permitted.

g Premedication is given 30 minutes before each administration of APX005M and includes a regimen containing an oral H_1 antagonist (e.g., loratadine 10 mg), oral non-steroidal anti-inflammatory- (e.g., ibuprofen 400 mg), acetaminophen 650 mg, and optionally an oral H_2 antagonist (e.g., ranitidine 150-300 mg, famotidine 10-40 mg), or also optionally, ondansetron doses (route of administration, and frequency per institutional standards). A window of -10 minutes is permitted (e.g. premedications may be administered up to 40 minutes, but no later than 30 minutes, prior to APX005M).

h See Section 12 for detailed SAE and AE reporting requirements. The observation period for collection of AEs and AESIs extends from the start of study drug through 100 days after the last dose of study drug or the initiation of new anti-cancer therapy (whichever occurs first). The observation period for collection of SAEs extends from the time the patient signs consent through 100 days after the last dose of study drug or initiated of new anti-cancer therapy (whichever occurs first). After treatment discontinuation, AEs related to study drug are to be followed until resolution or deemed irreversible by Investigator. SAEs related to study drug must be reported at all times, regardless of whether new anti-cancer therapy has been initiated, and followed until resolution or deemed irreversible by the Investigator.

i Blood samples for PK analysis are to be collected in Cycle 1 at pre-dose, EOI, 4 h (after starting infusion), Day 4 (24 h), Day 8, and Day 15. In Cycles 2-4, samples are to be collected at pre-dose and EOI, as described in the Laboratory Manual.

j Blood samples for detection of ADA are to be collected, before APX005M administration, at Day 3 of Cycles 1-4 and at EOT. PK and ADA sampling will occur as a single blood draw, as described in the Laboratory Manual.

k Only to be collected if the patient has given consent to keep tissue and blood biomarker samples for research at the time of screening assessment. If a patient discontinues treatment and begins any new anti-cancer therapy prior to the EOT visit, then EOT blood samples for immune biomarkers will not be collected. For patients that remain on study for 1 year or more, immune biomarkers should be collected at 1 year and then Q6M thereafter, as specified in Section 11.

l Circulating soluble analytes only.

m CA19-9 and CEA will be collected if performed as part of standard of care.

n Per standard of care to provide data for RECIST measurements as appropriate for each patient per the investigator physician. At a minimum at baseline this includes imaging of the chest, abdomen, and pelvis.

o Restaging radiographic studies will be obtained at 8-week intervals after initiating study treatment for the first year, and at 3-month intervals thereafter. A window of -7 days is permitted (see Section 10).

p See Section 10 for description of disease assessment collection after EOT.

q A baseline tumor tissue sample is mandatory for enrollment. Archival tissue must be identified or a fresh tumor biopsy (3 to 4 core needle or excisional biopsies) obtained. Fine needle action is not acceptable. If medically feasible, a mandatory biopsy will also be obtained during Cycle 2 (after second dose APX005M). Otherwise, any on-treatment biopsy will be accepted unless there is no lesion that can be safely biopsied. Additional biopsies may be performed for patients who have prolonged stable disease, defined as more than

two, consecutive disease assessments by RECIST v1.1 and/or if tumor shrinkage is initially demonstrated, followed by a new lesion and/or radiological disease progression. Ad hoc biopsy collection is also permitted with the approval of the medical monitor.

r Thyroid function testing is performed at Screening and on Day 1 of every other cycle starting from Cycle 3.

s Patients should be seen at follow-up visits on Day 30 (± 7 days) and Day 100 (± 14 days) after last dose to be assessed for AEs, regardless of whether new anti-cancer therapy has been initiated.

t Patients are to be followed-up until death or for up to 5 years. After the Day 100 follow-up visit, patients will be contacted by telephone approximately every 3 months to collect survival status and new anti-cancer therapy. In addition, ad hoc collection of survival and new anti-cancer therapy may be requested by the Sponsor.

u After Cycle 2, a window of ± 3 days is permitted at Day 1 for all subsequent cycles. Should Day 1 be moved, all subsequent cycle visits need to be moved, as well (e.g., if Day 1 is delayed 3 days, Days 3, 8 and 15 would be delayed 3 days, as well).

Table 12 Schedule of Assessments (For Arms Including APX005M, Phase 2)

		Cycle 1					Cycle 2					Cycle 3 and Subsequent Cycles					Follow-Up[i,v,w]		
**Study Day**	**Screening[a]**	**D1**	**D3[b]**	**D4[b]**	**D8**	**D15**	**D1**	**D3[b]**	**D4[b]**	**D8**	**D15**	**D1[x]**	**D3[b]**	**D8**	**D15**	**EOT**	**D30**	**D100**	**Survival**
Informed Consent	X																		
Inclusion/Exclusion Criteria	X																		
Medical/Disease History	X																		
Pregnancy Test[c]	X	X					X					X					X		
12-Lead Electrocardiogram[d]	X																		
Body Height	X																		
Physical Examination/ Performance Status	X	X					X					X				X	X	X	
Serum Chemistry and Hematology[e]	X	X	X	X	X	X	X	X	X	X	X	X	X	X	X	X	X	X	
Concomitant Medications Review	X	X					X					X				X	X	X	
Vital Signs (temperature, blood pressure, respiratory rate, pulse)[f]	X	X	X	X	X	X	X	X	X	X	X	X	X	X	X	X	X	X	
Body Weight	X	X					X					X					X		
Administration of Study Drugs[g, h]		X	X		X	X	X	X		X	X	X	X	X	X				
Adverse Events Evaluation[i]	X	X	X	X	X	X	X	X	X	X	X	X	X	X	X	X	X	X	
Blood Sampling for PK[j] and ADA[k] Assessment			X					X					X			X			
Blood Sampling for Immune Biomarkers[l]	X[m]	X	X[n]			X	X					X[l]				X[l]			
Stool Sample for Microbiome Profile [o]	X						X												
Tumor Marker[p]		X																	
Urinalysis		X																	
Disease Assessment[q, r]	X											X *Q8W*				X[s]			
Tumor Biopsy[t]	X								X							X (optional)			
Thyroid Function Testing[u]	X											X				X	X	X	
Follow-up for overall survival and new anti-cancer therapy[w]																X	X	X	X

ADA = anti-drug antibody; AE = adverse event; C = Cycle; CA19-9 = carbohydrate antigen 19-9; CEA = carcinoembryonic antigen; D = Day; EOI = end of infusion; EOT = end of treatment; PK = pharmacokinetics; Q8W = every 8 weeks; RECIST = Response Evaluation Criteria in Solid Tumors.

a Screening evaluations are to be conducted within 28 days before the start of protocol therapy. Some screening procedures may occur on Cycle 1 Day 1 as appropriate.

b For patients receiving APX005M, the Day 4 visit (or Day 11 visit if dosing is delayed) is optional after Cycle 2.

c Pregnancy test (women of childbearing potential), at Screening and within 24 h before first dose protocol therapy unless screening happens with 24 h of first dose, in which cases testing is not to be done twice. Either serum or urine testing may be used. During the study, monthly pregnancy testing (on Day 1 of each cycle) is required.

d Performed in triplicate. May be repeated if necessary.

e See Table 14 for specific chemistry and hematology laboratory assessments. All laboratory assessments should be reviewed prior to Day 8 of each cycle.

f For Cycles 1-2, on days when APX005M is given, vital signs should be measured pre-infusion, at the end of infusion (EOI), as well as 2 and 5 hours post-EOI. During all other visits, vital signs should be measured pre-infusion. A window of ±10 minutes is permitted.

g Patients must receive the first dose of study drug within 3 days of randomization.

h Premedication is given 30 minutes before each administration of APX005M and includes a regimen containing an oral H_1 antagonist (e.g., loratadine 10 mg), oral non-steroidal anti-inflammatory- (e.g., ibuprofen 400 mg), acetaminophen 650 mg, and optionally an oral H_2 antagonist (e.g., ranitidine 150-300 mg, famotidine 10-40 mg), or also optionally, ondansetron doses (route of administration, and frequency per institutional standards). A window of -10 minutes is permitted (e.g. premedications may be administered up to 40 minutes, but no later than 30 minutes, prior to APX005M).

i See Section 12 for detailed SAE and AE reporting requirements. The observation period for collection of AEs and AESIs extends from the start of study drug through 100 days after the last dose of study drug or the initiation of new anti-cancer therapy (whichever occurs first). The observation period for collection of SAEs extends from the time the patient signs consent through 100 days after the last dose of study drug or initiated of new anti-cancer therapy (whichever occurs first). After treatment discontinuation, AEs related to study drug are to be followed until resolution or deemed irreversible by Investigator. SAEs related to study drug must be reported at all times, regardless of whether new anti-cancer therapy has been initiated, and followed until resolution or deemed irreversible by the Investigator.

j Blood samples for PK analysis are to be collected at pre-dose and EOI, in Cycles 1 to 4, as described in the Laboratory Manual.

k Blood samples for detection of ADA are to be collected, before APX005M administration, at Day 3 of Cycles 1-4 and at EOT. PK and ADA sampling will occur as a single blood draw, as described in the Laboratory Manual.

l Immune biomarker collections through Cycle 4 and at EOT. If a patient discontinues treatment and begins any new anti-cancer therapy prior to the EOT visit, then EOT blood samples for immune biomarkers will not be collected. For patients that remain on study for 1 year or more, immune biomarkers should be collected at 1 year and then Q6M thereafter, as specified in Section 11.

m Only to be collected if the patient has given consent to keep tissue and blood biomarker samples for research at the time of screening assessment.

n Circulating soluble analytes only.

o Stool samples will be collected for microbiome profiling at screening (prior to C1D1) and, if possible, during Cycle 2. Otherwise, any on-treatment stool collection will be accepted.

p CA19-9 and CEA are required at Cycle 1 Day 1 and will continue to be collected any time on study if performed as part of standard of care.

q Per standard of care to provide data for RECIST measurements as appropriate for each patient per the investigator physician. At a minimum at baseline this includes imaging of the chest, abdomen, and pelvis.

r Restaging radiographic studies will be obtained at 8-week intervals after initiating study treatment for the first year, and at 3-month intervals thereafter. A window of -7 days is permitted (see Section 10).

s See Section 10 for description of disease assessment collection after EOT.

t A baseline tumor tissue sample is mandatory for enrollment. Archival tissue must be identified or a fresh tumor biopsy (3 to 4 core needle or excisional biopsies) obtained. Fine needle aspiration is not acceptable. If medically feasible, a mandatory biopsy will also be obtained during Cycle 2 (after second dose APX005M). Otherwise, any on-treatment biopsy will be accepted unless there is no lesion that can be safely biopsied. Additional biopsies may be performed for patients who have prolonged stable disease, defined as more

than two, consecutive disease assessments by RECIST v1.1 and/or if tumor shrinkage is initially demonstrated, followed by a new lesion and/or radiological disease progression. Ad hoc biopsy collection is also permitted with the approval of the medical monitor.

u Thyroid function testing is performed at Screening and on Day 1 of every other cycle starting from Cycle 3.

v Patients should be seen at follow-up visits on Day 30 (± 7 days) and Day 100 (± 14 days) after last dose to be assessed for AEs, regardless of whether new anti-cancer therapy has been initiated.

w Patients are to be followed-up until death or for up to 5 years. After the Day 100 follow-up visit, patients will be contacted by telephone approximately every 3 months to collect survival status and new anti-cancer therapy. In addition, ad hoc collection of survival and new anti-cancer therapy may be requested by the Sponsor.

x After Cycle 2, a window of ± 3 days is permitted at Day 1 for all subsequent cycles. Should Day 1 be moved, all subsequent cycle visits need to be moved, as well (e.g., if Day 1 is delayed 3 days, Days 3, 8 and 15 would be delayed 3 days, as well).

Table 13 Schedule of Assessments (For Arms Not Including APX005M, Phase 2)

		Cycle 1			Cycle 2			Cycle 3 and Subsequent Cycles				Follow-Up[h, r, s]		
**Study Day**	**Screening[a]**	**D1**	**D8**	**D15**	**D1**	**D8**	**D15**	**D1[t]**	**D8**	**D15**	**EOT**	**D30**	**D100**	**Survival**
Informed Consent	X													
Inclusion/Exclusion Criteria	X													
Medical/Disease History	X													
Pregnancy Test[b]	X	X			X			X				X		
12-Lead Electrocardiogram[c]	X													
Body Height	X													
Physical Examination/Performance Status	X	X			X			X			X	X	X	
Serum Chemistry and Hematology[d]	X	X	X	X	X	X	X	X	X	X	X	X	X	
Concomitant Medications Review	X	X			X			X			X	X	X	
Vital Signs (temperature, blood pressure, respiratory rate, pulse)[e]	X	X	X	X	X	X	X	X	X	X	X	X	X	
Body Weight	X	X			X			X				X		
Administration of Study Drugs[f, g]		X	X	X	X	X	X	X	X	X				
Adverse Events Evaluation[h]	X	X	X	X	X	X	X	X	X	X	X	X	X	
Blood Sampling for Immune Biomarkers[i]	X[j]	X		X	X			X[i]			X[i]			
Stool for Microbiome Profiling[k]	X				X									
Tumor Marker[l]		X												
Urinalysis		X												
Disease Assessment[m, n]	X							X *Q8W*			X[o]			
Tumor Biopsy[p]	X					X					X (optional)			
Thyroid Function Testing[q]	X							X			X	X	X	
Follow-up for overall survival and new anticancer therapy[s]											X	X	X	X

AE = adverse event; C = Cycle; CA19-9 = carbohydrate antigen 19-9; CEA = carcinoembryonic antigen; D = Day; EOT = end of treatment; Q8W = every 8 weeks; RECIST = Response Evaluation Criteria in Solid Tumors.

a Screening evaluations are to be conducted within 28 days before the start of protocol therapy. Some screening procedures may occur on Cycle 1 Day 1 as appropriate.

b Pregnancy test (women of childbearing potential), at Screening and within 24 h before first dose protocol therapy unless screening happens with 24 h of first dose, in which cases testing is not to be done twice. Either serum or urine testing may be used. During the study, monthly pregnancy testing (on Day 1 of each cycle) is required.

c Performed in triplicate. May be repeated if necessary.

d See Table 14 for specific chemistry and hematology laboratory assessments.

e Vital signs should be measured pre-infusion.

f After Cycle 2, a window of ± 3 days after end of previous cycle is permitted for Day 1 of all subsequent cycles.

g Patients must receive the first dose of study drug within 3 days of randomization.

h See Section 12 for detailed SAE and AE reporting requirements. The observation period for collection of AEs and AESIs extends from the start of study drug through 100 days after the last dose of study drug or the initiation of new anti-cancer therapy (whichever occurs first). The observation period for collection of SAEs extends from the time the patient signs consent through 100 days after the last dose of study drug or initiated of new anti-cancer therapy (whichever occurs first). After treatment discontinuation, AEs related to study drug are to be followed until resolution or deemed irreversible by Investigator. SAEs related to study drug must be reported at all times, regardless of whether new anti-cancer therapy has been initiated, and followed until resolution or deemed irreversible by the Investigator.

i Immune biomarker collections through Cycle 4 and at EOT. If a patient discontinues treatment and begins any new anti-cancer therapy prior to the EOT visit, then EOT blood samples for immune biomarkers will not be collected. For patients that remain on study for 1 year or more, immune biomarkers should be collected at 1 year and then Q6M thereafter, as specified in Section 11.

j Only to be collected if the patient has given consent to keep tissue and blood biomarker samples for research at the time of screening assessment.

k In Phase 2, Stool samples will be collected for microbiome profiling at screening (prior to C1D1) and, if possible, during Cycle 2. Otherwise, any on treatment stool collection will be accepted.

l CA19-9 and CEA are required at Cycle 1 Day 1 and will continue to be collected any time on study if performed as part of standard of care.

m Per standard of care to provide data for RECIST measurements as appropriate for each patient per the investigator physician. At a minimum at baseline this includes imaging of the chest, abdomen, and pelvis.

n Restaging radiographic studies will be obtained at 8-week intervals after initiating study treatment for the first year, and at 3-month intervals thereafter. A window of -7 days is permitted (see Section 10).

o See Section 10 for description of disease assessment collection after EOT.

p A baseline tumor tissue sample is mandatory for enrollment. Archival tissue must be identified or a fresh tumor biopsy (3 to 4 core needle or excisional biopsies) obtained. Fine needle aspiration is not acceptable. If medically feasible, a mandatory biopsy will also be obtained during Cycle 2 (after third dose nivolumab). Otherwise, any on-treatment biopsy will be accepted unless there is no lesion that can be safely biopsied. Additional biopsies may be performed for patients who have prolonged stable disease, defined as more than two, consecutive disease assessments by RECIST v1.1 and/or if tumor shrinkage is initially demonstrated, followed by a new lesion and/or radiological disease progression. Ad hoc biopsy collection is also permitted with the approval of the medical monitor.

q Thyroid function testing is performed at Screening and on Day 1 of every other cycle starting from Cycle 3.

r Patients should be seen at follow-up visits on Day 30 (± 7 days) and Day 100 (± 14 days) after last dose to be assessed for AEs, regardless of whether new anti-cancer therapy has been initiated.

s Patients are to be followed-up until death or for up to 5 years. After the Day 100 follow-up visit, patients will be contacted by telephone approximately every 3 months to collect survival status and new anti-cancer therapy. In addition, ad hoc collection of survival and new anti-cancer therapy may be requested by the Sponsor.

t After Cycle 2, a window of ± 3 days is permitted at Day 1 for all subsequent cycles. Should Day 1 be moved, all subsequent cycle visits need to be moved, as well (e.g., if Day 1 is delayed 3 days, Days 3, 8 and 15 would be delayed 3 days, as well).

Timing of Study Assessments for Treatment Delays

Study assessments must be completed on the day the patient receives treatment. If treatment is delayed, the study assessments will be postponed. Only clinical laboratory assessments that were not completed, or were clinically relevant on the missed treatment day, are required be performed/repeated on the day treatment resumes.

APX005M Dose Delays: Patients in Phase 1b receiving APX005M on Day 3 must return to the study site within 24 hours after dose administration (ie, on Day 4) for assessment of AEs, vital signs, and collection of samples for clinical laboratory tests, PK/ADA and translational assessments. If APX005M dosing is delayed from Day 3 to Day 10 due to toxicity, as noted in Section 8.5.5, then the Day 4 assessments must be performed on Day 11. For patients receiving APX005M in Phase 2, the Day 4 visit (or Day 11 visit if dosing is delayed) is optional after Cycle 2.

10 EFFICACY ASSESSMENTS

Participants will undergo tumor assessments as described in the Schedule of Assessments (see Section 9):

- Disease assessments are performed to provide data for RECIST. At a minimum, at baseline, this should include imaging of the chest, abdomen, and pelvis.
- Throughout the study, restaging radiographic studies will be obtained at 8-week intervals after initiating study treatment for the first year, and at 3-month intervals thereafter. A window of ±7 days is permitted.

Disease assessments performed as part of standard of care will continue to be collected after treatment discontinuation. The following criteria serve to provide additional guidelines around when disease assessments (i.e., RECIST measurements) should be collected after a patient has discontinued study treatment, and if treatment is continued beyond radiological progression:

- If a patient discontinues treatment due to radiographic disease progression (PD), as defined by RECIST v1.1, then no additional disease assessments are collected. These patients should be followed for survival status until death, withdrawal of consent, or study termination. Safety follow-up should continue as described in the Schedule of Assessments (Section 9).
- If a patient has elected to continue treatment after having demonstrated PD per RECIST v1.1 criteria, disease assessments should continue to be collected as described in the Schedule of Assessments (Q8W; Section 9).
- If a patient discontinues treatment due to symptomatic deterioration, every attempt should be made to continue collecting standard of care disease assessments:
    - If not medically feasible, clinical progression needs to be documented as the reason for treatment discontinuation.
    - If medically feasible, standard of care disease assessments should continue to be collected until the patient has demonstrated PD per RECIST v1.1 criteria.
    - In either scenario, these patients should be followed for survival status until death, withdrawal of consent, or study termination. Safety follow up should continue as described in the Schedule of Assessments (Section 9).
- If a patient discontinues treatment for any other reason (e.g. toxicity, protocol deviation), standard of care disease assessments should continue to be collected until the patient has demonstrated PD per RECIST v1.1 criteria. These patients should be followed for survival status until death, withdrawal of consent, or study termination. Safety follow up should continue as described in the Schedule of Assessments (Section 9).
- If a patient initiates new anti-cancer therapy, collection of disease assessments is no longer required. These patients should be followed for survival status until death, withdrawal of consent, or study termination. Safety follow up should continue as described in the Schedule of Assessments (Section 9).

- If a patient discontinues treatment and withdraws consent, no additional assessments or follow-up should be performed.

10.1 Response Criteria

Response and progression will be evaluated in this study using the international criteria proposed by the RECIST Version 1.1 Committee. Changes in only the largest diameter (unidimensional measurement) of the tumor lesions are used in the RECIST criteria.

Note: Lesions are either measurable or non-measurable using the criteria provided below. The term "evaluable" in reference to measurability will not be used because it does not provide additional meaning or accuracy. The irradiated index lesion is not included in the RECIST determination; however, tumor response of this lesion will be assessed and tabulated separately using criteria from RECIST.

10.1.1 Definitions of Measurable and Non-Measurable Disease

Measurable disease is defined as at least 1 lesion whose longest diameter can be accurately measured as ≥ 2.0 cm with conventional techniques or as ≥ 1.0 cm with spiral computed tomography (CT) scan. Clinical lesions will only be considered measurable when they are superficial (e.g., skin nodules, palpable lymph nodes). Lesions on chest x-ray are acceptable as measurable lesions when they are clearly defined and surrounded by aerated lung. However, CT is preferable.

Lymph nodes with a short axis of > 15 mm by CT are considered measurable and assessable as target lesions. Only the short axis measurement should be included in the sum of lesions in calculation of tumor response. Nodes that shrink to < 10 mm short axis are considered normal.

All other lesions (or sites of disease), including small lesions (longest diameter < 2.0 cm with conventional techniques or as < 1.0 cm with spiral CT) are considered non-measurable disease. Bone lesions, leptomeningeal disease, ascites, pleural/pericardial effusions, lymphangitis cutis/pulmonis, abdominal masses (not followed by CT or magnetic resonance imaging [MRI]), and cystic lesions are all non-measurable.

10.1.2 Guidelines for Evaluation of Measurable Disease

Assessment of measurable disease will be performed locally at each study site.

Measurement Methods: The same method of assessment and the same technique should be used to characterize each identified and reported lesion at baseline and during follow-up. For patients having only lesions measuring at least 1 cm to < 2 cm, spiral CT imaging must be used for both pre- and post-treatment tumor assessments. Imaging-based evaluation is preferred to evaluation by clinical examination when both methods have been used at the same evaluation to assess the antitumor effect of a treatment.

New lesions on the basis of positron emission tomography (PET)/CT using fluorodeoxyglucose (FDG)-PET/CT imaging can be identified according to the following:

- Negative FDG-PET/CT at baseline, with a positive FDG-PET/CT at follow-up is a sign of PD based on a new lesion.
- No FDG-PET/CT at baseline and a positive FDG-PET/CT at follow up:

- If the positive FDG-PET/CT at follow-up corresponds to a new site of disease confirmed by CT, this is PD.

If the positive FDG-PET/CT at follow-up is not confirmed as a new site of disease on CT, additional follow-up CT scans are needed to determine if there is truly progression occurring at that site (if so, the date of PD will be the date of the initial abnormal FDG-PET/CT scan).

If the positive FDG-PET/CT at follow-up corresponds to a pre-existing site of disease on CT that is not progressing on the basis of the anatomic images, this is not PD. A “positive” FDG-PET/CT scan lesion means one that is visually FDG avid relative to the background tissue on the attenuation corrected image.

10.1.3 Measurement of Effect

10.1.3.1 Target Lesions

All measurable lesions up to a maximum of 5 lesions representative of all involved organs should be identified as target lesions and recorded and measured at baseline. If the protocol specified studies are performed, and there are fewer than 5 lesions identified (as there often will be), there is no reason to perform additional studies beyond those specified in the protocol to discover new lesions. For any 1 organ, no more than 2 lesions need to be measured. Target lesions should be selected on the basis of their size (lesions with the longest diameter) and their suitability for accurate repetitive measurements (either by imaging techniques or clinically).

A sum of the longest diameter (LD) for all target lesions will be calculated and reported as the baseline sum LD. The baseline sum LD will be used as reference to further characterize the objective tumor response of the measurable dimension of the disease.

10.1.3.2 Non-Target Lesions

All other lesions (or sites of disease) should be identified as non-target lesions and should also be recorded at baseline. Measurements are not required, and these lesions should be followed in accord with the following section.

10.1.3.3 Response Criteria

All identified sites of disease must be followed on re-evaluation. Specifically, a change in objective status to either a PR or CR cannot be done without rechecking all identified sites (i.e., target and non-target lesions) of pre-existing disease.

10.1.3.4 Evaluation of target lesions

- CR: Disappearance of all target lesions
- PR: At least a 30% decrease in the sum of the LD of target lesions, taking as reference the baseline sum LD
- PD: At least a 20% increase in the sum of diameters of target lesions, taking as reference the smallest sum on study (this includes the baseline sum if that is the smallest on study). In addition to the relative increase of 20%, the sum must also demonstrate an absolute increase of at least 5 mm.
  (Note: the appearance of one or more new lesions at the end of cycle scan is also considered progression).

- SD: Neither sufficient shrinkage to qualify for PR nor sufficient increase to qualify for PD, taking as reference the smallest sum LD since the treatment started

10.1.3.5 Evaluation of non-target lesions

- CR: Disappearance of all non-target lesions
- Non-CR/Non-PR: Persistence of one or more non-target lesion without evidence of unequivocal progression of the non-target lesions or appearance of new lesions
- PD: Appearance of one or more new lesions and/or unequivocal progression of existing non-target lesions

Although a clear progression of "non-target" lesions only is exceptional, in such circumstances the opinion of the treating physician should prevail, and the progression status should be confirmed at a later time by the PI.

10.1.3.6 Overall Objective Status

The overall OR status for an evaluation is determined by combining the patient's status on target lesions, non-target lesions, and new disease.

Symptomatic deterioration: Patients with global deterioration of health status requiring discontinuation of treatment without objective evidence of disease progression at that time, and not either related to study treatment or other medical conditions, should be reported as PD due to "symptomatic deterioration." Every effort should be made to document the objective progression even after discontinuation of treatment due to symptomatic deterioration that may include weight loss > 10% of body weight, worsening of tumor-related symptoms, and/or decline in performance status of > 1 level on ECOG scale.

11 PHARMACOKINETICS AND PHARMACODYNAMICS

11.1 Pharmacokinetic Sampling

PK analysis will involve determination of circulating concentrations of APX005M at times specified in Table 11 and Table 12.

11.1.1 Blood Samples

Blood samples for PK analysis of APX005M levels will be collected as follows:

Phase 1b: PK in Cycle 1 at pre-dose, end of infusion (EOI), 4 h (after starting infusion), 24 h, Day 8, and Day 15. Cycles 2-4: pre-dose, EOI and EOT.

Phase 2: Pre-dose and EOI samples in Cycles 1-4 and EOT.

For patients who begin a new anti-cancer therapy prior to their EOT visit, an EOT PK blood draw is not required.

Samples will be collected at the time points indicated in the Schedule of Assessments (Table 11 and Table 12). The actual date and time of each blood sample collection will be recorded. The timing of PK samples may be altered and/or PK samples may be obtained at additional time points to ensure thorough PK monitoring.

Details of PK blood sample collection, processing, storage, and shipping procedures are provided in a separate Laboratory Manual.

11.1.2 Analytical Methodology

The concentration of APX005M will be determined from the samples using a validated analytical method. See Section 13.5 for a list of PK parameters that will be calculated.

11.2 Immune Endpoints

Specimen Collection: Tumor tissue (archival, fresh core or excisional tumor biopsies) must be available or collected prior to the start of treatment. If medically feasible, a mandatory biopsy will also be obtained during Cycle 2. For arms including APX005M, the biopsy will take place after the second dose of APX005M and, for arms not including APX005M, after the third dose of nivolumab. Otherwise, any on-treatment biopsy will be accepted unless there is no lesion that can be safely biopsied. Additional biopsies may be performed for patients who have prolonged stable disease, defined as stable for more than two, consecutive disease assessments by RECIST v1.1, and/or if tumor shrinkage is demonstrated, followed by a new lesion and/or radiological disease progression Ad hoc collections may also be performed with Medical Monitor approval.

Blood for plasma, serum, circulating tumor DNA and PBMC will be collectedly at baseline and serially during therapy as noted above. Tumor samples and blood samples will be processed according to the study Laboratory Manual.

Blood samples will be collected for immune biomarkers at Cycle 1 pre-dose on Day 3, Day 4 (24 hours after start of APX005M infusion), and Day 8 during Phase 1b. For Phase 2, blood samples will be collected as outlined in the schedule of assessments at screening, through Cycle 4 and EOT. Ad hoc collections may also be performed with Medical Monitor approval.

For any patient who remains on study for 1 year, immune biomarkers may also be collected at 1 year and then every 6 months (12, 18, 24, 30, etc. months).

Blood samples for detection of ADA are to be collected before APX005M administration in Cycles 1-4, and at the EOT.

Stool samples will be collected at screening (prior to Cycle 1, Day 1) and during Cycle 2, if possible. Otherwise, any on-treatment stool sample will be accepted.

11.2.1 Immune Biomarkers

Detailed sample collection, processing, storage, and shipping procedures are provided in a separate Laboratory Manual. Depending on sample availability, a battery of immune assays is planned, that may include but will not necessarily limited to the following:

Analysis of Myeloid and B Cell Activation: Using un-manipulated peripheral blood, monocytes, B cells, dendritic cells before and after treatment can be analyzed using flow cytometry to measure cell surface immune markers using a panel of immune parameters such as CD11b, CD19, CD123, CD11c (to define the subsets) and CD86, MHC class I and II, CD70, and CD54 (to measure activation). For each parameter and each cell type, the percentage of cells positive for the marker and/or mean fluorescence intensity (MFI) at time points after treated can be compared to baseline and the change are calculated as $\%_{after}/\%_{baseline}$ or $MFI_{after}/MFI_{baseline}$.

Tissue Assessment: Tissue can be analyzed by hematoxylin and eosin staining and by immunohistochemistry for PD-L1 and for immune markers (such as CD45, CD68, CD3, CD8, CD4, Foxp3, CD20, myeloperoxidase), tumor markers (AFP, Ki-67, cleaved caspase 3), vascular (CD31) and stromal markers (collagen type I); and by Masson's trichrome. The tumor may also be assessed by a mutational panel and if sufficient material is available, tumor whole exome sequencing (WES) and RNA sequencing may be performed. If RNA quality is insufficient, Nanostring technology or equivalent for immune activation gene expression may be performed instead of RNA-Seq or in addition. From germline WES from PBMC, HLA type can be determined. From tumor WES, tumor RNA-Seq, and germline WES, patient-specific neo epitopes arising from tumor somatic missense mutations can be predicted bioinformatically.

Analysis of T-cell Activation: Together with complete blood count differentials, multiplex flow cytometry analysis of PBMC can be used to measure both the percentages and absolute count (cells/mm^3) of important T-cell subsets defined by immunophenotyping, such as total CD3+ cells, CD3+ CD8+ T cells, CD3+ CD4+ T cells, and CD3+ CD4+ Foxp3+ regulatory T cells. For each subset, differentiation status (e.g., naïve, central memory, effector memory) or activation vs. exhaustion status can be assessed using additional markers such as Eomes, Tbet, Granzyme B, Ki-67, CD45RA, ICOS, CD45RO, CCR7, CD28, CD27, CD57, CD25, CD69, HLA-DR, CTLA4, and PD-1. When possible, trends may be tracked in T-cell subsets based on analysis of multiple post-treatment samples. NK cells subsets may also be assessed using CD16 and CD56, with CD69 as an activation marker.

An analysis of PBMC may be additionally performed using CyTOF technology for deeper analysis of immune subsets and activation status in peripheral blood.

Immune activation may be additionally assessed using RNA-Seq of PBMC.

Inflammatory Cytokines/Chemokines: Blood may be used to determine concentrations of cytokines and other circulating factors that may include but not limited to transforming growth factor (TGF)-β, IL-1, TNF-α, IL-6 and others using a multiplex platform or other standard analytical methods.

T-cell Receptor (TCR) Deep Sequencing: DNA isolated from PBMC (as well as from paraffin-embedded tissue) can be analyzed by deep sequencing to detect and track specific TCR clones. This technique permits assessment of specific adaptive immune response independent of having to know the particular relevant tumor antigen, which of course may vary patient to patient. Comparison of TCR beta sequence data in serial samples from blood and tumor can demonstrate de novo evolution of an anti-tumor T cell repertoire.

Circulating tumor material: Blood samples may be tested for circulating biomarkers including cell-free DNA and tumor cells.

Stool: Stool samples may be tested for microbiome profile using sequencing or metabolomic methods.

Additional Research: Beyond the assays noted above, and provided that sample material is left over, and with approval of the overall PI and sponsor, investigators/sponsor may perform additional research assays on tumor or blood samples collected in this protocol. Future Biomedical Research may be conducted on specimens consented for future biomedical research during this clinical trial. This research may include genetic analyses (DNA), gene expression profiling (RNA), proteomics, metabolomics (serum, plasma) and/or the measurement of other analytes, depending on which specimens are consented for future biomedical research.

Such research is for biomarker testing to address emergent questions not described elsewhere in the protocol (as part of the main trial) and will only be conducted on specimens from appropriately consented participants. The objective of collecting/retaining specimens for Future Biomedical Research is to explore and identify biomarkers that inform the scientific understanding of disease and/or their therapeutic treatments. The overarching goal is to use such information to understand disease, safety and potential treatments for future patients.

11.2.3 Anti-drug antibodies

Pre-APX005M samples for PK/ADA are planned for ADA analysis, as well as at EOT.

12 SAFETY ASSESSMENTS

Safety assessments (vital signs, physical examinations, ECG recording, AEs, clinical laboratory results (routine hematology and biochemistry) are to be performed at protocol-specified visits, as specified in the Schedule of Assessments (Table 11, Table 12, and Table 13).

12.1 Vital Signs

Vital signs (body temperature, heart rate, respiratory rate, systolic and diastolic blood pressure measurements) will be evaluated at the visits indicated in the Schedule of Assessments (Table 11, Table 12, and Table 13). Blood pressure measurements are to be taken in the same arm for the duration of the study. Body weight (without shoes) will be recorded whenever vital signs are recorded, and height (without shoes) will be recorded at Screening only.

Vital sign measurements will be repeated if clinically significant or machine/equipment errors occur. Out-of-range blood pressure, respiratory rate or heart rate measurements will be repeated at the Investigator's discretion. Any confirmed, clinically significant vital sign measurements must be recorded as AEs.

12.2 Physical Examination

A complete physical examination will be performed at Screening Visit 1, according to institutional standards. Physical examinations will be performed by a physician or designated nurse practitioner or physician's assistant. In addition, medical history will be recorded at Screening, including smoking history, if applicable.

A limited physical examination to verify continued patient eligibility and to follow up any change in medical history will be performed at the visits indicated in the Schedule of Assessments (Table 11, Table 12, and Table 13). Symptom-driven limited physical examinations will be performed as clinically indicated at any study visit. All changes not present at baseline or described in the past medical history and identified as clinically noteworthy must be recorded as AEs.

12.3 Electrocardiogram

A 12-lead resting ECG will be obtained at the visits indicated in the Schedule of Assessments (Table 11, Table 12, and Table 13).

At Screening, the Investigator will examine the ECG traces for signs of cardiac disease that could exclude the patient from the study. An assessment of normal or abnormal will be recorded and if the ECG is considered abnormal, the abnormality will be documented on the CRF. ECGs will be repeated if clinically significant abnormalities are observed or artifacts are present.

12.4 Laboratory Assessments

Samples for laboratory assessments (listed in Table 14) are to be obtained at designated visits as detailed in the Schedule of Assessments (Table 11 and Table 12 for arms including APX005M and Table 13 for arms not including APX005M).

All laboratory reports must be reviewed, signed, and dated by the Investigator. A legible copy of all reports must be filed with both the patient's CRF and medical record (source document) for that visit. Any laboratory test result considered by the Investigator to be clinically significant should be considered an AE (clinically significant AEs include those that require an intervention). Clinically significant abnormal values occurring during the study will be followed until repeat test results return to normal/baseline, stabilize, or are no longer clinically significant.

Table 14 Laboratory Assessments

Hematology	Chemistry	Urine	Thyroid Function Tests
Complete Blood Count (CBC) with differential, including:<br>Hematocrit (Hct)<br>Hemoglobin (Hb)<br>Platelet Count<br><br>White Blood Cell (WBC) Count with differential, including:<br>Eosinophils<br>Lymphocytes<br>Neutrophils	Albumin<br>Alkaline Phosphate<br>Alanine Aminotransferase (ALT)<br>Alanine Aminotransferase (AST)<br>Blood Urea Nitrogen (BUN)<br>Carbon Dioxide (CO_2)<br>Creatinine<br>Electrolytes (Na, K, Cl)<br>Total Bilirubin<br>Direct Bilirubin	Standard analysis; can be dipstick	Thyroid stimulating hormone (TSH)<br>Triiodothyronine (T3)Free Triiodothyronine (FT3)<br>Free thyroxine (FT4)
**Pregnancy test**: A pregnancy test will be performed on all female subjects of child-bearing potential at Screening as noted above. Either serum or urine test is permitted.			

12.5 Adverse Events, Adverse Events of Special Interest, and Serious Adverse Events

12.5.1 Time Period and Frequency for Collecting Adverse Events, Serious Adverse Events, and Other Reportable Safety Event Information

All AEs will be collected from the start of study drug through 100 days after the last dose of study drug, or until initiation of a new systemic anti-cancer therapy (whichever occurs first). All treatment-related AEs ongoing at the time of treatment discontinuation are to be followed until resolution, or deemed irreversible by the Investigator, regardless of whether new anti-cancer therapy has been initiated.

All SAEs, will be collected from the time the patient signs informed consent through 100 days after the last dose of study drug, or until initiation of a new systemic anti-cancer therapy (whichever occurs first). SAEs related to study drug must be reported at all times, regardless of whether new anti-cancer therapy has been initiated. All treatment-related SAEs must also to be followed until resolution, or deemed irreversible by the Investigator, regardless of whether new anti-cancer therapy has been initiated.

Prior to initiation of study drug, only SAEs that are related to a protocol-mandated intervention, including those that occur prior to the assignment of study procedures (eg, screening invasive procedures, such as biopsies) should be reported. After obtaining informed consent, but prior to initiation of study drug, other medical occurrences will be recorded as medical history.

If the Investigator learns of any SAE, including a death, at any time after the end of the AE reporting period, and he/she considers the event to be reasonably related to the study drug or study participation, the Investigator must promptly notify the Sponsor or its designee. The Investigator should report these events directly to the Sponsor or its designee, either by faxing or emailing the study-specific Serious Adverse Event Report Form (SAERF).

12.5.2 Definition of Adverse Events

An AE is any untoward medical occurrence in a patient, temporally associated with the use of study drug, whether or not considered related to the study drug.

An AE can be any unfavorable and unintended sign (including an abnormal laboratory finding), symptom, or disease (new or exacerbated) temporally associated with the use of a study drug. Investigators will seek information on AE at each patient contact. AEs reported by the patient (or, when appropriate, by a caregiver, surrogate, or the patient's legally authorized representative) or noted by study personnel will be recorded in the patient's medical record and on the Adverse Event eCRF page.

The Investigator and any qualified designees are responsible for detecting, documenting, and recording events that meet the definition of an AE or SAE and remain responsible for following up AEs that are serious, considered related to the study drug or study procedures, or that caused the patient to discontinue the study drug.

12.5.2.1 Definition of Serious Adverse Events

An AE is considered "serious" if in the view of either the Investigator or Sponsor, it meets 1 or more of the following criteria:

- Is fatal
- Is life-threatening
- Results in in-patient hospitalization or prolongation of existing hospitalization. If hospitalization occurs, then the SAERF must follow initial admission to the hospital, regardless of duration of hospitalization.
- Results in a persistent or significant incapacity or substantial disruption of the ability to conduct normal life functions
- Is a congenital anomaly/birth defect
- Is an important medical event

Important medical events are those that may not be immediately life-threatening but are clearly of major clinical significance. They may jeopardize the patient, and may require intervention to prevent one of the other serious outcomes noted above. For example, drug overdose or abuse, a seizure that did not result in inpatient hospitalization, or intensive treatment of bronchospasm in an emergency department would typically be considered serious.

A planned medical or surgical procedure is considered an SAE for this study, even if it requires hospitalization.

12.5.2.2 Definition of Adverse Events of Special Interest

An adverse event of special interest (AESI) is one of scientific and medical interest specific to understanding of the study drug(s) and may require close monitoring and rapid communication by the investigator to the sponsor. An AESI may be serious or non-serious. The rapid reporting of AESIs allows ongoing surveillance of these events in order to characterize and understand them in association with the use of this/these study drug(s).

AESIs for this study population, listed below, must be reported by Investigators to the Sponsor but will not be expedited on an individual basis. Instead these AESIs will be reviewed in aggregate during data review.

List of Adverse Events of Special Interest for this Study

- Cytokine release syndrome
- Infusional reactions
- Low platelet count
- Increased liver function test results

Infusion of biological products is commonly associated with infusion-related reactions. Anaphylaxis and infusion-related reactions have some common manifestations and may be difficult to distinguish from each other. Infusion-related reactions are commonly observed during or shortly after the first time exposure to therapeutic monoclonal antibodies delivered through IV infusion. These reactions are less common following subsequent exposures. Unlike infusion-related reactions, anaphylaxis is a rare event, usually occurring after subsequent exposure to an antigen, and it is most commonly accompanied by severe systemic skin and or mucosal reactions.

The Investigator is advised to carefully examine symptoms of adverse reactions observed during or shortly after exposure to APX005M, and consider the above-mentioned facts prior to making a final diagnosis. Reactions occurring at the time of or shortly after subsequent infusions of study drug(s) are to be judged by the Investigator at his/her own discretion.

Should the Investigator determine an infusion-related reaction or cytokine release syndrome have occurred, the underlying symptoms must be reported as individual Adverse Events in addition to the reaction or syndrome. This applies to the Adverse Event reporting guidelines detailed in Section 12.5, as well as Adverse Event eCRF entry.

AESIs that meet seriousness criteria as defined in Section 12.5.2.1, should be reported in the same way as SAEs, described in Section 12.5.5.

12.5.3 Recording Adverse Events

Patients will be instructed to report AEs at each study visit.

All AEs will be collected from the start of study drug through 100 days after the last dose of study drug, or until initiation of a new systemic anti-cancer therapy (whichever occurs first). All treatment-related AEs ongoing at the time of treatment discontinuation are to be followed until resolution, or deemed irreversible by the Investigator, regardless of whether new anti-cancer therapy has been initiated.

Specific guidelines for classifying AEs by intensity and relationship to study drug are given in Table 15 and Table 16.

The severity of AEs will be graded according to the NCI CTCAE version 4.03 (Grades 1 to 5). Grade refers to the severity of the AE. The CTCAE displays Grades 1 through 5 with unique clinical descriptions of severity for each AE based on the general guideline in Table 15.

When changes in the intensity of an AE occur more frequently than once a day, the maximum intensity for the event should be noted. If the intensity category changes over a number of days, then those changes should be recorded separately (with distinct onset dates).

Table 15 Classification of Adverse Events by Intensity

**Grade 1:** Mild; asymptomatic or mild symptoms; clinical or diagnostic observations only; intervention not indicated
**Grade 2:** Moderate; minimal, local or noninvasive intervention indicated; limiting age-appropriate instrumental ADL
**Grade 3:** Severe or medically significant but not immediately life-threatening; hospitalization or prolongation of hospitalization indicated; disabling; limiting self-care ADL
**Grade 4:** Life-threatening consequences; urgent intervention indicated
**Grade 5:** Death related to AE

Table 16 Classification of Adverse Events by Relationship to Study Drug

**UNRELATED**: This category applies to those AEs that are clearly and incontrovertibly due to extraneous causes (disease, environment, etc.).
**UNLIKELY**: This category applies to those AEs that are judged to be unrelated to the test drug, but for which no extraneous cause may be found. An AE may be considered unlikely to be related to study drug if or when it meets 2 of the following criteria: (1) it does not follow a reasonable temporal sequence from administration of the test drug; (2) it could readily have been produced by the patient's clinical state, environmental or toxic factors, or other modes of therapy administered to the patient; (3) it does not follow a known pattern of response to the test drug; or (4) it does not reappear or worsen when the drug is re-administered.
**POSSIBLY**: This category applies to those AEs for which a connection with the test drug administration appears unlikely but cannot be ruled out with certainty. An AE may be considered possibly related if or when it meets 2 of the following criteria: (1) it follows a reasonable temporal sequence from administration of the drug; (2) it could not readily have been produced by the patient's clinical state, environmental or toxic factors, or other modes of therapy administered to the patient; or (3) it follows a known pattern of response to the test drug.
**PROBABLY**: This category applies to those AEs that the investigator feels with a high degree of certainty are related to the test drug. An AE may be considered probably related if or when it meets 3 of the following criteria: (1) it follows a reasonable temporal sequence from administration of the drug; (2) it could not be reasonably explained by the known characteristics of the patient's clinical state, environmental or toxic factors, or other modes of therapy administered to the patient; (3) it disappears or decreases on cessation or reduction in dose (note that there are exceptions when an AE does not disappear upon discontinuation of the drug, yet drug-relatedness clearly exists; for example, as in bone marrow depression, fixed drug eruptions, or tardive dyskinesia); or (4) it follows a known pattern of response to the test drug.
**DEFINITELY**: This category applies to those AEs that the investigator feels are incontrovertibly related to test drug. An AE may be assigned an attribution of definitely related if or when it meets all of the following criteria: (1) it follows a reasonable temporal sequence from administration of the drug; (2) it could not be reasonably explained by the known characteristics of the patient's clinical state, environmental or toxic factors, or other modes of therapy administered to the patient; (3) it disappears or decreases on cessation or reduction in dose and recurs with re-exposure to drug (if rechallenge occurs); and (4) it follows a known pattern of response to the test drug.

The relationship to study drug (either APX005M or nivolumab) will be recorded on the eCRF.

At each contact with the patient, the Investigator will seek information on AEs by specific questioning and, as appropriate, by examination. Information on all AEs will be recorded immediately in the source document, and also in the appropriate AE module of the CRF. All clearly related signs, symptoms, and abnormal diagnostic procedures results should recorded in the source document, though should be grouped under one diagnosis.

All AEs occurring during the study period will be recorded. The clinical course of each event will be followed until resolution or stabilization, or until it has been determined that the study treatment or participation is not the cause. SAEs that are still ongoing at the end of the study period will be followed up to determine the final outcome. Any SAE that occurs after the study period and is considered to be possibly related to the study treatment or study participation will be recorded and reported immediately (see Section 12.5.5).

12.5.4 Disease-related Events and/or Disease-related Outcomes Not Qualifying as Adverse Events or Serious Adverse Events

Progression of the cancer under study is not itself considered a reportable event. Any suspected endpoint which upon review from the Investigator is not considered progression of the cancer under study must be reported as an SAE. Within 24 hours of determination that the event is not progression of the cancer under study, an SAERF should be forwarded to PICI Pharmacovigilance Group as described in Section 12.5.5.

All deaths will be recorded on the Death eCRF page.

12.5.5 Serious Adverse Event Reporting

All SAEs, will be collected from the time the patient signs informed consent through 100 days after the last dose of study drug, or until initiation of a new systemic anti-cancer therapy (whichever occurs first). SAEs related to study drug must be reported at all times, regardless of whether new anti-cancer therapy has been initiated. All treatment-related SAEs must also to be followed until resolution, or deemed irreversible by the Investigator, regardless of whether new anti-cancer therapy has been initiated.

Prior to the initiation of study drug, only SAEs that are related to a protocol-mandated intervention should be reported.

All SAEs must be reported to the PICI Pharmacovigilance Group. Any SAE occurring after the initial study drug due to any cause, whether or not related to the study drug, must be reported within 24 hours of site awareness of the event. Please fax or email the SAERF to the PICI Pharmacovigilance Group within 24 hours of event awareness. If technical issues arise, please contact the PICI Pharmacovigilance Group immediately.

<u>PICI Pharmacovigilance Group Contact Information</u>:

- Pharmacovigilance Fax Number: 415-610-5471
- Pharmacovigilance email: safety@parkerici.org
- Pharmacovigilance Telephone Number: 415-930-4414

If the Investigator contacts the PICI Pharmacovigilance Group by telephone, then the SAERF must follow within one business day.

The event must also be recorded on the standard AE eCRF. Preliminary reports of SAEs must be followed by detailed descriptions later on, including clear and anonymized photocopies of hospital case reports, consultant reports, death certificates, autopsy reports, and other documents when requested and applicable. SAE reports must be made whether or not the Investigator considers the event to be related to the investigational drug.

Appropriate remedial measures should be taken to treat the SAE, and the response should be recorded. Clinical, laboratory, and diagnostic measures should be employed as needed in order to determine the etiology of the problem. The Investigator must report all additional follow-up evaluations to the PICI Pharmacovigilance Group when requested. All SAEs will be followed until the Investigator and Sponsor agree the event is satisfactorily resolved.

Any SAE that is not resolved by the end of the study or upon discontinuation of the patient's active participation in the study is to be followed until it either resolves, stabilizes, returns to baseline values (if a baseline value is available), is deemed irreversible, or is shown to not be attributable to the study drug or procedures. If a subject withdraws consent for data collection, the event will not be followed.

If an SAE has not resolved at the time of the initial report and new information arises that changes the investigator's assessment of the event, a follow-up report including all relevant new or reassessed information (e.g., concomitant medication, medical history) should be submitted to the IRB. The Investigator is responsible for ensuring that all SAEs are followed until either resolved or stable.

12.5.6 Regulatory Reporting Requirements for Serious Adverse Events

Prompt notification by the investigator to the Sponsor of a SAE is essential so that legal obligations and ethical responsibilities towards the safety of patients and the safety of a study treatment under clinical investigation are met. Investigators must also comply with local requirements for reporting SAEs to the IRB/IEC or other local health authorities.

12.5.6.1 Sponsor Reporting of Serious Adverse Events

SAEs will be reported by the PICI Pharmacovigilance Group to regulatory authorities, the overall PI, Bristol-Myers Squibb, Apexigen, the IRB, as appropriate. The process for such reporting, including contact information and specific instructions for reporting to each of these organizations, is described in the Safety Monitoring Plan (a separate document).

12.5.7 Pregnancy

Female patients of child-bearing potential must have a negative pregnancy test at Screening. Following administration of study drug, any known cases of pregnancy in female patients or female partners of male patients will be reported until the patient completes or withdraws from the study. The pregnancy will be reported immediately by phone and by faxing a completed Pregnancy Report to the PICI Pharmacovigilance Group within 24 hours of knowledge of the confirmation of pregnancy. The pregnancy will be processed as an SAE, as a medically significant event, and the Investigator will follow the patient until completion of the pregnancy and must assess the outcome in the shortest possible time but not more than 30 days after completion of the pregnancy. The Investigator should notify the PICI Pharmacovigilance Group of the pregnancy outcome by submitting a follow-up Pregnancy Report. If the outcome of the pregnancy meets the following criteria:

- Spontaneous or therapeutic abortion (any congenital anomaly detected in an aborted fetus is to be documented)
- Stillbirth
- Neonatal death
- Congenital anomaly

The Investigator will report the event by phone and by faxing a completed SAERF to the PICI Pharmacovigilance Group within 24 hours of knowledge of the event.

12.5.8 Overdose

The Investigator must immediately notify PICI Pharmacovigilance Group of any occurrence of overdose with study drug. Overdose is defined as any dose higher than the dose specified to be administered in accordance with the protocol.

All overdoses should be reported as SAEs (see Section 12.5.5). Details of signs and symptoms, clinical management, and outcome should be reported, if available. Overdoses should also be captured as protocol deviations.

The Monitoring plan contains a section related to classification of deviations, and overdoses will be classified in that document. Deviations are to be captured in the deviation log and reviewed on an ongoing basis by the PICI Medical Monitor and Project Manager. The PI is notified by PICI of respective deviations and has the obligation to report to the IRB.

13 STATISTICAL ANALYSIS

This is a multi-center open-label Phase 1b/2 trial of CD40 agonistic monoclonal antibody (APX005M) administered with nab-paclitaxel and Gem with or without nivolumab in previously untreated metastatic pancreatic adenocarcinoma. The primary objectives of the Phase 1b study are to determine the feasibility, safety and DLTs of each treatment cohort and to determine the RP2D of APX005M in combination with NP/Gem and with nivolumab/NP/Gem. The primary objective of the randomized Phase 2 study is to evaluate OS in three treatment arms: nivolumab/NP/Gem, NP/Gem/APX005M and nivolumab/NP/Gem/APX005M by comparing the 1-year OS rate with the historical value for NP/Gem. The study is not powered for statistical comparison among arms, and no interim analyses are planned in Phase 2.

Phase 1b Design:

Four treatment cohorts will be evaluated for feasibility and safety, as shown in Table 17. During Phase 1b, dosing of the first 3 patients of each cohort will be staggered by at least one week. If at one week, and for each of the 3 patients, there are no ongoing symptoms of cytokine release syndrome related to the infusion, and if no DLT occurs, subsequent patients to the cohort may be dosed without restriction. Cohorts B1 and B2 will escalate the dose of APX005M when combined with NP/Gem, and then Cohorts C1 and C2 will escalate the dose of APX005M when combined with nivolumab/NP/Gem. Approximately 6 DLT-evaluable patients will be enrolled in each cohort. A1 (nivolumab/NP/Gem) will not be tested, since an external study is being conducted to confirm the safety of nivolumab in combination with NP/Gem. For all cohorts, a treatment cycle is 4 weeks (3 weeks of treatment with 1 week of rest).

In general, DLT is defined as any Grade 3 or higher toxicity that is treatment-related but not related to the natural progression of the tumor and occurs during the DLT observation period (see Section 6.1). The DLT observation period is defined as the time from first administration of investigational agents until prior to Cycle 2 Day 1.

The DLT-evaluable population consists of patients who received 2 or 3 doses of NP/Gem and 1 dose of APX005M during Cycle 1, thus have completed the DLT observation period. Patients who do not remain on study up to this time for reasons other than DLT will be replaced.

Dose escalation will proceed if 1 or fewer DLT-evaluable patients experience DLT during this observation period. Dose escalation will cease if 2 or more DLT-evaluable patients experience DLT.

Table 17 Phase 1b Statistical Design

Cohort	Regimen	Number of DLT-Evaluable Patients	Comments
A1	Nivolumab/NP/Gem	0	Nivolumab dose from external study
B1	NP/Gem/APX005M 0.1 mg/kg	6	
B2	NP/Gem/APX005M 0.3 mg/kg	6	
C1	Nivolumab/NP/Gem/APX005M 0.1 mg/kg	6	
C2	Nivolumab/NP/Gem/APX005M 0.3 mg/kg	6	Tested only if B2 is safe

DLT = dose-limiting toxicity; Gem = gemcitabine; NP = nab-paclitaxel

On the basis of discussions among the site PIs, Sponsors, and other stakeholders, concerning feasibility, safety, clinical and immune PD effects (totality of available data), the RP2D is defined by the highest APX005M dose with < 2 DLT in 6 DLT-evaluable patients, unless the totality of available data suggests a lower APX005M dose.

Phase 1b Objectives:

The primary objectives are to

1. Determine feasibility, safety, and DLT of each treatment cohort
2. Determine the RP2D of APX005M when combined with NP/Gem
3. Determine the RP2D of APX005M when combined with nivolumab/NP/Gem

The secondary objectives are to

1. Determine OR and DOR of each treatment cohort

The exploratory objectives are:

1. Assess the PK of APX005M
2. Assess immune pharmacodynamic effects of each treatment arm, in both blood and tumor tissue

Phase 1b Plans for Data Analysis: Statistical analyses will include the following:

- The number of patients treated in each cohort will be reported, and reasons why any patient is not DLT evaluable will be summarized.
- Approximately 6 DLT-evaluable patients will be fully analyzed in each treatment cohort.
- Feasibility, defined by the number of patients who complete the DLT observation period and receive the intended therapy without delays or dose modification, will be described for each treatment arm.

- Toxicities will be graded by NCI CTCAE v4.03, causality attributed, and tabulated by treatment arm.
- RP2D of APX005M when combined with NP/Gem and with nivolumab/NP/Gem will be determined.
- RECIST OR will be scored and tabulated along with DOR, by treatment arm.
- PK of APX005M
- Immune PD effects will be measured and summarized, including change from baseline, and reported by treatment arm.

Phase 2 Design

Once the RP2D of APX005M for Arm C has been defined, the randomized Phase 2 portion of the study will commence (Table 18). Patients will be randomized to 1 of 3 arms, defined by the addition of one or more immunotherapy agents to standard of care NP/Gem. The arms will be either A1 vs B2 vs C2 or A1 vs B1 vs C1. Note the APX005M dose must be the same in Arms B and C, regardless of whether a higher APX005M dose was determined to be safe in Arm B. For each regimen, efficacy will be evaluated by comparing the 1-year OS rate to the historical value for NP/Gem.

It is common for randomized Phase 2 studies that test the addition of an experimental agent to a standard of care regimen to include the standard of care arm. However, the setting for this study is unique. NP/Gem, the standard of care regimen, was reported recently in a very similar patient population, and the 1-year OS rate was estimated with extremely high precision (i.e., 1-year OS rate was 35% with 95% CI 30%-39%) based on 431 treated patients.[6] With hundreds of patients with PC treated with NP/Gem since that report, experts in this field agree that the 1-year OS rate estimate appears to be very robust. Thus, this study will not include a standard of care arm.

As shown in Table 18, 12 DLT-evaluable patients who were enrolled in Phase 1b at the RP2D (6 on Arm B and 6 on Arm C) will be included in the efficacy evaluation and approximately 93 additional patients will be randomized in Phase 2, for a total sample size of approximately 105 patients (35 per treatment arm). In step 1, 12 patients will be randomized in a 4:1:1 allocation, to achieve balance in the total number of patients enrolled in the arms (i.e., Arm A1 must be allocated more patients). Then, in step 2, 81 patients will be randomized in a 1:1:1 allocation.

Table 18 Phase 2 Statistical Design

Arm	Regimen	Phase 1b	Phase 2		Total
			Step 1	Step 2	
		Number of Patients	Number of Patients	Number of Patients	Number of Patients
A1	Nivolumab/NP/Gem	0	8	27	35
B2[a]	NP/Gem/APX005M 0.3 mg/kg	6	2	27	35
C2[a]	Nivolumab/NP/Gem/APX005M 0.3 mg/kg	6	2	27	35

a Or B1 and C1, if either B2 or C2 is not tolerable.

Phase 2 Objectives:

The primary objectives are to

1. Estimate OS of each treatment arm
2. Compare 1-year OS rate of each treatment arm to the historical rate for NP/Gem

The secondary objectives are to

1. Determine the ORR, DCR, DOR, and PFS of each treatment arm
2. Further characterize the feasibility and safety of each treatment arm

The exploratory objectives are to

1. Assess PK of APX005M in Cycles 1 to 4
2. Assess immune pharmacodynamic effects of each treatment arm, in both blood and tumor tissue
3. Assess associations between immune biomarkers and clinical outcomes
4. Evaluate baseline and on-treatment microbiome profiles and association with clinical outcomes
5. Construct multivariable linear models to dissect the pharmacodynamic effects of APX005M and nivolumab on immune biomarkers

Early Termination Rules for Unacceptable Toxicity in Phase 2

A Bayesian rule will be employed to monitor toxicity during Phase 2 (Table 19). A minimally informative beta (0.5, 2.5) prior has been assumed, which is information that is equivalent to half the weight of 1 DLT in 6 patients treated, the definition of a safe dose in Phase 1b. For each treatment arm, if the number of patients with an unacceptable toxicity (defined in Section 6.1) is greater than or equal to the number in Table 19, then termination of that particular treatment arm will be considered, as it is likely that the toxicity rate is > 30%, as noted by the Bayesian posterior probabilities. This rule is intentionally conservative early in the enrollment phase.

Table 19 Bayesian Termination Rules

**Rules for Toxicity Rate >30%**					
Patients treated on an arm	10	15	20	25	30
Patients with unacceptable toxicity	4	6	9	11	13
Posterior Probability [toxicity rate > 30%]	0.61	0.69	0.87	0.88	0.90
Action	Consider termination of arm, re-evaluate study design.				

Phase 2 Plans for Data Analysis:

Statistical analyses will include the following:

- 35 patients will be analyzed on each treatment arm. 12 DLT-evaluable patients from Phase 1 (Arms B and C) and 93 patients from Phase 2 will compose the population for the final analysis of efficacy.

- OS will be estimated by the Kaplan-Meier method for each treatment arm.
- The 1-year OS rate and 1-sided 95% confidence interval (CI) will be calculated for each treatment arm, to determine whether the lower bound of the CI excludes the historical value (or a recently updated value) for NP/Gem.
- A 1-sided one-sample Z test will also be conducted. The goal is to compare the survival probability at 1-year to the historical value of 0.35.
- ORR and DCR and their 95% CIs will be calculated for each treatment arm.
- DOR will be calculated from dates of first documented response and progression of disease.
- PFS will be estimated by the Kaplan-Meier method and the median PFS and 95% CI will be calculated for each treatment arm. Toxicities will be graded by NCI CTCAE v4.03 and summarized by treatment arm.
- PK of APX005M
- Immune pharmacodynamic endpoints (see Section 5.2.2.3) will be measured and summarized, including change from baseline, and reported by treatment arm. In addition to scatter and box plots, continuous variables will be summarized with the number of patients (N), mean, standard deviation, median, minimum, and maximum by treatment group.
- Associations between baseline values and changes in immune pharmacodynamic variables or microbiome profiles and clinical outcomes will be assessed. Logistic and Cox regression models will be employed for binary (responder/non-responder) outcomes and OS or PFS outcomes, respectively.
- Multivariable linear models will be constructed to dissect the effects of APX005M and nivolumab on immune biomarkers. To begin, a linear model is constructed with a post-treatment value of a biomarker at a selected time point as the dependent variable and the pre-treatment value, two indicator variables for APX005M and nivolumab treatment and treatment interaction term, as the independent variables. To analyze serial measurement of immune biomarkers, a linear mixed effects model is constructed to assess the impact of the individual and combined therapies on longitudinal changes, taking into account within-patient correlation of the serial samples.

13.1 Determination of Sample size

13.1.1 Phase 1b

Assuming 4 treatment cohorts will be evaluated for feasibility, safety, and DLTs, up to 24 DLT-evaluable patients will be enrolled.

13.1.2 Phase 2

Approximately ninety-three patients will be enrolled in Phase 2. This is a screening study, such that for each treatment arm, the 1-year OS rate will be estimated and compared with a historical value of 35% for NP/Gem.[6] The study is not powered to detect a meaningful difference in OS among the 3 arms, since these are novel experimental arms and OS is unknown.

The null hypothesis is a 1-year OS rate of 35% and the alternative hypothesis is a 1-year OS rate of 58%. The 1-year OS rate is estimated by the Kaplan-Meier method. A sample size of 35 patients on each arm provides 88% power to test this hypothesis, using a 1-sided one-sample Z test with 5% type I error rate, assuming a minimum of 1 year of follow-up for each patient. Moreover, the sample size of 35 patients on each arm provides 81% power to statistically test the null hypothesis versus a slightly more conservative alternative hypothesis that the 1-year OS rate is 55%, given the same design assumptions.

These calculations assume that at least 105 patients (35 patients x 3 arms) will be enrolled. There is no assumption about the duration of patient enrollment, only that there will be a minimum of 1 year of follow-up for each patient.

13.2 Analysis Populations

The safety population consists of all patients who received at least 1 dose of any study drug. This is the population for the primary analyses of safety. A subset of the safety population is the DLT-evaluable population, described in Section 6.1.

The DLT-evaluable population consists of patients who received 2 or 3 doses of NP/Gem during Cycle 1, as well as one dose of APX005M, thus have completed the DLT observation period (ie, from the time of first administration of investigational agents until prior to Cycle 2 Day 1). Patients who do not meet these criteria will be replaced in Phase 1b only, to assist with DLT and RP2D decision-making.

The efficacy population consists of (1) all patients who were randomized/enrolled in Phase 2 and received at least 1 dose of any study drug and (2) the 12 DLT-evaluable patients (6 on Arm B and 6 on Arm C) who were enrolled in Phase 1b at the RP2D. The efficacy population is the population for the primary analyses of efficacy.

13.3 Demographic and Baseline Characteristics

All data will be listed, and summary tables will be provided. Summary statistics will be presented by treatment arm. For continuous variables, data will be summarized with the number of patients (N), mean, standard deviation, median, minimum, and maximum by treatment group. For categorical variables, data will be tabulated with the number and proportion of patients for each category by treatment arm.

13.4 Efficacy Analysis

13.4.1 Primary Efficacy Endpoint

The primary efficacy endpoint will be determined as follows:

- OS is defined as the time from initiation of study therapy to date of death due to any cause or date of most recent patient contact. Patients who were alive are censored on their most recent contact date.
- 1-year OS rate in each treatment arm
- Subgroup analysis: For each treatment arm, OS will be analyzed in the subset of patients in the efficacy population who remain on study for at least 6 weeks. This subset

is in contrast to the efficacy population in the primary analysis. The 1-year OS rate and 1-sided 95% CI will again be calculated and compared with a historical value.

13.4.2 Secondary Efficacy Endpoints

The following secondary endpoints will be determined in the Phase 2 portion of the study:

- OR is determined by RECIST and the ORR is defined as the proportion of patients who achieve a CR or PR. A 95% CI for the rate will be constructed. The half-width of this confidence interval will be no greater than 16.5%, based on 35 patients in each treatment arm
- DCR is defined as the proportion of patients who achieve a CR, PR, or SD. A 95% CI for the rate will be constructed. The half-width of this CI will be no greater than 16.5%, based on 35 patients in each treatment arm
- DOR is defined as the time for first documentation of response (CR or PR) to first documentation of PD. PFS is defined as the time from initiation of study therapy to date of first documented PD, date of death due to any cause or date of most recent patient contact which documented progression-free status (i.e., clinic visit date or scan date). Patients who have not progressed or died are censored on their most recent progression-free date.

13.4.3 Exploratory Endpoints

Tissue Assessment: From germline WES, HLA type can be determined. From tumor WES, tumor RNA-Seq, and germline WES, patient-specific neo epitopes arising from tumor somatic missense mutations can be predicted bioinformatically.

Analysis of T-cell Activation: For each T-cell subset, differentiation status (e.g., naïve, central memory, effector memory) or activation vs. exhaustion status may be assessed using additional markers such as Eomes, Tbet, Granzyme B, Ki-67, CD45RA, ICOS, CD45RO, CCR7, CD28, CD27, CD57, CD25, CD69, HLA-DR, CTLA-4, and PD-1. When possible, trends will be tracked in T-cell subsets based on analysis of multiple post-treatment samples. NK cells subsets may also be assessed using CD16 and CD56, with CD69 as an activation marker.

Analysis of Myeloid and B-cell Activation: For each parameter of B-cell activation and each cell type, the percentage of cells positive for the marker and/or MFI at time points after treated can be compared to baseline and the change calculated as $\%_{after}/\%_{baseline}$ or $MFI_{after}/MFI_{baseline}$.

Inflammatory Cytokines/Chemokines: Concentrations of cytokines and other circulating factors may include but are not limited to TGF-β, IL-1, TNF-α, IL-6 and others may be determined and summarized.

TCR Deep Sequencing: Comparison of TCR beta sequence data in serial samples from blood and tumor may be used to demonstrate de novo evolution of an anti-tumor T-cell repertoire.

Microbiome profile: Stool samples may be tested for microbiome profile using sequencing or metabolomic methods.

13.5 Pharmacokinetic Analysis

Circulating concentrations of APX005M will be determined from venous blood samples collected periodically during the study, as indicated in the Schedule of Assessments (Table 11 and Table 12).

The standard PK parameters of peak plasma concentration (C_{max}), area under the curve (AUC_{0-x}), elimination half-life ($t_{1/2}$), elimination rate constant (λ), volume of distribution (V_z), and clearance (C) will be estimated by standard noncompartmental methods. This description could also include single- and multiple-dose parameters, use of WinNonlin program, and linear least squares.

13.6 Safety Endpoints

13.6.1 Phase 1b: Assessing Feasibility, Dose-Limiting Toxicity, and Recommended Dose for Phase 2

The following will be determined for the Phase 1b portion of the study:

- Feasibility is defined by the number of patients who complete the DLT observation period and receive the intended therapy without delays or dose modification.
- A DLT is defined as any Grade 3 or higher toxicity that is treatment-related but not related to the natural progression of the tumor and occurs during the DLT observation period. See Section 6.1 for further details.
- The RP2D of APX005M when combined with NP/Gem or nivolumab/NP/Gem is defined by the highest APX005M dose with < 2 DLTs in 6 DLT-evaluable patients, unless the totality of available data, including clinical and pharmacodynamic effects, suggests a lower APX005M dose.

13.6.2 Analysis of Adverse Events

All reported AEs will be coded using the most current version of the Medical Dictionary for Regulatory Activities. The incidence of TEAEs (events with onset dates on or after the start of the study drug) will be included in incidence tables. Events with missing onset dates will be included as treatment-emergent. If a patient experiences more than 1 occurrence of the same AE, the occurrence with the greatest severity and the closest association with the study drug will be used in the summary tables. SAEs and AEs causing treatment discontinuation will be tabulated. All AEs will be listed by patient, along with information regarding onset, duration, relationship and severity to study drug, action taken with study drug, treatment of event, and outcome.

Clinical laboratory data and vital signs will be summarized using descriptive statistics including mean values and mean change from baseline values, as well as numbers of patients with values outside limits of the normal range at each time point.

Summary tables will be provided for concomitant medications initiated during the study period.

13.7 Interim Analysis

Given the hypothesis generating nature of this study, the Sponsor may conduct up to 3 interim analyses of safety and efficacy during the Phase 1b and Phase 2 portions of the study. Should an interim analysis occur, it will be performed and interpreted by members of the Sponsor study team. If warranted, interim safety and efficacy results may be shared with study Investigators. The decision to conduct an interim analysis, its timing and scope, and who will have access to the results, will be documented in the sponsor's trial master file and statistical analysis plan prior to the conduct of the interim analysis. The clinical study report will also document that such an interim analysis occurred.

13.8 Data Monitoring

13.8.1 Data Review Team (DRT)

To ensure patients' safety during Phase 1b and Phase 2, a DRT will review the safety data on a regular basis. The DRT consists of members from the Sponsor, the overall PI, the lead statistician, and all active PIs. The DRT will decide on DLTs relevant for the treatment and will decide by consensus on dose escalation, dose de-escalation, prolongation of the DLT observation period, suspension of enrollment based on safety, PK or possibly pharmacodynamic data, and will recommend the dose level for the Phase 2 part.

14 STUDY MANAGEMENT

14.1 Approval and Consent

14.1.1 Regulatory Guidelines

This study will be conducted in accordance with the accepted version of the Declaration of Helsinki and/or all relevant federal regulations, as set forth in Parts 50, 56, 312, Subpart D, of Title 21 of the Code of Federal Regulations (CFR), and in compliance with Good Clinical Practice (GCP) guidelines.

14.1.2 Institutional Review Board/Independent Ethics Committee

Conduct of the study must be approved by an appropriately constituted IRB/Independent Ethics Committee (IEC). Approval is required for the study protocol, investigational drug brochure, protocol amendments, informed consent forms (ICFs), and patient information sheets.

14.1.3 Informed Consent

For each study patient, written informed consent will be obtained prior to any protocol-related activities. As part of this procedure, the PI or one of his/her associates must explain orally and in writing the nature, duration, and purpose of the study, and the action of the drug in such a manner that the patient is aware of the potential risks, inconveniences, or adverse effects that may occur. The patient should be informed that he/she may withdraw from the study at any time, and the patient will receive all information that is required by local regulations and International Council for Harmonization (ICH) guidelines. The PI will provide the Sponsor or its representative with a copy of the IRB/IEC-approved ICF prior to the start of the study.

14.2 Data Handling

Any data to be recorded directly on the eCRFs (to be considered as source data) will be identified at the start of the study. Data reported on the eCRFs that are derived from source documents should be consistent with the source documents, or the discrepancies must be explained.

Clinical data will be entered on eCRFs for transmission to the Sponsor. Data on eCRFs transmitted via the web-based, electronic data capture (EDC) system must correspond to and be supported by source documentation maintained at the study site, unless the study site makes direct data entry to the databases for which no other original or source documentation is maintained. In such cases, the study site should document which eCRFs are patient to direct data entry and should have in place procedures to obtain and retain copies of the information submitted by direct data entry. All study forms and records transmitted to the Sponsor must carry only coded identifiers such that personally identifying information is not transmitted. The primary method of data transmittal is via the secure, EDC system maintained by PICI. Access to the EDC system is available to authorized users via the study’s Internet website, where an assigned username and password are required for access.

Electronic CRFs will be considered complete when all missing and/or incorrect data have been resolved.

14.3 Source Documents

Source documents are considered to be all information in original records and certified copies of original records of clinical findings, observations, data or other activities in a clinical study necessary for the reconstruction and evaluation of the study.

14.4 Record Retention

Study records and source documents must be preserved for at least 15 years after the completion or discontinuation of/withdrawal from the study or 2 years after the last approval of a marketing application in an ICH region, whichever is the longer time period.

The Investigator agrees to comply with all applicable federal, state, and local laws and regulations relating to the privacy of patient health information, including, but not limited to, the Standards for Individually Identifiable Health Information, 45 CFR, Parts 160 and 164 (the Health Insurance Portability Accountability Act of 1996 [HIPAA] Privacy Regulation). The Investigator shall ensure that study patients authorize the use and disclosure of protected health information in accordance with HIPAA Privacy Regulation and in a form satisfactory to the Sponsor.

14.5 Monitoring

The study will be monitored to ensure that it is conducted and documented properly according to the protocol, GCP, and all applicable regulatory requirements.

On-site monitoring visits will be made at appropriate times during the study. Clinical monitors must have direct access to source documentation in order to check the completeness, clarity, and consistency of the data recorded on the eCRFs for each patient.

The Investigator will make available to the clinical monitor source documents and medical records necessary to verify eCRFs. In addition, the Investigator will work closely with the clinical monitor and, as needed, provide them appropriate evidence that the conduct of the study is being done in accordance with applicable regulations and GCP guidelines.

14.6 Quality Control and Quality Assurance

The Sponsor or its designee will perform the quality assurance and quality control activities of this study; however, responsibility for the accuracy, completeness, and reliability of the study data presented to the Sponsor lies with the Investigator generating the data.

The Sponsor will arrange audits as part of the implementation of quality assurance to ensure that the study is being conducted in compliance with the protocol, Standard Operating Procedures, GCP, and all applicable regulatory requirements. Audits will be independent of and separate from the routine monitoring and quality control functions. Quality assurance procedures will be performed at study sites and during data management to assure that safety and efficacy data are adequate and well documented.

14.7 Protocol Amendments and Protocol Deviations

14.7.1 Protocol Amendments

Amendments to the protocol that entail corrections of typographical errors, clarifications of confusing wording, changes in study personnel, and minor modifications that have no impact on the safety of patients or the conduct of the study will be classed as administrative amendments and will be submitted to the IRB/IEC for information only. The Sponsor will ensure that acknowledgment is received and filed. Amendments that are classed as substantial amendments must be submitted to the appropriate Regulatory Authorities and the IRBs/IECs for approval.

14.7.2 Protocol Deviations

Should a protocol deviation occur, the Sponsor must be informed. Protocol deviations and/or violations and the reasons they occurred will be included in the clinical study report. Reporting of protocol deviations to the IRB/IEC and in accordance with applicable Regulatory Authority mandates is an Investigator responsibility.

14.8 Ethical Considerations

This study will be conducted in accordance with the accepted version of the Declaration of Helsinki and/or all relevant federal regulations, as set forth in Parts 50, 56, 312, Subpart D, of Title 21 of the CFR, and in compliance with GCP guidelines.

IRBs/IECs will review and approve this protocol and the ICF. All patients are required to give written informed consent prior to participation in the study.

14.9 Financing and Insurance

Prior to the study commencing, the Sponsor (or its designee) and the Investigator (or the institution, as applicable) will agree on costs necessary to perform the study. This agreement will be documented in a financial agreement that will be signed by the Investigator (or the institution signatory) and the Sponsor (or its designee).

The Investigator is required to have adequate current insurance to cover claims for its negligence and/or malpractice. The Sponsor, or the manufacturer of the study drug, will provide insurance coverage for the clinical study as required by national regulations.

14.10 Publication Policy / Disclosure of Data

Both the use of data and the publication policy are detailed within the clinical study agreement. Intellectual property rights (and related matters) generated by the Investigator and others performing the clinical study will be patient to the terms of a clinical study agreement that will be agreed between the Institution and the Sponsor or their designee.

15 REFERENCES

1. Rahib L, Smith BD, Aizenberg R, et al. Projecting cancer incidence and deaths to 2030: the unexpected burden of thyroid, liver, and pancreas cancers in the United States. Cancer Res 2014;74(11):2913-21.

2. Neoptolemos JP, Stocken DD, Friess H, et al. A randomized trial of chemoradiotherapy and chemotherapy after resection of pancreatic cancer. N Engl J Med 2004;350(12):1200-10.

3. Oettle H, Post S, Neuhaus P, et al. Adjuvant chemotherapy with gemcitabine vs observation in patients undergoing curative-intent resection of pancreatic cancer: a randomized controlled trial. JAMA 2007;297(3):267-77.

4. Oettle H, Neuhaus P, Hochhaus A, et al. Adjuvant chemotherapy with gemcitabine and long-term outcomes among patients with resected pancreatic cancer: the CONKO-001 randomized trial. JAMA 2013;310(14):1473-81.

5. Conroy T, Desseigne F, Ychou M, et al. FOLFIRINOX versus gemcitabine for metastatic pancreatic cancer. N Engl J Med 2011 May 12;364(19):1817-25.

6. Von Hoff DD, Ervin T, Arena FP, et al. Increased survival in pancreatic cancer with nab-paclitaxel plus gemcitabine. N Engl J Med 2013;369(18):1691-703.

7. Pardoll DM. The blockade of immune checkpoints in cancer immunotherapy. Nat Rev Cancer 2012;12(4):252-64.

8. Wolchok JD, Kluger H, Callahan MK, et al. Nivolumab plus ipilimumab in advanced melanoma. N Engl J Med 2013;369(2):122-33.

9. Royal RE, Levy C, Turner K, et al. Phase 2 trial of single agent ipilimumab (anti-CTLA-4) for locally advanced or metastatic pancreatic adenocarcinoma. J Immunother 2010;33:828-33.

10. Brahmer JR, Tykodi SS, Chow LQM, et al. Safety and activity of anti-PD-L1 antibody in patients with advanced cancer. N Engl J Med 2012;366:2455-65.

11. Bauer C, Kühnemuth B, Deuwell P, et al. Prevailing over T cell exhaustion: New developments in the immunotherapy of pancreatic cancer. Cancer Lett 2016;381(1):259-68.

12. Aggarwal BB. Signalling pathways of the TNF superfamily: A double-edged sword. Nat Rev Immunol 2003;3(9):745-56.

13. Banchereau J, Bazan F, Blanchard D, et al. The CD40 antigen and its ligand. Annu

Rev Immunol 1994;12:881-922.

14. Clark EA, Ledbetter JA. How B and T cells talk to each other. Nature 1994;367(6462):425-8.

15. Grewal IS, Flavell RA. CD40 and CD154 in cell-mediated immunity. Annu Rev Immunol 1998;16:111-35.

16. Eliopoulos AG, Young LS. The role of the CD40 pathway in the pathogenesis and treatment of cancer. Curr Opin Pharmacol 2004;4(4):360-7.

17. Unek T, Unek IT, Agalar AA, et al. CD40 expression in pancreatic cancer. Hepatogastroenterology 2013;60(128):2085-93).

18. Hess S, Engelmann H. A novel function of CD40: Induction of cell death in transformed cells. J Exp Med 1996;183(1):159-67.

19. Tong AW, Stone MJ. Prospects for CD40-directed experimental therapy of human cancer. Cancer Gene Ther 2003;10(1):1-13.

20. Law CL, Grewal IS. Therapeutic interventions targeting CD40L (CD154) and CD40: The opportunities and challenges. Adv Exp Med Biol 2009;647:8-36.

21. Khong A, Nelson DJ, Nowak AK, et al. The use of agonistic anti-CD40 therapy in treatments for cancer. Int Rev Immunol 2012;31(4):246-66.

22. Rakhmilevich AL, Alderson KL, Sondel PM. T-cell-independent antitumor effects of CD40 ligation. Int Rev Immunol 2012;31(4):267-78.

23. Ruter J, Antonia SJ, Burris HA, et al. Immune modulation with weekly dosing of an agonist CD40 antibody in a phase I study of patients with advanced solid tumors. Cancer Biol Ther 2010;10(10):983-93.

24. Vonderheide RH, Flaherty KT, Khalil M, et al. Clinical activity and immune modulation in cancer patients treated with CP-870,893, a novel CD40 agonist monoclonal antibody. J Clin Oncol 2007;25(7):876-83.

25. Beatty GL, Chiorean EG, Fishman MP, et al. CD40 agonists alter tumor stroma and show efficacy against pancreatic carcinoma in mice and humans. Science 2011;331(6024):1612-6.

26. Forero-Torres A, Bartlett N, Beaven A, et al. Pilot study of dacetuzumab in combination with rituximab and gemcitabine for relapsed or refractory diffuse large B-cell lymphoma. Leuk Lymphoma 2013;54(2):277-83.

27. Lewis TS, McCormick RS, Stone IJ, et al. Proapoptotic signaling activity of the

anti-CD40 monoclonal antibody dacetuzumab circumvents multiple oncogenic transformation events and chemosensitizes NHL cells. Leukemia 2011;25(6):1007-16.

28. Johnson P, Challis R, Chowdhury F, et al. Clinical and biological effects of an agonist anti-CD40 antibody: A Cancer Research UK phase I study. Clin Cancer Res 2015;21(6):1321-8.

29. Mangsbo SM, Broos S, Fletcher E, et al. The human agonistic CD40 antibody ADC-1013 eradicates bladder tumors and generates T-cell-dependent tumor immunity. Clin Cancer Res. 2015;21(5):1115-26.

30. Li F, Ravetch JV. Inhibitory Fcgamma receptor engagement drives adjuvant and anti-tumor activities of agonistic CD40 antibodies. Science 2011;333(6045):1030-4.

31. Barr TA, Heath AW. Functional activity of CD40 antibodies correlates to the position of binding relative to CD154. Immunology 2001;102(1):39-43.

32. Zou W, Chen. Inhibitory B7-family molecules in the tumour microenvironment. Nat Rev Immunol 2008;8:467-77.

33. Topalian SL, Hodi FS, Brahmer JR, et al. Safety, activity, and immune correlates of anti-PD-1 antibody in cancer. N Engl J Med 2012;366(26):2443-54.

34. Rizvi NA, Hellmann MD, Brahmer JR, et al. Nivolumab in combination with platinum-based doublet chemotherapy for first-line treatment of advanced non-small-cell lung cancer. J Clin Oncol 2016;34(25):2969-79.

35. George B, Kelly K, Ko A, et al. Phase I study of nivolumab + Nab-paclitaxel in solid tumors: preliminary analysis of small cell lung cancer cohort: track: advanced NSCLC. J Thorac Oncol 2016;11:S211-12.

36. Wainberg, ZA, Hochster HS, George B, et al. Phase I Study of Nivolumab (Nivo) Nab-Paclitaxel (Nab-P) ± Gemcitabine (Gem) in Solid Tumors: Interim Results from the Pancreatic Cancer (PC) Cohorts. 2017 Gastrointestinal Cancers Symposium: abstract 412

37. Burris, H.A., Moore MJ, Andersen J, et al. Improvements in survival and clinical benefit with gemcitabine as first-line therapy for subjects with advanced pancreas cancer: a randomized trial. J Clin Oncol, 1997. 15: p. 2403-13.

38. Winograd R, Byrne KT, Evans RA, et al. Induction of T-cell immunity overcomes complete resistance to PD-1 and CTLA-4 blockade and improves survival in pancreatic carcinoma. Cancer Immunol Res 2015;3(4):399-411.

39. Byrne KT, Vonderheide H. CD40 stimulation obviates innate sensors and drives T cell immunity in cancer. Cell Rep 2016;15(12):2719-32.

16 APPENDICES

16.1 ECOG Performance Scale

Table 20 ECOG Performance Scale

Grade	ECOG Performance Status
0	Fully active, able to carry on all pre-disease performance without restriction
1	Restricted in physically strenuous activity but ambulatory and able to carry out work of a light or sedentary nature, eg, light housework, office work
2	Ambulatory and capable of all self-care but unable to carry out any work activities. Up and about more than 50% of waking hours
3	Capable of only limited self-care, confined to bed or chair more than 50% of waking hours
4	Completely disabled. Cannot carry on any self-care. Totally confined to bed or chair
5	Dead

ECOG = Eastern Cooperative Oncology Group.

(Oken MM, Creech RH, Tormey DC, et al. Toxicity and response criteria of the Eastern Cooperative Oncology Group. Am J Clin Oncol. 1982;5(6):649-55).

16.2 Nivolumab Toxicity Management Guide

These general guidelines constitute guidance to the Investigator and may be supplemented by discussions with the Medical Monitor representing the Sponsor. The guidance applies to all immuno-oncology agents and regimens.

A general principle is that differential diagnoses should be diligently evaluated according to standard medical practice. Non-inflammatory etiologies should be considered and appropriately treated.

Corticosteroids are a primary therapy for immuno-oncology drug-related adverse events. The oral equivalent of the recommended IV doses may be considered for ambulatory patients with low-grade toxicity. The lower bioavailability of oral corticosteroids should be taken into account when switching to the equivalent dose of oral corticosteroids.

Consultation with a medical or surgical specialist, especially prior to an invasive diagnostic or therapeutic procedure, is recommended.

The frequency and severity of the related adverse events covered by these algorithms will depend on the immuno-oncology agent or regimen being used.

GI Adverse Event Management Algorithm

Rule out non-inflammatory causes. If non-inflammatory cause is identified, treat accordingly and continue I-O therapy. Opiates/narcotics may mask symptoms of perforation. Infliximab should not be used in cases of perforation or sepsis.

Grade of Diarrhea/ Colitis (NCI CTCAE v4)	Management	Follow-up
**Grade 1**<br>Diarrhea:< 4 stools/day over baseline;<br>Colitis: asymptomatic	• Continue I-O therapy per protocol<br>• Symptomatic treatment	• Close monitoring for worsening symptoms.<br>• Educate patient to report worsening immediately<br>If worsens:<br>• Treat as Grade 2 or 3/4
**Grade 2**<br>Diarrhea: 4-6 stools per day over baseline; IV fluids indicated <24 hrs; not interfering with ADL<br>Colitis: abdominal pain; blood in stool	• Delay I-O therapy per protocol<br>• Symptomatic treatment	If improves to grade 1:<br>• Resume I-O therapy per protocol<br>If persists > 5-7 days or recur:<br>• 0.5-1.0 mg/kg/day methylprednisolone or oral equivalent<br>• When symptoms improve to grade 1, taper steroids over at least 1 month, consider prophylactic antibiotics for opportunistic infections, and resume I-O therapy per protocol.<br>If worsens or persists > 3-5 days with oral steroids:<br>• Treat as grade 3/4
**Grade 3-4**<br>Diarrhea (G3): ≥7 stools per day over baseline; incontinence; IV fluids ≥24 hrs; interfering with ADL<br>Colitis (G3): severe abdominal pain, medical intervention indicated, peritoneal signs<br>G4: life-threatening, perforation	• Discontinue I-O therapy per protocol<br>• 1.0 to 2.0 mg/kg/day methylprednisolone IV or IV equivalent<br>• Add prophylactic antibiotics for opportunistic infections<br>• Consider lower endoscopy	If improves:<br>• Continue steroids until grade 1, then taper over at least 1 month<br>If persists > 3-5 days, or recurs after improvement:<br>• Add infliximab 5 mg/kg (if no contraindication). Note: Infliximab should not be used in cases of perforation or sepsis

Patients on IV steroids may be switched to an equivalent dose of oral corticosteroids (e.g. prednisone) at start of tapering or earlier, once sustained clinical improvement is observed. Lower bioavailability of oral corticosteroids should be taken into account when switching to the equivalent dose of oral corticosteroids.

Renal Adverse Event Management Algorithm

Rule out non-inflammatory causes. If non-inflammatory cause, treat accordingly and continue I-O therapy

Grade of Creatinine Elevation (NCI CTCAE v4)	Management	Follow-up
**Grade 1**<br>Creatinine > ULN and > than baseline but ≤ 1.5x baseline	• Continue I-O therapy per protocol<br>• Monitor creatinine weekly	<u>If returns to baseline</u>:<br>•Resume routine creatinine monitoring per protocol<br><u>If worsens:</u><br>•Treat as Grade 2 or 3/4
**Grade 2-3**<br>Creatinine > 1.5x baseline to ≤ 6x ULN	• Delay I-O therapy per protocol<br>• Monitor creatinine every 2-3 days<br>• 0.5 to 1.0 mg/kg/day methylprednisolone IV or oral equivalent<br>• Consider renal biopsy with nephrology consult	<u>If returns to Grade 1:</u><br>•Taper steroids over at least 1 month, consider prophylactic antibiotics for opportunistic infections, and resume I-O therapy and routine creatinine monitoring per protocol<br><br><u>If elevations persist > 7 days or worsen:</u><br>•Treat as Grade 4
**Grade 4**<br>Creatinine > 6x ULN	• Discontinue I-O therapy per protocol<br>• Monitor creatinine daily<br>• 1.0-2.0 mg/kg/day methylprednisolone IV or IV equivalent<br>• Consult nephrologist<br>• Consider renal biopsy	<u>If returns to Grade 1</u> : Taper steroids over at least 1 month and add prophylactic antibiotics for opportunistic infections

Patients on IV steroids may be switched to an equivalent dose of oral corticosteroids (e.g. prednisone) at start of tapering or earlier, once sustained clinical improvement is observed. Lower bioavailability of oral corticosteroids should be taken into account when switching to the equivalent dose of oral corticosteroids.

Pulmonary Adverse Event Management Algorithm

Rule out non-inflammatory causes. If non-inflammatory cause, treat accordingly and continue I-O therapy. Evaluate with imaging and pulmonary consultation.

Grade of Pneumonitis (NCI CTCAE v4)	Management	Follow-up
**Grade 1**<br>Radiographic changes only	• Consider delay of I-O therapy<br>• Monitor for symptoms every 2-3 days<br>• Consider Pulmonary and ID consults	• Re-image at least every 3 weeks<br><u>If worsens:</u><br>• Treat as Grade 2 or 3-4
**Grade 2**<br>Mild to moderate new symptoms	• Delay I-O therapy per protocol<br>• Pulmonary and ID consults<br>• Monitor symptoms daily, consider hospitalization<br>• 1.0 mg/kg/day methyl-prednisolone IV or oral equivalent<br>• Consider bronchoscopy , lung biopsy	• Re-image every 1-3 days<br><u>If improves:</u><br>• When symptoms return to near baseline, taper steroids over at least 1 month and then resume I-O therapy per protocol and consider prophylactic antibiotics<br><u>If not improving after 2 weeks or worsening:</u><br>• Treat as Grade 3-4
**Grade 3-4**<br>Severe new symptoms; New/worsening hypoxia; Life-threatening	• Discontinue I-O therapy per protocol<br>• Hospitalize<br>• Pulmonary and ID consults<br>• 2-4 mg/kg/day methylprednisolone IV or IV equivalent<br>• Add prophylactic antibiotics for opportunistic infections<br>• Consider bronchoscopy, lung biopsy	<u>If improves to baseline:</u><br>• Taper steroids over at least 6 weeks<br><u>If not improving after 48 hours or worsening:</u><br>• Add additional immunosuppression

Patients on IV steroids may be switched to an equivalent dose of oral corticosteroids (e.g. prednisone) at start of tapering or earlier, once sustained clinical improvement is observed. Lower bioavailability of oral corticosteroids should be taken into account when switching to the equivalent dose of oral corticosteroids.

Hepatic Adverse Event Management Algorithm

Rule out non-inflammatory causes. If non-inflammatory cause, treat accordingly and continue I-O therapy. Consider imaging for obstruction.

Grade of Liver Test Elevation (NCI CTCAE v4)	Management	Follow-up
**Grade 1**<br>AST or ALT > ULN to 3.0 x ULN <u>and/or</u> T. bili > ULN to 1.5 x ULN	• Continue I-O therapy per protocol	• Continue LFT monitoring per protocol<br><u>If worsens:</u><br>• Treat as Grade 2 or 3-4
**Grade 2**<br>AST or ALT > 3.0 to ≤ 5 x ULN <u>and/or</u> T. bili > 1.5 to ≤ 3 x ULN	• Delay I-O therapy per protocol<br>• Increase frequency of monitoring to every 3 days	<u>If returns to baseline</u>:<br>•Resume routine monitoring, resume I-O therapy per protocol<br><br><u>If elevations persist > 5-7 days or worsen :</u><br>• 0.5-1 mg/kg/day methylprednisolone or oral equivalent and when LFT returns to grade 1 or baseline, taper steroids over at least 1 month, consider prophylactic antibiotics for opportunistic infections, and resume I-O therapy per protocol
**Grade 3-4**<br>AST or ALT > 5 x ULN <u>or</u> T.bili >3 x ULN	• Discontinue I-O therapy*<br>• Increase frequency of monitoring to every 1-2 days<br>• 1.0 to 2.0 mg/kg/day methylprednisolone IV or IV equivalent**<br>• Add prophylactic antibiotics for opportunistic infections<br>• Consult gastroenterologist	<u>If returns to grade 2</u>:<br>• Taper steroids over at least 1 month<br><br><u>If does not improve in >3-5 days, worsens or rebounds:</u><br>• Add mycophenolate mofetil 1 g BID<br>• If no response within an additional 3-5 days, consider other immunosuppressants per local guidelines

Patients on IV steroids may be switched to an equivalent dose of oral corticosteroids (e.g. prednisone) at start of tapering or earlier, once sustained clinical improvement is observed. Lower bioavailability of oral corticosteroids should be taken into account when switching to the equivalent dose of oral corticosteroids.

***I-O therapy may be delayed rather than discontinued if AST/ALT ≤ 8 x ULN or T.bili ≤ 5 x ULN.**

******The recommended starting dose for grade 4 hepatitis is 2 mg/kg/day methylprednisolone IV.

Endocrinopathy Management Algorithm

Rule out non-inflammatory causes. If non-inflammatory cause, treat accordingly and continue I-O therapy. Consider visual field testing, endocrinology consultation, and imaging.

Asymptomatic TSH elevation →

- Continue I-O therapy per protocol
- If TSH < 0.5 x LLN, or TSH > 2 x ULN, or consistently out of range in 2 subsequent measurements: include fT4 at subsequent cycles as clinically indicated; consider endocrinology consult

Symptomatic endocrinopathy →

- Evaluate endocrine function
- Consider pituitary scan

<u>Symptomatic with abnormal lab/pituitary scan:</u>

- Delay I-O therapy per protocol
- 1-2 mg/kg/day methylprednisolone IV or PO equivalent
- Initiate appropriate hormone therapy

<u>No abnormal lab/pituitary MRI scan but symptoms persist:</u>

- Repeat labs in 1-3 weeks / MRI in 1 month

→

<u>If improves (with or without hormone replacement):</u>

- Taper steroids over at least 1 month and consider prophylactic antibiotics for opportunistic infections
- Resume I-O therapy per protocol
- Patients with adrenal insufficiency may need to continue steroids with mineralocorticoid component

Suspicion of adrenal crisis (e.g. severe dehydration, hypotension, shock out of proportion to current illness →

- Delay or discontinue I-O therapy per protocol
- Rule out sepsis
- Stress dose of IV steroids with mineralocorticoid activity
- IV fluids
- Consult endocrinologist
- If adrenal crisis ruled out, then treat as above for symptomatic endocrinopathy

Patients on IV steroids may be switched to an equivalent dose of oral corticosteroids (e.g. prednisone) at start of tapering or earlier, once sustained clinical improvement is observed. Lower bioavailability of oral corticosteroids should be taken into account when switching to the equivalent dose of oral corticosteroids.

Skin Adverse Event Management Algorithm

Rule out non-inflammatory causes. If non-inflammatory cause, treat accordingly and continue I-O therapy.

Grade of Rash (NCI CTCAE v4)	Management	Follow-up
**Grade 1-2**<br>Covering ≤ 30% BSA*	• Symptomatic therapy (e.g. antihistamines, topical steroids)<br>• Continue I-O therapy per protocol	<u>If persists > 1-2 weeks or recurs:</u><br>• Consider skin biopsy<br>• Delay I-O therapy per protocol<br>• Consider 0.5-1.0 mg/kg/day methylprednisolone IV or oral equivalent. Once improving , taper steroids over at least 1 month, consider prophylactic antibiotics for opportunistic infections, and resume I-O therapy per protocol<br><u>If worsens:</u><br>• Treat as Grade 3-4
**Grade 3-4**<br>Covering >30% BSA; Life threatening consequences*^	• Delay or discontinue I-O therapy per protocol<br>• Consider skin biopsy<br>• Dermatology consult<br>• 1.0-2.0 mg/kg/day IV methylprednisolone IV or IV equivalent	<u>If improves to Grade 1:</u><br>• Taper steroids over at least 1 month and add prophylactic antibiotics for opportunistic infections<br>• Resume I-O therapy per protocol

Patients on IV steroids may be switched to an equivalent dose of oral corticosteroids (e.g. prednisone) at start of tapering or earlier, once sustained clinical improvement is observed. Lower bioavailability of oral corticosteroids should be taken into account when switching to the equivalent dose of oral corticosteroids.
*Refer to NCI CTCAE v4 for term-specific grading criteria.
^If SJS/TEN is suspected, withhold I-O therapy and refer patient for specialized care for assessment and treatment. If SJS or TEN is diagnosed, permanently discontinue I-O therapy.

Neurological Adverse Event Management Algorithm

Rule out non-inflammatory causes. If non-inflammatory cause, treat accordingly and continue I-O therapy.

Grade of Neurological Toxicity (NCI CTCAE v4)	Management	Follow-up
**Grade 1**<br>Asymptomatic or mild symptoms; Intervention not indicated	• Continue I-O therapy per protocol	Continue to monitor the patient.<br>If worsens:<br>• Treat as Grade 2 or 3-4
**Grade 2**<br>Moderate symptoms; Limiting instrumental ADL	• Delay I-O therapy per protocol<br>• Treat symptoms per local guidelines<br>• Consider 0.5 to 1.0 mg/kg/day methylprednisolone IV or PO equivalent	If improves to baseline:<br>•Resume I-O therapy per protocol when improved to baseline<br>If worsens:<br>• Treat as Grade 3-4
**Grade 3-4**<br>Severe symptoms; Limiting self-care ADL; Life-threatening	• Discontinue I-O therapy per protocol<br>• Obtain neurology consult<br>• Treat symptoms per local guidelines<br>• 1.0-2.0 mg/kg/day IV methylprednisolone IV or IV equivalent<br>• Add prophylactic antibiotics for opportunistic infections	If improves to Grade 2:<br>• Taper steroids over at least 1 month<br><br>If worsens or atypical presentation:<br>• Consider IVIG or other immunosuppressive therapies per local guidelines

Patients on IV steroids may be switched to an equivalent dose of oral corticosteroids (e.g. prednisone) at start of tapering or earlier, once sustained clinical improvement is observed. Lower bioavailability of oral corticosteroids should be taken into account when switching to the equivalent dose of oral corticosteroids.

16.3 Guidance for the Management of Infusion Reaction/Cytokine Release Syndrome

Precautions should be observed during the administration of APX005M and nivolumab. Emergency agents including oxygen, oral and endotracheal airways, intubation equipment epinephrine, antihistamines, and corticosteroids should be available and used if required at the Investigator's discretion.

Patients should be instructed that symptoms associated with cytokine release syndrome/infusion reaction can occur within 48 hours following the administration of the APX005M or nivolumab, and if such symptoms develop while they are at home, they should contact the Investigator and/or seek emergency medical care if appropriate.

- **Grade 2:** stop infusion and treat symptoms following guidance in Table 21. If symptoms resolve within two hours, the infusion may be restarted at 50% of the original infusion rate (e.g., from 100 mL/hr to 50 mL/hr).
- **Grade 3-4:** stop infusion and treat symptoms following guidance in Table 21.

Table 21 Infusion-related Reaction/Cytokine Release Syndrome Management Recommendations

<table>
<tr><th colspan="2">Suspected Cytokine Release Syndrome-related Toxicity</th><th>Recommended Treatment</th></tr>
<tr><td colspan="2">• Mild toxicity requiring symptomatic treatment only (e.g., fever, nausea, fatigue, headache, myalgia, malaise</td><td>• Vigilant supportive care
• Maintain adequate hydration
• Antipyretics, nonsteroidal anti-inflammatory drugs, antihistaminics, anti-emetics, analgesics as needed
• In case of mild symptoms persisting for > 24 hours assess for infections; empiric treatment of concurrent bacterial infections</td></tr>
<tr><td rowspan="2">• Symptoms or clinical findings requiring and responding to moderate intervention, such as:
○ O2 requirement < 40%
• Hypotension responsive to fluids ± low dose of 1 vasopressor (e.g., < 50 mg/min of phenylephrine)
○ CTCAE Grade 2 organ toxicity</td><td>No extensive comorbidities</td><td>• All of the above
• Monitor cardiac and other organ functions closely</td></tr>
<tr><td>• Extensive comorbidities
• Age ≥ 70 years</td><td rowspan="2">• All of the above
• Corticosteroids
• Consider tocilizumab</td></tr>
<tr><td colspan="2">• Symptoms or clinical findings requiring aggressive intervention, such as:
○ O2 requirement ≥ 40%
○ Hypotension requiring high dose or multiple vasopressors
○ Ventilator support required
○ CTCAE ≥ Grade 3 organ toxicity</td></tr>
</table>

16.4 Document Revision History

16.4.1 Key Revisions in Amendment 1

- Section 6.1: added "Grade 3 adverse events that compromise a major organ (e.g. Congestive Heart Failure) regardless of duration" per FDA request.
- Section 7.1: added "Glomerular Filtration Rate (GFR) ≥ 50 ml/min" per FDA request.
- Section 8.1.6: added safety monitoring post APX005M "After discharge, all patients should be monitored by a caregiver or by a healthcare professional for 24 hours after the first 2 infusions of APX005M and as clinically indicated thereafter" per FDA request.
- Table 10: added Thyroid function testing per FDA request
- Table 11: added Thyroid function testing per FDA request
- Section 9.2.1: added Thyroid function testing per FDA request
- Section 9.2.8: added Thyroid function testing per FDA request
- Section 16.3: added a new section titled "Guidance for the Management of Infusion Reaction/Cytokine Release Syndrome" per FDA request

16.4.2 Key Revisions in Amendment 2

- Synopsis: deleted "Reference therapy" to avoid confusion, and updated according to revisions in the protocol text
- Section 4.3.1: updated with latest clinical results with nivolumab per request of Scientific Review of leading site
- Section 4.5: updated rationale per request of Scientific Review of leading site
- Figure 1: clarified Phase II dose per request of IRB of record
- Section 7.1, inclusion criteria #5: replaced "CFR with "creatinine clearance ≥ 50 ml/min as measured by Cockcroft and Gault formula" for better comply of FDA's request
- Section 7.2: updated to avoid duplication and to be consistent with the rest of the protocol
- Section 7.3: deleted Protocol Deviation section as this is detailed in other documents
- Table 10: deleted Day 4 events under Cycle 3 and Subsequent cycles, as Day 4 visit is optional
- Section 11.2: Revised collection of blood samples for detection of anti-drug antibodies to be at before APX005M administration in Cycles 1-4, and then every other cycle and at the end of treatment.
- Table 12: deleted Table 12 as these are standard tests not specifically for safety analysis
- Section 16.4: added a new section "Document Revision History" for better track of document revisions.

16.4.3 Key Revisions in Amendment 3

- Inclusion/Exclusion Changes:
  - Synopsis and Section 7.1:
    - Language added to clarify requirement of baseline tumor tissue for trial participation, "A baseline tumor tissue sample is mandatory for enrollment."
  - Synopsis and Section 7.2:
    - Language added to exclusion 11 to allow the transient use of steroids, "Transient courses of steroids may be approved by the medical monitor on a case by case basis, dependent on dose and reason."
    - Language added indicating all screening laboratory values must be met without the use of transfusions or growth factors.
- Additions/Removals/Changes:
  - Synopsis:
    - Language regarding enrollment timeline updated to, "Target enrollment completion is within 24 months; however, enrollment will proceed until met or as determined by the study sponsor (PICI)."
  - Synopsis, Sections 5, 11 and 13:
    - Language added regarding exploratory endpoints/assays, as well as modified from "will" to "may," since analysis will depend on sample availability and amounts to perform analysis.
  - Section 6.1:
    - DLT criteria removed, "Failure to recover from a treatment-related AE to baseline or ≤ Grade 1 within 12 weeks of last dose of investigational product (except Grade 2 alopecia and Grade 2 fatigue)."
  - Sections 7 and 9:
    - Language updated to clarify staggered dosing parameters, "During Phase Ib, dosing of the first 3 patients of each cohort will be staggered by at least one week. If at one week, and for each of the 3 patients, there are no ongoing symptoms of cytokine release related to the infusion, and if no DLT occurs, subsequent patients to the cohort may be dosed without restriction."
  - Section 8.6.3:
    - Language added about steroid use, "A temporary course of steroids may be permitted once discussed and agreed upon by the medical monitor."
  - Section 9:
    - An EOS blood sampling for ADA added.

        - On days APX005M administered, time points for vital collection added at EOI, as well as at 2 and 5 hours thereafter.
        - Blood sampling for immune biomarkers at screening added.
        - A window of -7 days was added for disease assessments.
        - After cycle 2, a dosing window of ± 3 days was added for CXD1.
        - End of study visits added at 30 and 100 after last dose to assess patients for AEs, with a window of ± 7 days, physical exam, serum chemistry, vital, AE assessment and thyroid function test
    - Sections 11 and 12:
        - Language added regarding how samples may be used in the future.
        - Language removed regarding use of plasma for ADA assay.
        - Language removed to eliminate redundancy with the PICI0002 Laboratory Manual.
    - Section 12:
        - Requirements for vital sign collection updated to be in line with Table 10 and Section 9.
        - Requirements for physical examination updated.
    - Section 13:
        - Language regarding enrollment timelines removed to mirror updated language in the synopsis.
    - Table 10:
        - Language added regarding review of laboratory assessments, "All laboratory assessments should be should be reviewed prior to CXD8."
        - Window of ±10 minutes added for vital sign collection.
    - Table 12:
        - Table added to detail specific chemistry, hematology and urinalysis assessments, also referenced in Table 10, Table 11 and Section 9.
- Clarification of Document:
    - Document updated to address administrative changes and typographical errors.
    - Document updated to change "subject(s)" and "patient(s)" to "patient(s)" only, for consistency.
    - Document updated to change the naming of treatment cohorts to reflect the order in which study drugs are administered.
    - Synopsis, Sections 5, 9 and 11:
    - Updated to clarify time points for PK and ADA sampling.
    - Synopsis, Sections 7.1 and 9:

  - Updated to clarify blood sampling for immune biomarkers should only be collected at screening if patient has signed informed consent.
  - Updated to clarify the requirement of pre-treatment tumor tissue (fresh or archival) for trial participation.
- Sections 7 and 13:
  - Language added to clarify staggered dosing, “During Phase Ib, dosing of the first 3 patients of each cohort will be staggered by at least one week. If at one week, and for each of the 3 patients, there are no ongoing symptoms of cytokine release related to the infusion, and if no DLT occurs, subsequent patients to the cohort may be dosed without restriction.”
- Section 8.1.5:
  - Language added to clarify weight parameters for dosing, “Patient weight will be assessed at screening and day 1 of each cycle. Dose adjustments are not required unless the subject has a 10% change in comparison to their initial weight on Cycle 1, Day 1.”
- Section 9:
  - Instructions for APX005M pre-medication, as described in protocol Section 8.2, Table 2, included for clarity.
  - Corrected in line with Tables 10 and 11.
  - Language added to clarify that blood sampling for immune biomarkers should only be collected at screening if patient has signed informed consent.
- Section 12.5:
  - Updated to clarify SAE reporting guidelines.

16.4.4 Key Revisions in Amendment 4

Text revisions resulting from this amendment are incorporated in the synopsis and body of Protocol Amendment 4. Major changes to the protocol are summarized below.

- Eligibility criteria:
  - Synopsis and Section 7.1:
    - Moved the tumor biopsy sample criterion from #8 to #5.
    - Updated the AST/ALT criteria to be ≤ 5.0 x ULN for patients with liver metastases (#6).
  - Section 7.2:
    - Removed the contraception-related exclusion criterion (#17) as it is redundant with the inclusion criterion mandating use of contraception.

- Safety assessments:
  - Synopsis, Section 6.1, Section 13.2, and Section 13.6.1:
    - Revised definitions for DLTs, DLT-evaluable population, and DLT observation period for clarity.
  - Section 12.1:
    - Removed the language requiring patients to rest for at least 5 minutes in a sitting position.
  - Section 12.4:
    - Moved the tabular listing of laboratory assessments from the Section 9 (Study Procedures) to Section 12.4 (Laboratory Assessments) for clarity.
  - Section 12.5.2:
    - Added a new section for reporting of disease progression (titled, "Disease-related Events and/or Disease-related Outcomes Not Qualifying as Adverse Events or Serious Adverse Events) for clarity.
  - Section 12.5.4:
    - Updated the fax and telephone numbers for the PICI Pharmacovigilance Group due to updated point of contact information. In addition, clarified the process for SAE reporting to account for the updated PICI Pharmacovigilance Group point of contact information.
  - Section 12.5.5:
    - Added a section to clarify the regulatory reporting requirements for SAEs as this is a site responsibility and was not previously documented.
  - Section 12.5.6:
    - Corrected the reporting of pregnancies to indicate that they will be processed as SAEs.
- Toxicity management
  - Section 8.5.1 (Table 4):
    - Added a footnote to clarify that Day 1 toxicity management does not apply to Cycle 1 (but begins with Cycle 2 Day 1).
  - Section 8.5.5.1:
    - Removed this section (Criteria for Initiation of a New Cycle).
- Objectives/endpoints:
  - Synopsis and Section 5.2:
    - Revised endpoints to align with objectives.
- Study design
  - Synopsis, Section 6.1, Section 8, Section 8.6.1, and Section 13:

        - Removed reference to sequential enrollment in Phase 1b. Specified that Phase 1b enrollment may occur concurrently in Cohorts B2 and C1, and enrollment in Cohort C2 may begin once enrollment in Cohort C1 has been completed.
    - Section 6.1.4:
        - Added a new section to allow for treatment beyond unequivocal disease progression (per RECIST v1.1).
- Treatments:
    - Section 6.1, Section 8 (Table 2), Section 8.1.3, Section 8.1.4, Section 8.2.3, Section 8.2.4, and Section 8.4 (Table 3):
        - Added text to clarify that NP/Gem could be administered on a 3 weeks on, 1 week off or 2 weeks on, 1 week off schedule, depending on the presence or absence of toxicity.
    - Section 6.1, Section 8 (Table 2), Section 8.1.2, Section 8.2.2, and Section 8.4 (Table 3):
        - Added text to clarify that nivolumab doses should not be administered < 2 days apart, and nivolumab should not be administered on Day 15 if NP/Gem is held on Day 15.
    - Section 8.2.1:
        - Added text to clarify that for each cycle, APX005M should only be administered if patients received NP/Gem on Day 1.
- Study procedures:
    - Section 9:
        - Added Cycle 1 and Cycle 2, Day 15 weight assessments for non-APX005M arms (Table 12).
    - Section 9.1 (Patient Informed Consent):
        - Removed this section as informed consent has already been addressed by the inclusion criterion.
    - Section 9.2 (Procedures by Study Visit or Period):
        - Removed this section as it is redundant given the Schedule of Assessments.

In addition to the major changes described above, minor typos were corrected and editorial changes were made for clarity.

16.4.5 Key Revisions in Amendment 5

Text revisions resulting from this amendment are incorporated in the synopsis and body of Protocol Amendment 5. Major changes to the protocol are summarized below.

- Objectives and Endpoints/Exploratory:
  - Synopsis, Section 5.1.2.3, Section 5.2.2.3, Section 9, Section 11.2, Section 13:
    - Added: For Phase 2, evaluation of baseline and on-treatment microbiome profiles with treatment outcomes.
- Eligibility Criteria:
  - Synopsis and Section 7.2:
    - Added detail for Hashimoto syndrome: Patients with a history of Hashimoto syndrome within 3 years of the first dose of investigational agent, which resolved to hypothyroidism alone.
- Planned Sample Size:
  - Synopsis Section 6.1.2, Section 13.1:
    - Deleted: If 10% to 15% of Phase 1 patients are not DLT evaluable, then 27 patients may need to be enrolled. This text was removed, as additional patients may need to be enrolled to meet enrollment requirements.
- Statistical Methods and Planned Analyses:
  - Synopsis, Section 6.1, Section 13:
    - Revised text in definition of DLT-evaluable population to clarify 1 dose of APX005M required.
    - Added: For Phase 2, evaluation of baseline and on-treatment microbiome profiles with treatment outcomes.
- Investigational Plan:
  - Section 6.1, Section 9, Section 11.2:
    - Added language regarding timing and mandatory nature and of the on-treatment tumor biopsy, if medically feasible.
  - Section 6.1.1
    - Clarifications to enrollment timeframes for both Phase 1b and 2.
- Details of Study Treatment:
  - Section 8.1, Section 8.4, Section 8.5
    - Added text to permit delay of treatment, as allowed by the protocol, at the treating investigator’s discretion (e.g. toxicity, weather).
    - Added text to clarify APX005M doses should not be administered <2 weeks apart.

        - Added clarification around window for premedication treatment prior to APX005M.
        - Text added to clarify continuing treatment with other study treatments is allowed, with Medical Monitor approval, in the event APX005M is discontinued due to drug-related toxicity.

- Study Procedures:
    - Section 9:
        - Table 11 (Arms Including APX005M, Phase 1b):
            - Added pre-infusion vital signs.
            - Added language regarding timing and mandatory nature and of the on-treatment tumor biopsy, if medically feasible.
        - Table 12 (Arms Including APX005M, Phase 2):
            - Added pre-infusion vital signs.
            - Removed PK collection during Cycle 1, Days 4, 8 and 15.
            - Removed biomarker collection during Cycle 1, Days 4 and 8.
            - Removed biomarker collections after Cycle 4.
            - Added stool collection at screening and on-treatment for microbiome profiling.
            - Added language regarding timing and mandatory nature and of the on-treatment tumor biopsy, if medically feasible.
        - Table 13 (Arms Not Including APX005M, Phase 2):
            - Added pre-infusion vital signs.
            - Removed biomarker collections after Cycle 4.
            - Added stool collection at screening and on-treatment for microbiome profiling.
            - Added language regarding timing and mandatory nature and of the on-treatment tumor biopsy, if medically feasible.

In addition to the major changes described above, minor typos were corrected and editorial changes were made for clarity.

16.4.6 Key Revisions in Amendment 6

Text revisions resulting from this amendment are incorporated in the synopsis and body of Protocol Amendment 6. Major changes to the protocol are summarized below.

- Objectives and Endpoints:
  - Synopsis:
    - For Phase 2 exploratory objective #4, the specification of "Phase 2" for evaluation of baseline and on-treatment microbiome profiles was removed.
  - Synopsis and Section 5.2.2.2:
    - For Phase 2 secondary endpoint #5, assessment of "unacceptable toxicities in Phase 2" was added.
- Study Design:
  - Synopsis and Section 6.1:
    - Figure 1 Study Flow Chart was updated to reflect selection of recommended phase 2 dose.
    - Added criteria for "unacceptable toxicity", defined as any ≥ Grade 3 toxicity that is not treatment-related but not related to the natural progression of the tumor and occurs during the Phase 2 period.
- Study Duration:
  - Synopsis and Section 6.1.1:
    - Changed the duration of additional follow-up in Phase 2 from 6 to 12 months.
- Discontinuation/Withdrawal of Patients
  - Section 7.3.1:
    - Added this subheader (Discontinuation of Study Drug) for clarity. Also, specified that the primary reason for study drug discontinuation should be documented on the appropriate eCRF page.
  - Section 7.3.2:
    - Renamed this subsection as "Withdrawal/Discontinuation of Patients" (instead of "Withdrawal and Removal of Patients) for clarity. Added text to describe withdrawal/discontinuations of patients.
  - Section 7.5:
    - Added this section to describe lost to follow-up.
- Study Treatments and Toxicity Management:
  - Section 7.4, Section 9 (Table 11, Table 12, and Table 13), Section 11.2:
    - Changed "end of study" to "end of treatment" for clarity.

- Section 8.1.1 and Section 8.1.2:
  - Revised the storage and stability text for APX005M and nivolumab to be consistent with the Pharmacy Manual.
- Section 8.1.1, Section 8.1.3, Section 8.1.4, Section 8.2.1, Section 8.2.3, and Section 8.2.4:
  - Specified that patients may only receive a delayed dose of APX005M on Day 10 if they have received NP/Gem on Day 8.
  - Specified that patients whose Day 8 dose of NP/Gem is delayed due to toxicity may receive the dose on Day 22, but they must have received APX005M on Day 3.
- Section 8.2:
  - Reduced change in body weight (for dose adjustments) from 10% to ≥ 5%.
- Section 8.5:
  - Added a statement to clarify that if NP and/or Gem related toxicity leads to discontinuation of the chemotherapy, treatment with APX005M and nivolumab or either agent alone may be continued with agreement of the Medical Monitor.

- Study Procedures:
  - Section 9:
    - Added text to clarify the timing of study assessments for treatment delays.
    - Table 11 (Arms Including APX005M, Phase 1b):
      - Added follow-up columns (D30, D100, and survival) for clarity.
      - Clarified the timing of study assessments for APX005M treatment delays in footnote "b".
      - Added footnote "h" to clarify the AE collection period, particularly for follow-up.
      - Revised footnote "k" for clarity.
      - Revised footnote "m" to specify that the tumor markers, CA19-9 and CEA, will be collected if performed as part of standard of care.
      - Added missing footnotes "q" and "s".
    - Table 12 (Arms Including APX005M, Phase 2)
      - Added follow-up columns (D30, D100, and survival) for clarity.
      - Clarified the timing of study assessments for APX005M treatment delays in footnote "b".

        - Added footnote "h" to clarify the AE collection period, particularly for follow-up.
        - Revised footnote "l" for clarity.
        - Revised footnote "o" to specify that tumor markers, CA19-9 and CEA, are required at Cycle 1 Day 1 for all treatment arms, and if the result of either of these markers are not clinically significant at Cycle 1 Day 1, further collection will not be required.
        - Added footnotes "t" and "u".
    - Table 13 (Arms Not Including APX005M, Phase 2)
        - Added follow-up columns (D30, D100, and survival) for clarity.
        - Revised footnote "f" for clarity.
        - Added footnote "g" to clarify the AE collection period, particularly for follow-up.
        - Revised footnote "i" for clarity.
        - Revised footnote "k" to specify that tumor markers, CA19-9 and CEA, are required at Cycle 1 Day 1 for all treatment arms, and if the result of either of these markers are not clinically significant at Cycle 1 Day 1, further collection will not be required.
        - Revised footnote "p" for clarity.

- Safety Assessments:
    - Section 12.5.1:
        - Clarified the collection period for AEs and SAEs.
    - Section 2.5.2 and Section 2.5.2.1:
        - Clarified the definitions of AEs and SAEs.
    - Section 12.5.2.2:
        - Clarified the assessment of events as infusion-related reactions or cytokine release syndrome.
    - Section 12.5.2.3:
        - Added a definition for immune-mediated AEs.
    - Section 12.5.5:
        - Clarified SAE reporting.
- Statistical Analysis and Sample Size:
    - Synopsis and Section 6.1.2: Corrected the total sample size to show "approximately 117" instead of "120" patients.

- Synopsis, Section 6.1, Section 13, Section 13.1.2, Section 13.2:
  - For 1-year OS rate analysis, text was added to indicate that a 1-sided 1-sample Z test will also be performed and that the goal is to compare the survival probability at 1 year to the historical value of 0.35.

In addition to the major changes described above, minor typos were corrected and editorial changes were made for clarity.

16.4.7 Key Revisions in Amendment 7

Text revisions resulting from this amendment are incorporated in the synopsis and body of Protocol Amendment 7. Major changes to the protocol are summarized below.

- Study Procedures
  - Sections 6.1, 9 and 11.2
    - Added option for tissue collection at the time of disease progression, for patients with prolonged stable disease, as well as ad hoc tissue collections with Medical Monitor approval
    - Added language indicating the first dose of study drug must be administered within 3 days of randomization, per a previously-approved Note to File
  - Sections 6.1.1, 7.3.2 and 9
    - Clarified duration of survival follow-up
    - Revised frequency of follow-up for survival and new anti-cancer therapy status from Q6M to Q3M with the addition of ad hoc collections
  - Section 8.9.3
    - Added language permitting the transient use of steroids to control contrast agent allergies for radiographic studies as an exception to the Prohibited Medications
  - Section 9
    - Revised window for Day 100 Follow-up Visit from ± 7 days to ± 14 days
    - Added pregnancy test to the Phase 2 Day 30 Follow-Up Visit to align with the Phase 1b safety assessments
    - Clarified tumor marker collection requirements for Phase 2
  - Sections 9 and 10
    - Added language clarifying the collection requirements for disease assessments after treatment discontinuation and during treatment beyond radiological progression, per a previously-approved Note to File

  - Sections 9, 11.1.1, 11.2.3
    - Clarified PK and ADA collection required at EOT
  - Sections 9 and 11.2
    - Clarified immune biomarker collection required at EOT
    - Added immune biomarker collections for patients on treatment over 12 months, permitting blood draws at 12 months and Q6M thereafter
- Safety Assessments:
  - Sections 9 and 12.5.1, 12.5.3 and 12.5.5
    - Added language clarifying the collection and reporting periods for AEs, AESIs and SAEs
  - Section 12.5.2.2
    - Removed text defining infusion-related reaction versus cytokine release syndrome to maintain reporting consistency over life of study
    - Updated reporting language for Infusion-Related Reactions and Cytokine Release Syndrome to clarify all underlying symptom(s) must be reported as individual AEs in addition to the applicable syndrome(s)
  - Section 12.5.2.3
    - Removed text defining Immune-mediated Adverse Events to maintain reporting consistency over life of study
- Statistical Methods and Planned Analyses:
  - Section 13.1.2
    - Removed language specifying an intent-to-treat approach will be used for Phase 2 for alignment with the definition of the efficacy population indicated in the Synopsis, Section 13 and the Statistical Analysis Plan
  - Section 13.7
    - Added language allowing the Sponsor to conduct up to three interim analyses of safety and efficacy during the study

In addition to the major changes described above, minor typos were corrected and editorial changes were made for clarity.

Statistical Analysis Plan for

Protocol Number: PICI0002

Protocol Title: Open-label, Multicenter, Phase 1b/2 Clinical Study to Evaluate the Safety and Efficacy of CD40 Agonistic Monoclonal Antibody (APX005M) Administered Together with Gemcitabine and nab-Paclitaxel with or without PD-1 Blocking Antibody (Nivolumab) in Patients with Previously Untreated Metastatic Pancreatic Adenocarcinoma

**IND Number:**	132683
**Name of Products:**	APX005M (experimental)<br>Nivolumab (experimental)<br>Gemcitabine (standard of care)<br>nab-Paclitaxel (standard of care)
**Phase of Development:**	1b/2
**Indication:**	Previously untreated metastatic pancreatic cancer
**Sponsor:**	Parker Institute for Cancer Immunotherapy<br>1 Letterman Drive<br>Suite D3500<br>San Francisco, CA 94129
**Date Final:**	Version 1: 26 Sep 2019<br>Version 2: 14 Feb 2020

-CONFIDENTIAL-

This document and its contents are the property of and confidential to Parker Institute for Cancer Immunotherapy. Any unauthorized copying or use of this document is prohibited.

SPONSOR APPROVAL PAGE

DocuSigned by:
Christopher Cabanski
Signer Name: Christopher Cabanski
Signing Reason: I approve this document
Signing Time: 2/14/2020 | 1:10:30 PM PST
73CCABA749D14634B5D6314D83F02F85

Christopher Cabanski, PhD
Director, Biostatistics

Date: 2/14/2020 | 1:10:58 PM PST

DocuSigned by:
Ramy Ibrahim
Signer Name: Ramy Ibrahim
Signing Reason: I approve this document
Signing Time: 2/16/2020 | 4:43:42 AM PST
E5202C4F8B354F43850870D1D709EC1C

Ramy Ibrahim, MD
Chief Medical Officer

Date: 2/16/2020 | 4:43:45 AM PST

1 STATISTICAL ANALYSIS PLAN AMENDMENT RATIONALE

Version 2: 14 Feb 2020

The Statistical Analysis Plan was amended to add details about a second interim analysis of the Phase 2 portion of the study. Additional minor changes were made to improve clarity and consistency. No changes to the study endpoints, population definitions, or statistical methods were made.

2 TABLE OF CONTENTS

1 STATISTICAL ANALYSIS PLAN AMENDMENT RATIONALE 3
2 TABLE OF CONTENTS 4
3 INTRODUCTION 6
4 STUDY DESIGN 6
4.1 Protocol Synopsis 8
4.2 Study Objectives 8
4.2.1 Phase 1b 8
4.2.2 Phase 2 8
4.3 Study Endpoints 9
4.4 Determination of Sample Size 9
4.4.1 Phase 1b 9
4.4.2 Phase 2 10
4.5 Analysis Timing 10
5 STUDY CONDUCT 10
5.1 Randomization Details 10
5.2 Blinding 12
5.3 Data Monitoring 12
5.3.1 Early Termination Rules for Unacceptable Toxicity in Phase 2 12
6 STATISTICAL METHODS 13
6.1 Analysis Populations 13
6.1.1 Safety Population 13
6.1.2 DLT-evaluable Population 13
6.1.3 Efficacy Population 14
6.2 Analysis of Study Conduct 14
6.3 Analysis of Treatment Group Comparability 14
6.4 Efficacy Analysis 15
6.4.1 Comparisons of Interest 15
6.4.2 Type I Error Management 15
6.4.3 Covariate Adjustment 15
6.4.4 Primary Efficacy Endpoint 15
6.4.5 Secondary Efficacy Endpoints 16

6.4.5.1 Objective Response Rate 16
6.4.5.2 Duration of Response 16
6.4.5.3 Disease Control Rate 17
6.4.5.4 Progression-free Survival 17
6.4.6 Exploratory Efficacy Endpoints 17
6.4.7 Subgroup Analyses 17
6.4.8 Sensitivity Analyses 18
6.5 Safety Analysis 18
6.5.1 Exposure to Study Medication 19
6.5.2 Adverse Events 19
6.5.3 Laboratory Data 20
6.5.4 Vital Signs 20
6.6 Missing Data 20
6.6.1 Missing and Partial Missing Adverse Event Dates 20
6.6.2 Missing and Partial Missing Death Dates 20
6.7 Interim Analyses 21
7 DIFFERENCES COMPARED TO PROTOCOL 22
8 REFERENCES 23
9 APPENDICES 24
9.1 Protocol Synopsis 24

LIST OF TABLES

Table 1 Probability of Failing to Accept a Dose Level at Different Event Rates 9
Table 2 Phase 1b Treatment Assignment 11
Table 3 Phase 2 Design 11
Table 4 Bayesian Termination Rules 12

LIST OF FIGURES

Figure 1 Study Flow Chart 6

3 INTRODUCTION

The purpose of this document is to provide details of the planned analyses for Protocol PICI0002. The analyses specified in this document supersede the high-level analysis plan described in the protocol. Statistical analyses will be performed consistent with the principles of the ICH/FDA Guidance for Industry E9 Statistical Principles for Clinical Trials.

4 STUDY DESIGN

PICI0002 is a multi-center, open label, Phase 1b/2 study of the immunotherapy agents APX005M and nivolumab, in combination with Gemcitabine (Gem) and nab-Paclitaxel (NP) in patients with previously untreated metastatic pancreatic adenocarcinoma.

Phase 1b will involve 4 sequential treatment cohorts, and Phase 2 will involve randomization to 3 treatment arms, as shown in Figure 1.

Figure 1 Study Flow Chart

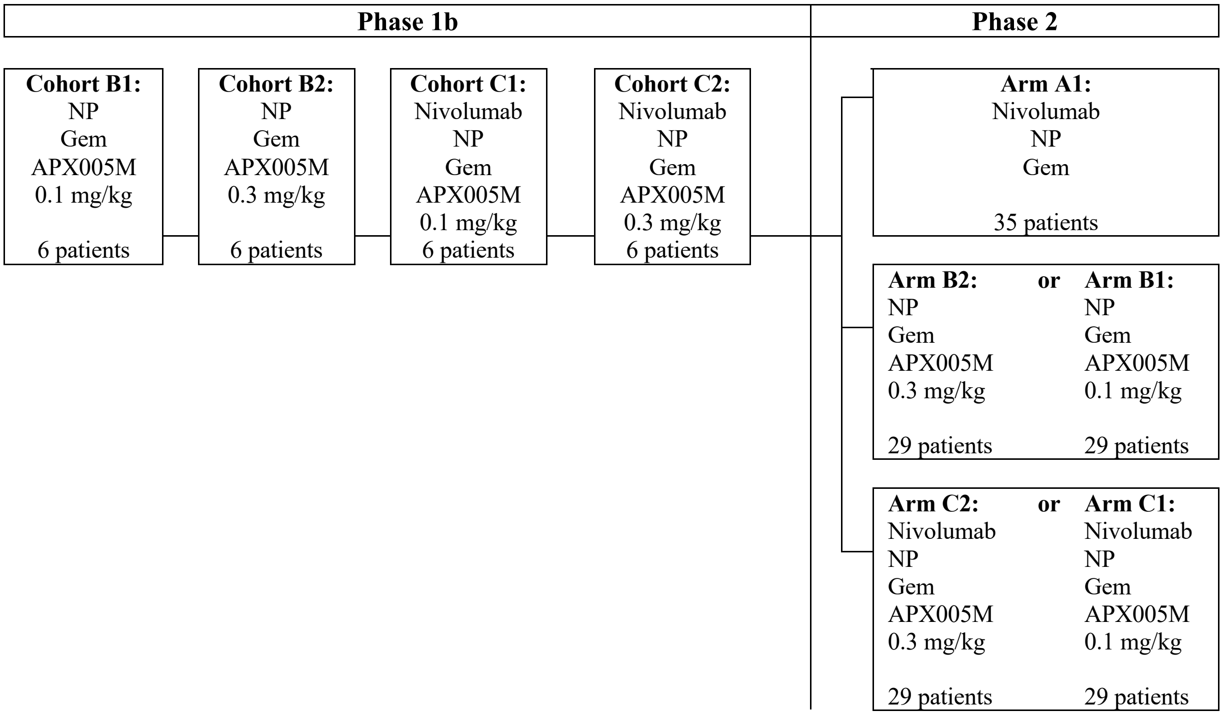

Phase 1b

In the Phase 1b portion of the study, 4 treatment cohorts will be evaluated sequentially for feasibility and safety. Each cohort of the study will have 6 DLT-evaluable patients, defined as patients who experienced a Dose-Limiting Toxicity (DLT) or who completed the DLT observation period, i.e. received at least 2 doses of NP/Gem and 1 dose of APX005M during

Cycle 1. Patients who do not experience a DLT and who do not complete the DLT observation period will be replaced.

In general, a DLT is defined as: (1) any Grade 3 or higher non-hematologic or Grade 4 hematologic toxicity occurring during the DLT observation period that is considered to be at least possibly related to APX005M and/or nivolumab, or (2) an exacerbation of known NP/Gem toxicity that is at least possibly related to APX005M and/or nivolumab. A more comprehensive definition of a DLT can be found in the protocol. The DLT observation period is defined as the time of first administration of investigational agents until Cycle 2 Day 1.

Dose escalation will proceed if 1 or fewer DLT-evaluable patients experience a DLT during this observation period. Dose escalation will cease if 2 or more DLT-evaluable patients in a cohort experience a DLT.

On the basis of discussions among the site PIs, Sponsor, and other stakeholders, concerning feasibility, safety, clinical and immune pharmacodynamic (PD) effects (totality of available data), the recommended Phase 2 dose is defined by the highest APX00M dose with < 2 DLT in 6 DLT-evaluable patients, unless the totality of available data suggests a lower APX005M dose.

Randomized Phase 2

Once the recommended Phase 2 dose (RP2D) of APX005M in combination with nivolumab from Cohort C in the Phase 1b portion of the study is determined, the randomized Phase 2 portion will commence. Patients will be randomized to Arm A1, Arm B2, or Arm C2, or to Arm A1, Arm B1, or Arm C1, if Cohort B2 and/or C2 are deemed unsafe in Phase 1b. Note that the APX005M dose must be the same in Arms B and C, regardless of whether a higher APX005M dose was determined to be safe in Arm B. For each regimen, efficacy will be evaluated by comparing the 1-year overall survival (OS) rate to the historical value for NP/Gem. A total of approximately 93 patients will be randomized in Phase 2 (35 Arm A1, 29 Arm B2, 29 Arm C2).

All patients will be followed up for survival status until death or a maximum of 5 years. Once a patient is in follow-up, follow up can be obtained with a clinic visit or a phone call approximately every 6 months.

It is common for randomized Phase 2 studies that test the addition of an experimental agent to a standard of care regimen to include the standard of care arm. However, the setting for this study is unique. NP/Gem, the standard of care regimen, was reported recently in a very similar patient population, and the 1-year OS rate was estimated with extremely high precision (i.e., 1-year OS rate was 35% with 95% CI 30%-39%) based on 431 treated patients (Von Hoff et al.). With hundreds of pancreatic patients treated with NP/Gem since that report, experts in this

field agree that the 1-year OS rate estimate appears to be very robust. Thus, this study will not include a standard of care arm.

The 12 DLT-evaluable patients who were enrolled in Phase 1b at the recommended Phase 2 doses (6 on Arm B and 6 on Arm C) will be included in the efficacy evaluation and 93 additional patients will be randomized in Phase 2, for a total sample size of 105 patients (35 per treatment arm).

4.1 Protocol Synopsis

The Protocol Synopsis is provided in Section 9.1.

4.2 Study Objectives

This study will be conducted in two phases, each with its own objectives.

4.2.1 Phase 1b

Primary Objectives:

1. To determine the feasibility, safety, and DLTs of each treatment cohort.
2. To determine the recommended Phase 2 dose of APX005M when combined with NP/Gem.
3. To determine the recommended Phase 2 dose of APX005M when combined with nivolumab/NP/Gem.

Secondary Objectives:

1. To determine objective response rate (ORR) and duration of responses (DOR) of each treatment cohort.

Exploratory Objectives:

1. To assess the pharmacokinetics (PK) of APX005M in Cycles 1 to 4.
2. To assess immune pharmacodynamic effects of each treatment cohort, in both blood and tumor tissue.

4.2.2 Phase 2

Primary Objectives:

1. To estimate the OS of each treatment arm.
2. To compare 1-year OS rate of each treatment arm to the historical rate for NP/Gem.

Secondary Objectives:

1. To determine the ORR, disease control rate (DCR), DOR, and progression-free survival (PFS) of each treatment arm.
2. To further characterize the feasibility and safety of each treatment arm.

Exploratory Objectives:

1. To assess the PK of APX005M in Cycles 1 to 4 (Arms B and C).
2. To assess immune pharmacodynamic effects of each treatment arm, in both blood and tumor tissue.
3. To assess associations between immune biomarkers and clinical outcomes.
4. To evaluate baseline and on-treatment microbiome profiles.
5. To construct multivariable linear models to dissect the pharmacodynamic effects of APX005M and nivolumab on immune biomarkers.

4.3 Study Endpoints

The study endpoints are listed in the Protocol Synopsis (Section 9.1).

4.4 Determination of Sample Size

4.4.1 Phase 1b

Assuming 4 treatment cohorts will be evaluated sequentially for feasibility, safety, and dose-limiting toxicities, 24 DLT-evaluable patients will be enrolled (6 per cohort). Patients who do not experience a DLT and who do not complete the DLT observation period will be replaced.

The Phase 1b sample size was determined by practical considerations and was not based on statistical power calculations. Six patients dosed in each group was deemed sufficient to characterize the feasibility, safety, and DLTs of each cohort. Based on this sample size, Table 1 provides the probability of failing to accept a cohort as safe, defined as ≥ 2 DLTs in 6 treated patients, assuming different DLT rates.

Table 1 Probability of Failing to Accept a Dose Level at Different Event Rates

DLT Event Rate	1%	10%	20%	25%	33%	50%
Probability [a]	0.001	0.11	0.34	0.47	0.64	0.89

DLT = dose-limiting toxicity

[a] Assumes $P(X \geq 2)$ where X is a binomial random variable with sample size n = 6 and p = DLT event rate.

4.4.2 Phase 2

The 12 DLT-evaluable patients who were enrolled in Phase 1b at the recommended Phase 2 doses (6 on Arm B and 6 on Arm C) will be included in the efficacy evaluation and approximately 93 additional patients will be randomized in Phase 2, for a total sample size of 105 patients (35 per treatment arm). This is a screening study, such that for each treatment arm, the 1-year OS rate will be estimated and compared with a historical value of 35% for NP/Gem (Von Hoff et al.). The study is not powered to detect a meaningful difference in OS among the 3 arms, since these are novel experimental arms and OS is unknown.

The null hypothesis is a 1-year OS rate of 35% and the alternative hypothesis is a 1-year OS rate of 58%. The 1-year OS rate is estimated by the Kaplan-Meier method. A sample size of 35 patients on each arm provides 88% power to test this hypothesis, using a 1-sided one-sample Z test with 5% type I error rate, assuming a minimum of 1 year of follow-up for each patient. Moreover, the sample size of 35 patients on each arm, provides 81% power to statistically test the null hypothesis versus a slightly more conservative alternative hypothesis that the 1-year OS rate is 55%, given the same design assumptions.

These calculations assume that 105 patients (35 patients x 3 arms) will be enrolled. There is no assumption about the duration of patient enrollment, only that there will be a minimum of 1 year of follow-up for each patient.

4.5 Analysis Timing

The database lock for analysis of the Phase 2 primary endpoint of 1-year overall survival (OS) rate will occur approximately one year after the last patient is randomized. No changes to the SAP will be allowed at the time of or subsequent to database lock.

Analysis of the Phase 1b endpoints may be performed prior to the time of primary analysis. These results may be presented and/or published prior to the primary analysis.

The study will formally end once all patients have been followed for survival status until death or a maximum of 5 years, withdrawal of consent, or loss to follow-up. A survival analysis of long-term follow-up may be performed after the primary analysis has been completed.

5 STUDY CONDUCT

5.1 Randomization Details

In Phase 1b, patients will be enrolled sequentially into 4 cohorts (B1, B2, C1, and C2) as summarized in Table 2. The Phase 1b portion of the study is non-randomized. Enrollment in Cohorts B2 and C1 may occur concurrently.

Table 2 Phase 1b Treatment Assignment

Arm	Regimen	Number of DLT-Evaluable Patients
B1	NP/Gem/APX005M 0.1 mg/kg	6
B2	NP/Gem/APX005M 0.3 mg/kg	6
C1	Nivolumab/NP/Gem/APX005M 0.1 mg/kg	6
C2	Nivolumab/NP/Gem/APX005M 0.3 mg/kg	6

DLT = dose-limiting toxicity; Gem = gemcitabine; NP = nab-paclitaxel

Once the RP2D of APX005M in combination with nivolumab from Cohort C in the Phase 1b portion of the study is determined, the randomized Phase 2 portion will commence. Patients will be randomized to Arm A1, Arm B2, or Arm C2, or to Arm A1, Arm B1, or Arm C1, if Cohort B2 and/or C2 are deemed unsafe in Phase 1b. Note that the APX005M dose must be the same in Arms B and C, regardless of whether a higher APX005M dose was determined to be safe in Arm B.

A total of 105 patients will be evaluated in the Phase 2 portion of the study, including 12 DLT-evaluable patients from Phase 1b (i.e., 6 patients on B1 and 6 patients on C1 or 6 patients on B2 and 6 patients on C2). The remaining 93 patients will be randomized and treated only in Phase 2. In step 1 of randomization, 12 of the 93 new patients will be randomized to the 3 arms in a 4:1:1 ratio in Arms A1, B2, and C2 (or A1, B1, and C1), to achieve balance in the total number of patients enrolled on the arms (since Arm A1 does not accrue in Phase 1b, more patients need to be enrolled in Arm A1). In step 2 of randomization, 81 patients will be randomized to Arms A1, B2, and C2 (or A1, B1, and C1) in a 1:1:1 allocation. The randomization design is outlined in Table 3.

Table 3 Phase 2 Design

Arm	Regimen	Phase 1b	Phase 2		Total
			Step 1	Step 2	
		Number of patients	Number of patients	Number of patients	Number of patients
A1	Nivolumab/NP/Gem	0	8	27	35
B2 [a]	NP/Gem/APX005M 0.3 mg/kg	6	2	27	35
C2 [a]	Nivolumab/NP/Gem/APX005M 0.3 mg/kg	6	2	27	35

[a] Or B1 and C1, if either B2 or C2 is not tolerable.
Gem = gemcitabine; NP = nab-paclitaxel

Randomization will be managed by the Parker Institute for Cancer Immunotherapy (PICI), using an interactive voice response system (IVRS). The randomization is not stratified by baseline patient or tumor characteristics.

5.2 Blinding

This is an open-label study with no blinding.

5.3 Data Monitoring

The study will be closely monitored, and data will be reviewed on an ongoing basis. In order to ensure the safety and well-being of participating patients, as well as the validity of data during the study, a Data Review Team (DRT) will review the safety and further emerging data on a regular basis. The DRT consists of members from the Sponsor, the overall Principal Investigator (PI), the lead statistician, and all active PIs. The DRT will adjudicate DLTs relevant for the treatment and will decide by consensus on dose escalation, dose de-escalation, prolongation of the DLT observation period, suspension of enrollment based on safety, PK, or possibly pharmacodynamic data, and will recommend the dose level for the Phase 2 portion.

5.3.1 Early Termination Rules for Unacceptable Toxicity in Phase 2

A Bayesian rule will be employed to monitor toxicity during Phase 2. A minimally informative beta (0.5, 2.5) prior has been assumed, which is information that is equivalent to ½ the weight of 1 DLT in 6 patients treated, the definition of a safe dose in Phase 1b. For each treatment arm, if the number of patients with an unacceptable toxicity (defined the Section 6.1 of the study protocol) is greater than or equal to the number in Table 4, then termination of that particular treatment arm will be considered, as it is likely that the toxicity rate is >30%, as noted by the Bayesian posterior probabilities. This rule is intentionally conservative early in the enrollment phase.

Table 4 Bayesian Termination Rules

Rules for Unacceptable Toxicity Rate >30%					
Patients treated on an arm	10	15	20	25	30
Patients with unacceptable toxicity	4	6	9	11	13
Posterior Probability [toxicity rate >30%]	0.61	0.69	0.87	0.88	0.90
Action	Consider termination of arm, re-evaluate study design.				

6 STATISTICAL METHODS

Summary statistics will be presented by treatment arm. For continuous variables, data will be summarized with the number of patients (N), mean, standard deviation, median, minimum, and maximum by treatment arm. For categorical variables, data will be tabulated with the number and proportion of patients for each category by treatment arm.

6.1 Analysis Populations

6.1.1 Safety Population

The safety population consists of all patients who received at least 1 dose of any study drug. This is the population for the analyses of safety. A subset of the safety population is the DLT-evaluable population.

For the safety analyses, patients will be analyzed according to the treatment regimen actually received. Using a conservative approach:

- Phase 1b patients who receive at least one dose of nivolumab but do not receive APX005M will be analyzed under Arm C1. Phase 2 patients who receive at least one dose of nivolumab but do not receive APX005M will be analyzed under Arm A1.
- Phase 1b patients who receive at least one dose of APX005M but do not receive nivolumab will be analyzed under Arm B1 or B2, depending on the highest dose level of APX005M received. Phase 2 patients who receive at least one dose of APX005M but do not receive nivolumab will be analyzed under Arm B2 (the dose level selected for Phase 2 enrollment).
- Phase 1b patients who receive at least one dose of both APX005M and nivolumab will be analyzed under Arm C1 or C2, depending on the highest dose level of APX005M received. Phase 2 patients who receive at least one dose of both APX005M and nivolumab will be analyzed under Arm C2 (the dose level selected for Phase 2 enrollment).
- Patients who receive at least one dose of NP/Gem but do not receive APX005M or nivolumab will be analyzed under the treatment arm assigned at enrollment (Phase 1b) or randomization (Phase 2).

6.1.2 DLT-evaluable Population

The DLT-evaluable population consists of patients who (1) were enrolled in Phase 1b, (2) received at least 2 doses of NP/Gem and 1 dose of APX005M during Cycle 1, and (3) completed the DLT observation period (i.e. from the time of first administration of study

intervention until Cycle 2 Day 1). Alternatively, patients who did not complete the DLT observation period due to a DLT event will also be considered DLT evaluable.

The DLT-evaluable population is the population for analyses of Phase 1b efficacy and DLTs. Patients will be grouped according to the treatment arm assigned at enrollment, regardless of the treatment actually received.

6.1.3 Efficacy Population

The efficacy population consists of (1) all patients who were randomized in Phase 2 and received at least 1 dose of any study drug and (2) the 12 DLT-evaluable patients (6 on Arm B and 6 on Arm C) who were enrolled in Phase 1b at the recommended Phase 2 dose. The efficacy population is the population for the primary analyses of efficacy in Phase 2. Patients will be grouped according to the treatment arm assigned at enrollment/randomization, regardless of the treatment actually received.

6.2 Analysis of Study Conduct

The number of patients enrolled in Phase 1b and randomized in Phase 2 will be tabulated by treatment arm. Patient disposition (e.g. the number of patients enrolled/randomized, receiving at least one dose of study drug) and time on study will be tabulated by treatment arm and may be represented graphically (e.g. swim lane plot). Reasons for premature discontinuation from study treatment and reasons for premature discontinuation from the study, including the 5-year follow-up period, will be summarized.

Feasibility, defined by the number of Phase 1b patients who complete the DLT observation period and receive the intended therapy without delays or dose modification, will be described for each treatment arm.

6.3 Analysis of Treatment Group Comparability

Demographic and baseline characteristics, including but not limited to age, sex, race, ethnicity, Eastern Cooperative Oncology Group (ECOG) performance status at baseline, cancer location (pancreas body, head, or tail) and cancer stage at initial diagnosis and enrollment will be summarized by treatment arm using descriptive statistics for all enrolled/randomized patients.

The baseline value of any variable will be defined as the last available value recorded prior to the first administration of study drug.

Previous and concomitant cancer therapy will also be summarized, including radiotherapy and surgery, as well as subsequent anti-cancer therapy. Previous and concurrent diseases and medications will be listed.

6.4 Efficacy Analysis

Phase 2 efficacy analyses will be conducted on the efficacy population (see Section 6.1.3), with patients grouped according to the treatment assigned at randomization. Phase 1b efficacy analyses will be conducted on the DLT-evaluable population.

Efficacy summaries will include data from patients who discontinued study drug early but continued with study assessments and may include data collected at unscheduled visits, early termination visits, or follow-up visits.

6.4.1 Comparisons of Interest

The 1-year OS rate and 1-sided 95% confidence interval will be calculated for each treatment arm, to determine whether the lower bound of the confidence interval (CI) excludes the assumed historical value for NP/Gem of 35%.

This study is not powered for statistical comparisons between arms.

6.4.2 Type I Error Management

Due to the exploratory nature of this study, no control of type I error will be applied for any of the endpoints.

6.4.3 Covariate Adjustment

Unless otherwise noted, analyses of primary and secondary efficacy endpoints will not be adjusted for additional covariates.

6.4.4 Primary Efficacy Endpoint

There is no primary efficacy endpoint for Phase 1b. The Phase 2 primary efficacy endpoint is the 1-year OS rate in each treatment arm. OS is defined as the time from initiation of study therapy to date of death due to any cause. Patients who are not reported as having died at the time of analysis will be censored at their most recent contact date they were known to be alive. Patients who do not have post-baseline survival information will be censored at the date of randomization. See Section 6.6.2 for handling of missing or partial death dates.

OS will be estimated by the Kaplan-Meier method for each treatment arm. For each treatment arm, the following parameters and analyses will be provided: Kaplan-Meier product-limit estimates of the OS distribution functions, the total number of patients, the total censored, the total deaths, the OS time (median and its 95% CI; 25th and 75th percentiles), and the survival rates at monthly intervals (i.e. 3, 6, 9, 12, etc. months).

The 1-year OS rate and 1-sided 95% confidence interval will be calculated for each treatment arm, to determine whether the lower bound of the confidence interval (CI) excludes the assumed historical value for NP/Gem of 35%.

A 1-sided one-sample Z test will also be conducted. The goal is to compare the survival probability at time t to the historical value. The null hypothesis is H_0: $S(t) \leq s^*$ at time t. The alternative hypothesis is H_1: $S(t) > s^*$, a one-sided test.

The Z test is:

$$\frac{\hat{S}(t) - s^*}{\widehat{SE}(\hat{S}(t))}$$

where $\hat{S}(t)$ and $\widehat{SE}$ are sample estimates. For this study, $t = 1$ year, $\hat{S}(t)$ = estimated 1-year OS probability from the Kaplan-Meier analysis, $\widehat{SE}(\hat{S}(t))$ = standard error of $\hat{S}(t)$ and null hypothesis $s^* = 0.35$.

A survival follow-up analysis may be performed based on more mature data.

6.4.5 Secondary Efficacy Endpoints

Secondary efficacy endpoints for Phase 1b include ORR and DOR. The Phase 1b efficacy endpoints will be analyzed for the DLT-evaluable population.

Secondary efficacy endpoints for Phase 2 include ORR, DCR, DOR, and PFS. The Phase 2 efficacy endpoints will be analyzed for the efficacy population.

6.4.5.1 Objective Response Rate

ORR, on the basis of investigator assessment, is defined as the proportion of patients who attain a complete response (CR) or partial response (PR). Per RECIST v1.1, confirmation of objective response is not required for this secondary endpoint. Patients without a post-baseline tumor assessment will be considered non-responders, as well as patients with a best overall response of stable disease (SD), progressive disease (PD) or not evaluable (NE). For a patient to have a best overall response of SD, he/she must have at least one post-baseline tumor assessment of SD at least 7 weeks after treatment initiation (8 weeks minus 7-day window; study day 49).

A 95% confidence interval for the rate will be estimated for each treatment arm using the Clopper-Pearson method.

Spider plots and waterfall plots will be generated to visualize changes in the sum of target lesions.

6.4.5.2 Duration of Response

For patients who have experienced an objective response (CR or PR) during the study as assessed by the investigator, DOR is defined as the time from the first tumor assessment that documents response (CR or PR, whichever is recorded first) to first documentation of

radiographic PD per RECIST v1.1. Patients who have not progressed at the time of analysis will be censored at the last tumor assessment date prior to the start of subsequent systemic anti-cancer therapy. The Kaplan-Meier method will be used to estimate the median DOR for each treatment arm with 95% confidence limits.

6.4.5.3 Disease Control Rate

DCR is defined as the proportion of patients who achieve a best response of CR, PR, or SD. For a patient to have a best overall response of SD, he/she must have at least one post-baseline tumor assessment of SD at least 7 weeks after treatment initiation (8 weeks minus 7-day window; study day 49). Patients without a post-baseline tumor assessment will be considered non-responders, as well as patients with a best overall response of PD or NE. A 95% confidence interval for the rate will be estimated for each treatment arm using the Clopper-Pearson method.

DCR will also be estimated at 6 and 12 months. For these landmark analyses, DCR is defined as the proportion of patients who have a response of CR, PR, or SD at the first tumor assessment on or after the landmark time. Accounting for the Q8W (i.e. every 8 weeks) schedule and 7-day window for disease assessment, 6-month DCR will consider the first evaluable scan after study day 161 (24 weeks – 7 days) and 12-month DCR will consider the first evaluable scan after study day 329 (48 weeks – 7 days). Patients without an evaluable disease assessment after the landmark time will be considered non-responders.

6.4.5.4 Progression-free Survival

PFS is defined as the time from initiation of study therapy to date of first documented radiographic progression of disease or date of death due to any cause, whichever occurs first. PFS will be determined on the basis of investigator assessment of progression using RECIST v1.1. Patients who have not progressed or died at the time of analysis will be censored at the last tumor assessment date prior to the start of subsequent systemic anti-cancer therapy. Patients with no post-baseline tumor assessment will be censored at the date of study therapy initiation. The Kaplan-Meier method will be used to estimate the median PFS for each treatment arm with 95% confidence limits.

PFS will also be estimated at various landmark times (e.g. 6 and 12 months).

6.4.6 **Exploratory Efficacy Endpoints**

Exploratory endpoints defined in the protocol and listed in Section 9.1 are outside the scope of this SAP. Biomarker analyses will be detailed in a Translational Analysis Plan.

6.4.7 **Subgroup Analyses**

For each treatment arm, OS will be analyzed in the subset of patients in the efficacy population who remain on treatment for at least 3 cycles (i.e. receive at least one dose of any study drug

in Cycle 4) vs. those who remain on treatment for 3 cycles or less. This subset is in contrast to the efficacy population analyzed in the primary analysis. The 1-year OS rate and 1-sided 95% confidence interval will be calculated.

6.4.8 Sensitivity Analyses

The following sensitivity analyses will be performed:

- Efficacy analyses will be performed on the Phase 2 Efficacy Population, defined as all patients who were randomized in Phase 2 and received at least 1 dose of any study drug. This population similar to the Efficacy Population but excludes the 12 DLT-evaluable patients who were enrolled in Phase 1b at the recommended Phase 2 dose.
- If the number of patients dosed in Phase 1b does not match the DLT-evaluable population, ORR and DOR will be calculated for all patients who received at least one dose of study drug in Phase 1b (i.e. the safety population).
- If any Phase 2 patients were randomized but not dosed, Phase 2 primary and secondary efficacy endpoints may be analyzed using an intention-to-treat (ITT) approach. For these analyses, all randomized patients will be included, grouped according to the treatment assigned at randomization.
- The impact of response confirmation on ORR will be assessed by requiring confirmation of CR or PR at least 4 weeks after initial documentation.
- The effect of death on DOR will be assessed by defining DOR as the time from the first tumor assessment that documents response (CR or PR, whichever is recorded first) to first documentation of radiographic progressive disease or death, whichever occurs first. Patients who have not progressed or died at the time of analysis will be censored at the last tumor assessment date prior to the start of subsequent systemic anti-cancer therapy.

6.5 Safety Analysis

Safety will be assessed through the summary of adverse events (AEs), serious adverse events (SAEs), DLTs, laboratory test results (hematology and serum chemistry), vital signs, and physical examinations. This may include data collected at unscheduled visits, early termination visits, or follow-up visits.

The baseline value of any variable will be defined as the last available value recorded prior to the first administration of study medication. If multiple values are available at the same visit, the mean of the values will be used for analysis at that timepoint.

Safety outcomes will be summarized based on the safety population (see Section 6.1.1). Safety summaries will be presented by the treatment regimen actually received.

6.5.1 Exposure to Study Medication

The number of patients exposed to each study drug and the extent of exposure (as number of doses, cumulative dose received, and relative dose received) will be summarized using descriptive statistics. Relative dose received is defined as the total amount of each drug actually received divided by the amount of drug the patient would have been expected to receive had he/she received all planned doses without any dose modifications until the date of treatment discontinuation.

6.5.2 Adverse Events

All reported AEs will be coded using the latest version of the Medical Dictionary for Regulatory Activities (MedDRA) and graded using the NCI Common Terminology Criteria for Adverse Events (CTCAE) version 4.03.

A treatment-emergent adverse event (TEAEs) is defined as any new adverse event or any worsening of an existing condition with an onset date on or after the first dose of study drug. AEs with relationship to study drug of “Possibly”, “Probably”, “Definitely” or unknown (missing) will be considered treatment related.

For each patient and each AE, the worst severity/grade recorded will be attributed and used in the severity summaries.

Listings for all AEs, SAEs, and TEAEs will be presented by patient. Listings will include information regarding onset day/date, end day/date, duration, relationship to study drug, severity, action taken with study drug, whether concomitant medication was administered and outcome.

Summaries of TEAEs (overall, by system organ class and preferred term and by severity) by treatment arm will be provided for each of the following categories:

- AEs
- AEs by most extreme severity
- Treatment-related AEs
- SAEs
- Treatment-related SAEs
- AEs leading to discontinuation of study treatment
- DLTs
- AEs from patients enrolled in Phase II that meet the protocol-defined unacceptable toxicity criteria

The proportion of events that are resolved at the time of analysis will be presented. In addition, patient deaths and the primary cause of death will be listed, as well as any cases of pregnancy.

6.5.3 Laboratory Data

Descriptive summaries of clinical laboratory values at each timepoint, including changes from baseline, will be generated for hematology and chemistry parameters by treatment arm. The proportion of patients with values outside the normal upper and lower limit at each visit will be displayed.

In addition, select laboratory parameters (including, but not limited to, AST, ALT, alkaline phosphatase and total bilirubin) will be summarized by grade using the CTCAE grading scale.

Missing laboratory values will not be imputed. Analysis will only occur on observed values.

6.5.4 Vital Signs

Vital signs will be summarized by treatment arm using descriptive statistics including mean values and mean change from baseline.

Missing vital signs will not be imputed. Analysis will only occur on observed values.

6.6 Missing Data

6.6.1 Missing and Partial Missing Adverse Event Dates

If the AE start date is not a complete date, the following rules will be applied to determine whether the event is treatment emergent.

- If the start date is completely missing: The AE will be assumed to be treatment-emergent unless the AE stop date is earlier than the date of first dose of study drug.
- If the day part of the AE start date is missing: If the month and year of the start date are later than or equal to the month and year of the date of first dose of study drug, then the AE will be assumed to be treatment emergent. If the month and year of the stop date are earlier than the month and year of the date of first dose of study drug, then the event will be assumed to be non-treatment emergent.
- If the day and month of the start date are missing: If the year of the start date is later than or equal to year of the date of first dose of study drug, then the AE will be assumed to be treatment emergent. If the year of the stop date is earlier than the year of the date of first dose of study drug, then the event will be assumed to non-treatment emergent.

6.6.2 Missing and Partial Missing Death Dates

For death dates, the following conventions will be used for imputing partial dates:

- If only the day of the month is missing, the 1st of the month will be used to replace the missing day. The imputed date will be compared to the last known date alive plus 1 day, and the maximum will be considered as the death date.

- If the month or the year is missing, the death date will be imputed as the last known date alive plus 1 day.
- If the date is completely missing but the reason for death is present, the death date will be imputed as the last known date alive plus 1 day.

6.7 Interim Analyses

Given the hypothesis generating nature of this study, the Sponsor may conduct two interim analyses of safety and efficacy during the Phase 1b and Phase 2 portions of the study. These interim analyses are strictly meant to support decision making for future studies. No adaptations (e.g. sample size re-estimation, early stopping for futility or efficacy, dropping/adding arms, modifying dose levels) will be made to the study based on the interim results. The analysis will be performed and interpreted by members of the Sponsor study team and management. Full results will be shared with management of the pharmaceutical partners of this study (Apexigen and Bristol-Myers Squibb), as their input is critical for decision making and design of future studies using this treatment regimen. Safety and limited efficacy results will be shared with study Investigators because they are key contributors to the design of an in-development clinical trial that may use the treatment regimens being tested in this study. To limit potential bias, any interim efficacy data shared with Investigators while patients are still receiving study therapy will only be presented as graphical summaries (spider and/or waterfall plots of BOR) with cohort names/identifiers masked.

The first interim analysis will be conducted approximately 3 months after the last patient is randomized in the Phase 2 portion. The following endpoints will be summarized for the first interim analysis:

- Study conduct including enrollment, demographics, and disposition
- Adverse events
- Phase 1b efficacy endpoints: ORR, DCR, DOR, OS, PFS
- Phase 2 efficacy endpoints: ORR, DCR

The second interim analysis will be conducted approximately 8 months after the last patient is randomized in the Phase 2 portion. The following endpoints will be summarized for the second interim analysis:

- Study conduct including enrollment, demographics, and disposition
- Adverse events
- Phase 1b efficacy endpoints: ORR, DCR, DOR, OS, PFS

- Phase 2 efficacy endpoints: ORR, DCR, DOR, PFS

The Phase 2 primary endpoint of OS will not be analyzed for Phase 2 patients during either interim analysis. Due to the exploratory nature of this study, no control of type I error will be applied for any of the endpoints at the interim or final analysis.

7 DIFFERENCES COMPARED TO PROTOCOL

- Section 4.4 (Determination of Sample Size): The SAP provides additional justification for the Phase 1b sample size.
- Section 6.1.1(Safety Population): The SAP clarifies that for the safety analyses, patients will be analyzed according to the treatment actually received.
- Section 6.1.2 (DLT-evaluable Population): The SAP clarifies that the DLT-evaluable population is the population for analyses of Phase 1b efficacy and DLTs.
- Section 6.4.7 (Subgroup Analyses): The protocol states that OS will be analyzed in the subset of patients who remain on study for at least 6 weeks. The SAP modifies this subgroup to patients who remain on treatment for at least 3 cycles.
- Section 6.7 (Interim Analyses): The SAP provides justification for and details about the two optional interim analyses of efficacy data.

8 REFERENCES

ICH/FDA Guidance for Industry E9 Statistical Principles for Clinical Trials. U.S. Department of Health and Human Services, Food and Drug Administration, September 1998.

Von Hoff DD, Ervin T, Arena FP, et al. Increased survival in pancreatic cancer with nab-paclitaxel plus gemcitabine. N Engl J Med 2013;369(18):1691-703.

9 APPENDICES

9.1 Protocol Synopsis

**Title of Study:**	Open-label, Multicenter, Phase 1b/2 Clinical Study to Evaluate the Safety and Efficacy of CD40 Agonistic Monoclonal Antibody (APX005M) Administered Together with Gemcitabine and nab-Paclitaxel with or without PD-1 Blocking Antibody (Nivolumab) in Patients with Previously Untreated Metastatic Pancreatic Adenocarcinoma
**Protocol Number:**	PICI0002
**Phase of Development:**	1b/2
**Objectives:**	**Phase 1b:**<br>Primary:<br>1. To determine the feasibility, safety and dose-limiting toxicities (DLT) of each treatment cohort.<br>2. To determine the recommended Phase 2 dose (RP2D) of APX005M when combined with nab-paclitaxel (NP)/gemcitabine (Gem).<br>3. To determine the RP2D of APX005M when combined with nivolumab/NP/Gem.<br>Secondary:<br>1. To determine objective response (OR) and duration of response (DOR) of each treatment cohort.<br>Exploratory:<br>1. To assess the pharmacokinetics (PK) of APX005M in Cycles 1 to 4.<br>2. To assess immune pharmacodynamic effects of each treatment cohort, in both blood and tumor tissue.<br>**Phase 2:**<br>Primary:<br>1. To estimate overall survival (OS) of each treatment arm.<br>2. To compare 1-year OS rate of each treatment arm to the historical rate for NP/Gem.<br>Secondary:<br>1. To determine the objective response rate (ORR), disease control rate (DCR), DOR, and progression-free survival (PFS) of each treatment arm.<br>2. To further characterize the feasibility and safety of each treatment arm.<br>Exploratory:<br>1. To assess the PK of APX005M in Cycles 1 to 4 (Arms B and C).<br>2. To assess immune pharmacodynamic effects of each treatment arm, in both blood and tumor tissue.<br>3. To assess associations between immune biomarkers and clinical outcomes.<br>4. To evaluate baseline and on-treatment microbiome profiles.<br>5. To construct multivariable linear models to dissect the pharmacodynamic effects of APX005M and nivolumab on immune biomarkers.

<table>
<tr><td>Study Design:</td><td>This is a multicenter, open-label, Phase 1b/2 study to evaluate the immunotherapy agents APX005M and nivolumab, in combination with Gem and NP in patients with previously untreated metastatic pancreatic adenocarcinoma.

Phase 1b
In the Phase 1b portion of the study, the following 4 treatment cohorts will be evaluated for feasibility and safety:
B1: NP/Gem/APX005M 0.1 mg/kg
B2: NP/Gem/APX005M 0.3 mg/kg
C1: Nivolumab/NP/Gem/APX005M 0.1 mg/kg
C2: Nivolumab/NP/Gem/APX005M 0.3 mg/kg
Enrollment in Cohorts B2 and C1 may occur concurrently. Enrollment in Cohort C2 may begin once enrollment in Cohort C1 has been completed.
Each cohort in the Phase 1b portion of the study will include approximately 6 DLT-evaluable patients. A cohort corresponding to Arm A of Phase 2 (nivolumab/NP/Gem) will not be tested, since an external study is being conducted to confirm the safety of nivolumab in combination with NP/Gem.
DLT is defined as any Grade 3 or higher toxicity that is treatment-related but not related to the natural progression of the tumor and occurs during the DLT observation period.

Phase 2 (Randomized)
Patients will be randomized to one of three arms: Arm A1, Arm B2, or Arm C2 (shown below).
<u>Treatment arms:</u>
A1: Nivolumab/NP/Gem
B2: NP/Gem/APX005M 0.3 mg/kg
C2: Nivolumab/NP/Gem/APX005M 0.3 mg/kg

A total of approximately 93 patients will be randomized/enrolled in Phase 2 (35 Arm A1, 29 Arm B2, 29 Arm C2). Twelve DLT-evaluable patients from the Phase 1b study, enrolled at the RP2D of APX005M in Arm C (i.e., 6 patients on B2 and 6 patients on C2) will be included in the Phase 2 analysis. Thus, each arm will enroll 35 patients, for a total of approximately 105 patients. In the first step of randomization, 12 patients will be randomized in a 4:1:1 allocation to achieve balance in the total number of patients in each arm (since Arm A1 did not enroll patients in Phase 1b, more patients have to be allocated to Arm A1). Once the 12 patients are randomized, step 2 will randomize the remaining 81 patients in a 1:1:1 allocation.</td></tr>
</table>

Selection of Patients:	Main Inclusion Criteria:<br>1. Patient has histologically or cytologically documented diagnosis of pancreatic adenocarcinoma with metastatic disease. Locally advanced patients are not eligible.<br>2. Patient must have measurable disease by Response Evaluation Criteria in Solid Tumors (RECIST) v1.1.<br>3. Patients must be age 18 years or older.<br>4. Patients must have an Eastern Cooperative Oncology Group (ECOG) performance status of 0 or 1.<br>5. A baseline tumor tissue sample is mandatory for enrollment. If archival tumor tissue is not available, then a fresh tumor biopsy must be provided.<br>6. Patients must have the following laboratory values at Screening, without transfusions or growth factors, within 2 weeks of the first dose of investigational agents:<br>a. Absolute neutrophil count (ANC) ≥1.5 x 10^9/L (in absence of growth factor support)<br>b. Platelet count ≥150 x 10^9/L<br>c. Hemoglobin ≥9 g/dL(without transfusion support)<br>d. Serum creatinine ≤1.5 mg/dL, and creatinine clearance ⩾ 50 ml/min as measured by Cockcroft and Gault formula<br>e. Aspartate aminotransferase (AST) and alanine aminotransferase (ALT) ≤2.5 x institution's ULN for patients with no concurrent liver metastases, OR ≤5.0 x institution's ULN for patients with concurrent liver metastases<br>f. Total bilirubin ≤1.5 x ULN, except in patients with documented Gilbert's Syndrome who must have a total bilirubin ≤3 x ULN<br>7. Women of childbearing potential (WOCBP) must have a negative pregnancy test (serum or urine) within the 7 days prior to study drug administration, and a negative urine pregnancy test within the 3 days before the first study drug administration, or a negative serum pregnancy test within 24 hours before the first study drug administration.<br>8. WOCBP and male patients who are sexually active with WOCBP must agree before receiving the first dose of study drugs to use 2 highly effective methods of contraception (including a physical barrier) during the study and for 5 months for women and 7 months for men following the last dose of study drug, as described in the body of the protocol.<br>9. Patients must have the ability to understand and willingness to sign a written informed consent document.<br>Main Exclusion Criteria:<br>1. Patient must not have received any prior treatment, including chemotherapy, for metastatic pancreatic adenocarcinoma, with the following exceptions and notes:<br>a. Patients who have received prior adjuvant or neoadjuvant therapy for pancreatic adenocarcinoma are eligible if the last dose of adjuvant therapy was more than 4 months before the date of study entry. In this case, prior Gem and/or NP are allowable.<br>b. Prior resection surgery is allowable.<br>c. Patients initially diagnosed with locally advanced pancreatic cancer who have undergone chemotherapy then resection and were with no evidence of disease are eligible if metastatic relapse of disease has occurred and if the last dose of chemotherapy was more than 4 months before the date of study entry.

	2. Patients must not have another active invasive malignancy, with the following exceptions and notes:<br>a. History of a non-invasive malignancy, such as cervical cancer in situ, non-melanomatous carcinoma of the skin, in situ melanoma, or ductal carcinoma in situ of the breast, is allowed.<br>b. History of malignancy that is in complete remission after treatment with curative intent is allowed.<br>c. No current or history of a hematologic malignancy is allowed, including patients who have undergone a bone marrow transplant.<br>3. History of clinically significant sensitivity or allergy to monoclonal antibodies, their excipients or intravenous gamma globulin<br>4. Previous exposure to CD40, PD-1, PD-L1, CTLA-4 antibodies or any other immunomodulatory agent<br>5. History of (non-infectious) pneumonitis that required corticosteroids or current pneumonitis, or history of interstitial lung disease<br>6. Patients must not have a known or suspected history of an autoimmune disorder, including but not limited to inflammatory bowel disease, celiac disease, Wegner syndrome, Hashimoto syndrome, systemic lupus erythematosus, scleroderma, sarcoidosis, or autoimmune hepatitis, within 3 years of the first dose of investigational agent, except for the following:<br>a. Patients with Type 1 diabetes mellitus, hypothyroidism only requiring hormone replacement, skin disorders such as vitiligo, or alopecia not requiring systemic therapy, or conditions not expected to recur in the absence of an external trigger are eligible.<br>b. Patients with a history of Hashimoto syndrome within 3 years of the first dose of investigational agent, which resolved to hypothyroidism alone.<br>7. Patients must not have an uncontrolled intercurrent illness, including an ongoing or active infection, current pneumonitis, symptomatic congestive heart failure (New York Heart Association class III or IV), unstable angina, uncontrolled hypertension, cardiac arrhythmia, interstitial lung disease, active coagulopathy, or uncontrolled diabetes.<br>8. Patients must not have a history of myocardial infarction within 6 months or a history of arterial thromboembolic event within 3 months of the first dose of investigational agent.<br>9. Patients must not have a history of human immunodeficiency virus, hepatitis B (HB), or hepatitis C, except for the following:<br>a. Patients with anti-HB core antibody but with undetectable HB virus deoxyribonucleic acid (DNA) and negative for HB surface antigen<br>b. Patients with resolved or treated hepatitis C virus (HCV) (i.e. HCV antibody positive but undetectable HCV RNA)<br>10. Patients must not have a history of primary immunodeficiency.<br>11. Patients must not receive concurrent or prior use of an immunosuppressive agent within 14 days of the first dose of investigational agent, with the following exceptions and notes:<br>a. Systemic steroids at physiologic doses (equivalent to dose of 10 mg oral prednisone) are permitted. Steroids as anti-emetics for chemotherapy are not allowed.<br>b. Intranasal, inhaled, topical, intra-articular, and ocular corticosteroids with minimal systemic absorption are permitted.

	c. Patients with a condition with anticipated use of systemic steroids above the equivalent of 10 mg prednisone are excluded.<br>d. Transient courses of steroids may be approved by the Medical Monitor on a case by case basis, dependent on dose and reason.<br>12. Patients must not have a history of clinically manifested central nervous system (CNS) metastases.<br>a. Patients with known or suspected leptomeningeal disease or cord compression are not eligible.<br>13. Patients must not have had major surgery as determined by the PI within 4 weeks before the first dose of study drug.<br>14. Patients must not have received another investigational agent within the shorter of 4 weeks or 5 half-lives before the first dose of investigational agent.<br>15. Patients must not have received a live attenuated vaccine within 28 days before the first dose of investigational agent, and patients, if enrolled, should not receive live vaccines during the study or for 180 days after the last dose of investigational agent.<br>16. Females who are pregnant or lactating or who intend to become pregnant during participation in the study are not eligible to participate.<br>17. Patients who have any clinically significant psychiatric, social, or medical condition that, in the opinion of the investigator, could increase the patient's risk, interfere with protocol adherence, or affect the patient's ability to give informed consent are ineligible to participate in the study.
**Planned Sample Size:**	Up to 24 DLT-evaluable patients will be enrolled in the Phase 1b portion of the study. A total of approximately 93 patients will be randomized/enrolled in Phase 2. Thus, the total sample size is expected to be approximately 117 patients.
**Investigational Therapy:**	**Phase 1b:**<br>APX005M (0.1 or 0.3 mg/kg) in combination with NP (125 mg/m^2) and Gem (1000 mg/m^2), all administrated intravenously (IV)<br>**OR**<br>APX005M (0.1 or 0.3 mg/kg) in combination with nivolumab (240 mg), NP (125 mg/m^2) and Gem (1000 mg/m^2), all administered IV<br><br>**Phase 2:**<br>Nivolumab (240 mg) in combination with NP (125 mg/m^2) and Gem (1000 mg/m^2), all administered IV<br>**OR**<br>APX005M (0.3 mg/kg) in combination with NP (125 mg/m^2) and Gem (1000 mg/m^2), all administrated IV<br>**OR**<br>APX005M (0.3 mg/kg) in combination with nivolumab (240 mg), NP (125 mg/m^2) and Gem (1000 mg/m^2), all administered IV

**Treatment Duration:**	Assuming all 4 cohorts are tested, upon completion of enrollment to Phase 1b, 1 additional month of follow-up will occur before declaring the RP2D of APX005M. Then the Phase 2 portion of the study will have 12 additional months of follow-up. Target enrollment completion is within 24 months; however, enrollment will proceed until met or as determined by the study sponsor (Parker Institute for Cancer Immunotherapy [PICI]). Considering several months for data management and statistical analysis, the total duration of the study is likely to be 5 years.<br>Patients will undergo screening and, if eligible, will undergo treatment in the assigned arm of the study until unacceptable toxicity, progression of disease, or withdrawal of consent. All patients will be followed for survival status until death or a maximum of 5 years.
**Study Endpoints:**	**Phase 1b:**<br>Primary:<br>• The frequency of DLT<br>• The RP2D of APX005M when combined with NP/Gem or nivolumab/NP/Gem<br>• The incidence of treatment-emergent AEs (TEAEs), serious adverse events (SAEs), and adverse events (AEs) causing discontinuation<br>Secondary:<br>• OR is determined by RECIST v1.1<br>• DOR is defined as the time from first documentation of response (complete response [CR] or partial response [PR]) to first documentation of progressive disease (PD)<br>Exploratory:<br>• PK of APX005M will be determined in Cycles 1 to 4.<br>• Immune pharmacodynamic endpoints may include, but are not limited to, the following:<br>o Changes in the tumor microenvironment (including cellularity, stromal content, cellular infiltration, and tumor apoptosis) may be assessed by tumor multiplex immunohistochemistry or other appropriate technology. Pharmacodynamic and PK parameters, if available, may be used to influence the RP2D.<br>o Gene expression may be determined by tumor RNA sequencing, peripheral blood RNA sequencing, or other appropriate technology. Other sequencing technologies, such as ATAC sequencing, may be performed.<br>o Tumor genomics may be determined when possible by Clinical Laboratory Improvement Amendment-certified mutational panel assessments and/or by whole exome sequencing.<br>o For variant calling and human leukocyte antigen (HLA) determination, normal tissue whole exome sequencing may be performed. In some cases, data regarding germ-line BRCA1/2 mutations or microsatellite instability may be incorporated into analyses.<br>o Cytokine and/or circulating factor analysis may be determined by a multiplex assay or other appropriate technology.<br>o Flow cytometry or other related technologies, such as CyTof analysis of peripheral blood, may be used to assess phenotype, function, and other changes in immune cellular subsets.<br>o Other markers to measure tumor burden, including circulating tumor DNA, tumor cells, and protein markers, may be measured in an exploratory fashion if material is available

	**Phase 2:**<br>Primary:<br>• OS is defined as the time from initiation of study therapy to date of death due to any cause or date of most recent patient contact. Patients who have not died are censored on their most recent contact date.<br>• 1-year OS rate in each treatment arm.<br>Secondary:<br>• Investigators' assessment of OR is determined by RECIST v1.1 and the ORR is defined as the proportion of patients who achieve a CR or PR.<br>• DCR is defined as the proportion of patients who achieve a CR or PR or SD.<br>• DOR is defined as the time from first documentation of response (CR or PR) to first documentation of PD.<br>• PFS is defined as the time from initiation of study therapy to date of first documented progression of disease, date of death due to any cause or date of most recent patient contact which documented progression-free status (i.e., clinic visit date or scan date). Patients who have not progressed or died are censored on their most recent progression-free date.<br>• The incidence of AEs defined as unacceptable toxicities in Phase 2, TEAEs, SAEs, and AEs causing treatment discontinuation<br>• Clinical laboratory data and vital signs (descriptive statistics) and numbers of patients with values outside limits of the normal range at each time point.<br>Exploratory:<br>▪ The exploratory endpoints for Phase 2 are the same as those described above for Phase 1b with the addition of evaluation of baseline and on-treatment microbiome profiles with treatment outcomes.
**Statistical Methods and Planned Analyses:**	This is a multi-center, open-label Phase 1b/2 study of CD40 agonistic monoclonal antibody, APX005M, and/or PD-1 blocking antibody, nivolumab, in combination with NP and Gem and for patients with newly diagnosed metastatic pancreatic cancer. The primary objectives of the Phase 1b study are to determine the feasibility, safety and DLT of each treatment cohort and to determine the RP2D of APX005M in combination with NP/Gem and with nivolumab/NP/Gem. The primary objective of the randomized Phase 2 study is to evaluate OS in three treatment arms: nivolumab/NP/Gem, NP/Gem/APX005M and nivolumab/NP/Gem/APX005M by comparing the 1-year OS rate with the historical value for NP/Gem.<br>**The safety population** consists of all patients who received at least 1 dose of any study drug. This is the population for the primary analyses of safety.<br>**The DLT-evaluable population** consists of patients who received 2 or 3 doses of NP/Gem and 1 dose of APX005M during Cycle 1, thus have completed the DLT observation period (ie, from the time of first administration of study drugs until prior to Cycle 2 Day 1). Patients who do not meet these criteria will be replaced in Phase 1b only, to assist with DLT and RP2D decision-making.<br>**The efficacy population** consists of (1) all patients who were randomized/enrolled in Phase 2 and received at least 1 dose of any study drug and (2) the 12 DLT-evaluable patients (6 on Arm B and 6 on Arm C) who were enrolled in Phase 1b at the RP2D. The efficacy population is the population for the primary analyses of efficacy.<br>**Phase 1b Design:** Four treatment cohorts will be evaluated for feasibility and safety. Cohorts B1 and B2 will escalate the dose of APX005M when combined with NP/Gem, and then Cohorts C1 and C2 will escalate the dose of APX005M

when combined with nivolumab/NP/Gem. Enrollment in Cohorts B2 and C1 may occur concurrently. Enrollment in Cohort C2 may begin once enrollment in Cohort C1 has been completed. Approximately 6 DLT-evaluable patients will be enrolled in each cohort. A1 (nivolumab/NP/Gem) will not be tested in Phase 1b, since an external study is being conducted to confirm the safety of nivolumab in combination with NP/Gem.

Statistical analyses will include the following:

- The number of patients treated in each cohort will be reported, and reasons why any patient is not DLT evaluable will be summarized.
- Approximately 6 DLT-evaluable patients will be fully analyzed in each treatment cohort.
- Feasibility issues will be described for each treatment cohort.
- Toxicities will be graded by NCI-CTCAE, causality attributed, and tabulated by treatment cohort.
- RP2D of APX005M when combined with NP/Gem and with nivolumab/NP/Gem will be determined.
- RECIST OR will be scored and tabulated along with DOR, by treatment cohort.
- PK of APX005M.
- Immune pharmacodynamic effects will be measured, including change from baseline, and reported by treatment cohort.

Phase 2 Design: Once the RP2D of APX005M has been defined, the randomized Phase 2 portion of the study will commence. Patients will be randomized to one of 3 arms, defined by the addition of one or more immunotherapy agents to standard of care NP/Gem. The arms will be either A1 vs B2 vs C2 or A1 vs B1 vs C1. Note that the APX005M dose must be the same in Arms B and C, regardless of whether a higher APX005M dose was determined to be safe in Arm B. For each regimen, efficacy will be evaluated by comparing the 1-year overall survival (OS) rate to the historical value for NP/Gem.

Statistical analyses will include the following:

- Thirty-five patients will be analyzed on each treatment arm. Twelve DLT-evaluable patients from Phase 1b (Arms B and C) and 93 patients from Phase 2 will comprise the population for the final analysis of efficacy.
- OS will be estimated by the Kaplan-Meier method for each treatment arm.
- The 1-year OS rate and 1-sided 95% confidence interval (CI) will be calculated for each treatment arm, to determine whether the lower bound of the CI excludes the historical value for NP/Gem. A 1-sided one-sample Z test will also be conducted. The goal is to compare the survival probability at 1-year to the historical value of 0.35.
- ORR and DCR and their 95% CIs will be calculated for each treatment arm.
- PFS will be estimated by the Kaplan-Meier method for each treatment arm.
- DOR will be calculated from dates of first documented response and progression of disease.
- Toxicities will be graded by CTCAE v4.03 and tabulated by treatment arm.
- PK of APX005M in Cycles 1 to 4 (Arms B and C).
- Immune pharmacodynamic effects may be measured, including but not limited to change from baseline, and reported by treatment arm.
- Test of associations between immune biomarkers and clinical outcomes

	• Construct multivariable linear models to dissect the pharmacodynamic effects of APX005M and nivolumab on immune biomarkers.
