# Supplementary material for: Sotigalimab and/or nivolumab with chemotherapy in first-line metastatic pancreatic cancer: clinical and immunologic analyses from the randomized phase 2 PRINCE trial
Source: Nat Med. 2022 Jun 3;28(6):1167–77. doi: 10.1038/s41591-022-01829-9 (PMC9205784; doi:10.1038/s41591-022-01829-9)
Supplement: Supplementary file 2 — Reporting Summary [file 41591_2022_1829_MOESM2_ESM.pdf]

nature portfolio

Corresponding author(s): Vonderheide RH and Padron LJ

Last updated by author(s): Apr 11, 2022

Reporting Summary

Nature Portfolio wishes to improve the reproducibility of the work that we publish. This form provides structure for consistency and transparency in reporting. For further information on Nature Portfolio policies, see our Editorial Policies and the Editorial Policy Checklist.

Statistics

For all statistical analyses, confirm that the following items are present in the figure legend, table legend, main text, or Methods section.

n/a	Confirmed	
☐	☒	The exact sample size (*n*) for each experimental group/condition, given as a discrete number and unit of measurement
☐	☒	A statement on whether measurements were taken from distinct samples or whether the same sample was measured repeatedly
☐	☒	The statistical test(s) used AND whether they are one- or two-sided<br>*Only common tests should be described solely by name; describe more complex techniques in the Methods section.*
☐	☒	A description of all covariates tested
☐	☒	A description of any assumptions or corrections, such as tests of normality and adjustment for multiple comparisons
☐	☒	A full description of the statistical parameters including central tendency (e.g. means) or other basic estimates (e.g. regression coefficient) AND variation (e.g. standard deviation) or associated estimates of uncertainty (e.g. confidence intervals)
☐	☒	For null hypothesis testing, the test statistic (e.g. *F*, *t*, *r*) with confidence intervals, effect sizes, degrees of freedom and *P* value noted<br>*Give P values as exact values whenever suitable.*
☒	☐	For Bayesian analysis, information on the choice of priors and Markov chain Monte Carlo settings
☒	☐	For hierarchical and complex designs, identification of the appropriate level for tests and full reporting of outcomes
☒	☐	Estimates of effect sizes (e.g. Cohen's *d*, Pearson's *r*), indicating how they were calculated

Our web collection on statistics for biologists contains articles on many of the points above.

Software and code

Policy information about availability of computer code

Data collection	Clinical data was collected in Medidata Rave EDC.<br>Mass cytometry samples were acquired on a Helios CyTOF instrument (Fluidigm).<br>Flow cytometry data were acquired on a BD Symphony A5 cytometer (BD Biosciences).<br>Serum proteins were quantified using Olink multiplex proximity extension assay (PEA) panels (Olink Proteomics; www.olink.com) according to the manufacturer's instructions.<br>For whole exome and transcriptome sequencing, FFPE tumor and normal PBMC samples were profiled using ImmunoID NeXT (Personalis, Inc., Menlo Park, CA, USA), an augmented exome/transcriptome platform and analysis pipeline. Paired-end sequencing was performed on NovaSeq instrumentation (Illumina, San Diego, CA, USA).<br>For multispectral imaging, seven color multiplex-stained slides were imaged using the Vectra Multispectral Imaging System version 3 (Akoya).
Data analysis	Clinical data analyses: R version 4.1.0.<br>Flow and mass cytometry data were analyzed using CellEngine™ version 1 cloud-based flow cytometry analysis software (CellCarta, Montreal, Quebec, Canada).<br>Raw mass spectrometric data were analyzed using the software Spectronaut (version SM 2.8.210609.47784, Biognosys) with the default settings, but Qvalue sparse filtering was enabled with a global imputing strategy and a hybrid library comprising all DIA and DDA runs conducted in this study.<br>The images acquired from multiplex immunofluorescent imaging were analyzed using the CellEngine™ software (CellCarta) alongside Mantis Viewer, a custom in-house open-source software used for fluorescent image visualization (http://doi.org/10.5281/zenodo.4009579).<br>All molecular data was analyzed for association with outcomes and treatment using the R programming language (version 4.0.5) with the packages and versions listed in Supplementary Table 15. Association with survival was analyzed for cell population percentages, protein values, and gene expression signatures by separating patients into two groups based on the median value across all patients in all cohorts. Kaplan-Meier plots were created and log-rank p-value significance was determined using the survminer (v. 0.4.9) and survival (v. 3.2-13) packages. To visualize differences between any defined groups or visualize changes on treatment, ggplot2 (v. 3.3.5) and base R plotting were used. To determine differences between pretreatment and on-treatment values as well as differences between survival groups (>1 year and

<1 year) at any given timepoint, a two-sided Wilcoxon sign-rank test with a significance cutoff of p=0.05 was used. Median log fold change was calculated to determine additional pharmacodynamic differences seen from pretreatment to on-treatment. Circus plots for multi-omic analysis were generated using the DIABLO method in the mixOmics (v. 6.16.3) R package. Heatmaps were generated using pheatmap (v. 1.0.12) and correlations across data types were calculated using the Spearman method. Cox proportional hazard multivariable models were also generated in relation to survival in each arm with individual biomarkers in Supplementary Table 9 with an additional clinical variate, de novo/recurrent staging at initial diagnosis or prior chemotherapy usage, using the survival and survminer packages. Forest plots were generated for the most significant circulating biomarkers in each arm to determine hazard ratio, and confidence interval of each biomarker in relation to each other.
Additional R packages used include wick v. 1.1, dplyr v. 1.0.7, plyr v. 1.8.6, Reshape2 v. 1.4.4, Data.table v. 1.14.0, tidyr v. 1.1.4, tidyverse v. 1.3.1, ggpubr v. 0.4.0, limma v. 3.48.3, readxl v. 1.3.1, msigdbr v. 7.4.1, stringr v. 1.4.0, venn v. 1.10, and reader v. 2.0.1.

For manuscripts utilizing custom algorithms or software that are central to the research but not yet described in published literature, software must be made available to editors and reviewers. We strongly encourage code deposition in a community repository (e.g. GitHub). See the Nature Portfolio guidelines for submitting code & software for further information.

Data

Policy information about availability of data

All manuscripts must include a data availability statement. This statement should provide the following information, where applicable:

- Accession codes, unique identifiers, or web links for publicly available datasets
- A description of any restrictions on data availability
- For clinical datasets or third party data, please ensure that the statement adheres to our policy

Summary datasets generated during and/or analyzed during the current study are available in the Sequence Read Archive under BioProject ID PRJNA789990. These datasets include a de-identified limited clinical dataset with demographic and response information for each patient, raw RNA and DNA sequencing files, and summary tables of cell proportions found via mIF, CyTOF, and flow cytometry. The full clinical dataset generated in this study is considered commercially sensitive and, therefore, is not publicly available. Requests for additional clinical data should be emailed to the corresponding author and should include a brief description of the proposed analysis. Requests for data access will be reviewed individually and a decision will be communicated within 4 weeks of receipt. Data might be shared in the form of aggregate data summaries and via a data transfer agreement, which will outline any potential restrictions on data use. Individual patient-level raw data containing confidential or identifiable patient information are subject to patient privacy and cannot be shared.

Field-specific reporting

Please select the one below that is the best fit for your research. If you are not sure, read the appropriate sections before making your selection.

☒ Life sciences ☐ Behavioural & social sciences ☐ Ecological, evolutionary & environmental sciences

For a reference copy of the document with all sections, see nature.com/documents/nr-reporting-summary-flat.pdf

Life sciences study design

All studies must disclose on these points even when the disclosure is negative.

Sample size	The null hypothesis was a 1-year OS rate of 35% and the alternative hypothesis was a 1-year OS rate of 55%. Planned enrollment was 105 patients (35 per arm), which included 12 DLT-evaluable patients from the non-randomized Phase Ib. A sample size of 35 patients per arm provided 81% power to test this hypothesis, using a 1-sample Z test of the Kaplan-Meier estimate of the 1-year OS rate (and its standard error) with 1-sided 5% type I error rate.
Data exclusions	Six patients were randomized but not dosed and were excluded from analysis of safety and efficacy data.
Replication	This was a prospectively designed Phase 2 clinical trial. Due to the limited sample size and available samples, none of the experiments described in the Results were replicated. Replication of clinical and related translational experiments will require a new clinical trial.
Randomization	Patients were randomly assigned to one of three arms: nivo/chemo, sotiga/chemo, or sotiga/nivo/chemo. Twelve dose limiting toxicity (DLT)-evaluable patients (6 each on sotiga/chemo and sotiga/nivo/chemo) from the non-randomized Phase 1b study were included in analyses of Phase 2 efficacy. To achieve balance in the total number of patients enrolled in each arm, the first 12 patients enrolled in Phase 2 were randomly allocated in a 4:1:1 ratio to nivo/chemo, sotiga/chemo or sotiga/nivo/chemo, respectively (because nivo/chemo did not accrue patients in Phase 1b, more patients needed to be enrolled in that arm). The remaining patients were randomly allocated in a 1:1:1 ratio. Randomization was managed by the Parker Institute for Cancer Immunotherapy using Medidata RTSM, an interactive voice/web response system (IxRS). A permuted block design, without stratification by baseline patient or tumor characteristics, was used for randomization. Patients who were randomized but did not receive any study drug were replaced via randomization of additional patients.
Blinding	This trial was open label with no blinding.

Reporting for specific materials, systems and methods

We require information from authors about some types of materials, experimental systems and methods used in many studies. Here, indicate whether each material, system or method listed is relevant to your study. If you are not sure if a list item applies to your research, read the appropriate section before selecting a response.

Materials & experimental systems

n/a	Involved in the study
☐	☒ Antibodies
☒	☐ Eukaryotic cell lines
☒	☐ Palaeontology and archaeology
☒	☐ Animals and other organisms
☐	☒ Human research participants
☐	☒ Clinical data
☒	☐ Dual use research of concern

Methods

n/a	Involved in the study
☒	☐ ChIP-seq
☐	☒ Flow cytometry
☒	☐ MRI-based neuroimaging

Antibodies

Antibodies used

The antibodies used for mass cytometry and flow cytometry are detailed in Supplemental Table 11 (CyTOF Antibody Panel) and Supplementary Table 12 (T cell Phenotyping Antibody Panel (X50)). Antibodies used for multiplex imaging staining panels are detailed in Supplementary Table 13. Serum proteomics analysis was performed using Olink Target 96 Immuno-Oncology and Immune Response panels as a paid service by Olink. Specific antibody clones were not disclosed. Specific lot number information is not available.
Optimized concentrations/dilutions for antibodies used in CyTOF experiments were: CD45, CD3, CD19, CD117, CD11b, CD4, CD8a, CD11c, CD14, FcER1, CD123, gdTCR, CD45RA, CD366, CD274, CD27, Tbet, CD152, FoxP3, CD33, CD45RO, CD127, CD197, Ki67, CD25, TCRVa24-Ja18, CD38, HLA-DR, CD56, CD16 (all used at 1:100 per manufacturer's recommendation); CD66d, 3 ug/mL; CD7, 3 ug/mL; CD86, 6 ug/mL; CD1c, 3 ug/mL; CD64, 6 ug/mL; CD206, 3 ug/mL; CD141, 3 ug/mL; CD154, 3 ug/mL; CD40, 1.5 ug/mL; CD192, 6 ug/mL; nivolumab, 1 ug/mL; anti-human IgG4, 1 ug/mL.

Optimized concentrations/dilutions for antibodies used in the high parameter flow cytometry experiments were: CD45RA, 1:200; CD8a, 1:160; CD185, 1:400; CD25, 1:200; CD226, 1:65; CD27, 1:500; CD4, 1:800; CD197, 1:40; CD223, 1:100; CD14, 1:40; CD19, 1:160; CD41a, 1:260; CD3, 1:65; CD137, 1:100; CD244, 1:20; CD366, 1:200; CD39, 1:100; CD28, 1:100; CD278, 1:100; CD127, 1:160; CD38, 1:160; TIGIT, 1:40; Eomes, 1:100; CD152, 1:400; FoxP3, 1:400; T-bet, 1:600; TCF1, 1:125; Ki67, 1:600; KLRG1, 1:100; nivolumab, 1 mg/mL; anti-human IgG4, 1:200.

Opal polymer HRP Ms + Rb, 1X ready to use, Akoya Biosciences SKU# ARH1001EA
Goat anti-Mouse Poly HRP, 1X ready to use, Invitrogen Cat. No. B40961
Goat anti-Rabbit Poly HRP, 1X ready to use, Invitrogen Cat. No. B40962

Validation

Each primary antibody used for flow or mass cytometry are widely used and validated by the manufacturer. Antibodies are tested by immunofluorescent staining with flow cytometric analysis on human peripheral blood mononuclear cells, lymphocytes, and PHA-stimulated (day 3) peripheral blood lymphocytes. Antibody panel validation was performed by carefully titrating each individual antibody and running fluorescence minus one (FMO) or fluorescence minus many (FMM) control stains on several healthy donor PBMC or PHA-activated PBMC. Antibodies used for multiplex immunofluorescent analyses were validated by the manufacturers for immunohistochemistry (IHC). Additionally, equivalency of single-marker optimized antibody IHC developed with 3, 3'-diaminobendidine (DAB) on human tonsil tissue was demonstrated with corresponding multiplexed immunofluorescence (mIF) on tonsil.
For Olink proteomics panels, additional details about the 172 markers, detection range, data normalization and standardization are available at https://www.olink.com/resources-support/document-download-center/

Human research participants

Policy information about studies involving human research participants

Population characteristics

Patients ≥18 years of age with mPDAC were enrolled. Inclusion and exclusion criteria were identical for the Phase 1b and Phase 2 portions of the study. Prior treatment for metastatic disease was not allowed, though prior adjuvant and neoadjuvant chemo/radiotherapy was allowed if completed > 4 months prior to enrollment. Patients were required to have archival or fresh tumor specimens available before treatment or be able to undergo a biopsy to acquire tissue. Additional key eligibility criteria included Eastern Cooperative Oncology Group (ECOG) performance status score of 0-1, adequate organ function, and the presence of at least one measurable lesion per Response Evaluation Criteria in Solid Tumors version 1.1 (RECIST version 1.1). Patients were excluded if they had previous exposure to agonistic CD40, anti-PD-1, anti-PD-L1 monoclonal antibodies, or any other immunomodulatory anticancer agent. Patients were also excluded if they had ongoing or recent autoimmune disease requiring systemic immunosuppressive therapy, had undergone solid-organ transplantation, or had a concurrent cancer, unless indolent or not considered to be life-threatening (e.g., basal-cell carcinoma). Patient characteristics observed in this study are provided in Table 1, Supplementary Table 1 and Supplementary Table 2.

Recruitment

Patients were recruited via in-hospital or community clinic referral to one of 7 participating academic hospitals in the US. Self-selection bias is unlikely to have any meaningful impact on study results. Patients were not compensated for their participation in this trial. Patient recruitment for the randomized Phase 2 portion of the PRINCE study was competitive, utilizing Medidata RTSM, an IxRS system, for Phase 2 randomization. From August 30, 2018, through June 10, 2019, 99 patients were randomly allocated into one of three treatment arms.

Ethics oversight

The protocol and all amendments were approved by the lead Institutional Review Board at the University of Pennsylvania and were accepted at all participating sites.

Note that full information on the approval of the study protocol must also be provided in the manuscript.

Clinical data

Policy information about clinical studies

All manuscripts should comply with the ICMJE guidelines for publication of clinical research and a completed CONSORT checklist must be included with all submissions.

Clinical trial registration	NCT03214250
Study protocol	The study protocol is uploaded as a supplementary file.
Data collection	From August 30, 2018, through June 10, 2019, 99 patients were randomly allocated into one of three treatment arms. In this Phase 1b/2 study, patients ≥18 years of age with mPDAC were enrolled from 7 academic hospitals in the US which are part of the Parker Institute for Cancer Immunotherapy pancreas cancer consortium. Data presented in this manuscript was collected between August 3, 2017 (first patient screened in Phase 1b) and March 24, 2021 (clinical cutoff date).
Outcomes	The primary endpoint was the 1-year OS rate of each treatment arm, compared to the historical rate of 35% for gemcitabine/nab-paclitaxel. Secondary endpoints were progression-free survival (PFS), duration of response (DOR), objective response rate (ORR), disease control rate (DCR), and the incidence of adverse events. Key exploratory endpoints included the evaluation of immune pharmacodynamic (PD) effects and tumor and immune biomarker analyses.<br>Patients were assessed radiographically every 8 weeks for the first year and every 3 months thereafter, regardless of dose delays. Disease assessments were collected until radiographic progression or initiation of subsequent therapy, whichever occurred first. Patients were subsequently followed for survival.

Flow Cytometry

Plots

Confirm that:

☒ The axis labels state the marker and fluorochrome used (e.g. CD4-FITC).

☒ The axis scales are clearly visible. Include numbers along axes only for bottom left plot of group (a 'group' is an analysis of identical markers).

☒ All plots are contour plots with outliers or pseudocolor plots.

☒ A numerical value for number of cells or percentage (with statistics) is provided.

Methodology

Sample preparation	Blood samples for isolation of peripheral blood mononuclear cells (PBMC) were collected longitudinally at participating clinical sites, shipped overnight and processed at a central location (Infinity Biologix, Piscataway, NJ, USA) over a Ficoll gradient and cryopreserved in 90% Human Serum Type AB with 10% DMSO at 5 million PBMC/mL overnight at -80C and subsequently stored in liquid nitrogen vapor phase long term storage.
Instrument	Mass cytometry samples were acquired on a Helios CyTOF instrument (Fluidigm). Flow cytometry data were acquired on a BD Symphony A5 cytometer (BD Biosciences).
Software	Flow and mass cytometry data were analyzed using CellEngine™ cloud-based flow cytometry analysis software (CellCarta, Montreal, Quebec, Canada).
Cell population abundance	For mass cytometry 300,000-400,000 events were collected. The range of live DNA+ events analyzed was between 100,000-350,000 events. For fluorescence cytometry, 300,000-3 million events were collected. The range of live singlets analyzed was between 300,000-1.5 million events.
Gating strategy	Mass cytometry acquired raw FCS files were normalized with the preloaded normalizer algorithm on CyTOF software using the EQ beads spiked into each sample Normalized CyTOF FCS files were manually gated for different populations to create two-dimensional plots. Cell debris and doublets were manually removed by gating on the residual and offset parameters (native to the acquisition software). Live DNA+ events were gated using Iridium-positive and cisplatin-negative events, followed by selecting on CD45+ cells. Populations were then defined based on known lineage combinations of cell surface markers. For manual gating on biaxial plots, the positive population of each marker was defined as the events above the negative population on the same plot. For fluorescence cytometry acquired FCS files, CD3+ T cell populations were defined after gating for lymphocytes (by FSC-A/SSC-A) and excluding debris followed by gating on viable CD3+ T cells (CD3+CD14-CD19-CD41a-Live/Dead-) using a lineage dump gate and exclusion of live/dead fixable dye.<br>Supervised gating for cytometry was performed manually by scientists blinded to clinical outcome. High level gates were tailored per patient across all time points. Single marker gates were drawn uniformly for analysis across patients and time points, with example gating strategy provided in Supplemental Figure 6 (CyTOF gating) and Supplemental Figure 2 (T cell X50 gating).

☒ Tick this box to confirm that a figure exemplifying the gating strategy is provided in the Supplementary Information.
